# The denatured state of HIV‐1 protease under native conditions

**DOI:** 10.1002/prot.26189

**Published:** 2021-08-03

**Authors:** Heike I. Rösner, Martina Caldarini, Gregory Potel, Daniel Malmodin, Maria A. Vanoni, Alessandro Aliverti, Ricardo A. Broglia, Birthe B. Kragelund, Guido Tiana

**Affiliations:** ^1^ BRIC University of Copenhagen Copenhagen N Denmark; ^2^ Structural Biology and NMR Laboratory (SBiNlab), Department of Biology University of Copenhagen Copenhagen N Denmark; ^3^ Department of Physics Università degli Studi di Milano and INFN Milan Italy; ^4^ Lawrence Livermore National Laboratory Livermore California USA; ^5^ Dipartimento di Bioscienze Università degli Studi di Milano Milan Italy; ^6^ Niels Bohr Institutet University of Copenhagen Copenhagen Ø Denmark; ^7^ Center for Complexity and Biosystems Università degli Studi di Milano Milan Italy

**Keywords:** advanced molecular dynamics, denatured state, NMR

## Abstract

The denatured state of several proteins has been shown to display transient structures that are relevant for folding, stability, and aggregation. To detect them by nuclear magnetic resonance (NMR) spectroscopy, the denatured state must be stabilized by chemical agents or changes in temperature. This makes the environment different from that experienced in biologically relevant processes. Using high‐resolution heteronuclear NMR spectroscopy, we have characterized several denatured states of a monomeric variant of HIV‐1 protease, which is natively structured in water, induced by different concentrations of urea, guanidinium chloride, and acetic acid. We have extrapolated the chemical shifts and the relaxation parameters to the denaturant‐free denatured state at native conditions, showing that they converge to the same values. Subsequently, we characterized the conformational properties of this biologically relevant denatured state under native conditions by advanced molecular dynamics simulations and validated the results by comparison to experimental data. We show that the denatured state of HIV‐1 protease under native conditions displays rich patterns of transient native and non‐native structures, which could be of relevance to its guidance through a complex folding process.

## 1 INTRODUCTION

The denatured state *D*_0_ that proteins populate transiently under native conditions^1^ is important to determine their folding,^2^ stability,^3^ aggregation,^4^ and misfolding;^5^ properties that can have direct implication for disease states. Except for a few specific proteins,^6^, ^7^, ^8^ *D*_0_ is so poorly populated that it escapes experimental observation. To overcome this problem, induced denatured states can be stabilized by chemical agents like urea, guanidine hydrochloride (GdmCl) or acids, populating the states *D*_urea_, *D*_GdmCl_, and *D*_acid_, respectively; states that are not necessarily similar to *D*_0_ and which show variation among themselves. However, from a thermodynamic point of view, calorimetry experiments^9^ showed that the unfolding enthalpy of lysozyme, denatured by acid, GdmCl, and temperature, is identical once the energy associated with the denaturant mean (e.g., the ionization energy in the case of pH) was subtracted. From these data, it was concluded that the states denatured by different means are thermodynamically indistinguishable.^9^

One could then ask whether the conformational properties of the different denatured states *D*_urea_, *D*_GdmCl_, *D*_acid_, and *D*_0_ are similar as well. Although these states were originally believed to be randomly disordered,^10^ recent studies have revealed them to contain transient secondary^11^, ^12^, ^13^, ^14^, ^15^ and even tertiary structures.^16^, ^17^ Such results were made possible mainly thanks to the development of NMR techniques and in particular of secondary chemical shift analysis.

In the present work, we studied the denatured states of a monomeric variant of human immunodeficiency virus (HIV)‐1 protease* (mHIV‐1‐PR_1–95_), a protein necessary for HIV‐1 to replicate in infected cells.^18^ The denatured state of HIV‐1 protease under native conditions is particularly important because it was suggested as a possible target of antiretroviral drugs that prevent the correct folding of the protein and thus of its enzymatic activity.^19^, ^20^, ^21^ Moreover, the native conformation of mHIV‐1‐PR_1–95_ displays a topology, which is more complex than that of typical proteins of comparable size, a feature possibly encoded also in its denatured state. In fact, its native conformation displays two pseudo‐knots and the associated Plaxco's contact order,^22^ quantifying the nonlocality of native contacts, is 15, much larger than the values 8–10 of typical proteins of comparable length.

**FIGURE 1:**
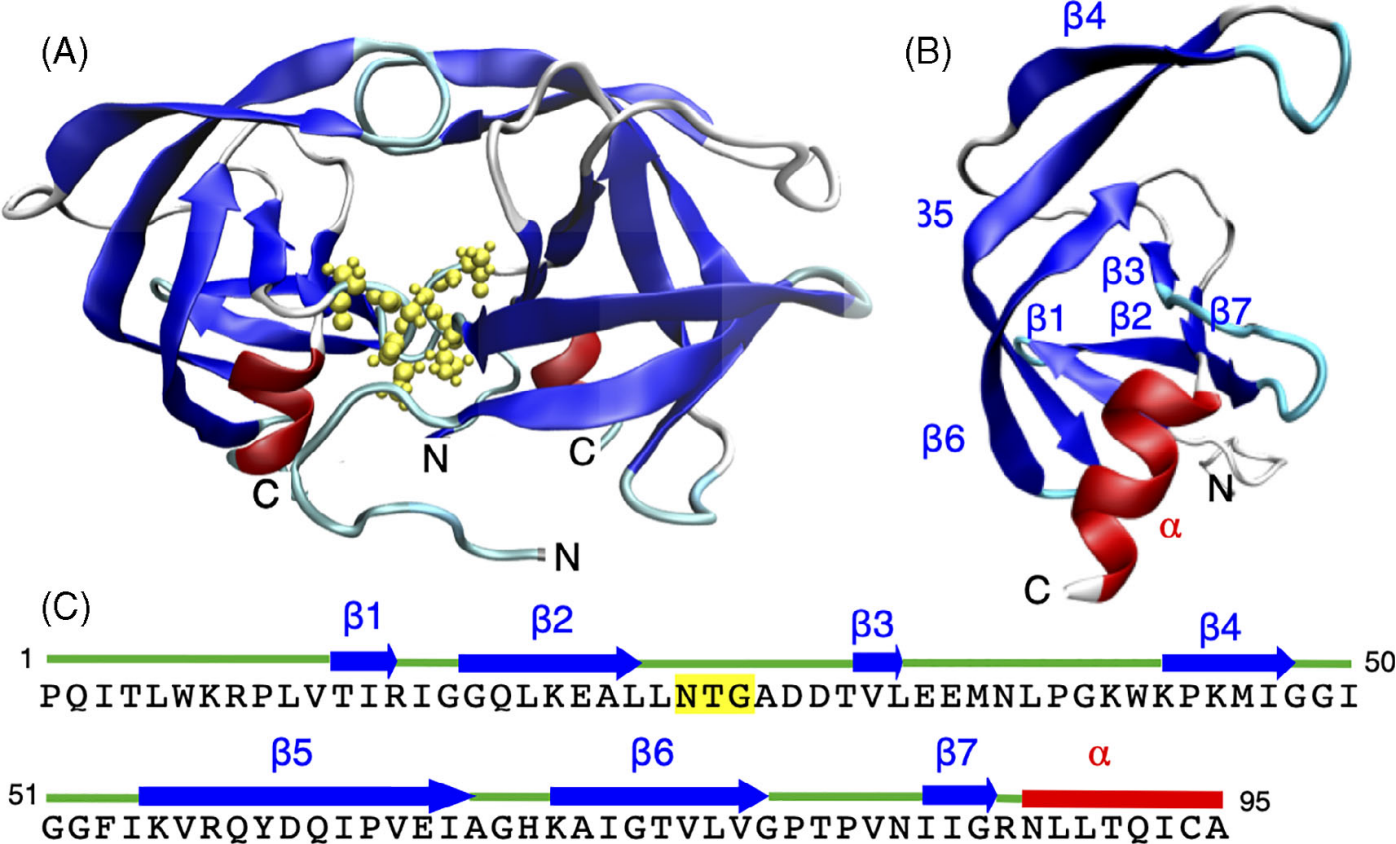
The native conformation of HIV‐1‐PR and mHIV‐1‐PR_1–95_. (A) Structure of the HIV‐1‐PR homodimer (PDB code 1BVG). The active site is highlighted by yellow spheres. (B) Structure of the 1–95 variant mHIV‐1‐PR_1–95_ (PDB code 1Q9P). (C) The sequence of mHIV‐1‐PR; the active site is highlighted in yellow [Color figure can be viewed at wileyonlinelibrary.com]

HIV‐1 protease is an aspartic acid protease, which in its active form exists as a homodimer^23^ (Figure 1A). Analysis of its folding kinetics identified a monomeric intermediate that associates to form the native dimer structure.^24^ Deletion of the last four C‐terminal residues stabilizes a monomeric, fully folded form.^25^ In fact, the native structure of this mHIV‐1‐PR_1–95_, which predominantly contains β‐sheet structure and a C‐terminal α‐helix,^18^ is highly similar to the structure in the dimer (cf. Figure 1B). Both the unfolding and refolding kinetics of mHIV‐1‐PR studied in urea by fluorescence display two time scales, suggesting the presence of at least one kinetic intermediate and the typical refolding time of mHIV‐1‐PR_1–95_ is of the order of a minute.^24^ Also, mechanical unfolding experiments suggest the presence of folding and unfolding intermediates.^26^ Interestingly, mHIV‐1‐PR was shown to display cold denaturation well above zero degrees Celsius,^27^ a feature that allowed us to compare the denatured states *D*_urea_, *D*_GdmCl_, and *D*_acid_ to a further state *D*_cold_.

The native and non‐native states of the wild‐type and of several variants of HIV‐1‐PR have also been characterized both in silico and *in vivo*.^28^, ^29^, ^30^ In spite of its central role as a target for anti‐retroviral therapies, biochemical and biophysical data on HIV‐1 protease are still limited. A tethered dimer in GdmCl,^31^, ^32^ a wild‐type dimer in acetic acid^33^ and HIV‐1‐protease embedded in its viral precursor protein in urea^34^ constitute some of these states. However, none of these studies were performed on the same variant of the protein, prohibiting a direct comparison of the results.

In the present work, we have performed titration experiments in different chemical denaturants using the exact same mHIV‐1‐PR_1–95_ variant and followed the changes by spectroscopy. This allowed us to monitor how the conformational properties of the denatured state depend on the kind and concentration of the denaturing agent, eventually extrapolating the properties of *D*_0_. The main quantity we investigated was the secondary chemical shifts, measured by heteronuclear NMR experiments. A nontrivial problem one has to face is then to interpret these data in terms of conformational properties of the protein. To assist us in this goal, we performed advanced molecular dynamics simulations of mHIV‐1‐PR_1–95_ in water, building an ensemble of conformations that describes the denatured state *D*_0_. The correctness of the simulated *D*_0_ was validated by back‐calculating the secondary chemical shifts from the simulation and comparing them with those obtained from the extrapolation to zero denaturant of the NMR results.

## 2 MATERIALS AND METHODS

### 2.1 Protein expression, purification, and sample preparation

A synthetic gene encoding the HIV‐1 protease monomer lacking the last 4 residues, mHIV‐1‐PR_1–95_ was a kind gift from Dr. Celia Schiffer, University of Massachusetts Medical School, and was cloned into a pET11a vector. The protein was expressed in *Escherichia coli Rosetta (DE3) cells upon induction with 0.2 mM IPTG*. For the synthesis of isotope labeled protein, Spectra9 LB media (Euriso‐top, France—cod. CGM‐3030‐CN‐1, 1 L) enriched with ^15^N and ^13^C was used. Cells obtained from 0.4 L of culture were lysed by sonication at 4°C in extraction buffer: 20 mM Tris/HCl, 1 mM ethylenediaminetetraacetic acid (EDTA), and 10 mM 1,4‐dithiothreitol (DTT), pH 8. The protein was refolded as described previously.^26^ For spectroscopic measurements, the protein was dialyzed against 20 mM sodium phosphate, pH 6.0.

### 2.2 Fluorescence and CD experiments

Fluorescence experiments were performed with a Varian Eclipse fluorimeter on 4 μM protein in 20 mM sodium phosphate at pH 6.0 and 25°C by adding different concentrations of denaturant. Circular dichroism (CD) measurements were conducted at 230 nm and a protein concentration of 15 μM in 20 mM sodium phosphate, pH 6, and containing different amounts of denaturant at 25°C using a JASCO J810 spectropolarimeter and a 1 mm path length. A total of 120 data points were recorded over 1 min and averaged. The actual urea and GdmCl concentrations were confirmed by refractive index measurements. For the temperature transition, CD measurements were conducted at 205 nm and a protein concentration of 10 μM in 20 mM sodium phosphate, pH 6. The temperature was increased in 1°C steps from 3°C to 20°C and in 2°C steps from 20°C to 90°C using a Peltier control unit. To account for the slow refolding kinetics, each point was allowed to equilibrate 5 min prior to detection.

### 2.3 NMR experiments

#### 2.3.1 Backbone assignment and *R*_1_ , *R*_2_ , and hetNOE relaxation experiments

All NMR spectra were recorded either on an Agilent DD2 800 MHz or a Varian INOVA 750 MHz spectrometer using a room temperature probe, and standard pulse programs from the Vnmrj BioPack. For assignment, we prepared 11 different aliquots of ^15^N‐^13^C‐labeled ~200 μM protein solution in 20 mM sodium phosphate, pH 6.0, and 10% D_2_O (v/v), 125 μM 2,2‐dimethyl‐2‐silanepentane‐5‐sulfonic acid (DSS) containing 4, 6, and 8 M urea, 0.75, 1, 2, and 4 M GdmCl, or 9% (v/v), 25% (v/v), and 45% (v/v) acetic acid, respectively, and one containing no extra additives. For relaxation experiments, identical samples were prepared containing a ^15^N‐labeled ~200 μM protein solution. The backbone nuclei were assigned using HSQC,^35^ HNCA, HNCO,^36^ HN(CA)CO,^37^ HNCOCA,^38^ HNCACB,^39^ CBCACONH,^40^ HNN,^41^ and ^15^N‐edited NOESY‐HSQC^42^ spectra recorded at 25°C for the samples containing 4 and 8 M urea, and using only HSQC, HNCA, HNCO, HNCOCA, HNN, and ^15^N‐edited NOESY‐HSQC spectra for 1 M GdmCl and 25% (v/v) acetic acid. For the remaining samples, only the HSQC, HNCA, HNCOCA, and HNCO spectra were used for backbone assignment. The assignment was completed for 95% of all nonproline residues for samples containing acetic acid, 96% for samples containing GdmCl, 97% for samples containing urea, and 97% for cold denatured protein.

To analyze the *T*_1_ and *T*_2_ relaxation times and heteronuclear nuclear Overhauser effects (hetNOEs), five series of spectra were recorded on ^15^N‐labeled protein in 20 mM sodium phosphate, pH 6.0, and 10% D_2_O (v/v), 125 μM DSS, also containing 4 or 8 M urea, 1 M GdmCl or 25% (v/v) acetic acid, at 25°C.^43^ We chose eight different delay times: 0, 100, 200, 300, 500, 700, 900, and 1200 ms for recording *T*_1_ and nine different delay times: 10, 50, 90, 130, 170, 190, 210, 230, and 250 ms for recording *T*_*2*_ relaxation times. For the hetNOE a relaxation delay of 8 s was used.

#### 2.3.2 Pulsed‐filed‐gradient NMR diffusion experiments

The above‐described protein samples were used to record sets of 60 bipolar pulse‐pair stimulated echo experiments using a watergate scheme for water suppression with varying gradient strength.^44^ As internal reference, 0.5% (v/v) dioxane was added to all samples to correct for viscosity effects by the solvent. All spectra were obtained at 25°C using 32 transients on a 750 MHz Varian INOVA spectrometer.

#### 2.3.3 2‐D and 3‐D NMR spectra processing

The X‐carrier frequency was determined by referencing to internal DSS. The DSS frequency was obtained from a 1D ^1^H spectra recorded immediately before the remaining experiments. Indirect referencing was used in the ^15^N and ^13^C dimensions by use of conversion factors.^45^ The spectra were processed using nmrPipe^46^ and qMDD.^47^ Spectrometer frequencies and carrier frequencies in ppm were inserted with four decimals. Zero‐filling to nearest power of 2 was used. The processed spectra were assigned and analyzed in CcpNmr Analysis.^48^ The assigned HSQC spectra were further used to extract the relaxation decays from the series of spectra recorded to determine the *T_1_* and *T_2_* relaxation times. Relaxation decay curves were fitted to single exponentials and relaxation times determined using the *relax* software^49^, ^50^ The values of *R*_1_, *R*_2_, and the hetNOE recorded at 17.6 Tesla were used to derive the spectral density function at three frequencies (0, *ω*_H_, and *ω*_N_) analyzed by reduced spectral density mapping using *relax*.^49^, ^50^

#### 2.3.4 DOSY processing

Each set of 60 1D‐^1^H spectra was separately processed and analyzed using The DOSY Toolbox^51^ and MATLAB.^52^ Spectra were phased in zero order and smoothed using a 10 Hz Lorentzian efficiently removing most visible noise. The function msbackadj was used rather than the internal DOSY Toolbox baseline correction routine.

#### 2.3.5 Analysis of the chemical shifts

Secondary chemical shifts associated with different atoms were systematized using the formula (Δ(*δC* ^α^) + Δ(*δC*') − 0.5*Δ(*δN*)).^7^

#### 2.3.6 Fit of dynamics parameters

The *R_2_* parameters were fitted with the function described in equation (3) in the supplementary materials of Reference 53. The fit was done with a nonlinear least‐square algorithm employing a Levenberg‐Marquardt algorithm. To avoid overfitting, we performed fits with different number of exponentials, eventually choosing the minimum number of exponentials which gave a chi^2^ lower than 5.

NMR data have been deposited at the BioMagResBank with the accession number: 25255.

### 2.4 Molecular dynamics simulations

The mHIV‐1‐PR_1–95_ system was described with the Amber 99SBdisp force field^54^ in Tip4/pd water and simulated with Gromacs 2020.4.^55^ The protein was prepared in a dodecahedric box of 571 nm^3^ with 19160 water molecules and 4 Cl^−^ ions to neutralize the charge. A preliminary simulation of 50 ns at 700 K and constant volume was carried out, followed by 100 ns at 300 K and 1 atm. From the latter simulation, 110 conformations were extracted to act as starting conformations of the production run. A replica‐exchange simulation was then performed with 110 replicas whose temperature range from 300 to 500 K for a total of 68 μs.

Once the first 30 ns were removed, the replica at 300 K was analyzed to validate the simulation against the NMR data. Secondary chemical shifts were calculated for each conformation with Sparta+^56^ and averaged over all of them. To calculate secondary chemical shifts, we used Bax's reference values.^56^

To predict the *R_1_* relaxation parameters qualitatively, we extracted 50 conformations from the 300 K trajectory, using each of them as starting point of a 1 ns simulation at fixed temperature. The root mean square fluctuations (RMSF) around each of the 50 average conformations were calculated and then averaged together. The experimental *R_2_* values were compared to the solvent‐accessible surface area (SASA) of each residue, averaged over the full 300 K trajectory.

The clustering of the 300 K trajectory was performed with a tailor‐made Python code that uses the fraction *q* of common contacts as underlying metric, normalized to the maximum between the numbers of contacts of the two structures. A contact is defined if the center of mass of two residues is closer than 0.65 nm. In the calculation of *q*, only pairs of residues which were further apart by at least three other residues along the chain were considered.

## 3 RESULTS

### 3.1 Denaturation of mHIV‐1

Following the far‐UV CD spectra of folded mHIV‐1‐PR_1–95_ and cold‐denatured mHIV‐1‐PR_1–95_ (Figure S1 in the SI), we observed nearly identical spectra over a wide range of wavelength spanning from 208 to 250 nm. This is due to the presence of dominating aromatic contributions in the far‐UV region,^57^ which result in an atypical CD spectrum of a β‐sheet protein. To monitor the unfolding temperature of mHIV‐1‐PR_1–95_, we therefore chose to record the mean residue ellipticity at 205 nm as a function of increasing temperature from 3 °C to 90 °C (Figure S2).

Besides cold denaturation occurring at 10°C, already described in Reference 27, we observed heat denaturation with an apparent midpoint temperature *T*_m_^app^ of approximately 50 °C and a third transition at ~80 °C, corresponding to the irreversible aggregation of the protein. Due to aggregation, the heat‐denatured state was not considered for high‐resolution NMR studies. Under all conditions explored, the native state was never fully populated and hence all equilibrium unfolding transitions could not be satisfactorily fitted to a standard equilibrium transition curve.

**FIGURE 2:**
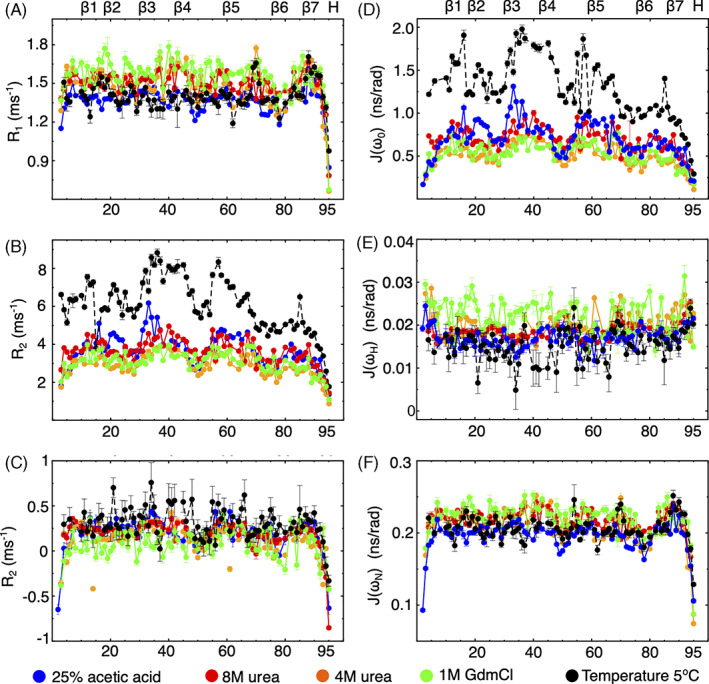
Equilibrium unfolding of HIV‐1‐PR_1–95_. Top: mean residue ellipticity at 230 nm of a 15 μM HIV1‐PR_1–95_ in 20 mM sodium phosphate, pH 6, measured in the presence of increasing concentrations of urea (left), GdmCl (middle) and acetic acid (right) at 25°C. Bottom: wavelength of maximum fluorescence emission of 4 μM mHIV‐1‐PR_1–95_ sample 20 mM sodium phosphate buffer, pH 6, measured in the presence of increasing concentrations of urea, GdmCl or acetic acid, at 25°C (excitation wavelength: 295 nm) [Color figure can be viewed at wileyonlinelibrary.com]

In the presence of increasing amounts of urea, mHIV‐1‐PR_1–95_ showed a very broad transition indicative of a noncooperative unfolding (Figure 2). Interestingly, close to 2 M urea, the unfolding transition was more than 95% complete as judged from CD measurements, but not according to fluorescence emission. Thus, the data did not seem to agree with the expected behavior of a two‐state unfolding mechanism. At protein concentrations as high as those used for the NMR experiments, mHIV‐1‐PR_1–95_ showed strong visible aggregation making reliable measurements below 4 M urea impossible. In all NMR experiments, the protein was >95% unfolded as judged from the CD signal. Monitoring the hydrodynamic radius *R*_*h*_ by pulsed‐filed‐gradient (PFG) NMR experiments at 4 M urea showed that the hydrodynamic radius, *R*_*h*_ = 27.2 ± 0.5 Å, was comparable to data in Reference 58. However, when increasing the urea concentration from 4 to 8 M urea, mHIV‐1‐PR_1–95_ underwent further expansion from 27.2 ± 0.5 to 28.0 ± 0.6 Å (Table 1).

**TABLE 1:** *R*_*h*_ of mHIV‐1‐PR_1–95_ measured in different denaturants by PFG‐NMR

Urea		GdmCl		Acetic acid	
c (urea)	*R*_h_ (Å)	c (GdmCl)	*R*_*h*_ (Å)	% acetic acid	*R*_h_ (Å)
4 M	27.2	0.75 M	24.0	9	27.2
6 M	27.7	1 M	25.8	25	29.8
8 M	28.0	2 M	26.4	45	—^a^

*Notes*: All measurements were performed in 90% H_2_O/10% D_2_O at 25°C. The experimental uncertainty was estimated to ±2% based on triplicate measurements.

^a^ The fitted data to this measurement were not available due to overlap with signals arising from acetic acid.

Compared to urea denaturation, the equilibrium transition curve was steeper and appeared more cooperative using GdmCl. The secondary structure of mHIV‐1‐PR_1–95_ had already fully disappeared in the presence of less than 0.5 M GdmCl as monitored by CD (Figure 2). Again, fluorescence emission indicated mHIV‐1‐PR_1–95_ to be >95% unfolded at a much higher concentration of denaturant than for CD, indicating that the monomer did not follow a two‐state unfolding mechanism. At a denaturant concentration below 0.75 M GdmCl, protein aggregation was observed and NMR experiments were only recorded when more than 95% of the protein was denatured. Similar to the case in urea, the *R*_*h*_ increased with increasing concentration of GdmCl. For three selected samples, the *R*_*h*_ increased from 24 ± 0.5 Å at 0.75 M GdmCl to 26.2 ± 0.5 Å at 2 M GdmCl (Table 1).

The acid denatured state appears crucial for successful refolding of the dimeric protein^59^ and changes in protonation states can result in small but distinct differences in the preferences for local structure. The addition of just 0.1% acetic acid to 20 mM sodium phosphate, pH 6, caused the pH of the sample to drop to 4, and in an identical buffer containing 0.75% acetic acid, pH was 3.4. Addition of 5% acetic acid or more decreased pH below 3, where dimeric HIV‐1‐PR is reported to be largely unfolded.^59^ We observed a midpoint of denaturation at about 0.5% acetic acid, which corresponded to a measured pH of 3.6. From CD experiments, further addition of acetic acid caused additional structural changes even when full acid denaturation was complete, when judged from fluorescence emission spectra (Figure 2). In addition, we observed an increase of *R*_*h*_ from 27.2 Å ± 0.5 at 9% acetic acid to 29.8 ± 0.6 Å at 25% acetic acid (Table 1). This increase was significantly larger than for the other two denaturants.

Interestingly, in the absence of denaturant, mHIV‐1‐PR_1–95_ is folded except for the N‐terminal region.^18^ In addition, the wild‐type protein folds through a monomeric phase before dimerization.^24^, ^30^, ^60^, ^61^ Inspection of the HSQC spectrum of mHIV‐1‐PR_1–95_ recorded in 20 mM sodium phosphate (pH 6.0) at 25°C revealed a small but nondisputable second population. Under these experimental conditions, the folding rate of the monomer^24^ is about 1 min^−1^, the equilibrium thus being in the slower regime of chemical exchange for NMR experiments. Hence, the second set of peaks most likely originated from the denatured state *D*_*0*_.

### 3.2 Chemical shift analysis

For each type of denaturant, the heteronuclear backbone resonances were assigned at three different denaturant concentrations (Figure S3 in the SI). Moreover, the cold denatured state described in Reference 27 was taken into account. The secondary chemical shifts of the protein in different denaturation conditions were rather similar to each other (Figure S4).

**FIGURE 3:**
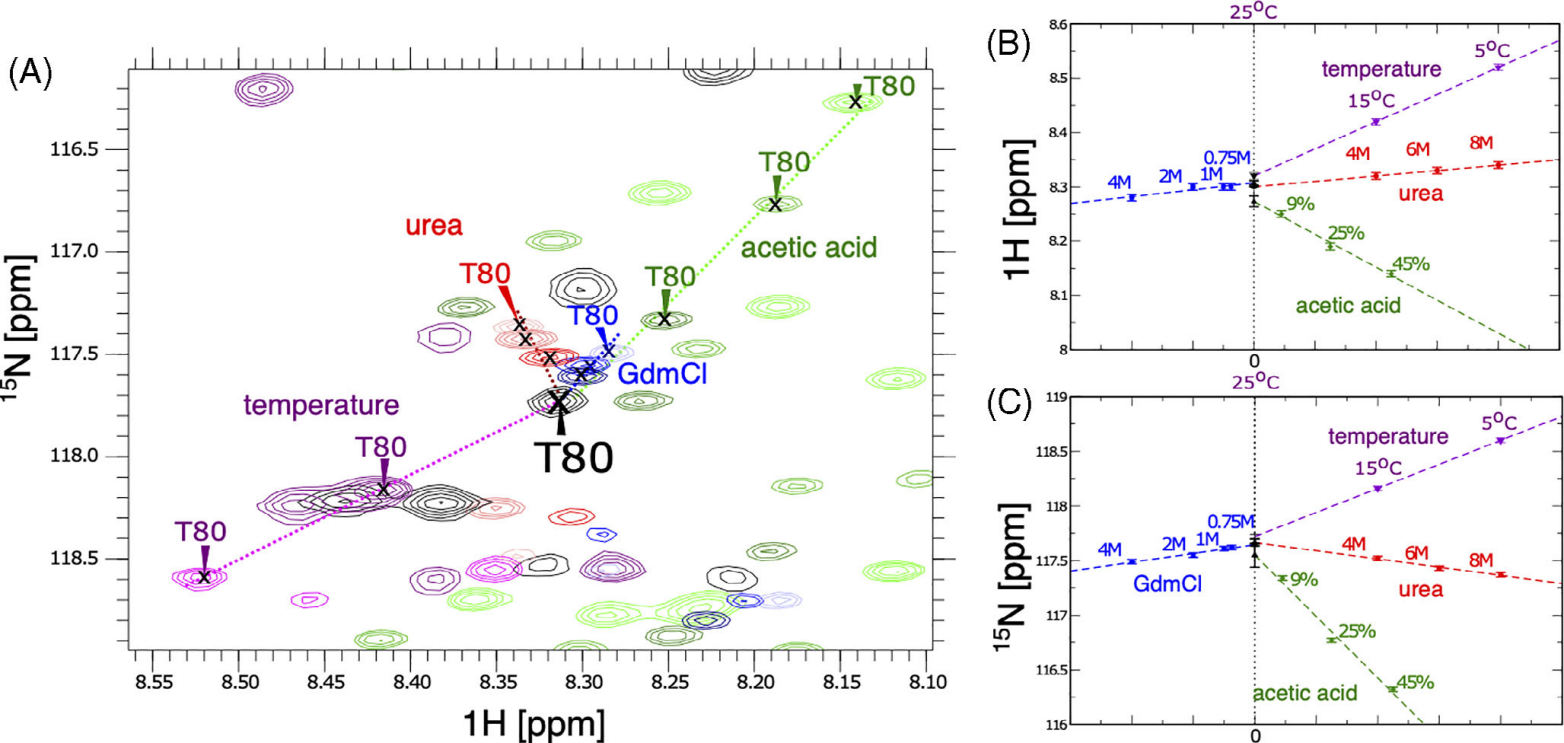
Convergence to the denatured state *D*_0_. (A) ^15^N‐HSQC spectra (zoom) of mHIV‐1‐PR_1–95_ showing T80 in 12 different ^15^N‐HSQC spectra recorded under various denaturing conditions. All peaks converge to the same position for T80 in *D*_0_. Red peaks: urea 8 M (light red), urea 6 M (medium red) and urea 4 M (dark red). Green peaks: acetic acid 45% (light green), acetic acid 25% (medium green) and acetic acid 9% (dark green). Blue peaks: GdmCl 4 M (light blue), GdmCl 2 M (medium blue), GdmCl 1 M (dark blue). Purple peaks: sodium phosphate 20 mM, pH 6) at 5°C (light violet), 15°C (dark violet). Black peaks: sodium phosphate 20 mM, pH 6 at 25°C (denatured state *D*_0_). (B) Schematic of T80 ^1^H chemical shifts of mHIV‐1‐PR as a function of denaturant concentrations; the *x*‐values are annotated in the graph (red: urea; blue: GdmCl; green: acetic acid; purple: different temperatures; black: physiological condition at 25°C [*D* ^phys^ state]). All ^1^H chemical shifts converge to the *D*_0_ state value. (C) Same for ^15^N chemical shifts [Color figure can be viewed at wileyonlinelibrary.com]

To describe the transient structures in the denatured state under nondenaturing conditions, *C* ^*α*^, *C*′, N, and H^N^ chemical shifts from individual titration series were extrapolated to the low intensity peaks observed at zero denaturant, as described in Figure 3. As a result, in 16 cases, the weak cross peaks observed at the position defined by the extrapolated values could be unambiguously assigned in the set of spectra recorded at physiological conditions at 25°C in the absence of any denaturant. For these 16 cases, the assignment by extrapolation was cross‐checked and confirmed by 3D backbone spectra. The same extrapolation procedure was applied to all residues and the remaining plots are shown in Figure S3 together with nine of the identified cross peaks in the HSQCs.

**FIGURE 4:**
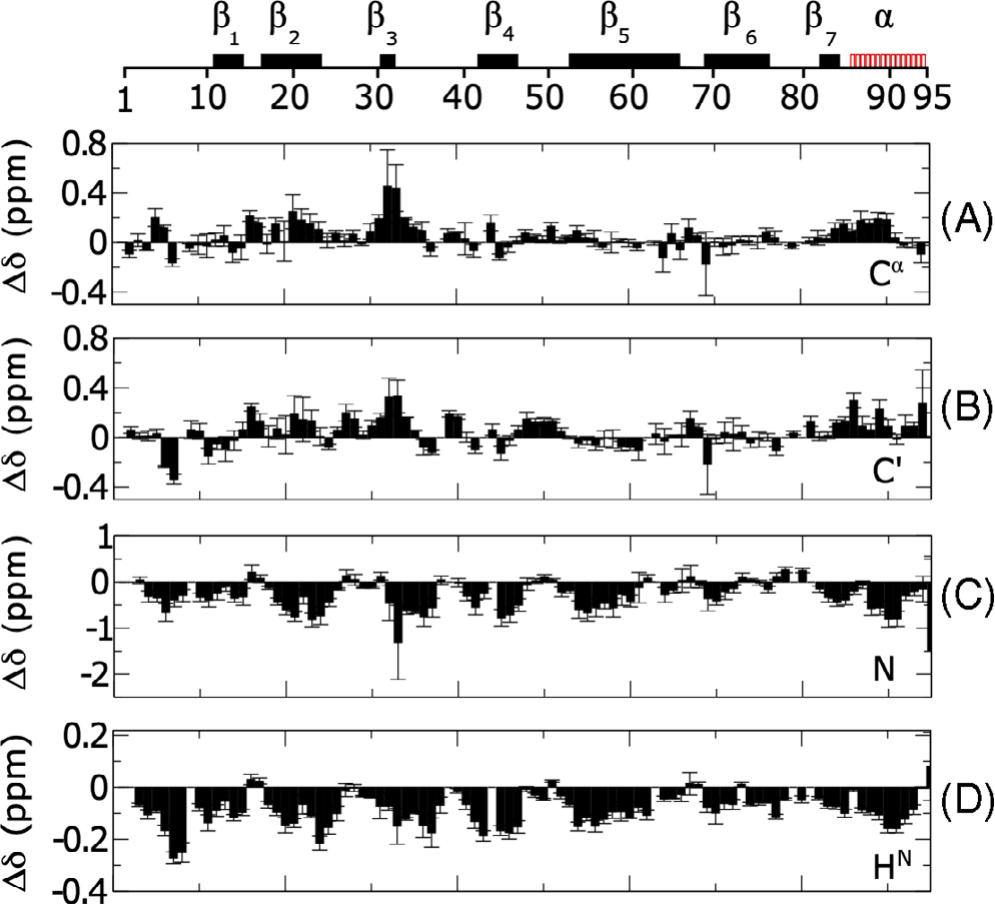
Secondary chemical shift analysis extrapolated for the *D*_0_ state of mHIV‐1‐PR_1–95_. For each residue, the chemical shifts under different experimental conditions were extrapolated to zero denaturant. Here we report the average of these extrapolations. The secondary chemical shifts were calculated by the use of the intrinsic random coil reference. The error bars indicate the total error of the procedure. Different nuclei were monitored (A) *C* ^α^, (B) *C* ^′^, (C) *N*, and (D) H^N^. The secondary structure of mHIV‐1‐PR_1–95_ is shown at the top [Color figure can be viewed at wileyonlinelibrary.com]

In Figure 4, we report the extrapolated secondary chemical shifts for the *C* ^*α*^, *C*′, N, and H^N^ backbone atoms averaged over the chemical shifts obtained from the four different extrapolations under different denaturing conditions. In these plots, we make use of intrinsic reference (i.e., the chemical shifts at highest denaturant concentration), although other choices gave similar results (see Figure S5 in the SI). The error bars indicate the associated SE and quantify the precision of the assignment under native conditions. Most residues displayed small errors compared to the average. A few discrepancies were observed for charged residues. The largest deviations were associated with the titration with acetic acid and were observed for three aspartic acids, D29, D30, and D60, and the single histidine, H69. Weaker effects were seen for four glutamates, E21, E34, E35, and E65. All the fits are displayed in Figure S6 in the SI.

### 3.3 Polypeptide chain dynamics

We next measured ^15^N spin‐lattice/spin‐spin relaxation rates as well as heteronuclear NOEs for the mHIV‐1‐PR_1–95_ at 25°C, at a field strength of 17.6 T (750 Mhz). These relaxation parameters are sensitive towards motions on the subnanosecond timescale. In addition, the *R*_2_ relaxation rate provides insights into motions on the millisecond to microsecond timescale. Full sets of relaxation data could be extracted for a total of 79 (5°C), 82 (4 M urea), 85 (8 M urea), 87 (1 M GdmCl), and 88 (25% acetic acid) residues, respectively.

**FIGURE 5:**
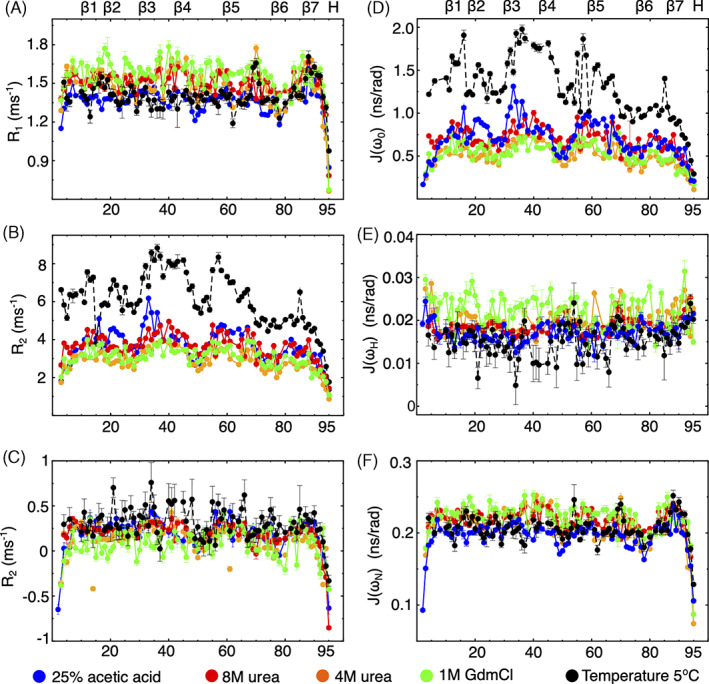
Chain dynamics in HIV‐1‐PR_1–95_ at different denaturant conditions. NMR relaxation rates (A–C) and spectral densities (D–F) in 25% acetic acid (blue dots), in 8 M urea (red dots), in 4 M urea (orange dots), in 0.9 M GdmCl (green dots), and in buffer at 5°C (black dots), respectively. Secondary structures of folded HIV‐1‐PR _1–95_ are shown in the top [Color figure can be viewed at wileyonlinelibrary.com]

For all five denatured states, the *R*_1_ values remained more or less constant throughout the sequence, with average values of 1.44 ± 0.04 (5°C), 1.48 ± 0.01 (4 M urea), 1.50 ± 0.01 (8 M urea), 1.56 ± 0.03 (1 M GdmCl), and 1.36 ± 0.02 ms^−1^ (25% acetic acid), respectively (Figure 5). The N‐ and the C‐termini showed lower *R*_1_ compared to the rest of the protein, consistent with faster timescale movements usually experienced for chain termini. In all five profiles, we observed a stretch (V77–V82) of significantly lower values followed by a stretch (I84–L89) of significantly increased values. The average *R*_1_ rates for the acetic acid and the cold denatured states were clearly reduced compared to those associated with the other two denatured states.

Measurements of the heteronuclear steady‐state NOEs showed mostly positive values apart from those associated with the N‐ and C‐terminal regions. The profile of the heteronuclear NOE did not agree with a fully unfolded state, but rather followed a profile of four arcs for all four denatured states.

The *R*_2_ value is usually the most informative parameter for denatured proteins as it can reveal regions that undergo chemical exchange. For a fully extended protein where chain dynamics is dominated by unrestrained segmental motion, this profile usually adopts the shape of an inverted U, with a plateau along the chain and steep drops at the N‐ and C‐terminal ends.^62^ For all five denatured states, the *R*_2_ profiles deviated from an inverted U‐shape. Instead, they displayed a four‐arcs‐like pattern distributed almost evenly over the sequence, and covering R8‐L24, V32‐G48, V56‐G68, and G78‐A95 (cf. Figure S7 in the SI), respectively. This unusual pattern of *R*_2_ rates persisted at 8 M urea where the unfolded mHIV‐1‐PR_1–95_ showed a more elongated conformation, as testified by the corresponding *R*_h_ value (Table 1).

The probability function of finding motions at a given angular frequency *ω* can be described by the spectral density function *J*(*ω*). As unfolded states cannot be described in terms of an overall rotational correlation time, we instead chose to describe the relaxation data by reduced spectral density mapping.^63^ We used the values of *R*_1_, *R*_2_, and the heteronuclear NOE recorded at 17.6 T to derive the spectral density function at three frequencies (0, *ω*_H_, and *ω*_N_, cf. Figure 5). Neither *J*(*ω*_H_) nor *J*(*ω*_N_) showed large variation in their profiles when plotted against the sequence, in agreement with the related profiles for the hetNOE and the *R*_1_ values, respectively. Instead, the *J*(0) values displayed the same pattern of four arcs as described for *R*_2_. Of importance, we note that the arches mostly revolve around prolines.

### 3.4 Experimental validation of molecular dynamics simulation of *D*_0_

A replica‐exchange simulation of 68 μs of mHIV‐1‐PR_1–95_ in water is performed with 110 temperatures in the range from 300 to 500 K, as described in the Materials and Methods. The degree of equilibration of the simulation seems acceptable, as testified by the good exchange between replicas (cf. Figure S8) and by the convergence of the average contact map (cf. Figure S9).

**FIGURE 6:**
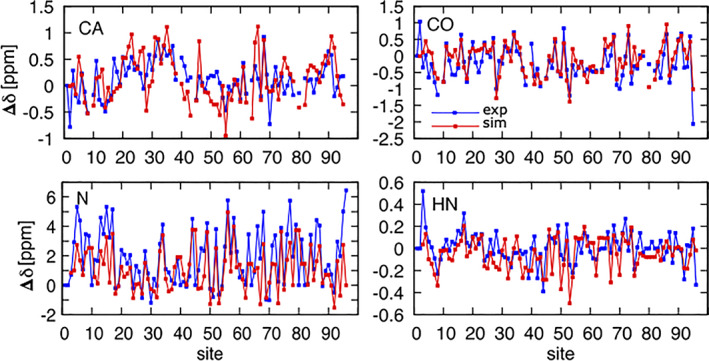
Secondary chemical shift comparison between experiment and simulation. A comparison between the average secondary chemical shifts of *D*_0_ predicted by the MD simulation (red line) and those extrapolated from the experiment to zero denaturant (blue line) [Color figure can be viewed at wileyonlinelibrary.com]

To validate the simulation, we calculated the average secondary chemical shifts from the simulated trajectory using Sparta+^56^ and compared them with the experimental values extrapolated for *D*_0_ (Figure 6). The Pearson's correlation coefficients are *r* = 0.68 for CA, *r* = 0.63 for *C*′, *r* = 0.67 for N, and *r* = 0.54 for HN (also cf. Figure S10). Thus, the simulated data are in good agreement with the experimental values (*p* < 10^−5^, as calculated from a random bootstrap of the data).

The average radius of gyration, *R*_g_, calculated from the simulated conformations, was 2.19 ± 0.48 nm. The corresponding *R*_h_ can be estimated^64^ to be 2.45 ± 0.61 nm. This is equal, within the error bars, to the hydrodynamic radius 2.51 ± 0.19 nm obtained as extrapolation to zero denaturant from the data of Table 1. Another, more qualitative comparison was done between the experimental and simulated relaxation parameters *R*_1_ and *R*_2_. The reason why a direct comparison cannot be done is that a replica‐exchange simulation is efficient in sampling the equilibrium conformations of the protein at the price of generating an unphysical time‐dependent trajectory that would be necessary for calculating the NMR relaxation parameters.

**FIGURE 7:**
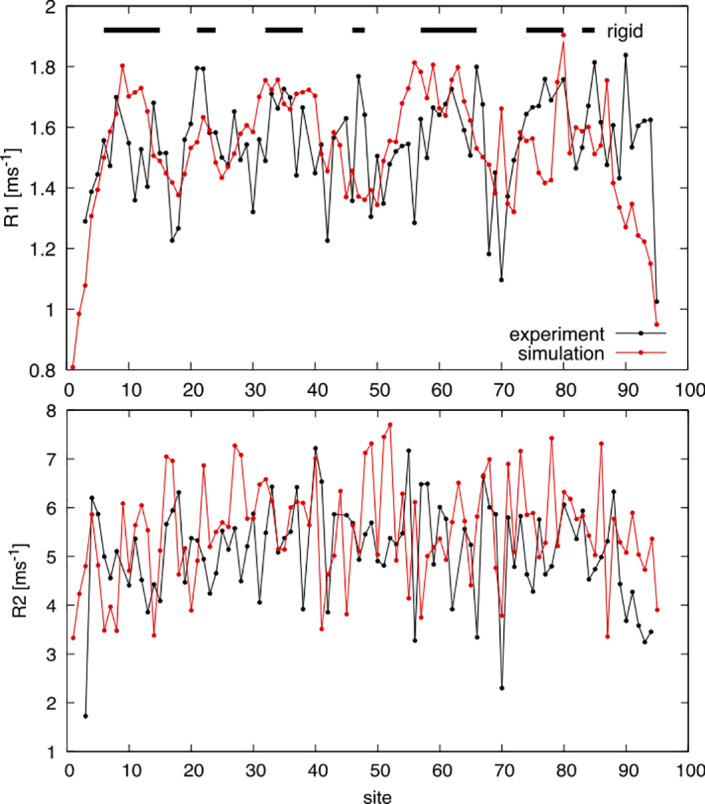
The experimental *R*_1_ and *R*_2_ relaxation parameters compared with proxies of the same quantities calculated from the MD simulations. In the case of *R*_1_, we plotted (in red) the function (2.7‐2*RMSF/nm)/ms, where the RMSF is calculated from 1 ns MD simulations starting from the sampled conformations. In the case of *R*_2_, we used as proxy the function (8.7–3*SASA/nm^2^)/ms [Color figure can be viewed at wileyonlinelibrary.com]

To give an approximate estimate of *R*_1_ from the simulation, we performed 20 plain‐MD simulations at fixed temperature (300 K) starting from 20 conformations extracted from the replica‐exchange trajectory. Each simulation lasted for 1 ns, that is the time scale described by the *R*_1_ parameter. From each simulation, we calculated the RMSF around the average conformation. We expected that *R*_1_ is anticorrelated with the RMSF. The comparison between the experimental *R*_1_ and the (rescaled and shifted) RMSF is displayed in Figure 7. Although the linear correlation is not high (*r* = 0.21), 74% of points stay on the same side with respect to the median (*p* = 10^−8^), suggesting the two curves indicate similar regions of rigid and flexible residues (black bars above the curves).

The values of *R*_2_ that reflect the conformational freedom of residues on the μs–ms timescale, were compared with the total SASA of each amino acid, calculated on the replica‐exchange simulation. Again, the linear correlation is low (*r* = 0.16) but 68% of the points stay on the same side with respect to the median (*p* = 10^−4^), indicating that residues that are experimentally more flexible are those less constrained by other parts of the polymer in the simulation.

### 3.5 Analysis of the ensemble of conformations of *D*_0_

The ensemble of conformations generated by the replica‐exchange algorithm at 300 K was further analyzed to characterize *D*_0_. The average *R*_g_ of value 2.19 ± 0.48 nm (cf. Figure S11) was consistently larger than the value 1.28 nm of the native conformation. The fact that the contact probability between pairs of residues as a function of their distance along the chain is a power law with an exponent ≈−1.8 (cf. Figure S12) suggests that the chain is, on average, in a coil state.

The average number of contacts is 60.6 ± 15.3 (cf. Figure S13) and the fraction q_N_ of native contacts is low (≈0.018 ± 0.011). To be noted the fraction *q*_N_ of native contacts in the denatured state is poorly correlated with the commonly employed root mean square deviation (RMSD) (cf. Figure S14). In fact, the RMSD is a highly nonlinear function of the diversity between conformations in that it is very sensitive to conformational changes between similar conformations and quite insensitive to large conformational changes between dissimilar conformations. Since the denatured state is expected to be conformationally very heterogeneous, we compared pairs of conformations using the fraction *q* of common contacts (and we compared a conformation to the native one using the fraction *q*_*N*_ of native contacts).

**FIGURE 8:**
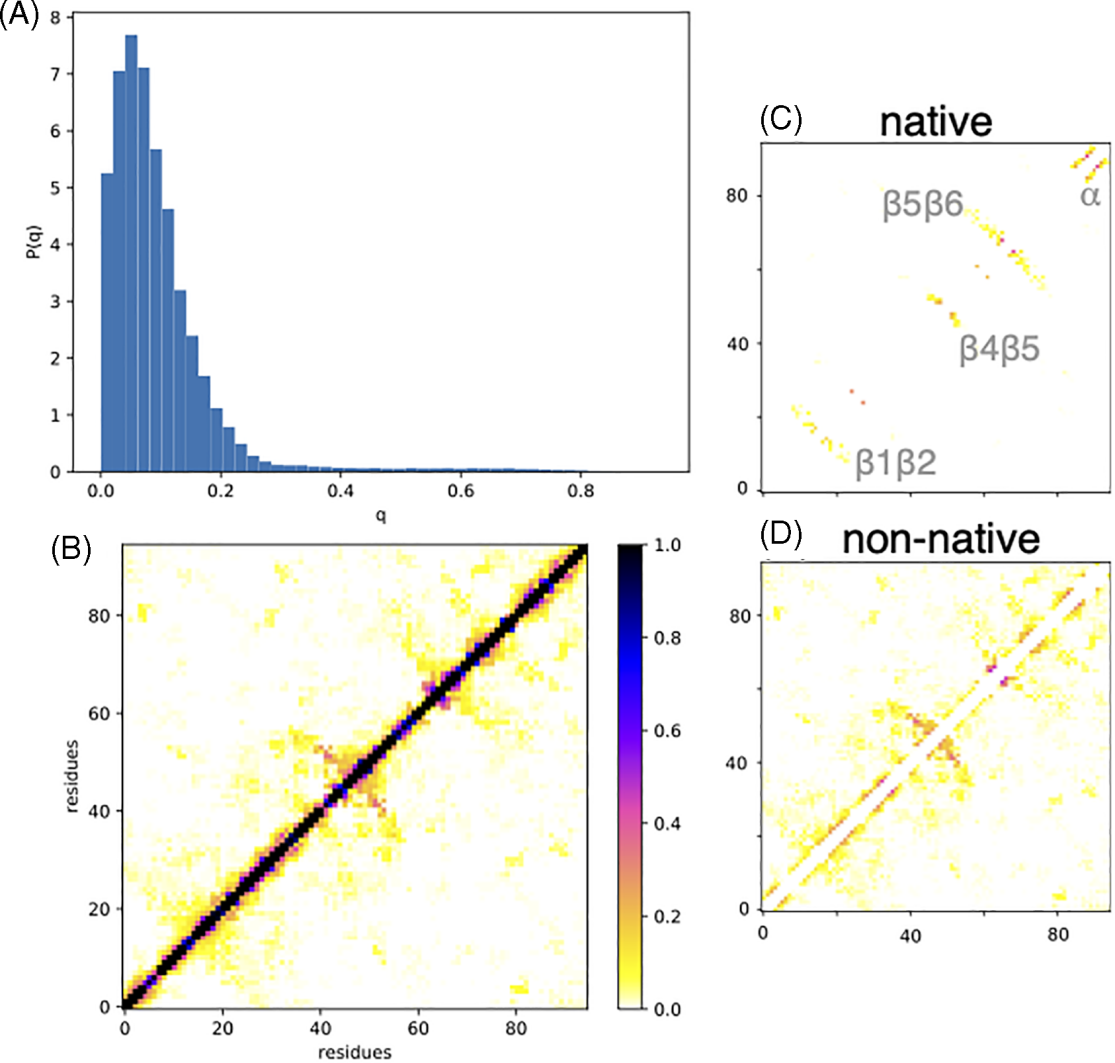
Equilibrium properties of the simulated protein (A) the distribution of similarity *q* between the conformations of *D*_0_, (B) the average contact map of mHIV‐1‐PR_1–95_, (C) the average contact map limited to native contacts, (D) the average contact map limited to non‐native contacts [Color figure can be viewed at wileyonlinelibrary.com]

In Figure 8A, the distribution of common contacts *q* between denatured conformations is plotted. Its average is 0.09 ± 0.08 but it displays a tail up to 0.7. Not surprisingly, the denatured state *D*_0_ thus appears conformationally very heterogeneous. However, its average contact map (cf. Figure 8B) displays well‐defined secondary structures that can reach a probability of 0.4 and also tertiary structures populated with probabilities up to 0.15. Some of these structures are native‐like and include the hairpin β1–β2, the hairpin β4–β5, the hairpin β5–β6, and the terminal α‐helix (cf. Figure 8C). Non‐native contacts (cf. Figure 8D) include a set of alternative structures in the region of the hairpin β4–β5, some fluctuating structure around P63 and a small amount of tertiary contacts.

**FIGURE 9:**
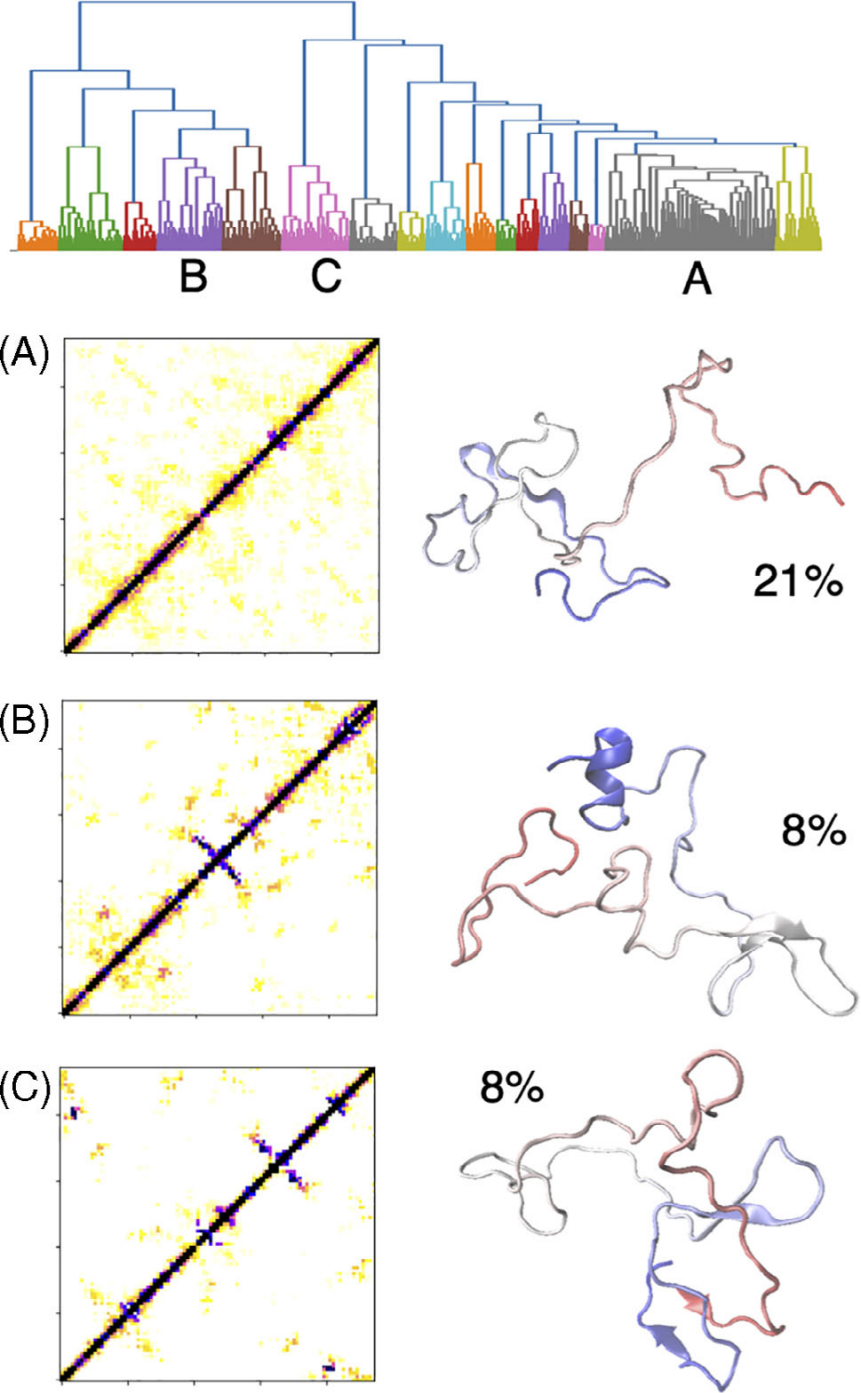
Cluster analysis of the conformations sampled at 300 K. In the dendrogram of structural similarity some clusters are indicated with Latin letters. For the three most populated clusters (labeled A, B, and C) the average contact map (normalized to the number of conformations of each cluster, same color code as Figure 8) and the central conformation (the N terminus in red, the C terminus in blue) are shown. The percental equilibrium population is also indicated for each cluster [Color figure can be viewed at wileyonlinelibrary.com]

A cluster analysis was performed for the simulated conformations of *D*_0_ at 300 K with the Ward algorithm. The fraction *q* of common contacts was used as underlying metrics for the clustering instead of the more common RMSD because of the reasons described above. We could identify 17 clusters. In Figure 9, we displayed the three most populated clusters (others can be found in Figure S15). The most populated cluster (labeled A) has a population of 21% and is poorly structured; it contains most conformations with a low number of contacts. The only stable structure is a turn involving P63. Clusters B and C have a population of 8% each. Cluster B displays a non‐native β‐hairpin involving residues 39–45 and the native, C‐terminal α‐helix. Cluster C displays a β‐hairpin involving residues 56–63, a β‐turn 80–83, and tertiary contacts between this and the N‐terminal region 4–6.

In the other clusters (cf. Figure S15), particular recurrent non‐native contacts in the region 40–50 and the native α‐helix are seen.

## 4 DISCUSSION

The denatured state *D*_0_ of a protein under native solvent conditions is important to determine its behavior in the cell, but it is usually hard to characterize because of its intrinsic instability and low population. By studying the dependence of NMR observables as secondary chemical shifts and relaxation parameters under different denaturing conditions and extrapolating their values to native conditions, we could provide a conformational characterization of *D*_0_ of the HIV‐1‐PR_1–95_.

A remarkable result was that the extrapolations of these quantities to native conditions were rather independent on the denaturant and matched the minor population of *D*_0_ present at native conditions.

In 1976, Pfeil and Privalov showed in a series of experiments^9^ that the unfolding enthalpy of lysozyme, denatured by pH, GdmCl, and temperature was identical, once the mean energy associated with the denaturant (e.g., the ionization energy in the case of pH) was subtracted. From this, they concluded that the states denatured by different means are thermodynamically indistinguishable. Ever since it has been discussed whether the denatured states generated by different means of denaturation were structurally different or not. In the present structural study, the extrapolation of chemical shifts to nondenaturing conditions plays a similar role to that of subtracting the denaturing energy in Privalov's experiment and all the extrapolations seem to agree very well with the presence of a single denatured state.

The interpretation of the raw data produced by NMR experiments in terms of conformational properties of *D*_0_ is particularly difficult for a state composed of a plethora of heterogeneous conformations. In this case, MD simulations can be a valuable complement to the experimental data because of their ability to probe the system at atomic scale. A critical issue in this respect is whether MD simulations can provide a realistic picture of the state of interest of the protein. To address this concern, we compared the secondary chemical shifts, the hydrodynamic radius and the relaxation parameters predicted by the simulation with the experimental values. The good agreement we found is a consequence of two factors. First, we used advanced sampling techniques of simulation that favor the diffusion in the conformational space of the system, allowing it to sample a heterogeneous conformational space. Second, we employed a force field^54^ that was particularly adjusted to simulate intrinsically‐disordered proteins,^54^ namely systems with conformational properties that are analogous to those of the denatured state of a structured protein. It is important to stress that the tools to analyze a simulation of the denatured state are different than those typically used for native‐like states. For example, while the commonly used RMSD is a poor quantifier of the similarity between pairs of conformations with subtle common features, the fraction *q* of common contacts is a more sensitive tool.

The detection of transient native and non‐native structures in the denatured state of proteins is important to understand their fast folding^65^, ^66^ and their aggregation.^4^, ^67^ In the case of a viral protein as HIV‐1‐PR, such structures can also be relevant as targets of antiviral molecules.^19^, ^20^, ^21^ We found in *D*_0_ specific secondary structures, both native and non‐native, displaying an equilibrium probability of up to ≈30% and also specific tertiary structures with equilibrium probability of up to ≈10%. Among them, the most stable elements seem to be the native C‐terminal α‐helix and a non‐native β‐like structure at the center of the protein. In particular, we observed transient populations of the hairpin β1–β2, the hairpin β4–β5, the hairpin β5–β6, and the terminal α‐helix. Except for the C‐terminal helix, the remaining structures correlate with the arches described by elevated *R*_2_ values (Figure 4) and further suggest that the simulations are capturing the details of the ensemble. Furthermore, several substates of *D*_0_ were identified by cluster analysis, each with peculiar conformational features, both native and non‐native. Interestingly, we observed positive secondary chemical shift values for *C* ^*α*^ and *C* ^′^ at few places along the chain (Figure 4), suggesting the presence of transient helicity in regions that in the native structure form β‐strands. Thus, non‐native interactions appear relevant to the unfolded state of HIV‐1‐PR and may play roles in guidance through the folding process.

## 5 CONCLUSIONS

The combination of experimental NMR techniques and of advanced MD simulation allowed us to characterize the denatured state of a complex protein as the HIV‐1 protease under native conditions. This state displays transient native and non‐native secondary and tertiary structures which could be of key relevance for guidance through the complex folding process. The strategy we used for HIV‐1 protease can be easily applied to other proteins of comparable length.

## CONFLICT OF INTEREST

The authors declare no conflict of interest.

### PEER REVIEW

The peer review history for this article is available at https://publons.com/publon/10.1002/prot.26189.

## Supporting information


**Figure S1** A) CD monitored temperature transition of mHIV‐1‐PRΔ95‐99. The mean residue ellipticity at 230 nm of a 10 μM mHIV1‐PRΔ95‐99 in 20 mM sodium phosphate, pH 6, recorded as a function of temperature. B) Far‐UV CD spectra of a 10 μM mHIV1‐ PRΔ95‐99 in 20 mM sodium phosphate, pH 6 recorded at different temperatures: Red: 5°C, black: 24°C, magenta 35°C, marron 45°C, violet 55°C, dark violet 65°C, blue 75°C, cyan 85°C (data taken from ref. 27).
**Figure S2**: CD monitored temperature transitions of mHIV‐1‐PRΔ95‐99. The mean residue ellipticity at 205 nm of a 10 μM HIV1‐ PRΔ95‐99 in 20 mM sodium phosphate, pH 6, was recorded as a function of temperature (data taken from ref. 27).
**Figure S3**: Zoom of the different 15 N‐HSQC spectra of HIV‐1‐PRΔ95‐99. A) 0 M, 4 M, 6 M, 8 M urea, B) 0.75 M, 1 M, 2 M, 4 M GdmCl, C) 0%, 9%, 25%, 45% acetic acid, D) 25, 15 and 5°. Peaks for 17G, 40G, 55 K, 67A, 71A and 76 L are folded in the 15 N‐dimension.
**Figure S4**: Secondary chemical shifts analysis calculated by combining Cα, C′ and 15 N chemical shifts assigned for HIV‐1‐PRΔ95‐99 as described by Reed et al.
**Figure S5**: Secondary chemical shifts obtained with three different reference values for the denatured state of HIV‐1‐PRΔ95‐99 in urea.
**Figure S6**. In the following pages, the chemical shift and the linear fit applied to all assigned amino acids.
**Figure S7**: Transverse relaxation rates under different conditions (black dots) and least‐square multiexponential fit (red curve). To avoid overfitting, we performed fits with different number of exponentials, eventually choosing the minimum number of exponentials which gave a chi2 lower than 5. We could fit 6 (5°C), 5 (4 M urea), 5 (8 M urea), 4 (1 M GdnHCl) and 4 (25% acetic acid) clusters for the R2 relaxation rates. Cluster 1 was split up, for reasons of comparison, into clusters (1a and 1b) comprising residues P1‐ R8 and P9‐L24. Clusters 2 and 5 were centered round one (P38) and two prolines (P79, P81), respectively. Clusters 3 and 4 were split by a single proline (P63).
**Figure S8**: the variation of temperature of the different replicas during the MD simulation.
**Figure S9**: The simulated contact probability between residues of mHIV‐1, calculated using only the first half of the simulation (upper‐left panel), using only the second half (upper‐right panel), and the difference between the two (lower panel).
**Figure S10**: Scatter plots of the simulated secondary chemical shifts vs the experimental values extrapolated at zero denaturant.
**Figure S11**: Distribution of the radius of gyration of D0 obtained from the simulation. Figure S12: Contact probability in log‐log scale between residues as a function of their distance along the chain. The dashed line indicates a power law with power − 1.8.
**Figure S13**: The distribution of number of contacts in the conformations sampled by the simulation at 300 K.
**Figure S14**: The RMSD of the sampled conformations with respect to the native conformation plotted vs the fraction qN of native contacts.
**Figure S15**: Some minority clusters. The central conformation is shown together with the mean contact map and the relative population of the cluster.

## Data Availability

The NMR data are deposited to the BMRB database with access number 25255.

## Endnote

This is exactly the same protein used in Reference 27, in spite of the unfortunate notation used in that reference.
