# Supplementary material for: The denatured state of HIV‐1 protease under native conditions
Source: Proteins. 2021 Aug 3;90(1):96–109. doi: 10.1002/prot.26189 (PMC9290662; doi:10.1002/prot.26189)
Supplement: Supplementary file 1 — Figure S1 A) CD monitored temperature transition of mHIV‐1‐PRΔ95‐99. The mean residue ellipticity at 230 nm of a 10 μM mHIV1‐PRΔ95‐99 in 20 mM sodium phosphate, pH 6, recorded as a function of temperature. B) Far‐UV CD spectra of a 10 μM mHIV1‐ PRΔ95‐99 in 20 mM sodium phosphate, pH 6 recorded at different temperatures: Red: 5°C, black: 24°C, magenta 35°C, marron 45°C, violet 55°C, dark violet 65°C, blue 75°C, cyan 85°C (data taken from ref. 27). Figure S2: CD monitored temperature transitions of mHIV‐1‐PRΔ95‐99. The mean residue ellipticity at 205 nm of a 10 μM HIV1‐ PRΔ95‐99 in 20 mM sodium phosphate, pH 6, was recorded as a function of temperature (data taken from ref. 27). Figure S3: Zoom of the different 15 N‐HSQC spectra of HIV‐1‐PRΔ95‐99. A) 0 M, 4 M, 6 M, 8 M urea, B) 0.75 M, 1 M, 2 M, 4 M GdmCl, C) 0%, 9%, 25%, 45% acetic acid, D) 25, 15 and 5°. Peaks for 17G, 40G, 55 K, 67A, 71A and 76 L are folded in the 15 N‐dimension. Figure S4: Secondary chemical shifts analysis calculated by combining Cα, C′ and 15 N chemical shifts assigned for HIV‐1‐PRΔ95‐99 as described by Reed et al. Figure S5: Secondary chemical shifts obtained with three different reference values for the denatured state of HIV‐1‐PRΔ95‐99 in urea. Figure S6. In the following pages, the chemical shift and the linear fit applied to all assigned amino acids. Figure S7: Transverse relaxation rates under different conditions (black dots) and least‐square multiexponential fit (red curve). To avoid overfitting, we performed fits with different number of exponentials, eventually choosing the minimum number of exponentials which gave a chi2 lower than 5. We could fit 6 (5°C), 5 (4 M urea), 5 (8 M urea), 4 (1 M GdnHCl) and 4 (25% acetic acid) clusters for the R2 relaxation rates. Cluster 1 was split up, for reasons of comparison, into clusters (1a and 1b) comprising residues P1‐ R8 and P9‐L24. Clusters 2 and 5 were centered round one (P38) and two prolines (P79, P81), respectively. Clusters 3 and 4 were s [file PROT-90-96-s001.pdf]

Supporting information

The denatured state of HIV-1 protease under native conditions

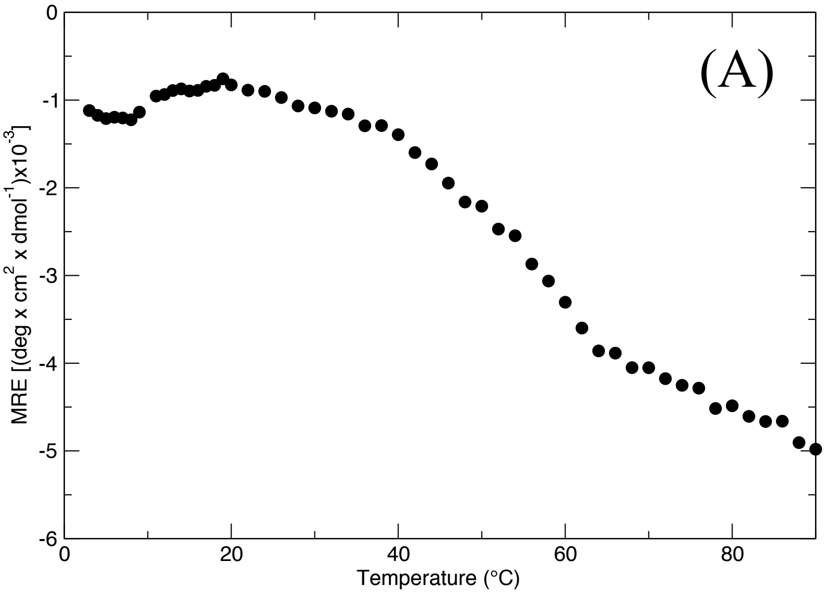

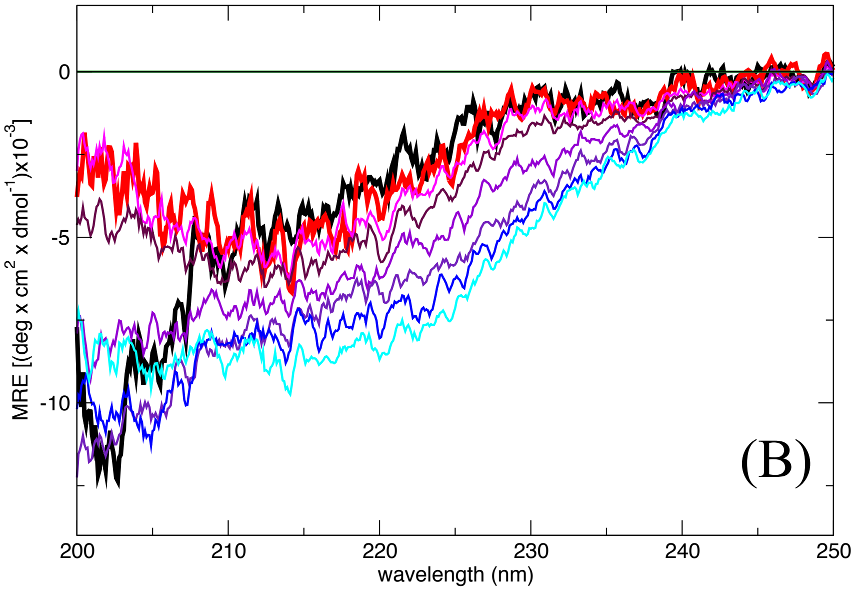

Figure S1: A) CD monitored temperature transition of mHIV-1-PR$_{\Delta 95-99}$. The mean residue ellipticity at 230 nm of a 10 µM mHIV1-PR$_{\Delta 95-99}$ in 20 mM sodium phosphate, pH 6, recorded as a function of temperature. B) Far-UV CD spectra of a 10 µM mHIV1- PR$_{\Delta 95-99}$ in 20 mM sodium phosphate, pH 6 recorded at different temperatures: Red: 5 ºC, black: 24 ºC, magenta 35 ºC, marron 45 ºC, violet 55 ºC, dark violet 65 ºC, blue 75 ºC, cyan 85 ºC (data taken from ref. 27).

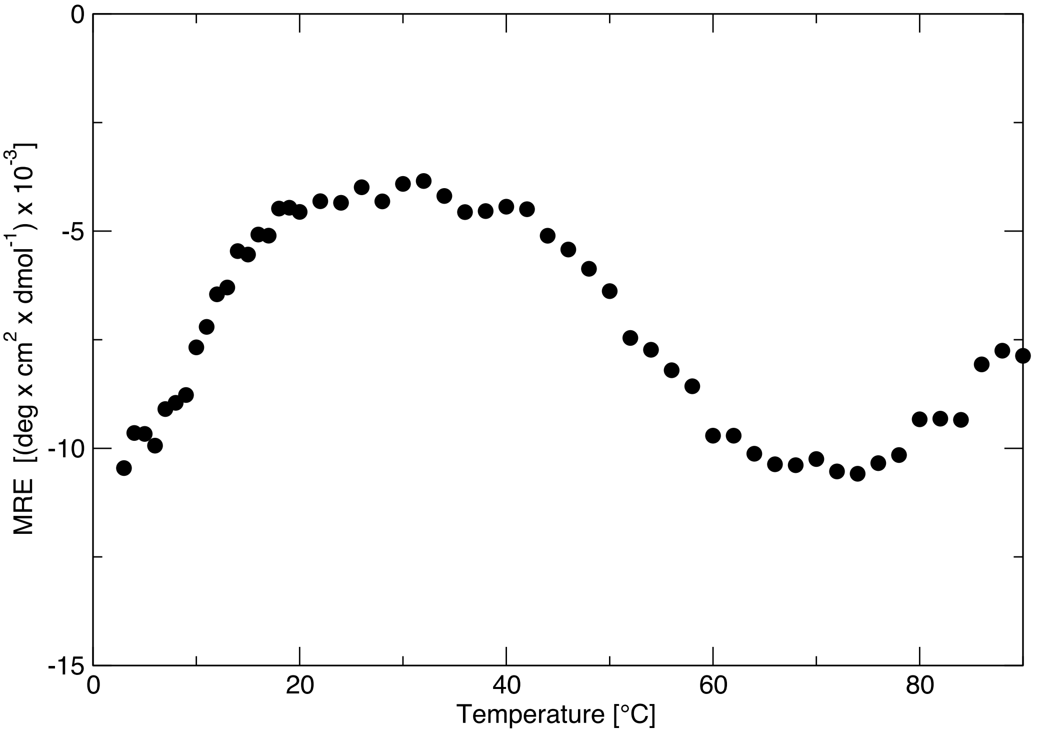

Figure S2: CD monitored temperature transitions of mHIV-1-PR$_{\Delta 95\text{-}99.}$ The mean residue ellipticity at 205 nm of a 10 μM HIV1- PR$_{\Delta 95\text{-}99}$ in 20 mM sodium phosphate, pH 6, was recorded as a function of temperature (data taken from ref. 27).

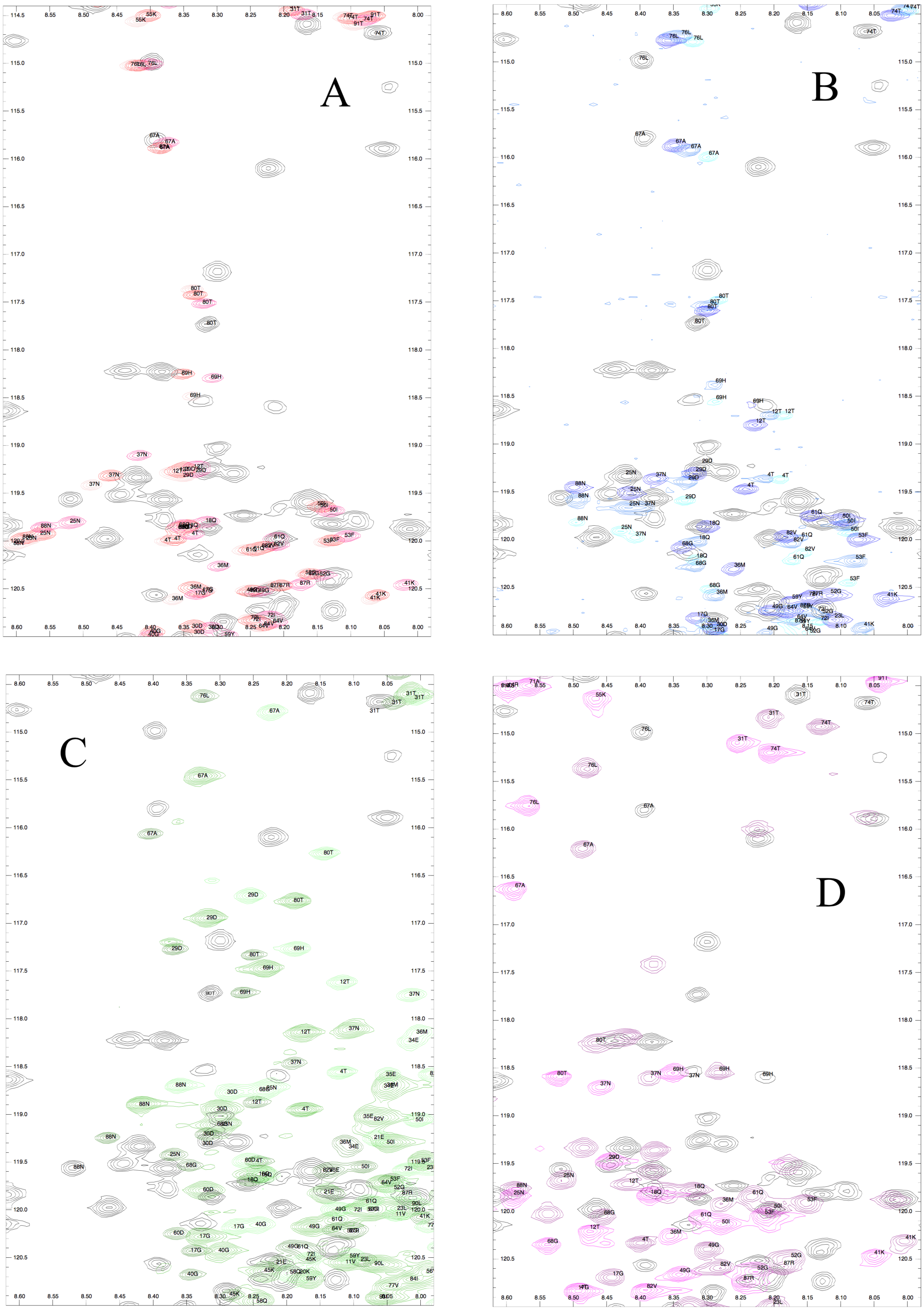

Figure S3: Zoom of the different ^{15}N-HSQC spectra of HIV-1-PR$_{\Delta 95\text{-}99}$. A) 0M, 4M, 6M, 8M urea, B) 0.75M, 1M, 2M, 4M GdmCl, C) 0%, 9%, 25%, 45% acetic acid, D) 25, 15 and 5 degrees. Peaks for 17G, 40G, 55K, 67A, 71A and 76L are folded in the ^{15}N-dimension.

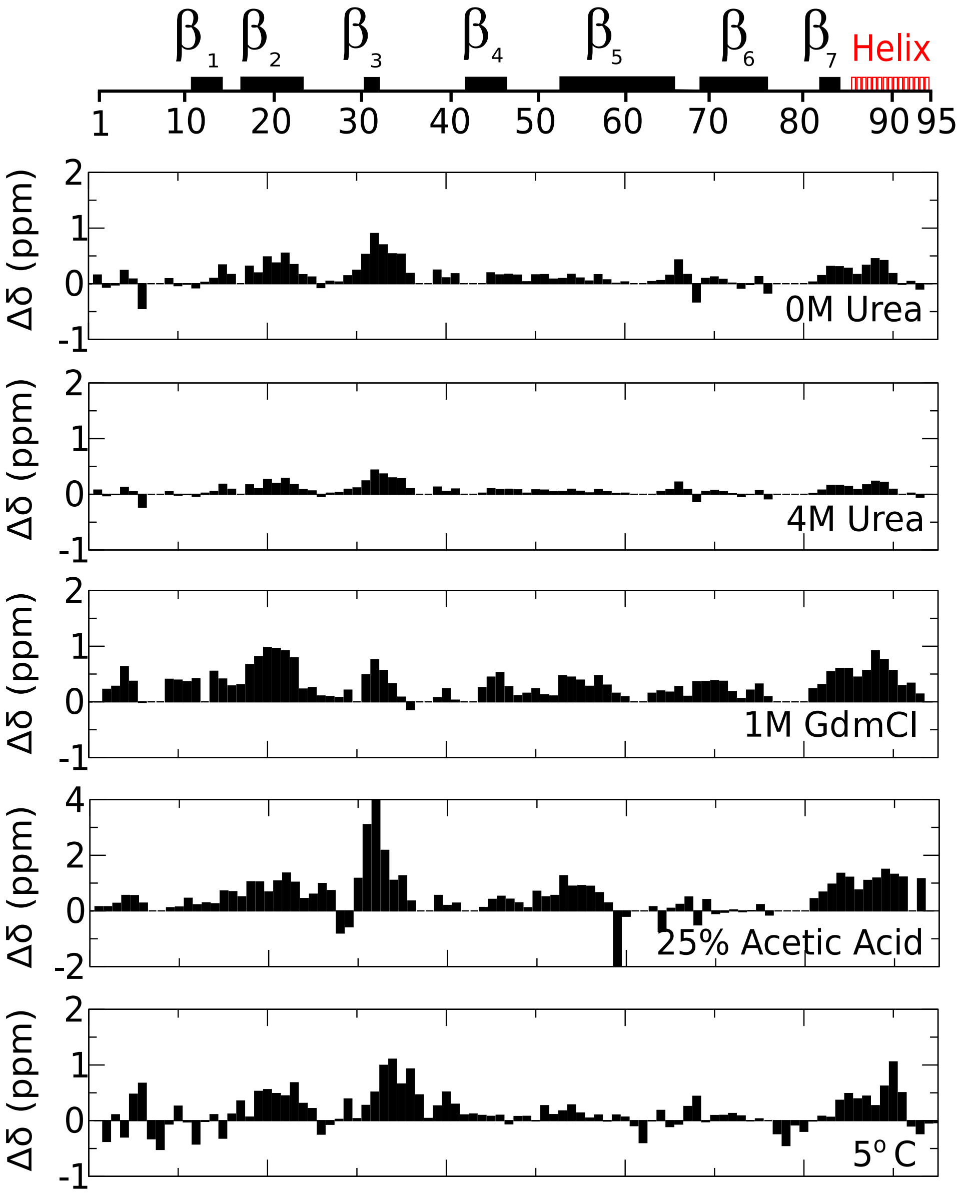

Figure S4: Secondary chemical shifts analysis calculated by combining C^{α} , C' and ^{15}N chemical shifts assigned for HIV-1-$PR_{\Delta 95\text{-}99}$ as described by Reed *et al.*

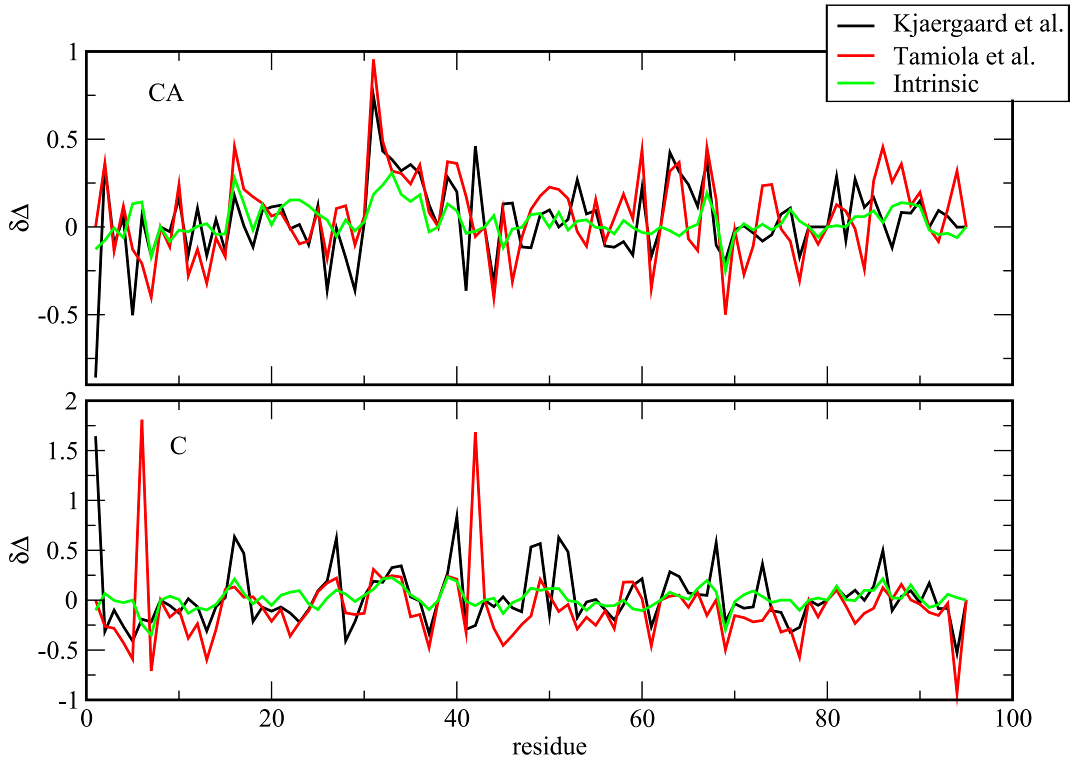

Figure S5: Secondary chemical shifts obtained with three different reference values for the denatured state of HIV-1-PR$_{\Delta 95\text{-}99}$ in urea.

Figure S6. In the following pages, the chemical shift and the linear fit applied to all assigned amino acids.

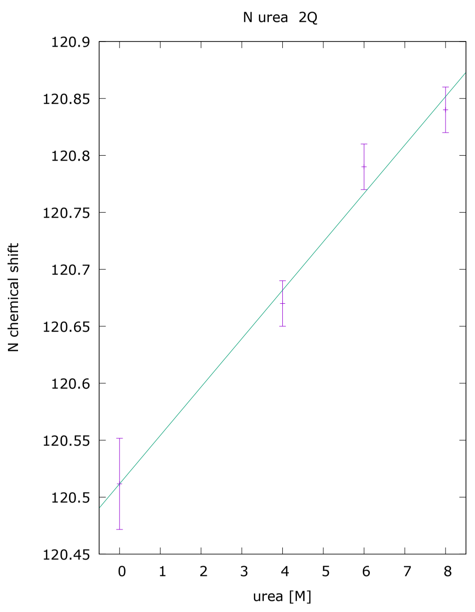
N urea 2Q
N chemical shift
urea [M]

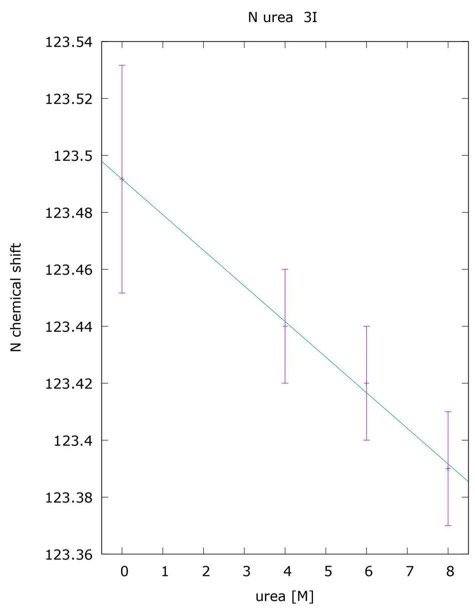
N urea 3I
N chemical shift
urea [M]

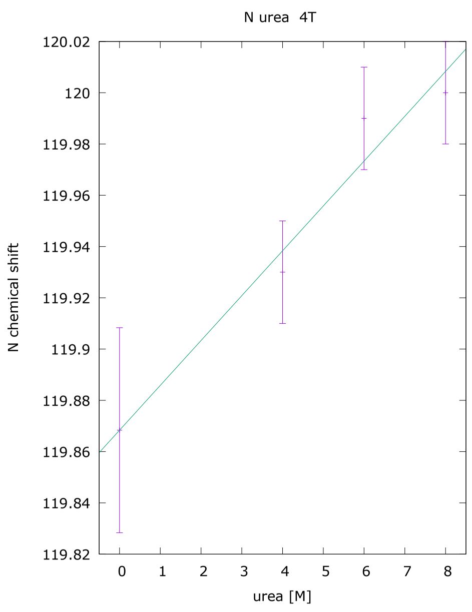
N urea 4T
N chemical shift
urea [M]

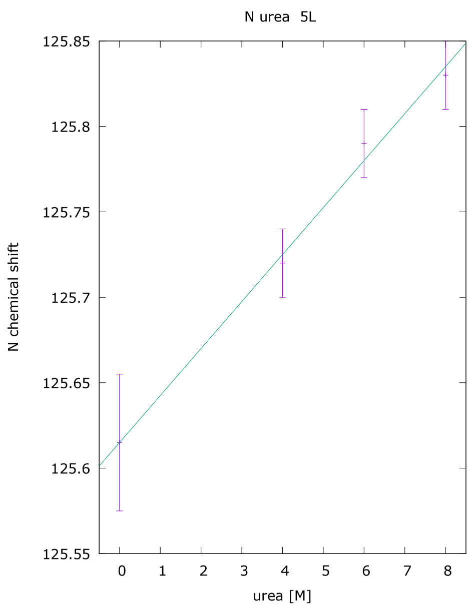
N urea 5L
N chemical shift
urea [M]

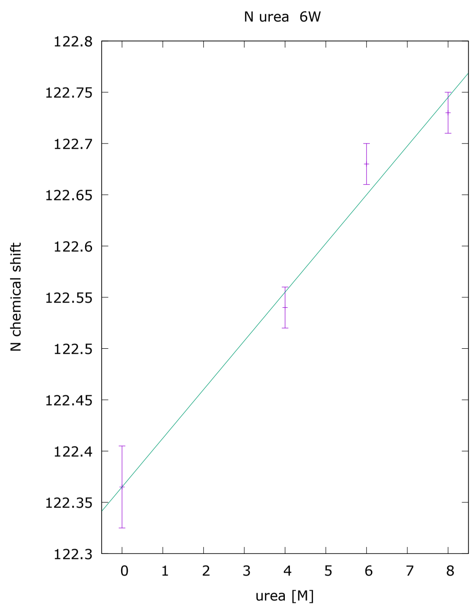
N urea 6W
N chemical shift
urea [M]

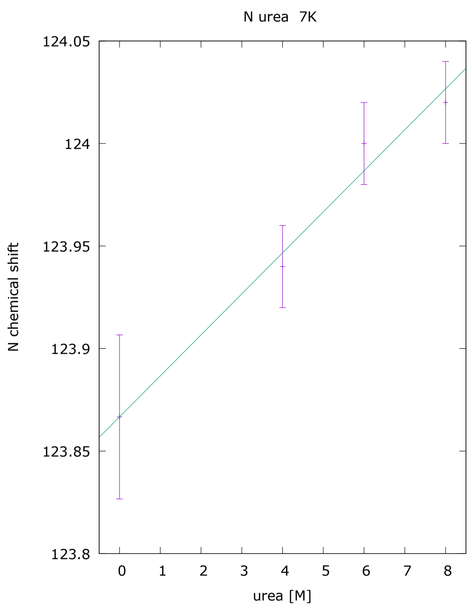
N urea 7K
N chemical shift
urea [M]

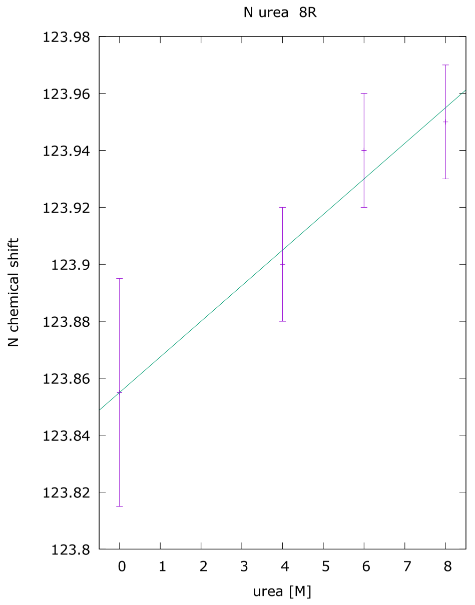
N urea 8R
N chemical shift
urea [M]

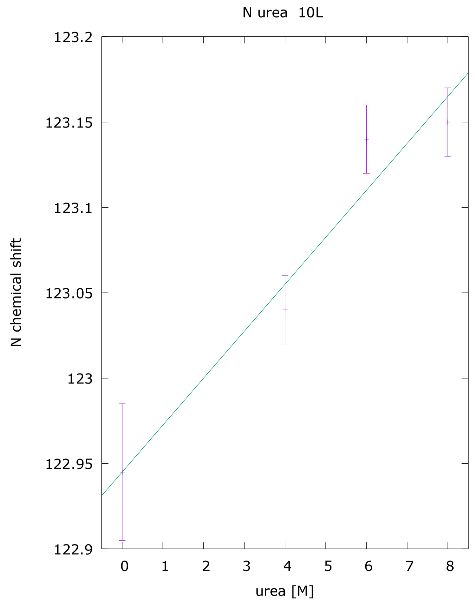
N urea 10L
N chemical shift
urea [M]

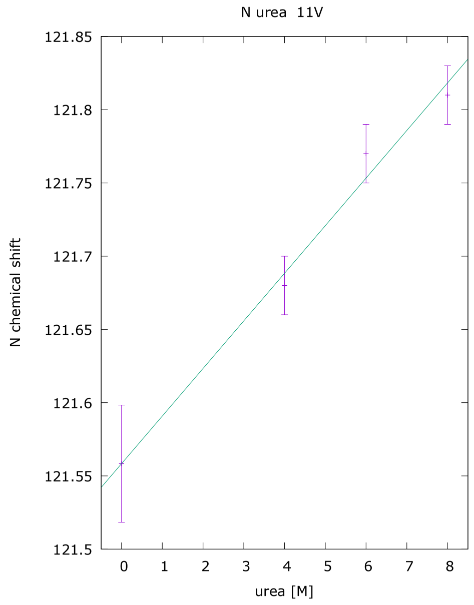
N urea 11V
N chemical shift
urea [M]

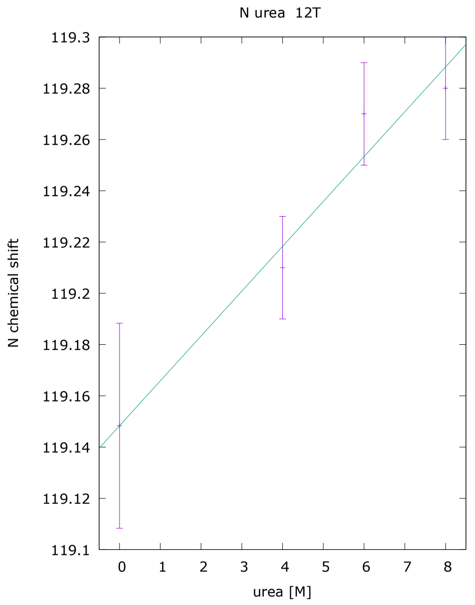
N urea 12T
N chemical shift
urea [M]

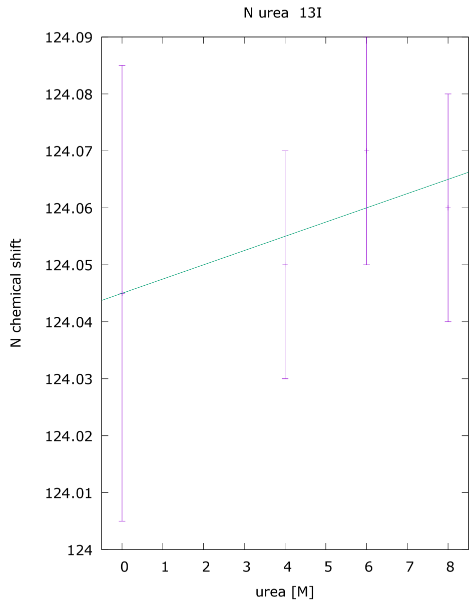
N urea 13I
N chemical shift
urea [M]

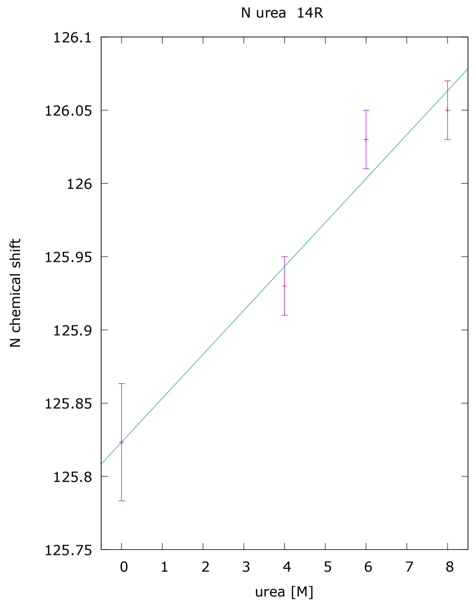
N urea 14R
N chemical shift
urea [M]

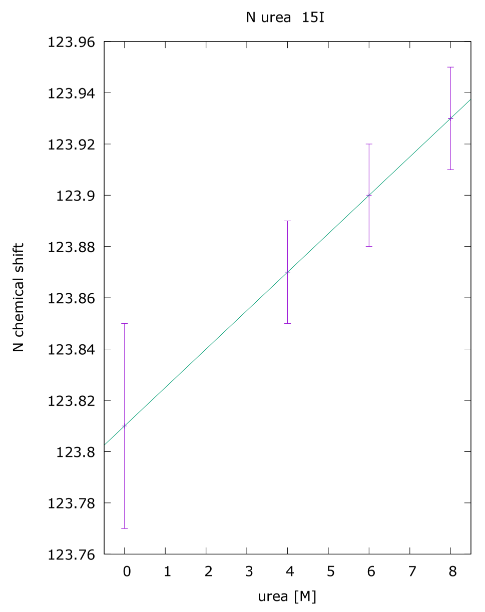
N urea 15I
N chemical shift
urea [M]

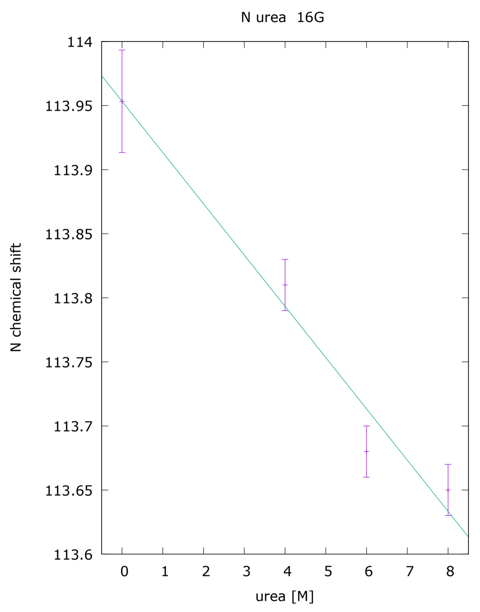
N urea 16G
N chemical shift
urea [M]

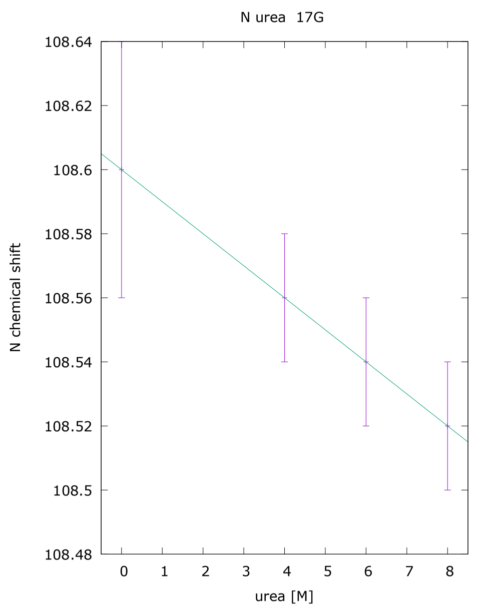
N urea 17G
N chemical shift
urea [M]

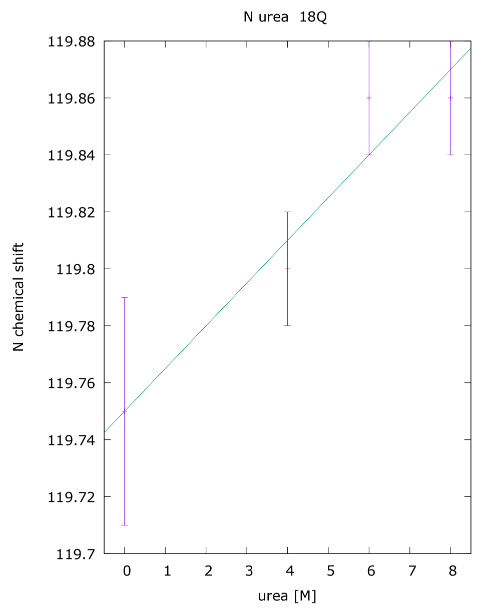
N urea 18Q
N chemical shift
urea [M]

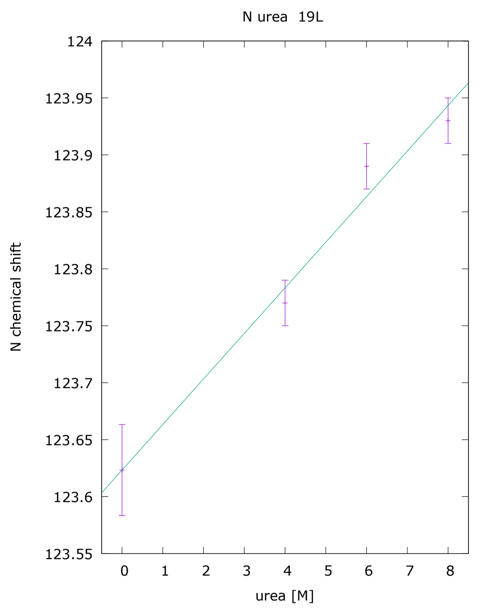
N urea 19L
N chemical shift
urea [M]

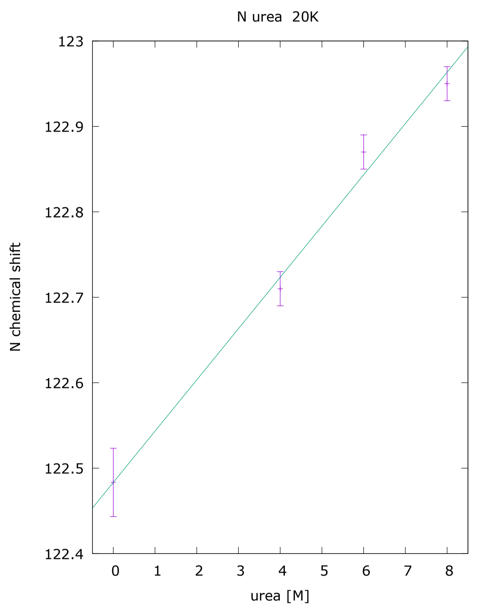
N urea 20K
N chemical shift
urea [M]

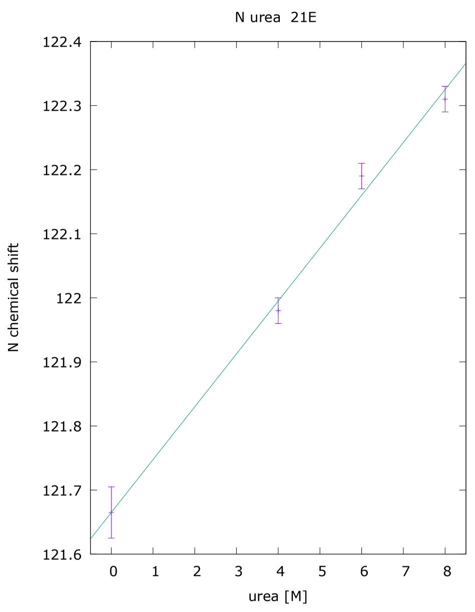
N urea 21E
N chemical shift
urea [M]

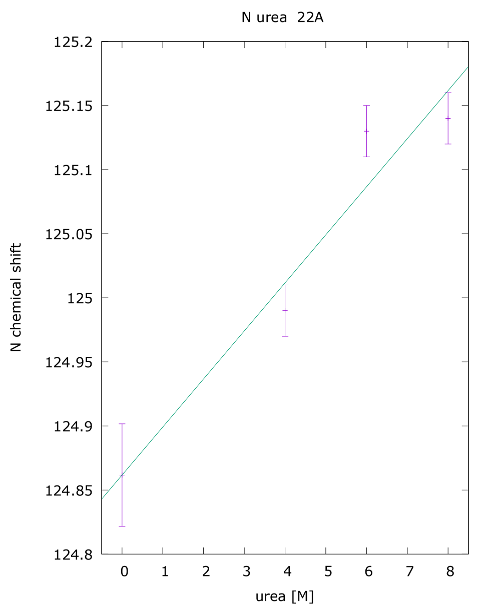
N urea 22A
N chemical shift
urea [M]

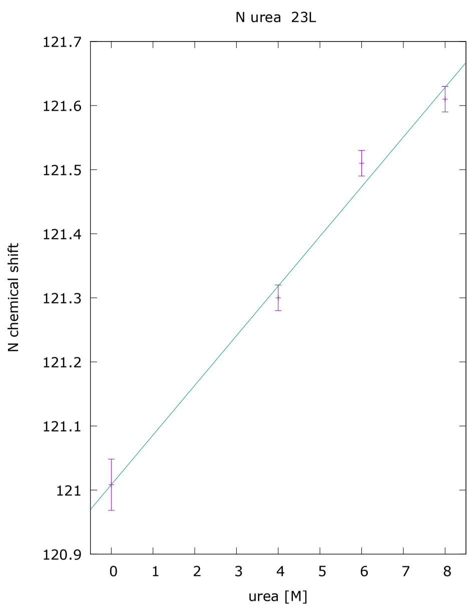
N urea 23L
N chemical shift
urea [M]

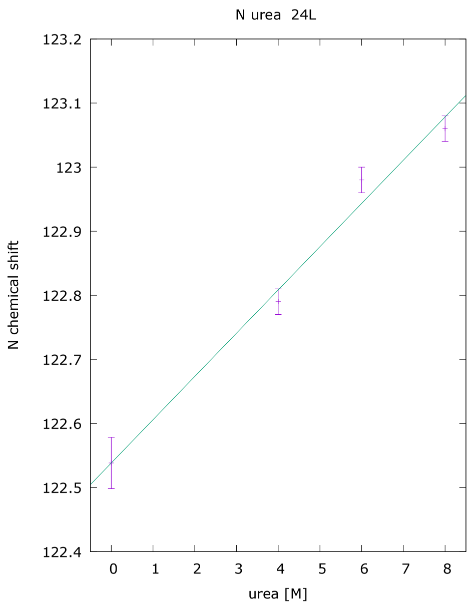
N urea 24L
N chemical shift
urea [M]

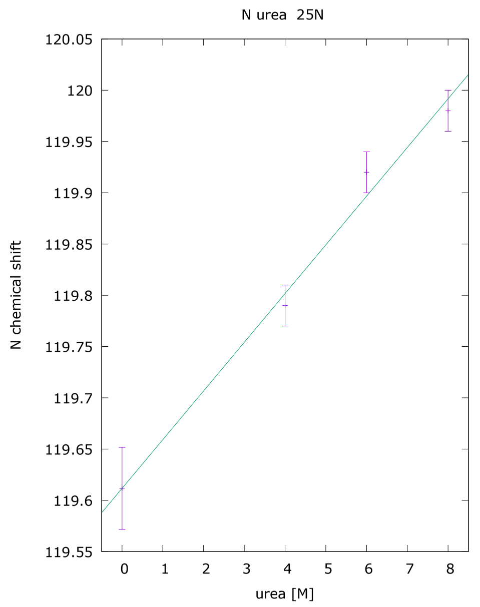
N urea 25N
N chemical shift
urea [M]

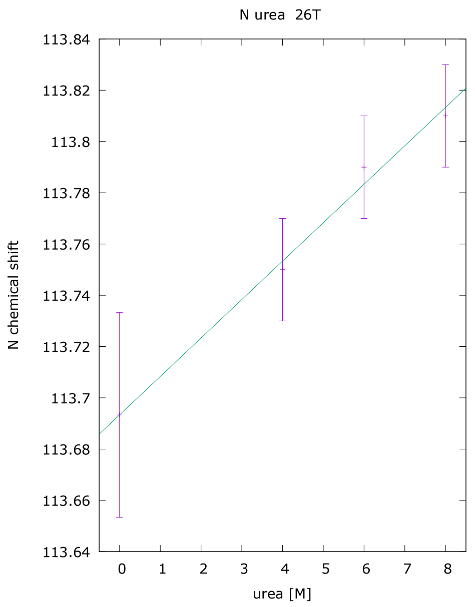
N urea 26T
N chemical shift
urea [M]

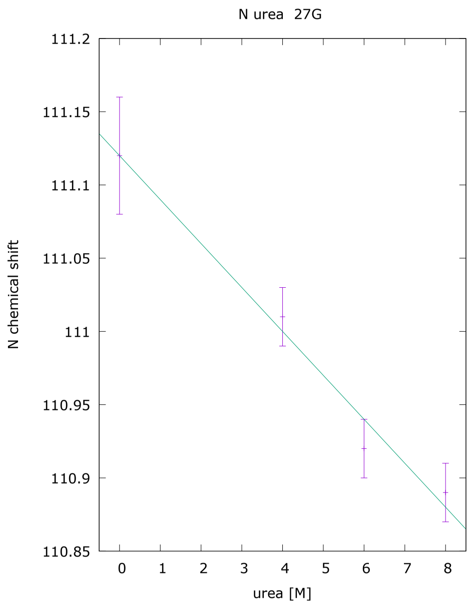
N urea 27G
N chemical shift
urea [M]
111.2
111.15
111.1
111.05
111
110.95
110.9
110.85
0 1 2 3 4 5 6 7 8

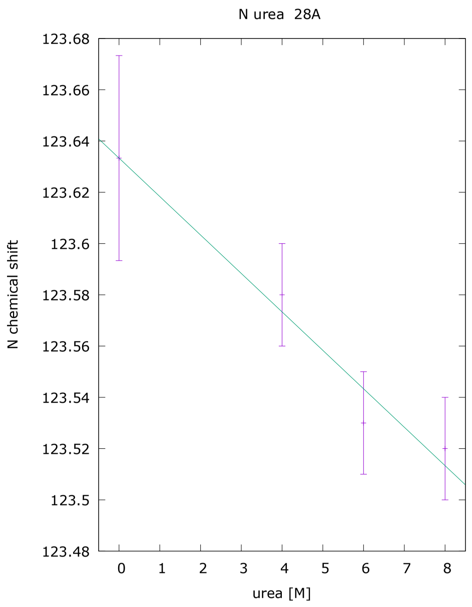
N urea 28A
N chemical shift
urea [M]
123.68
123.66
123.64
123.62
123.6
123.58
123.56
123.54
123.52
123.5
123.48
0 1 2 3 4 5 6 7 8

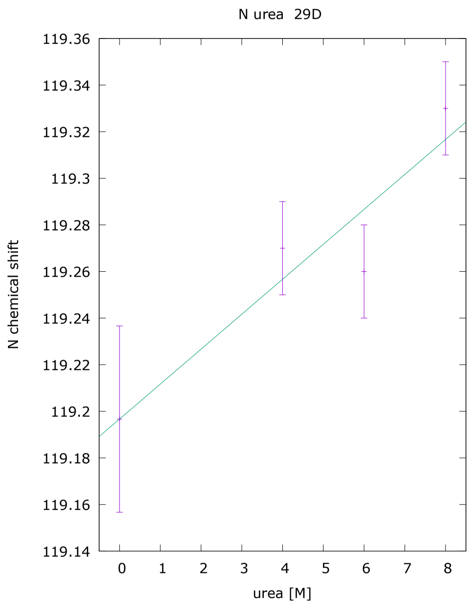
N urea 29D
N chemical shift
urea [M]
119.36
119.34
119.32
119.3
119.28
119.26
119.24
119.22
119.2
119.18
119.16
119.14
0 1 2 3 4 5 6 7 8

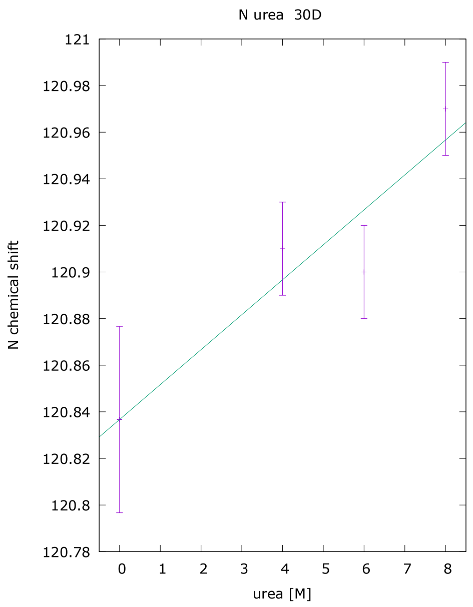
N urea 30D
N chemical shift
urea [M]
121
120.98
120.96
120.94
120.92
120.9
120.88
120.86
120.84
120.82
120.8
120.78
0 1 2 3 4 5 6 7 8

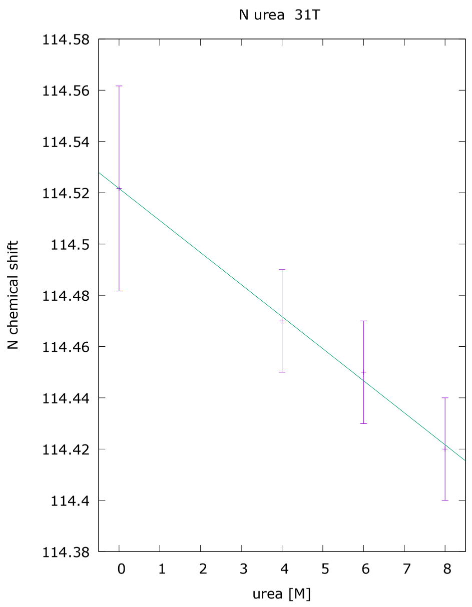
N urea 31T
N chemical shift
urea [M]
114.58
114.56
114.54
114.52
114.5
114.48
114.46
114.44
114.42
114.4
114.38
0 1 2 3 4 5 6 7 8

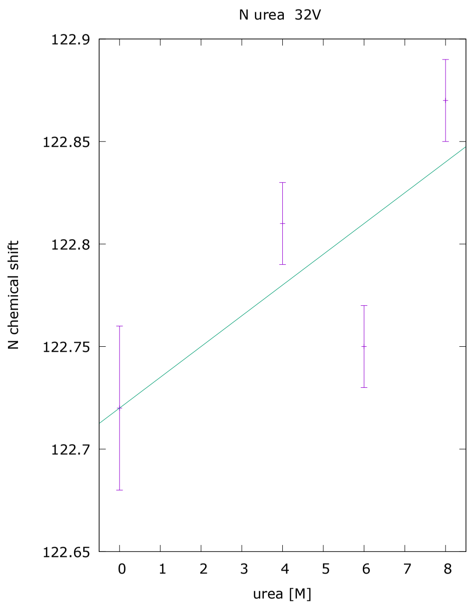
N urea 32V
N chemical shift
urea [M]
122.9
122.85
122.8
122.75
122.7
122.65
0 1 2 3 4 5 6 7 8

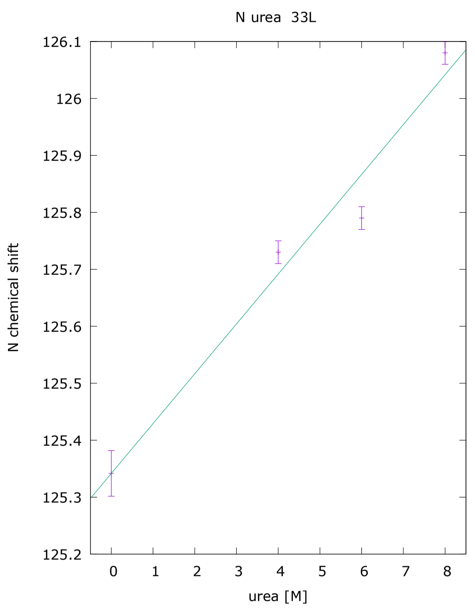
N urea 33L
N chemical shift
urea [M]

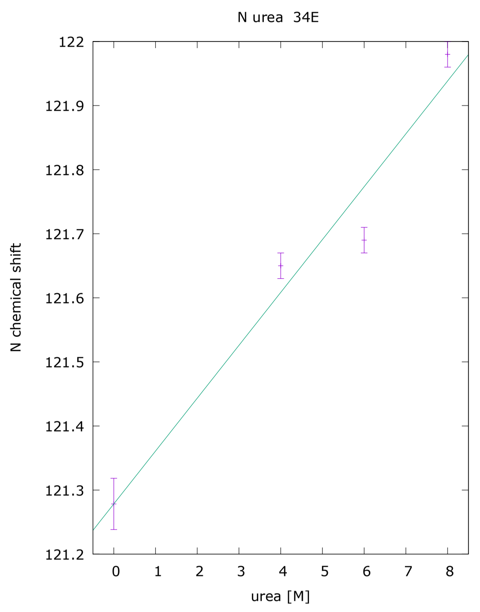
N urea 34E
N chemical shift
urea [M]

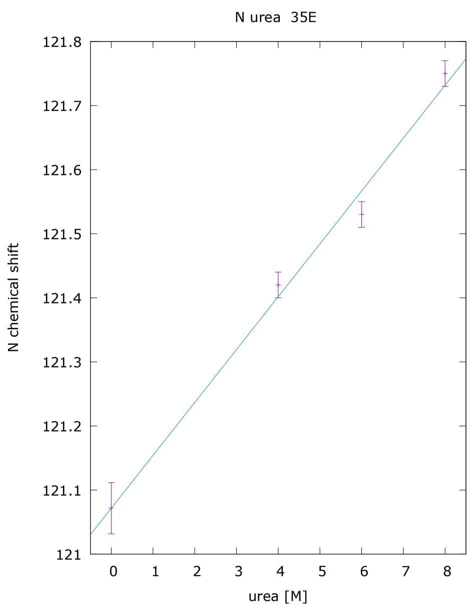
N urea 35E
N chemical shift
urea [M]

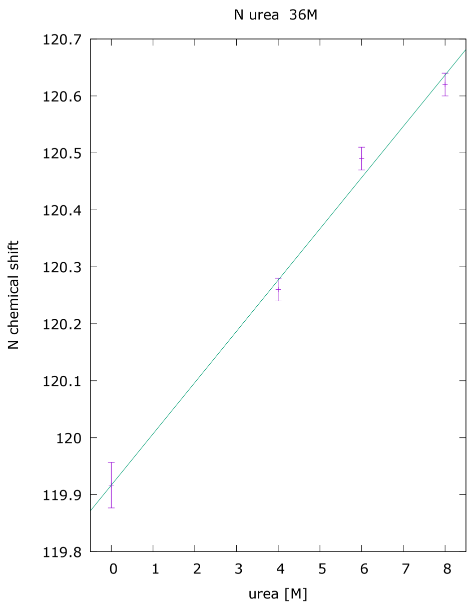
N urea 36M
N chemical shift
urea [M]

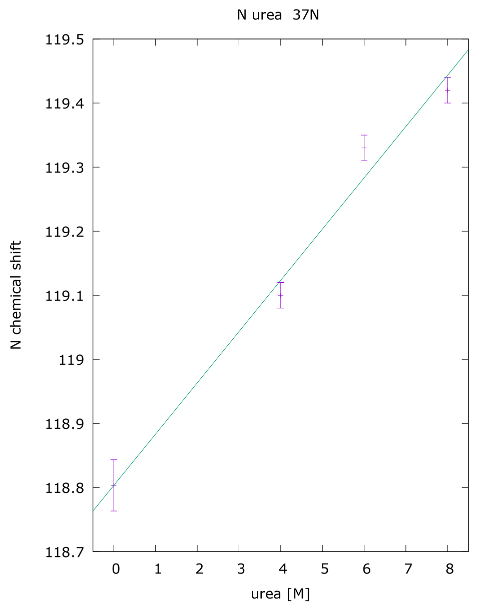
N urea 37N
N chemical shift
urea [M]

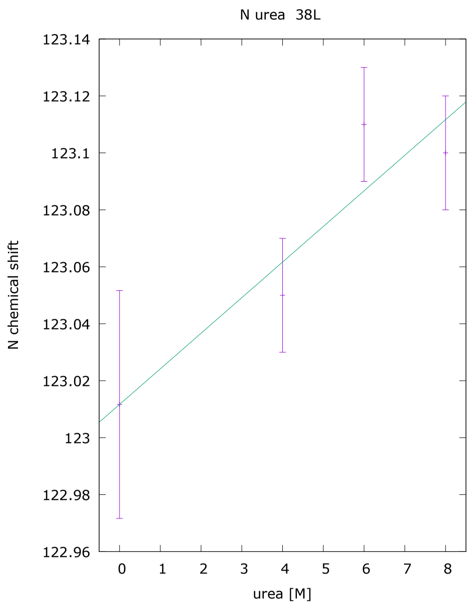
N urea 38L
N chemical shift
urea [M]

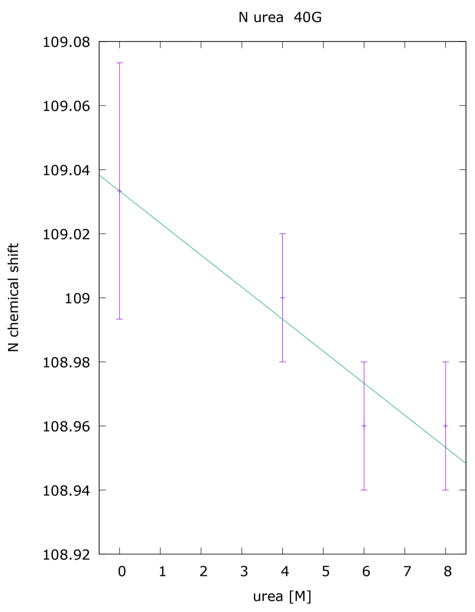
N urea 40G
N chemical shift
urea [M]

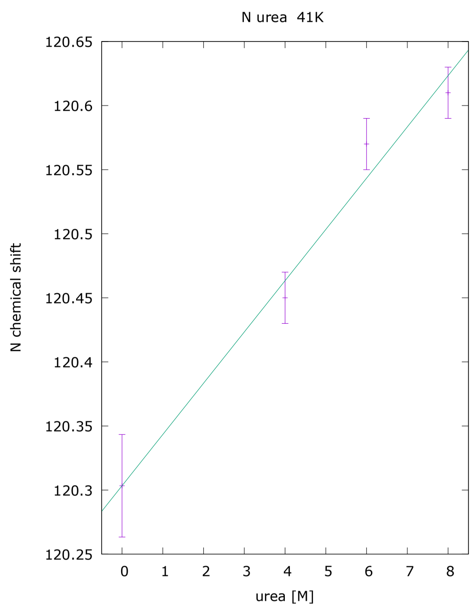
N urea 41K
N chemical shift
urea [M]

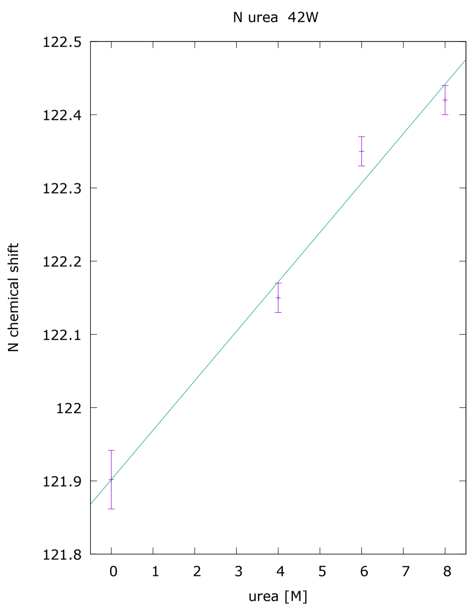
N urea 42W
N chemical shift
urea [M]

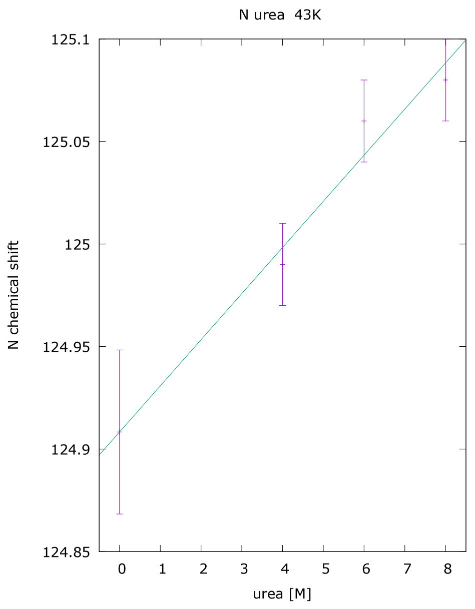
N urea 43K
N chemical shift
urea [M]

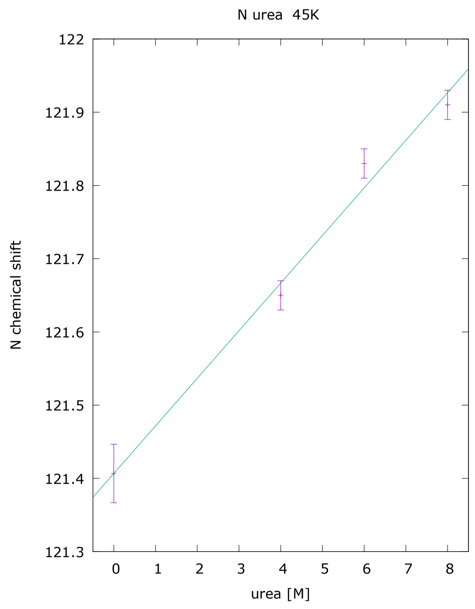
N urea 45K
N chemical shift
urea [M]

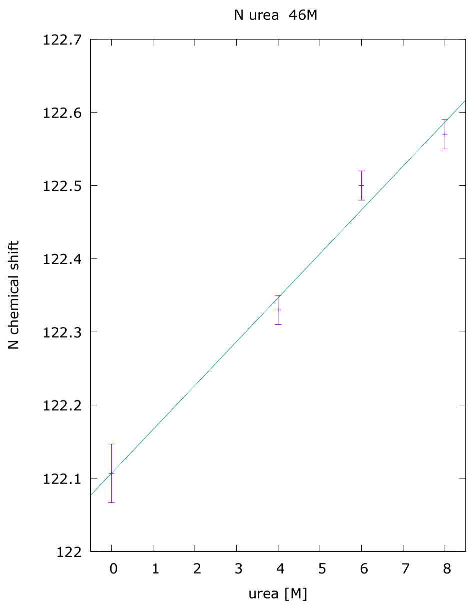
N urea 46M
N chemical shift
urea [M]

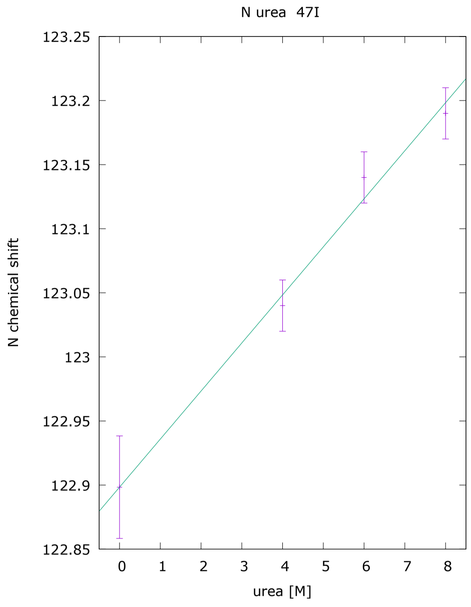
N urea 47I
N chemical shift
123.25
123.2
123.15
123.1
123.05
123
122.95
122.9
122.85
0 1 2 3 4 5 6 7 8
urea [M]

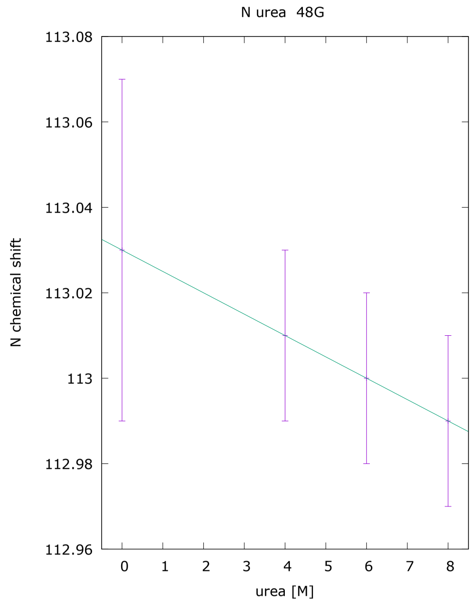
N urea 48G
N chemical shift
113.08
113.06
113.04
113.02
113
112.98
112.96
0 1 2 3 4 5 6 7 8
urea [M]

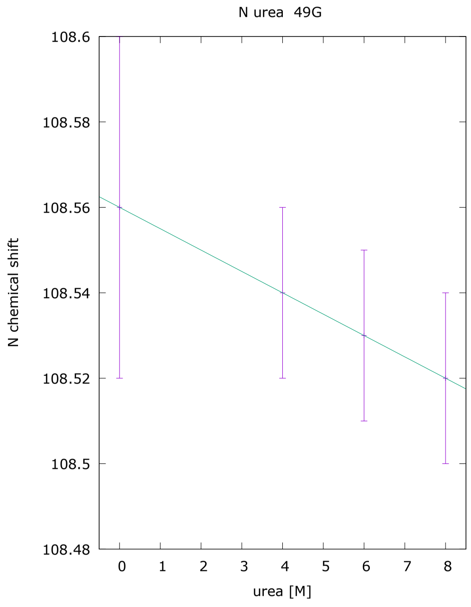
N urea 49G
N chemical shift
108.6
108.58
108.56
108.54
108.52
108.5
108.48
0 1 2 3 4 5 6 7 8
urea [M]

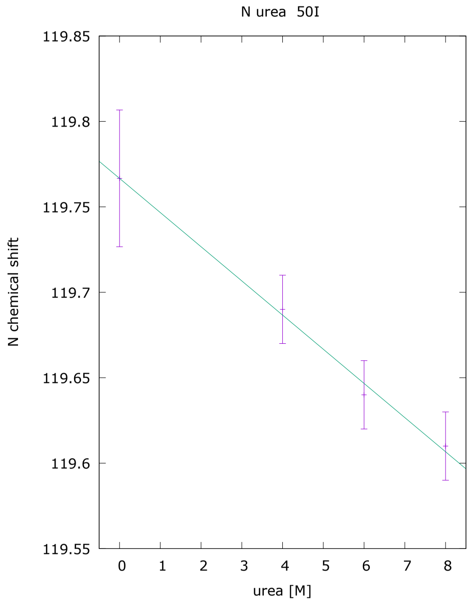
N urea 50I
N chemical shift
119.85
119.8
119.75
119.7
119.65
119.6
119.55
0 1 2 3 4 5 6 7 8
urea [M]

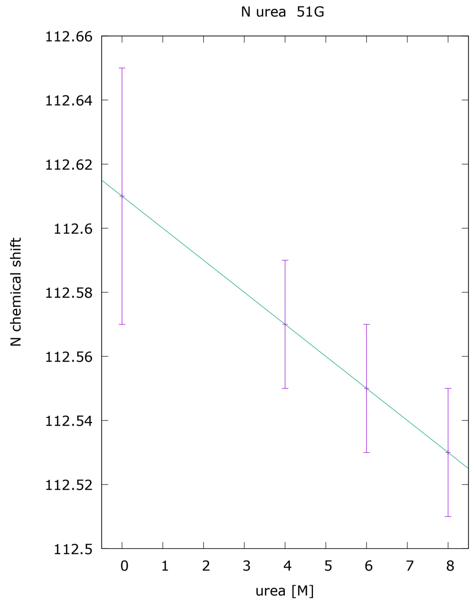
N urea 51G
N chemical shift
112.66
112.64
112.62
112.6
112.58
112.56
112.54
112.52
112.5
0 1 2 3 4 5 6 7 8
urea [M]

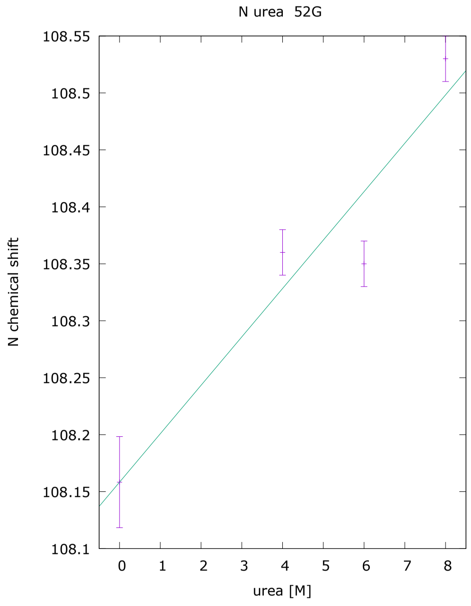
N urea 52G
N chemical shift
108.55
108.5
108.45
108.4
108.35
108.3
108.25
108.2
108.15
108.1
0 1 2 3 4 5 6 7 8
urea [M]

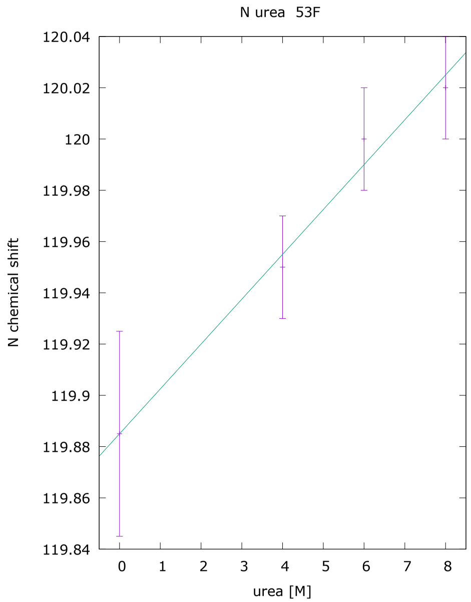
N urea 53F
N chemical shift
urea [M]

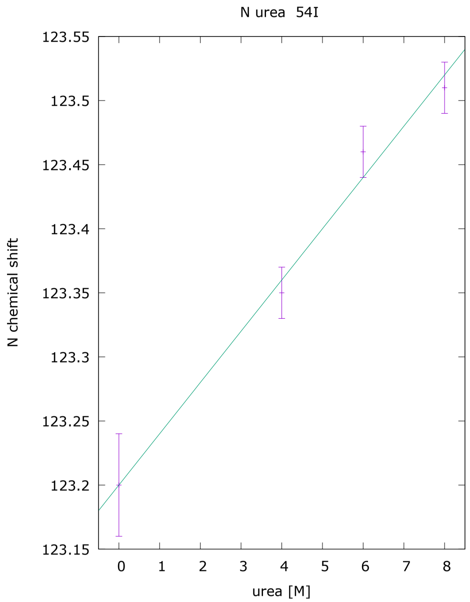
N urea 54I
N chemical shift
urea [M]

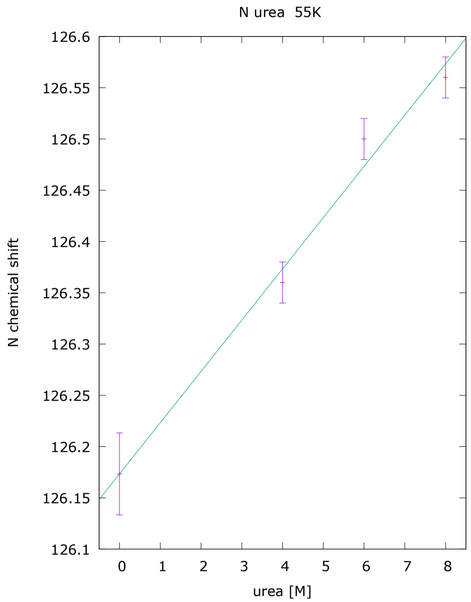
N urea 55K
N chemical shift
urea [M]

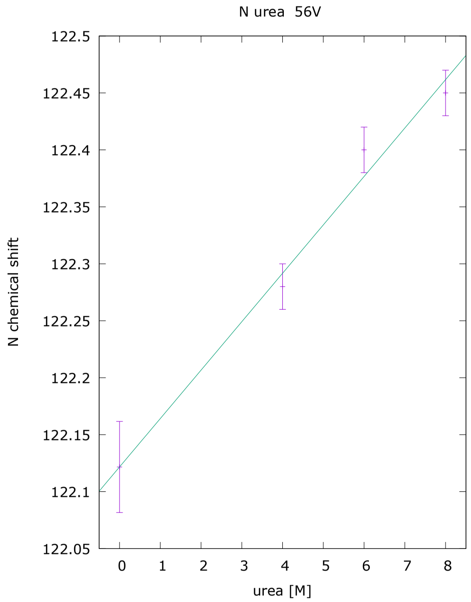
N urea 56V
N chemical shift
urea [M]

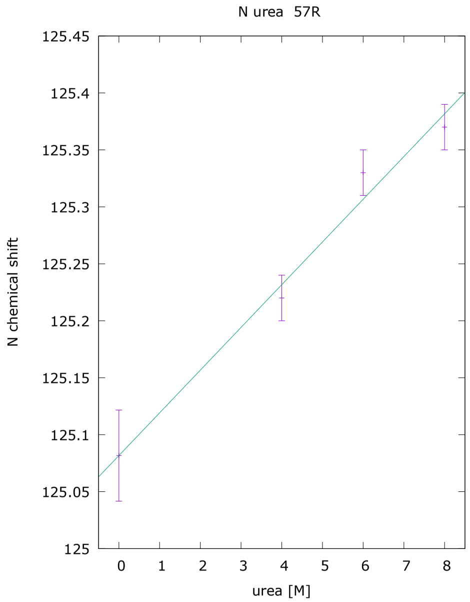
N urea 57R
N chemical shift
urea [M]

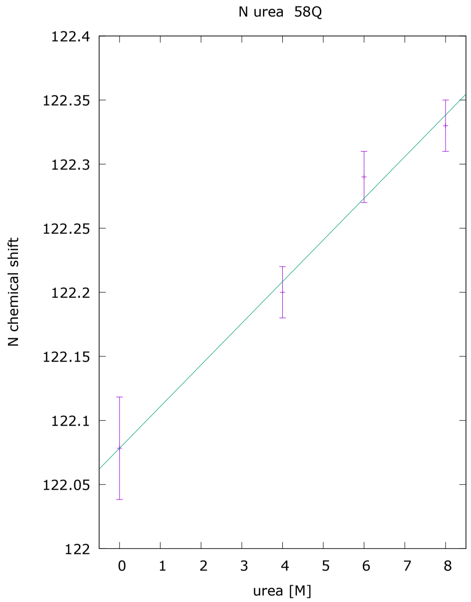
N urea 58Q
N chemical shift
urea [M]

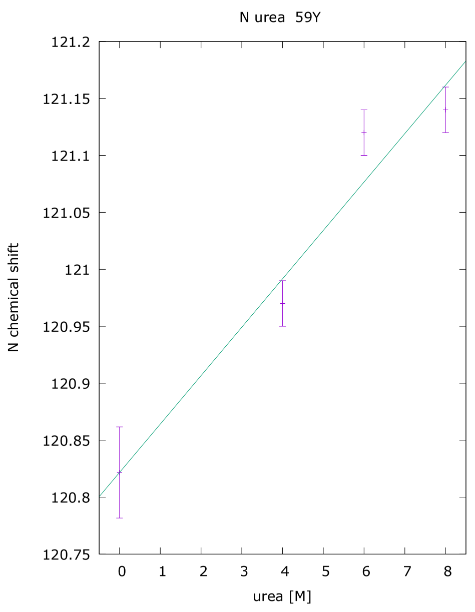
N urea 59Y
N chemical shift
urea [M]

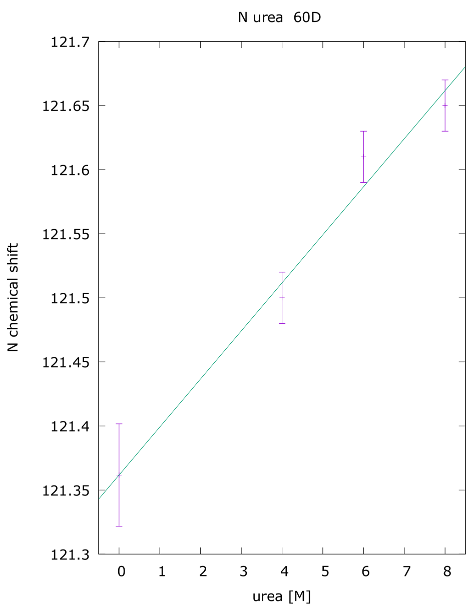
N urea 60D
N chemical shift
urea [M]

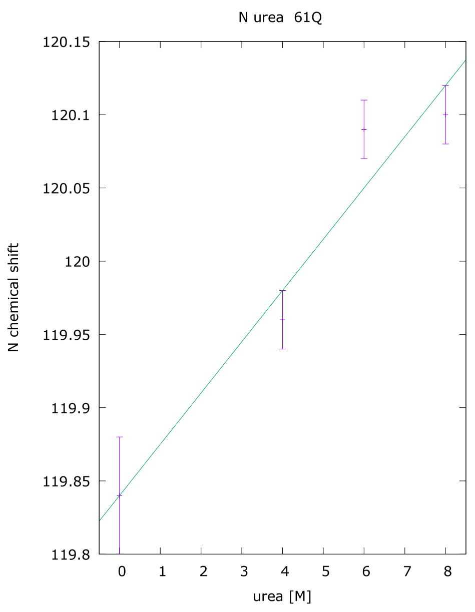
N urea 61Q
N chemical shift
urea [M]

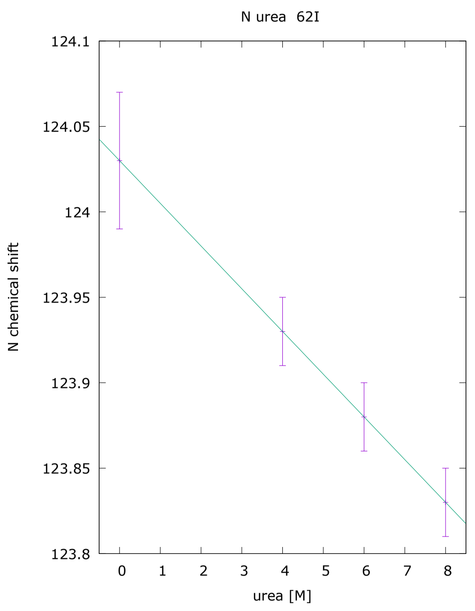
N urea 62I
N chemical shift
urea [M]

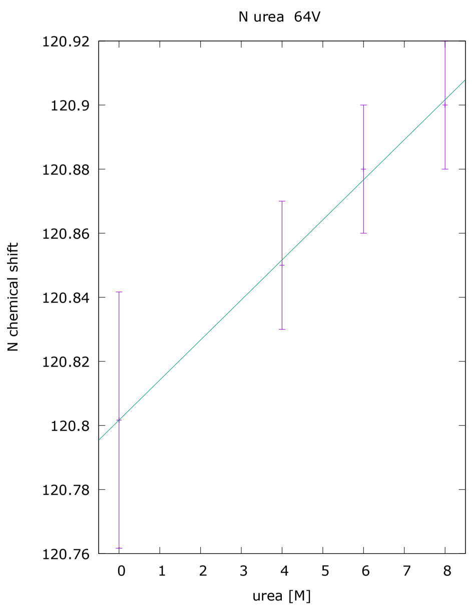
N urea 64V
N chemical shift
urea [M]

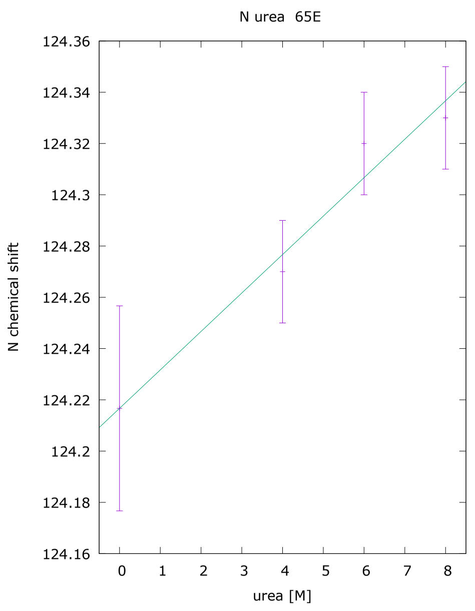
N urea 65E
N chemical shift
urea [M]

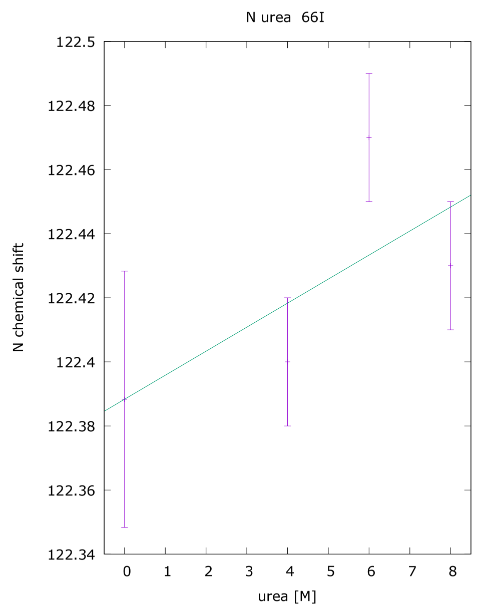
N urea 66I
N chemical shift
122.5
122.48
122.46
122.44
122.42
122.4
122.38
122.36
122.34
0 1 2 3 4 5 6 7 8
urea [M]

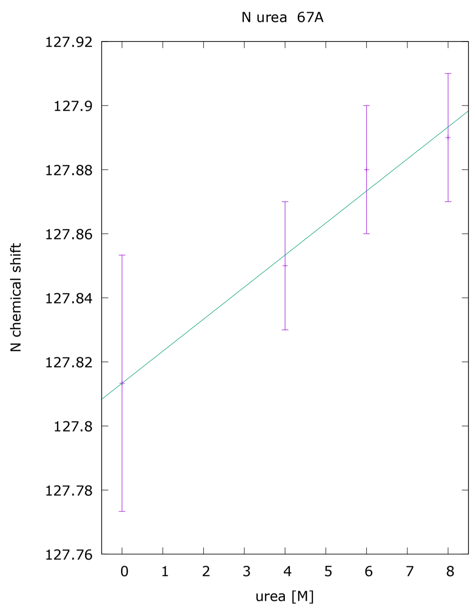
N urea 67A
N chemical shift
127.92
127.9
127.88
127.86
127.84
127.82
127.8
127.78
127.76
0 1 2 3 4 5 6 7 8
urea [M]

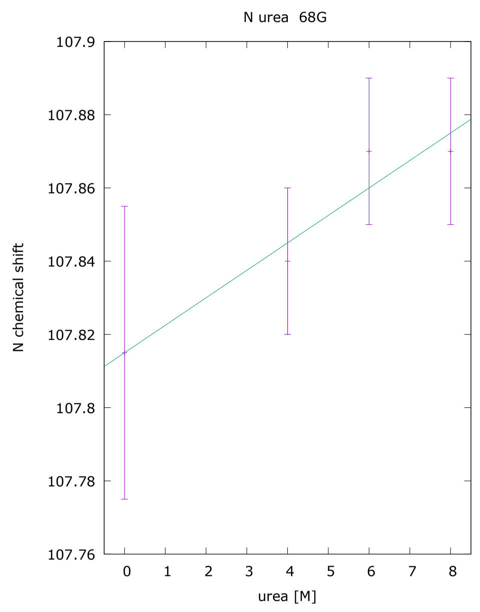
N urea 68G
N chemical shift
107.9
107.88
107.86
107.84
107.82
107.8
107.78
107.76
0 1 2 3 4 5 6 7 8
urea [M]

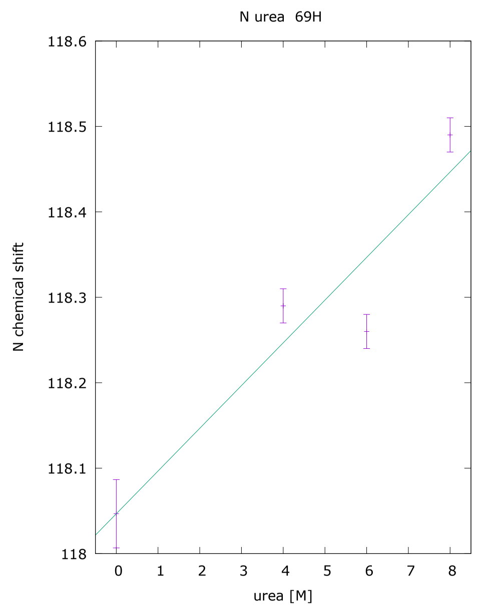
N urea 69H
N chemical shift
118.6
118.5
118.4
118.3
118.2
118.1
118
0 1 2 3 4 5 6 7 8
urea [M]

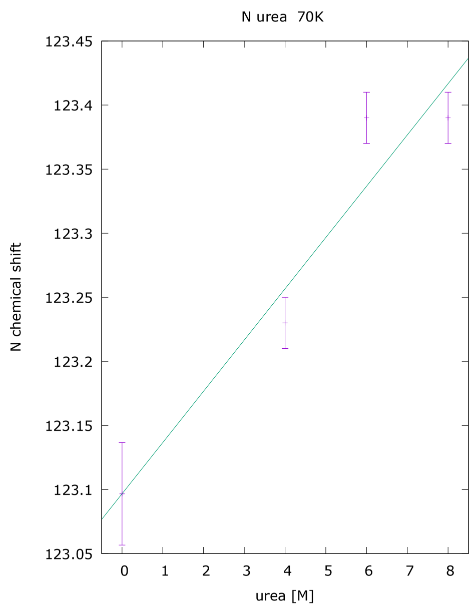
N urea 70K
N chemical shift
123.45
123.4
123.35
123.3
123.25
123.2
123.15
123.1
123.05
0 1 2 3 4 5 6 7 8
urea [M]

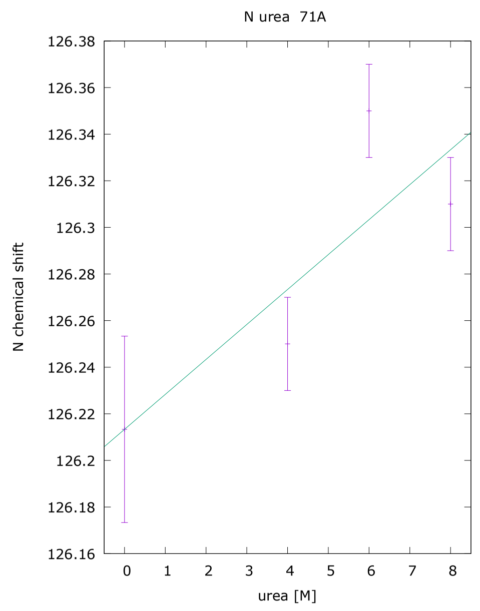
N urea 71A
N chemical shift
126.38
126.36
126.34
126.32
126.3
126.28
126.26
126.24
126.22
126.2
126.18
126.16
0 1 2 3 4 5 6 7 8
urea [M]

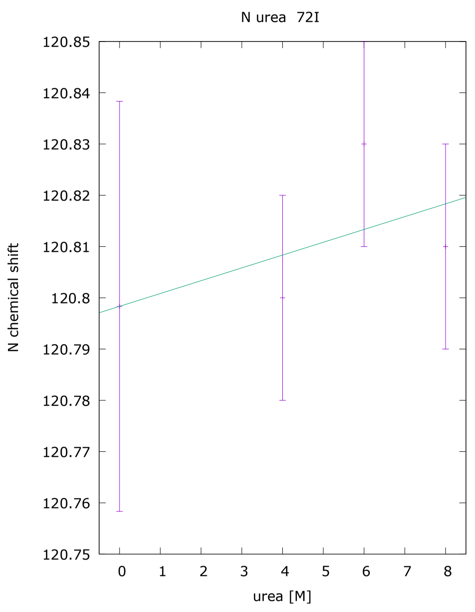
N urea 72I
N chemical shift
urea [M]

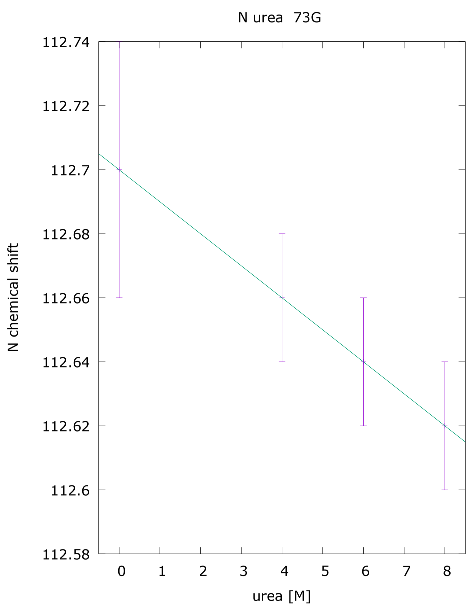
N urea 73G
N chemical shift
urea [M]

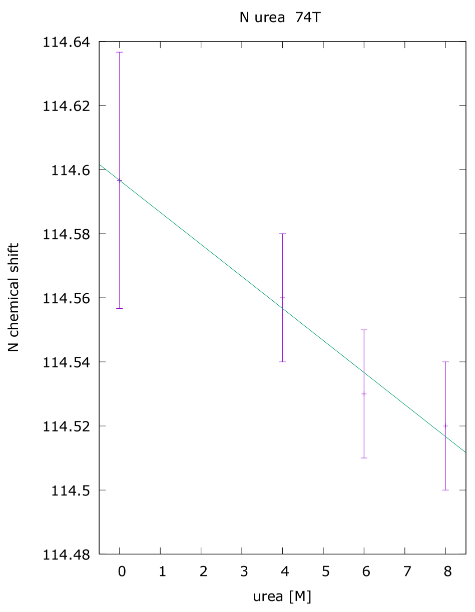
N urea 74T
N chemical shift
urea [M]

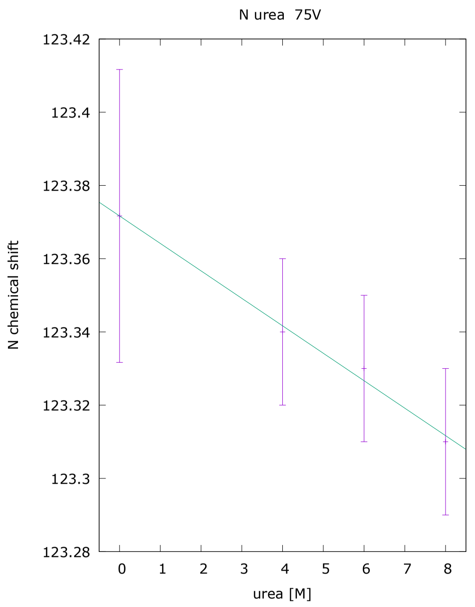
N urea 75V
N chemical shift
urea [M]

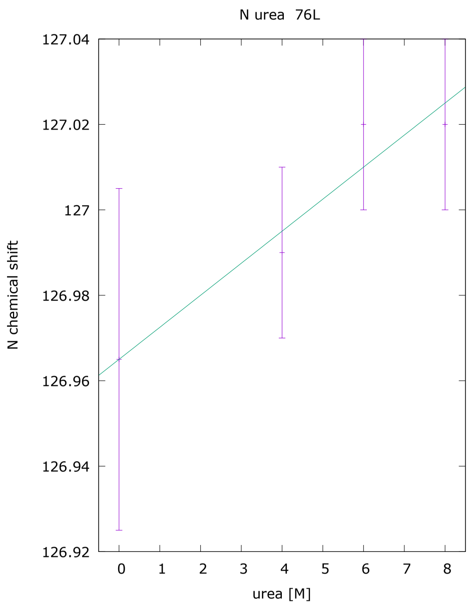
N urea 76L
N chemical shift
urea [M]

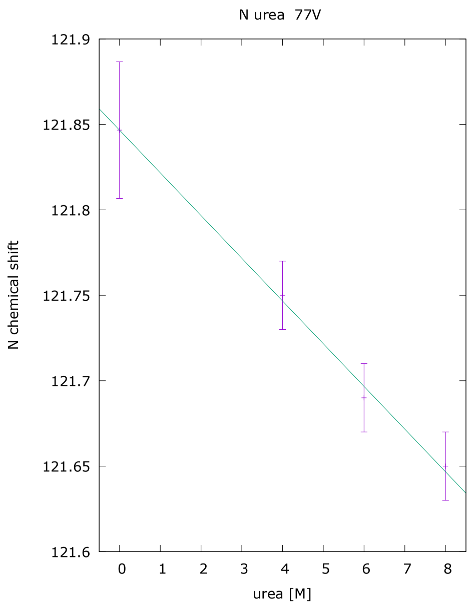
N urea 77V
N chemical shift
urea [M]

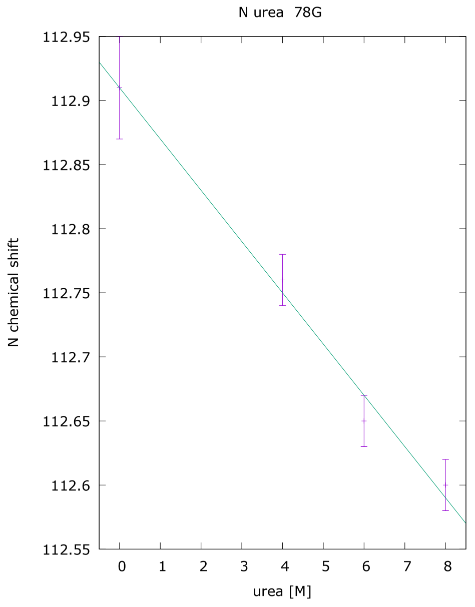
N urea 78G
N chemical shift
112.95
112.9
112.85
112.8
112.75
112.7
112.65
112.6
112.55
0 1 2 3 4 5 6 7 8
urea [M]

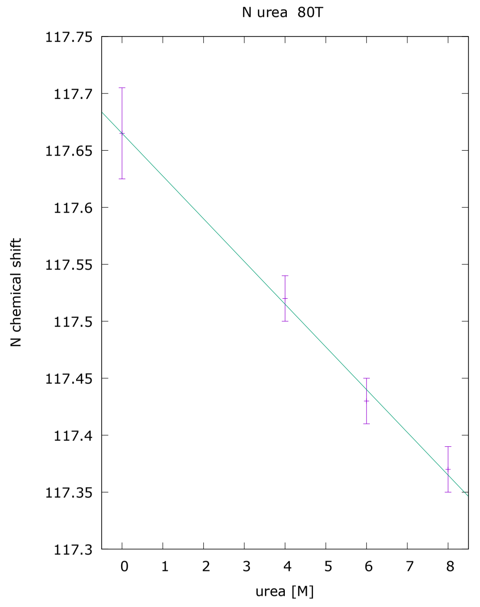
N urea 80T
N chemical shift
117.75
117.7
117.65
117.6
117.55
117.5
117.45
117.4
117.35
117.3
0 1 2 3 4 5 6 7 8
urea [M]

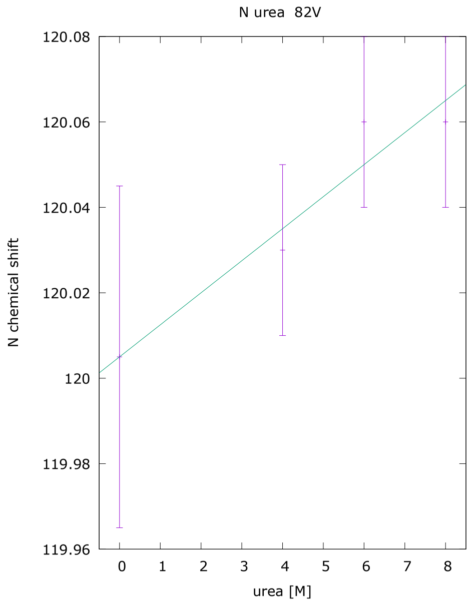
N urea 82V
N chemical shift
120.08
120.06
120.04
120.02
120
119.98
119.96
0 1 2 3 4 5 6 7 8
urea [M]

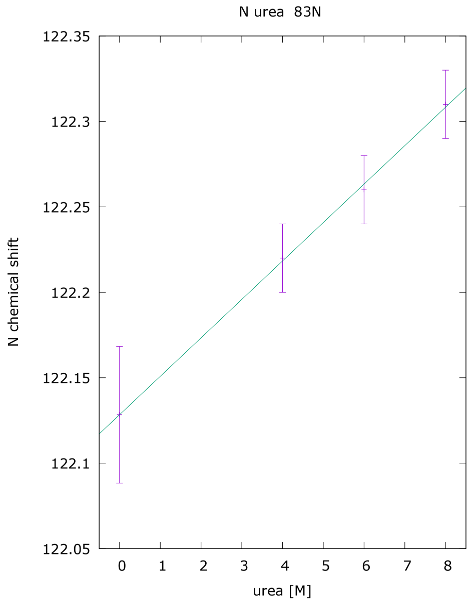
N urea 83N
N chemical shift
122.35
122.3
122.25
122.2
122.15
122.1
122.05
0 1 2 3 4 5 6 7 8
urea [M]

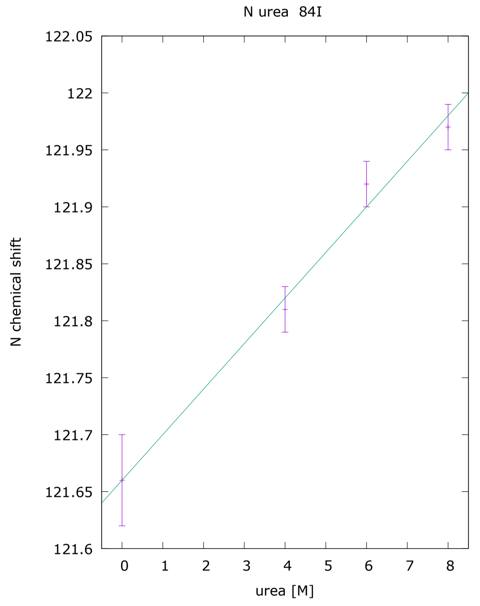
N urea 84I
N chemical shift
122.05
122
121.95
121.9
121.85
121.8
121.75
121.7
121.65
121.6
0 1 2 3 4 5 6 7 8
urea [M]

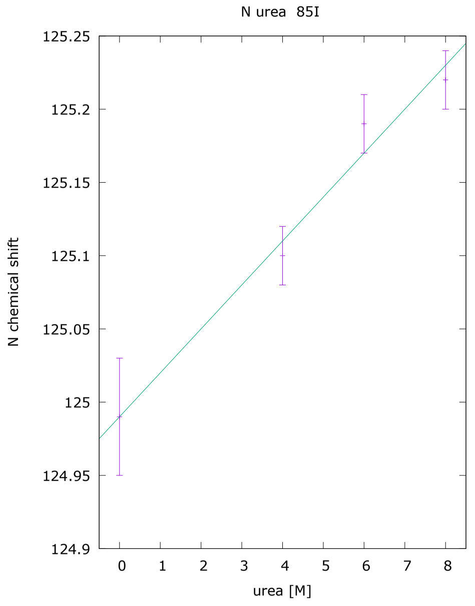
N urea 85I
N chemical shift
125.25
125.2
125.15
125.1
125.05
125
124.95
124.9
0 1 2 3 4 5 6 7 8
urea [M]

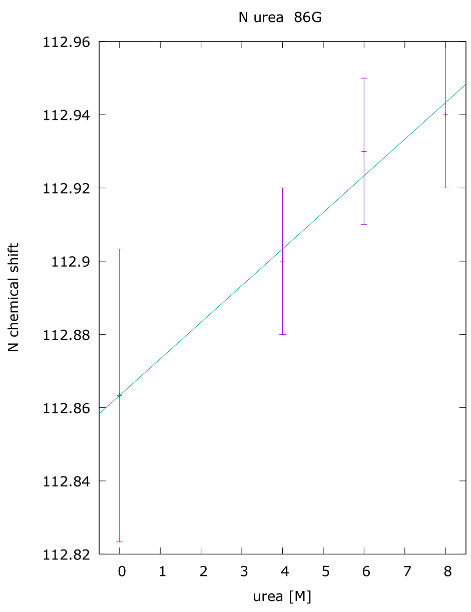
N urea 86G
N chemical shift
urea [M]

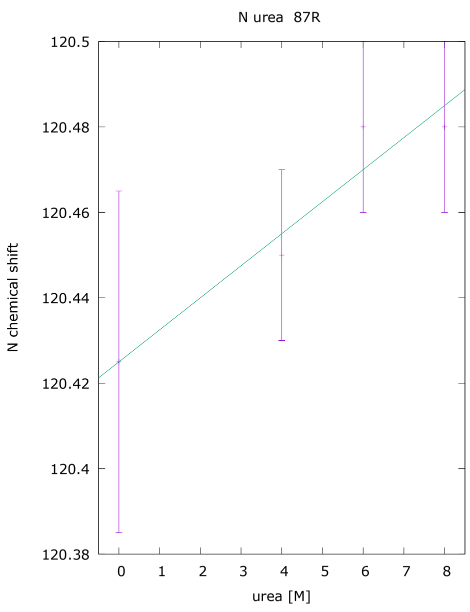
N urea 87R
N chemical shift
urea [M]

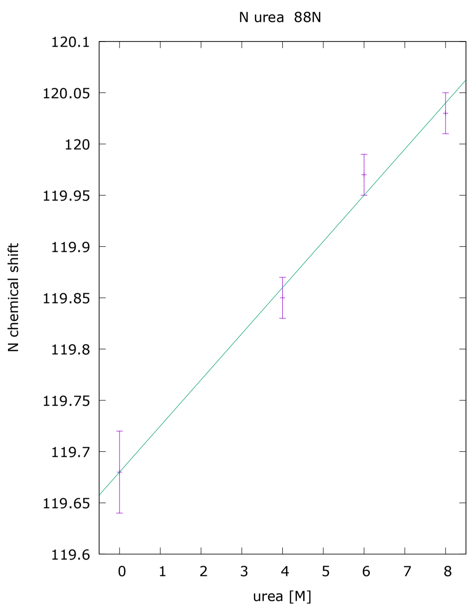
N urea 88N
N chemical shift
urea [M]

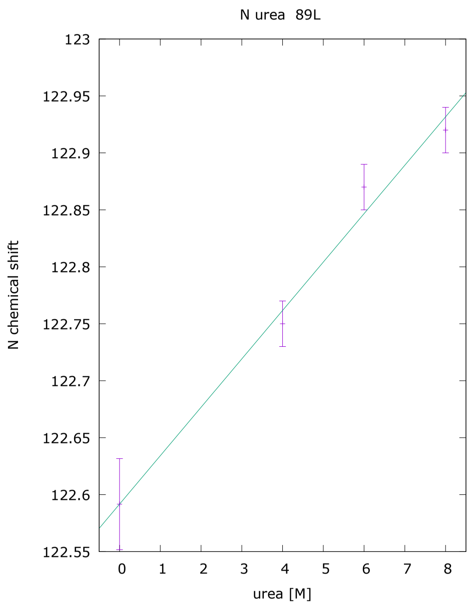
N urea 89L
N chemical shift
urea [M]

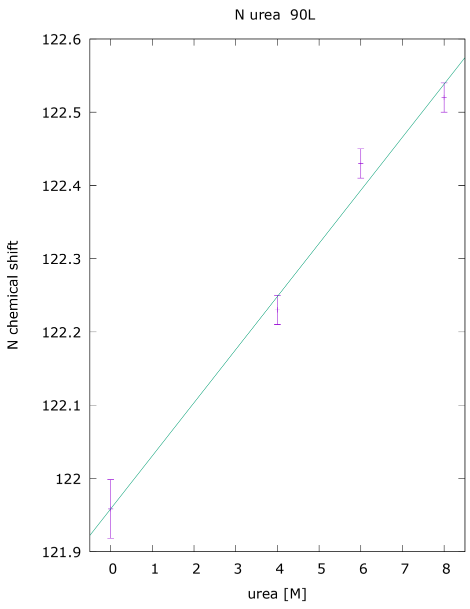
N urea 90L
N chemical shift
urea [M]

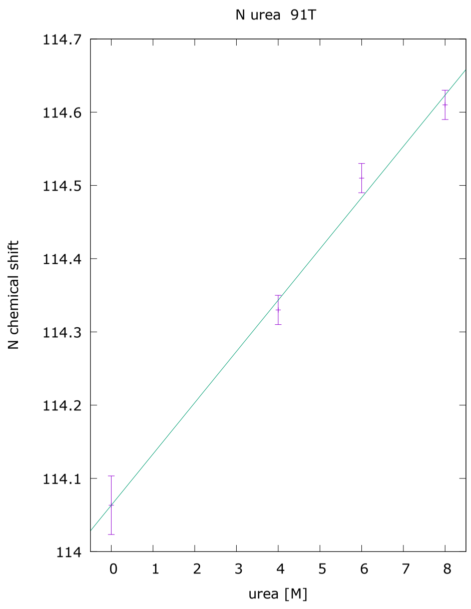
N urea 91T
N chemical shift
urea [M]

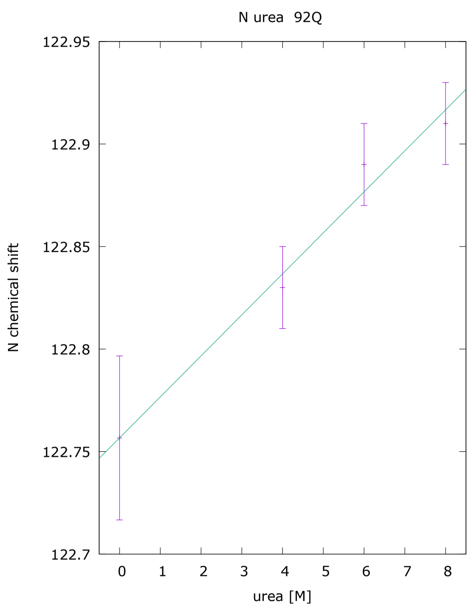
N urea 92Q
N chemical shift
urea [M]

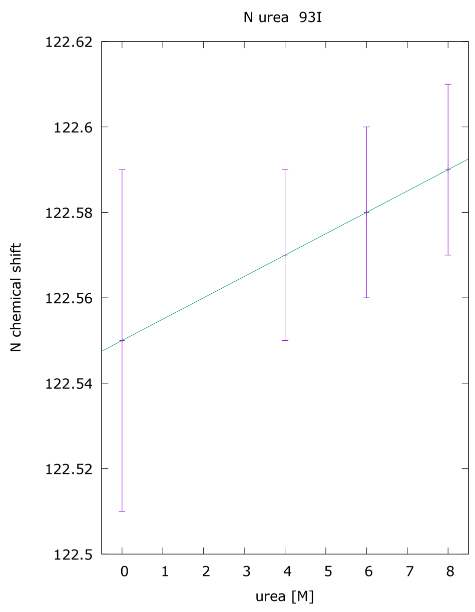
N urea 93I
N chemical shift
urea [M]

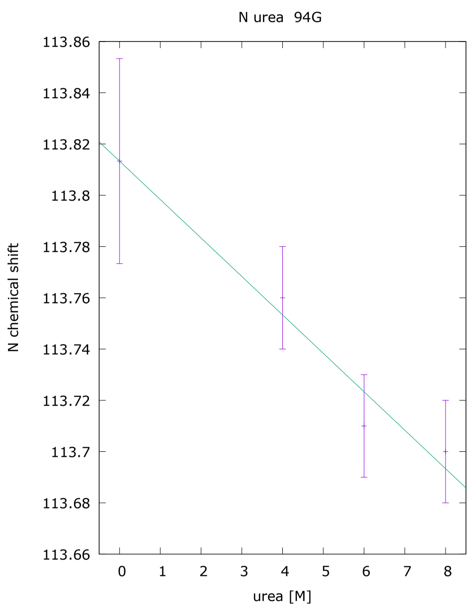
N urea 94G
N chemical shift
urea [M]

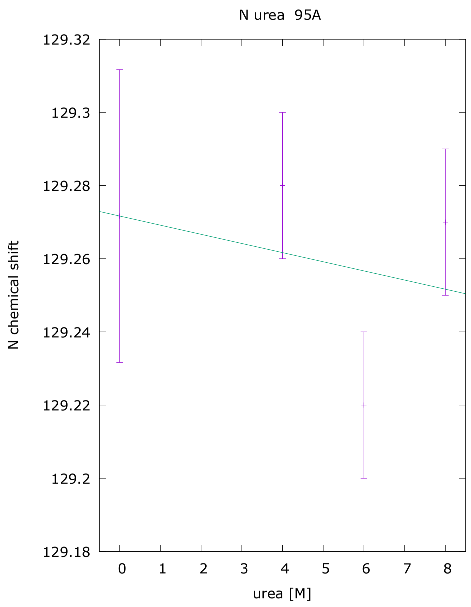
N urea 95A
N chemical shift
urea [M]

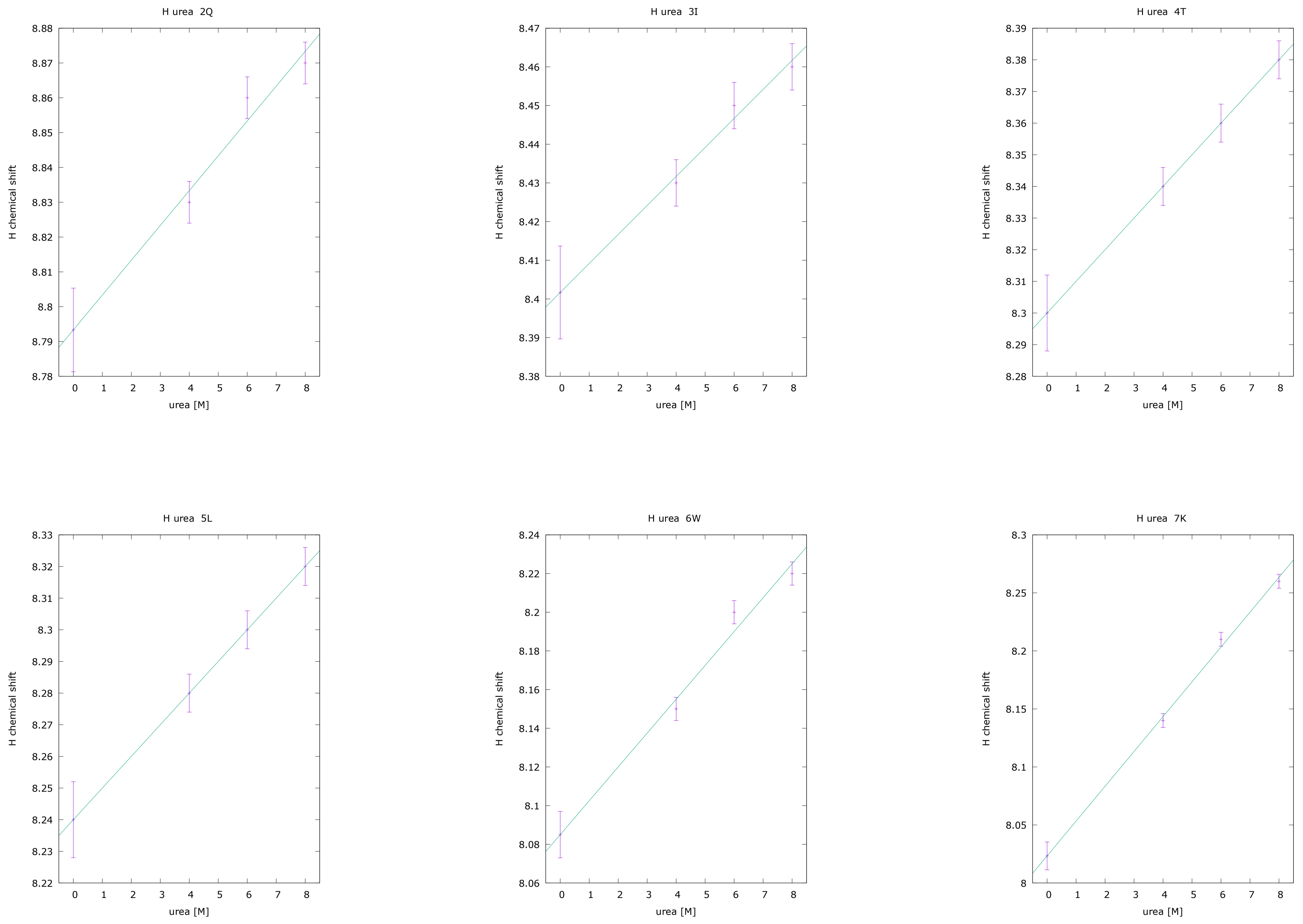
H urea 2Q
H urea 3I
H urea 4T
H urea 5L
H urea 6W
H urea 7K
H chemical shift
urea [M]

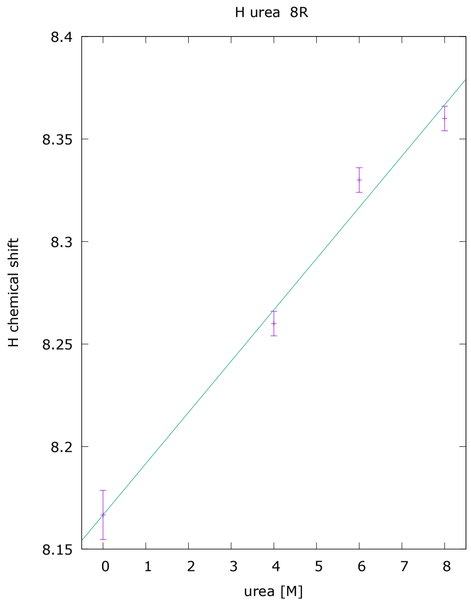
H urea 8R
H chemical shift
8.4
8.35
8.3
8.25
8.2
8.15
0 1 2 3 4 5 6 7 8
urea [M]

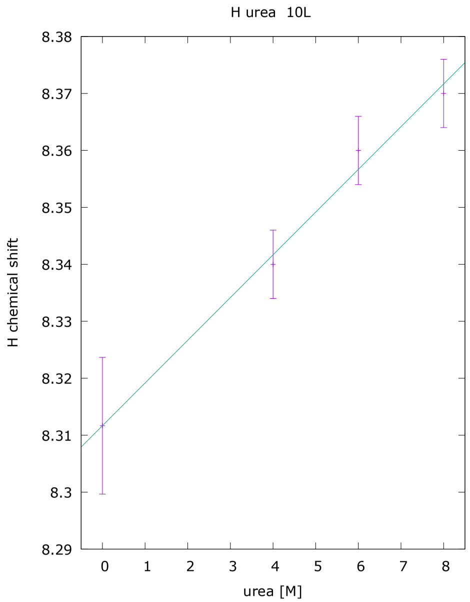
H urea 10L
H chemical shift
8.38
8.37
8.36
8.35
8.34
8.33
8.32
8.31
8.3
8.29
0 1 2 3 4 5 6 7 8
urea [M]

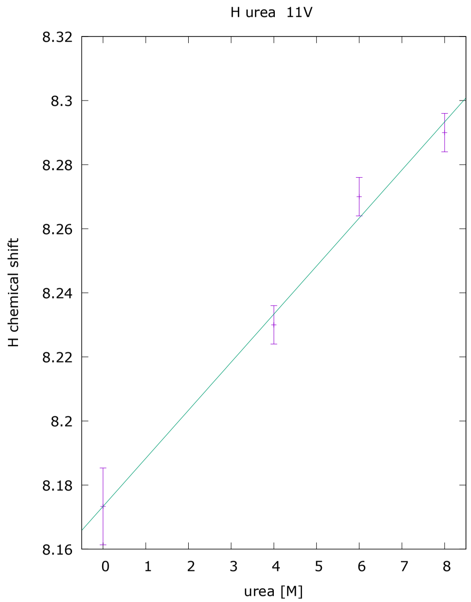
H urea 11V
H chemical shift
8.32
8.3
8.28
8.26
8.24
8.22
8.2
8.18
8.16
0 1 2 3 4 5 6 7 8
urea [M]

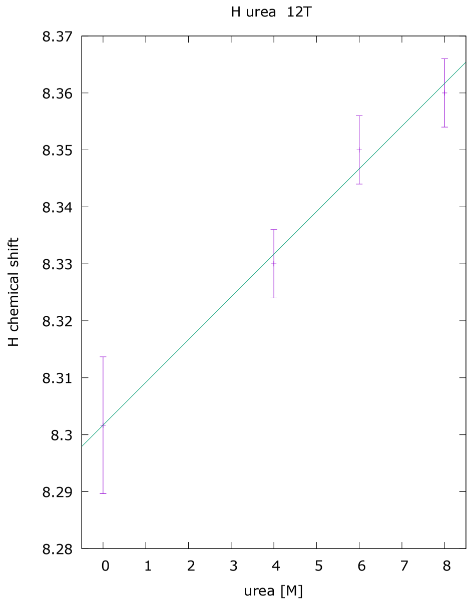
H urea 12T
H chemical shift
8.37
8.36
8.35
8.34
8.33
8.32
8.31
8.3
8.29
8.28
0 1 2 3 4 5 6 7 8
urea [M]

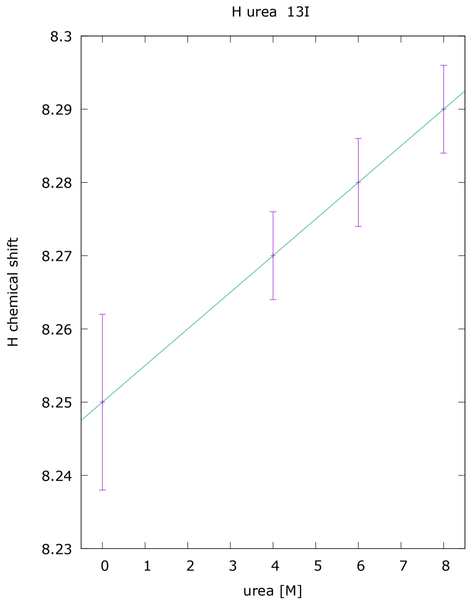
H urea 13I
H chemical shift
8.3
8.29
8.28
8.27
8.26
8.25
8.24
8.23
0 1 2 3 4 5 6 7 8
urea [M]

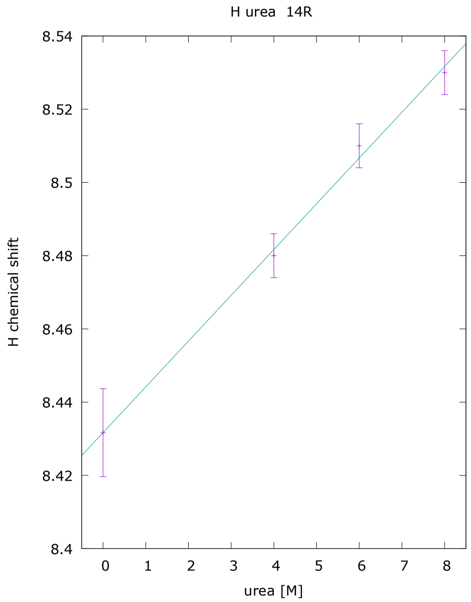
H urea 14R
H chemical shift
8.54
8.52
8.5
8.48
8.46
8.44
8.42
8.4
0 1 2 3 4 5 6 7 8
urea [M]

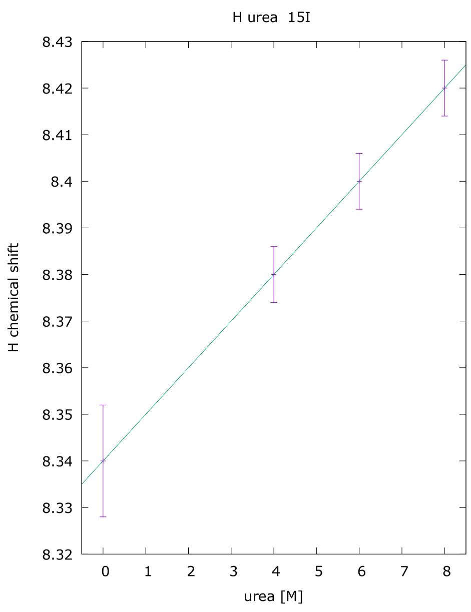
H urea 15I
H chemical shift
urea [M]
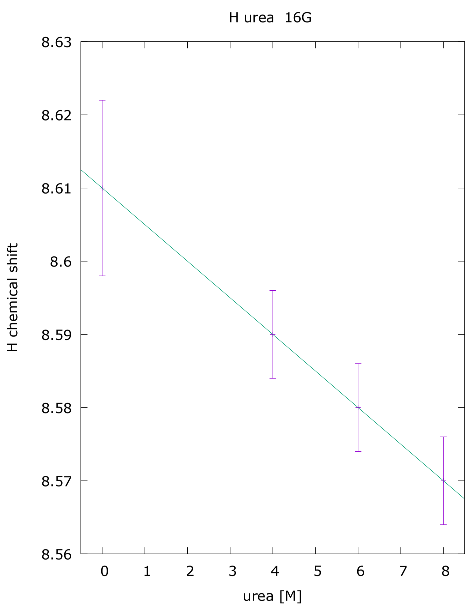
H urea 16G
H chemical shift
urea [M]
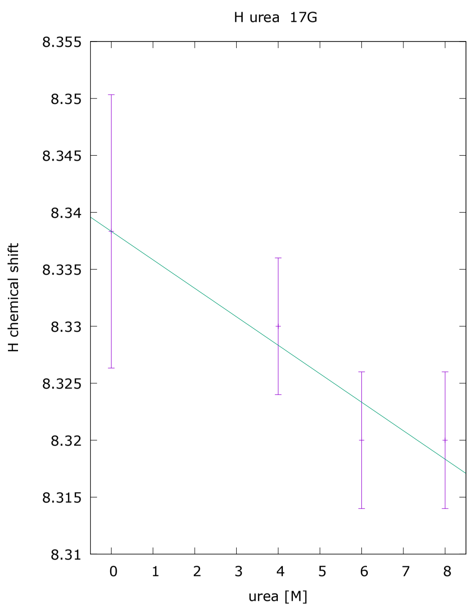
H urea 17G
H chemical shift
urea [M]
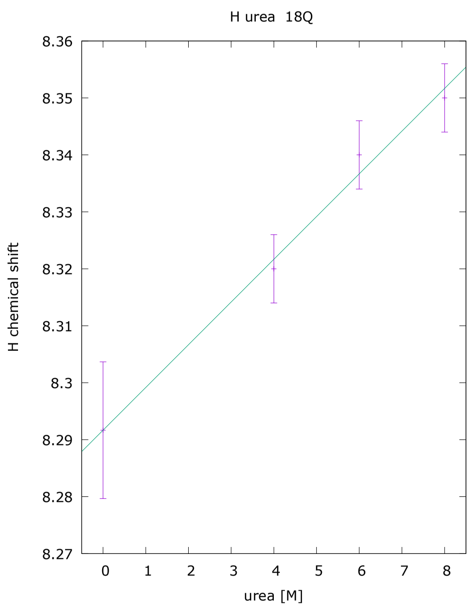
H urea 18Q
H chemical shift
urea [M]
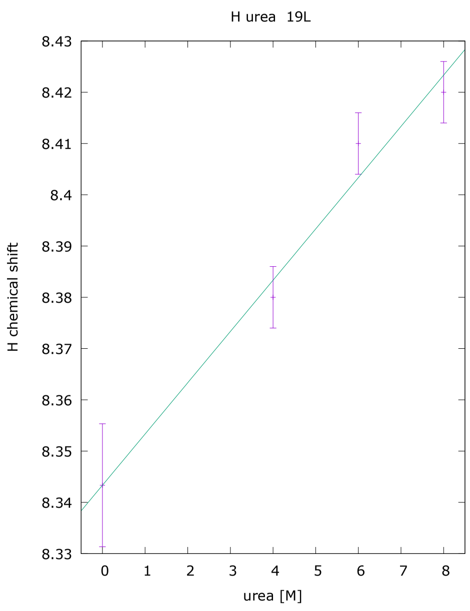
H urea 19L
H chemical shift
urea [M]
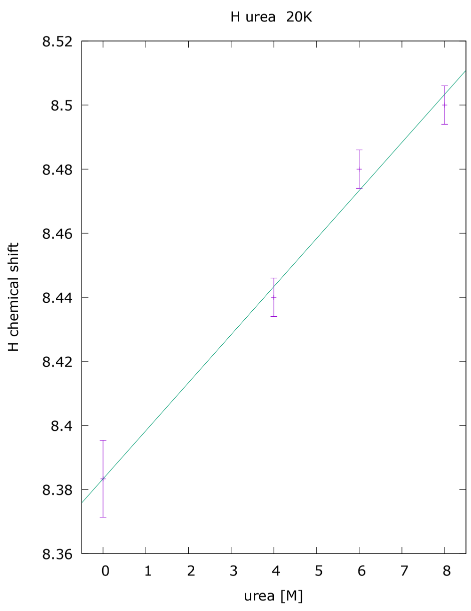
H urea 20K
H chemical shift
urea [M]

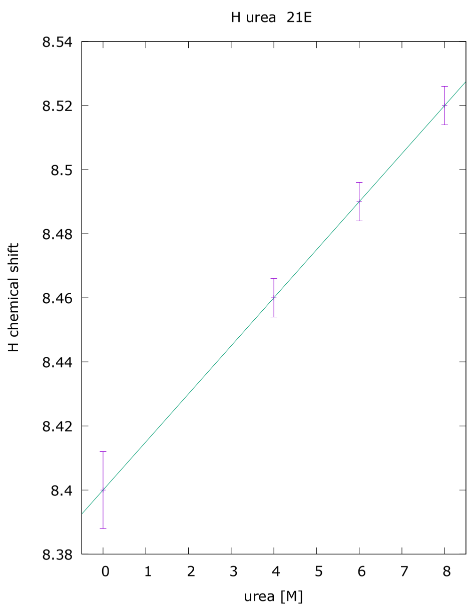
H urea 21E
H chemical shift
urea [M]

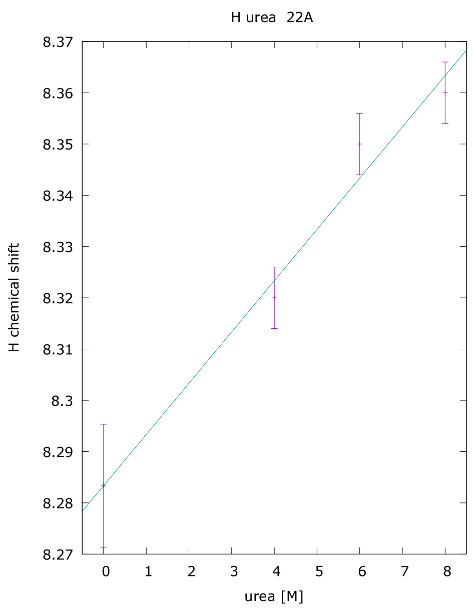
H urea 22A
H chemical shift
urea [M]

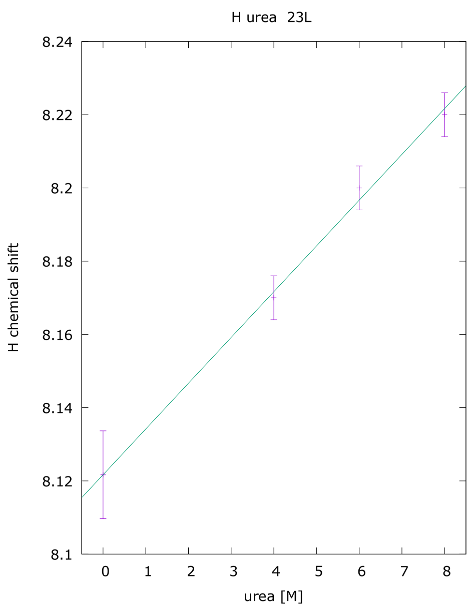
H urea 23L
H chemical shift
urea [M]

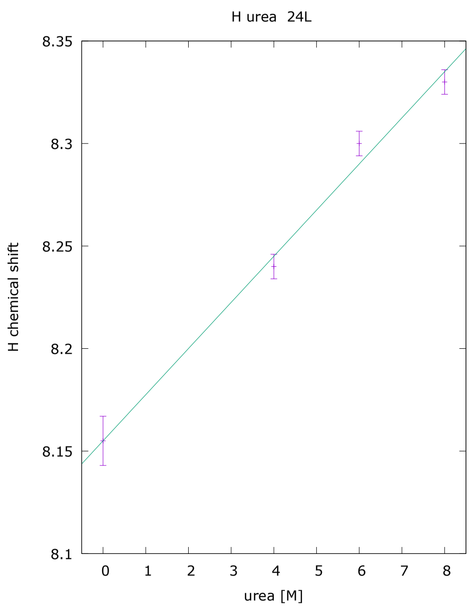
H urea 24L
H chemical shift
urea [M]

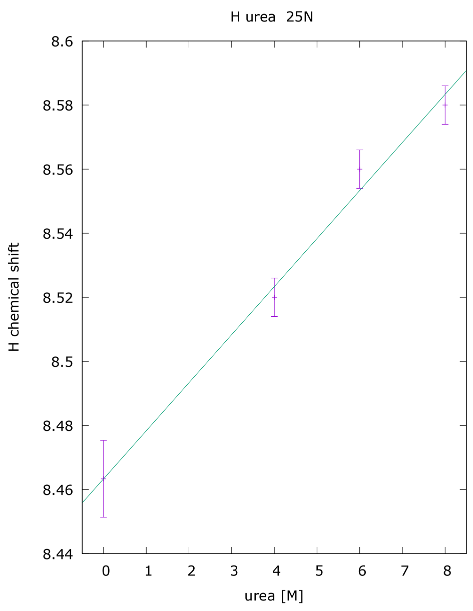
H urea 25N
H chemical shift
urea [M]

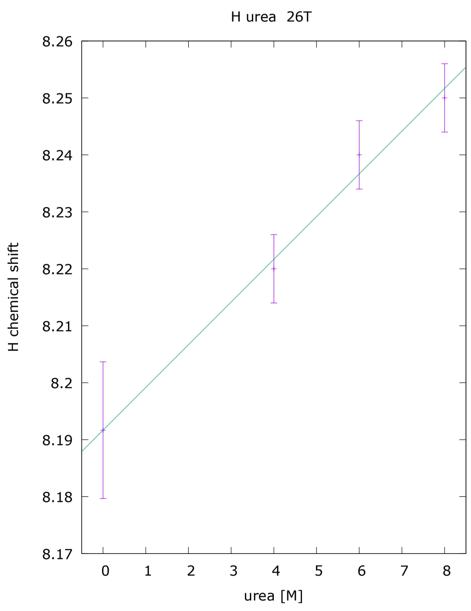
H urea 26T
H chemical shift
urea [M]

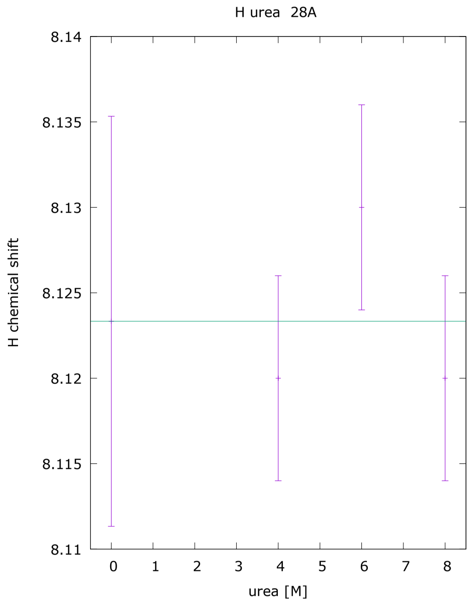
H urea 28A
H chemical shift
urea [M]

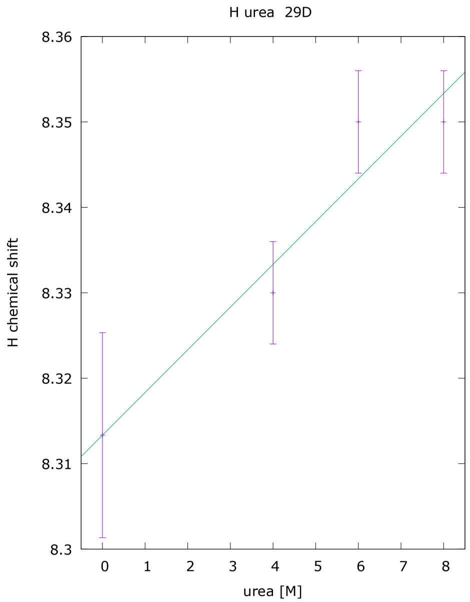
H urea 29D
H chemical shift
urea [M]

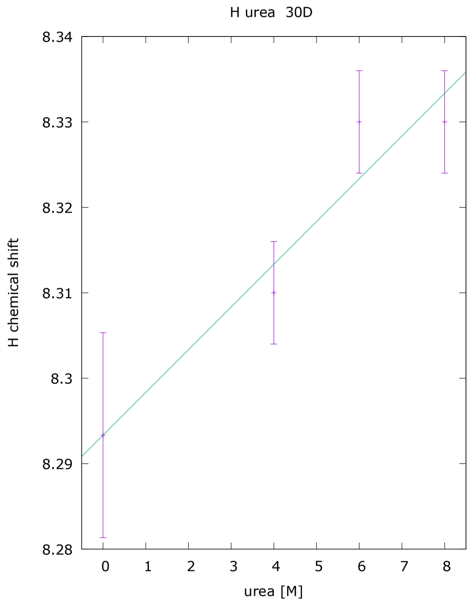
H urea 30D
H chemical shift
urea [M]

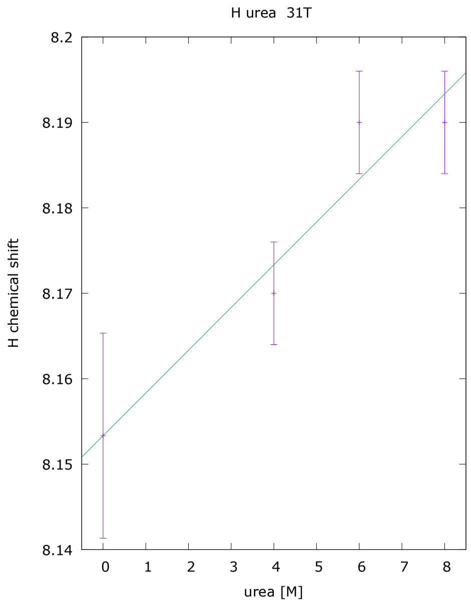
H urea 31T
H chemical shift
urea [M]

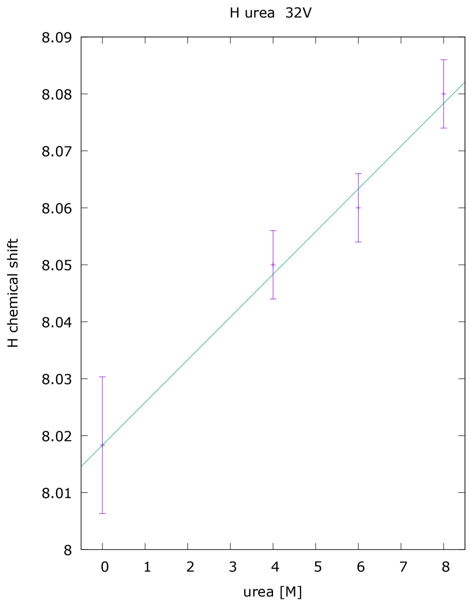
H urea 32V
H chemical shift
urea [M]

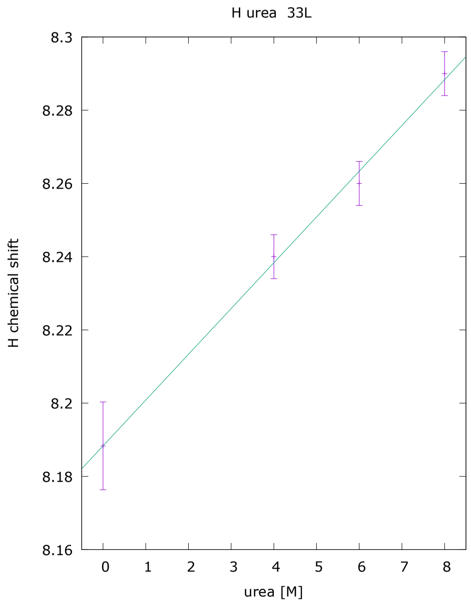
H urea 33L
H chemical shift
urea [M]

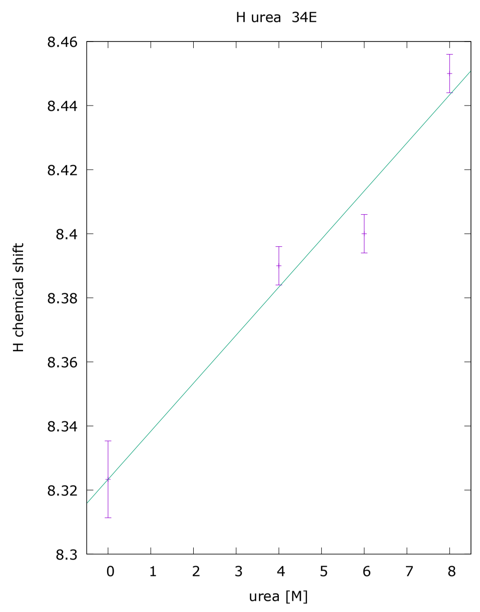
H urea 34E
H chemical shift
urea [M]

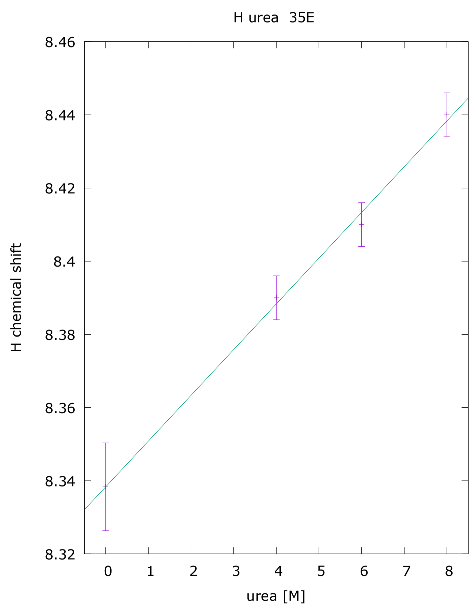
H urea 35E
H chemical shift
urea [M]

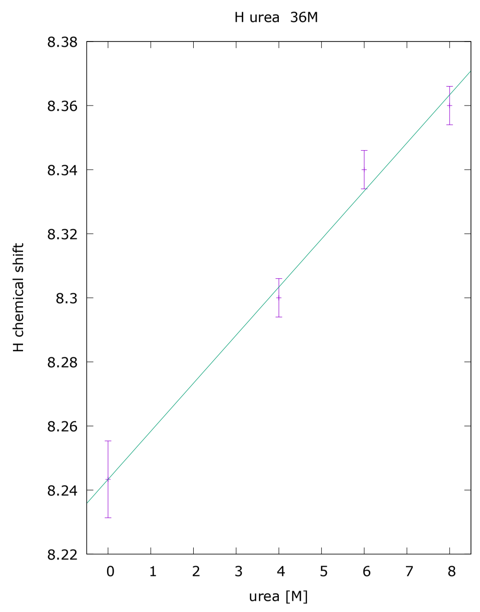
H urea 36M
H chemical shift
urea [M]

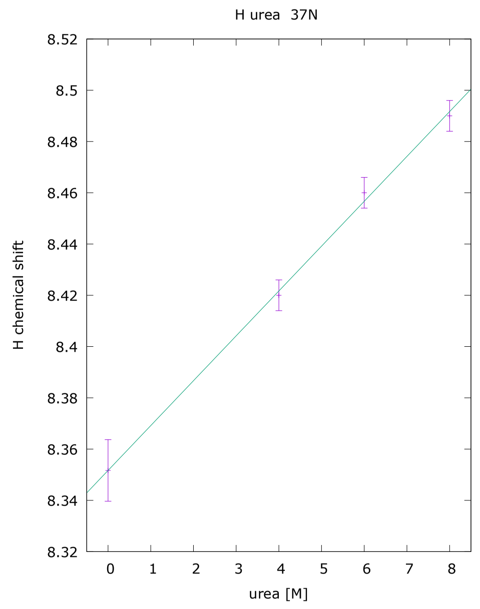
H urea 37N
H chemical shift
urea [M]

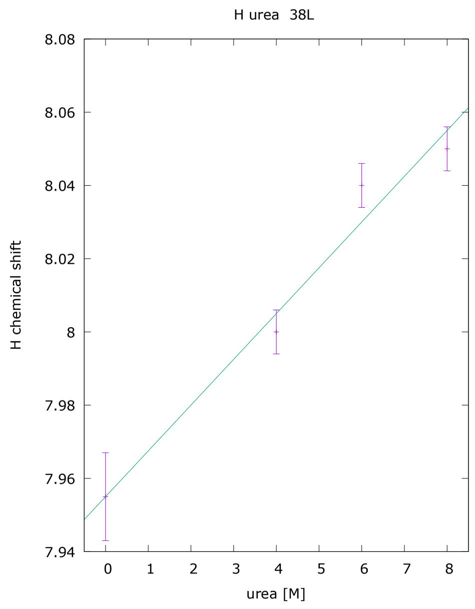
H urea 38L
H chemical shift
urea [M]

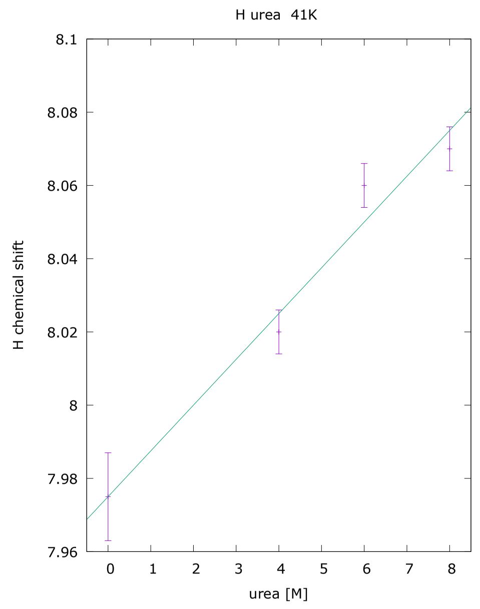
H urea 41K
H chemical shift
urea [M]

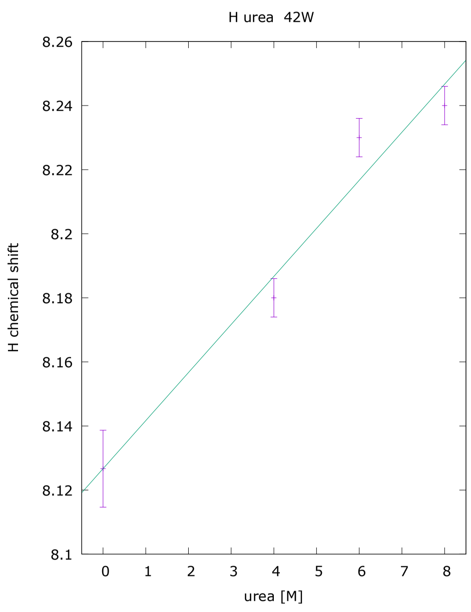
H urea 42W
H chemical shift
urea [M]

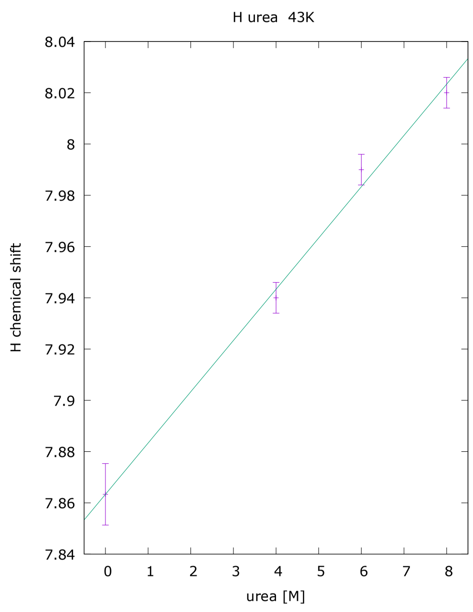
H urea 43K
H chemical shift
urea [M]

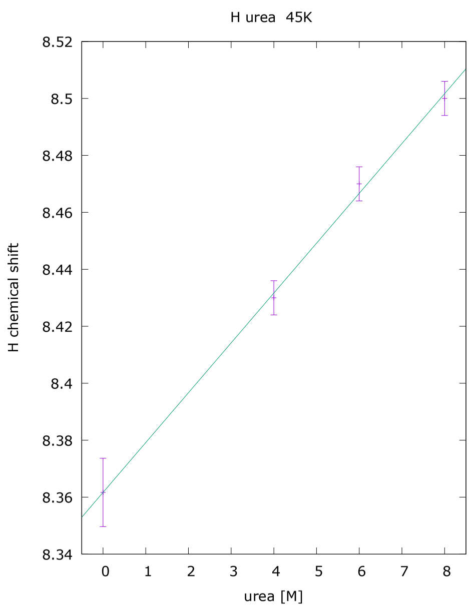
H urea 45K
H chemical shift
urea [M]

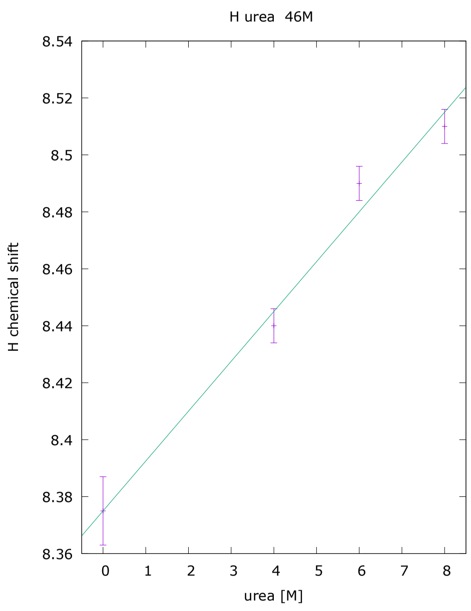
H urea 46M
H chemical shift
urea [M]

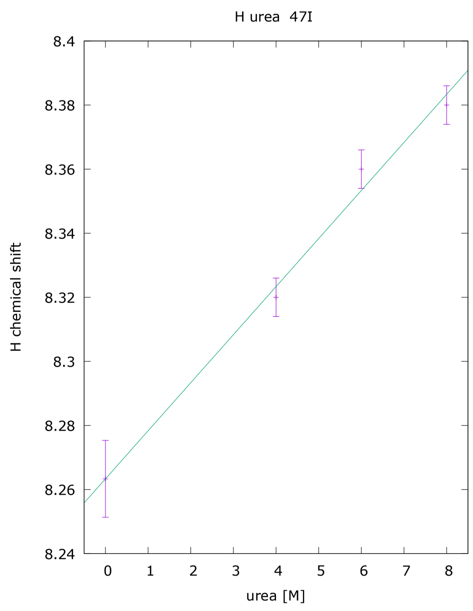
H urea 47I
H chemical shift
urea [M]

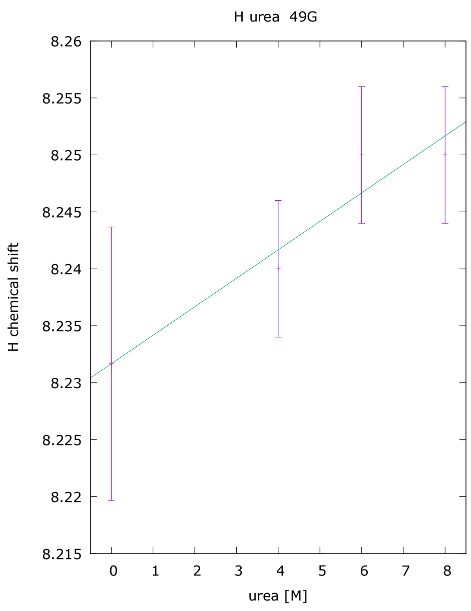
H urea 49G
H chemical shift
urea [M]

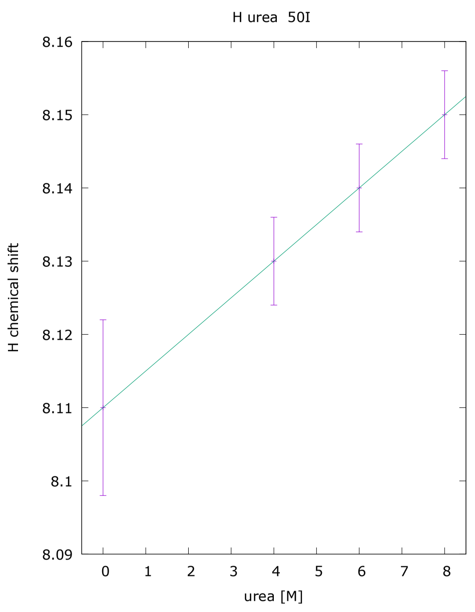
H urea 50I
H chemical shift
urea [M]
8.09
8.1
8.11
8.12
8.13
8.14
8.15
8.16
0 1 2 3 4 5 6 7 8

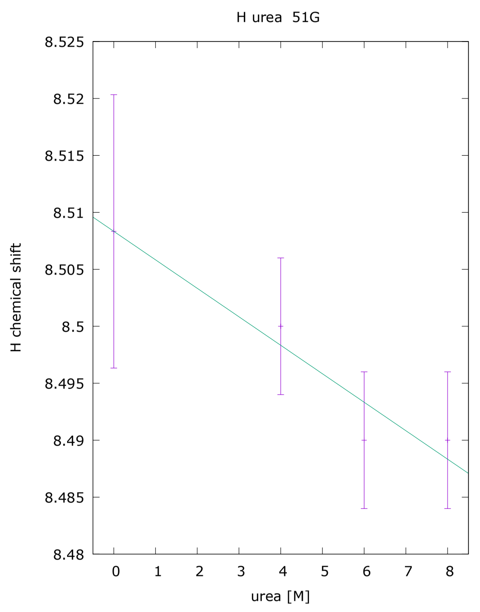
H urea 51G
H chemical shift
urea [M]
8.48
8.485
8.49
8.495
8.5
8.505
8.51
8.515
8.52
8.525
0 1 2 3 4 5 6 7 8

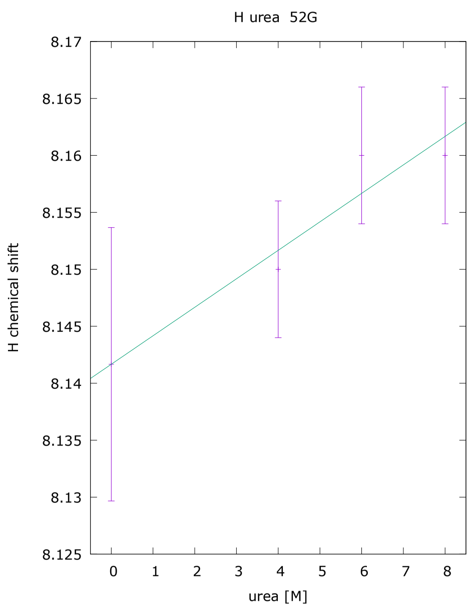
H urea 52G
H chemical shift
urea [M]
8.125
8.13
8.135
8.14
8.145
8.15
8.155
8.16
8.165
8.17
0 1 2 3 4 5 6 7 8

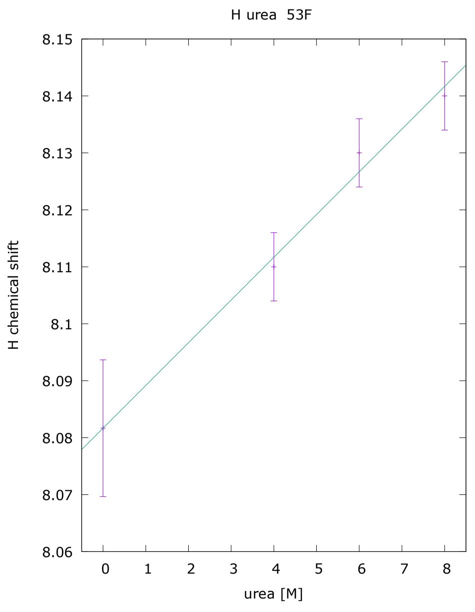
H urea 53F
H chemical shift
urea [M]
8.06
8.07
8.08
8.09
8.1
8.11
8.12
8.13
8.14
8.15
0 1 2 3 4 5 6 7 8

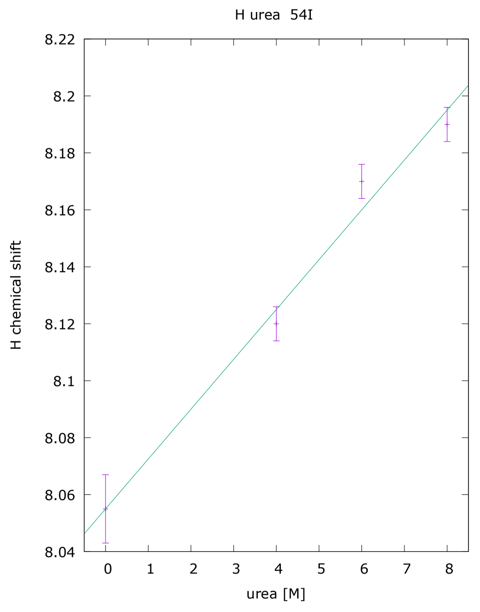
H urea 54I
H chemical shift
urea [M]
8.04
8.06
8.08
8.1
8.12
8.14
8.16
8.18
8.2
8.22
0 1 2 3 4 5 6 7 8

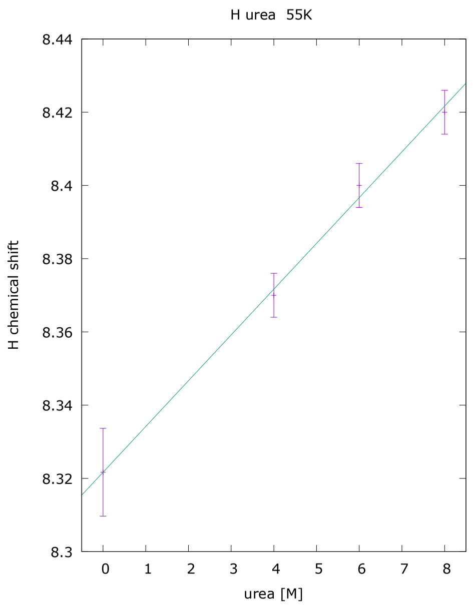
H urea 55K
H chemical shift
urea [M]
8.3
8.32
8.34
8.36
8.38
8.4
8.42
8.44
0 1 2 3 4 5 6 7 8

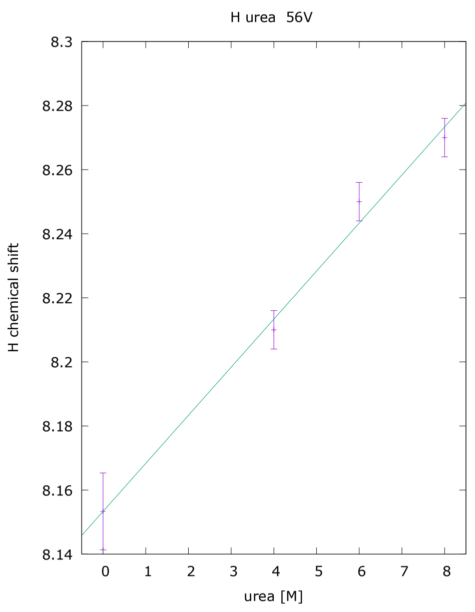
H urea 56V
H chemical shift
8.3
8.28
8.26
8.24
8.22
8.2
8.18
8.16
8.14
0
1
2
3
4
5
6
7
8
urea [M]

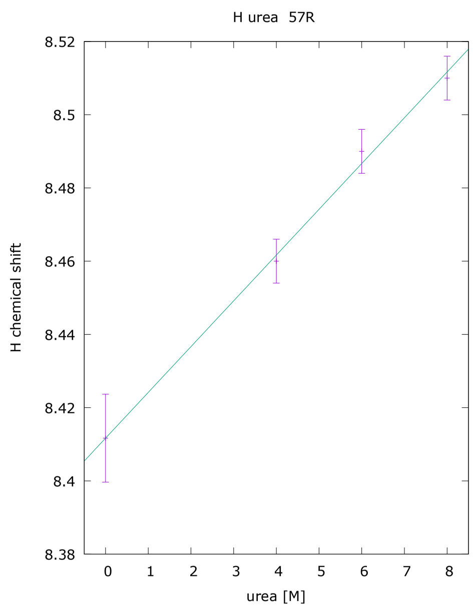
H urea 57R
H chemical shift
8.52
8.5
8.48
8.46
8.44
8.42
8.4
8.38
0
1
2
3
4
5
6
7
8
urea [M]

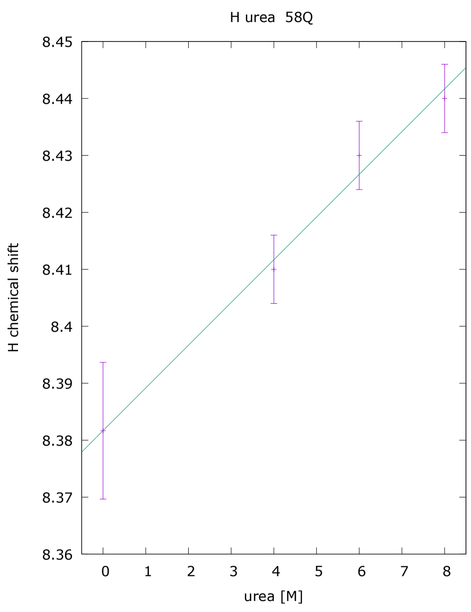
H urea 58Q
H chemical shift
8.45
8.44
8.43
8.42
8.41
8.4
8.39
8.38
8.37
8.36
0
1
2
3
4
5
6
7
8
urea [M]

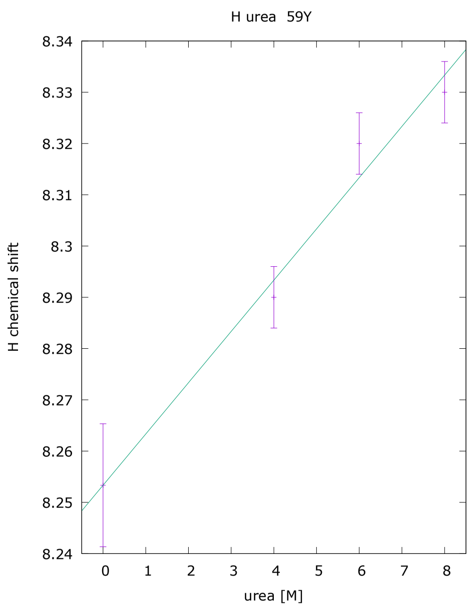
H urea 59Y
H chemical shift
8.34
8.33
8.32
8.31
8.3
8.29
8.28
8.27
8.26
8.25
8.24
0
1
2
3
4
5
6
7
8
urea [M]

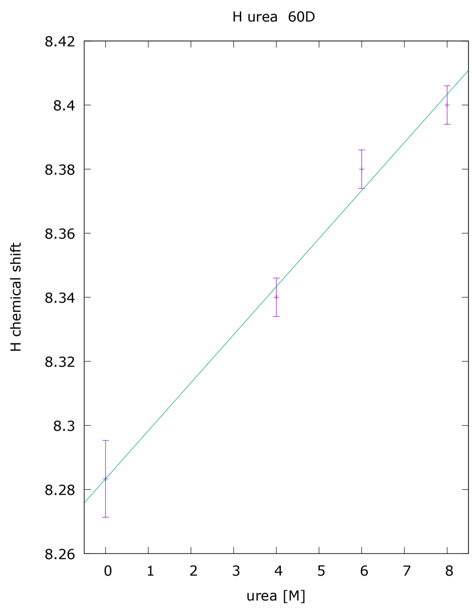
H urea 60D
H chemical shift
8.42
8.4
8.38
8.36
8.34
8.32
8.3
8.28
8.26
0
1
2
3
4
5
6
7
8
urea [M]

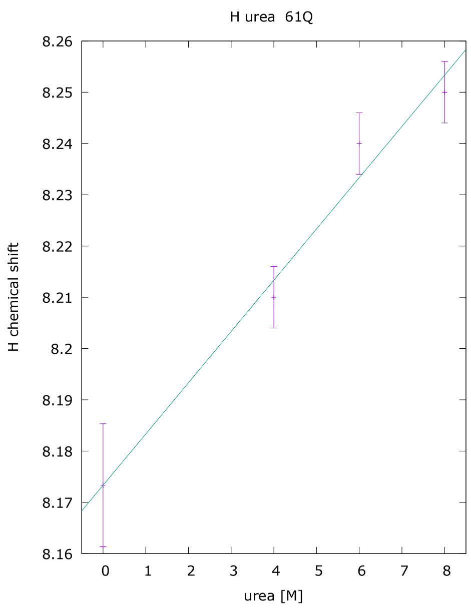
H urea 61Q
H chemical shift
8.26
8.25
8.24
8.23
8.22
8.21
8.2
8.19
8.18
8.17
8.16
0
1
2
3
4
5
6
7
8
urea [M]

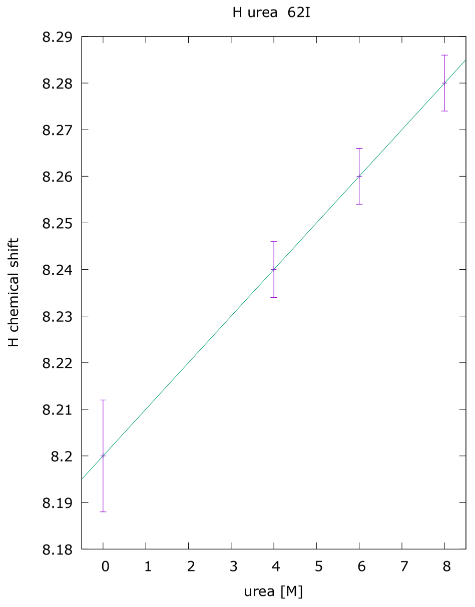
H urea 62I
H chemical shift
urea [M]

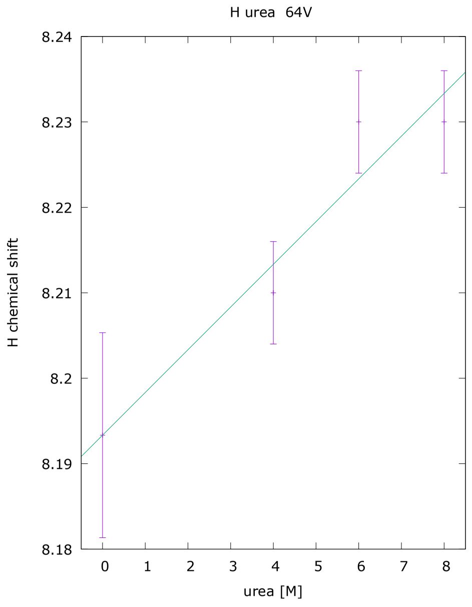
H urea 64V
H chemical shift
urea [M]

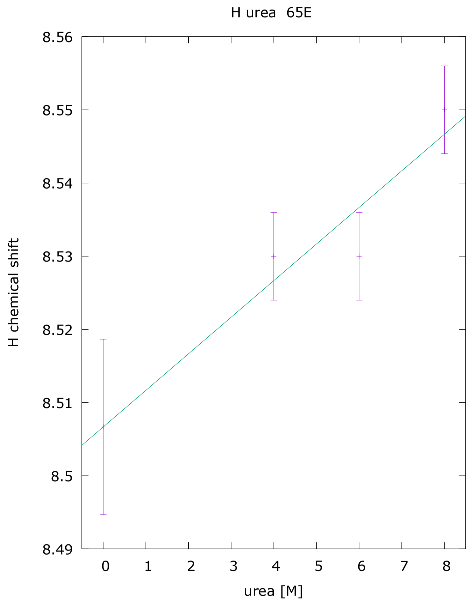
H urea 65E
H chemical shift
urea [M]

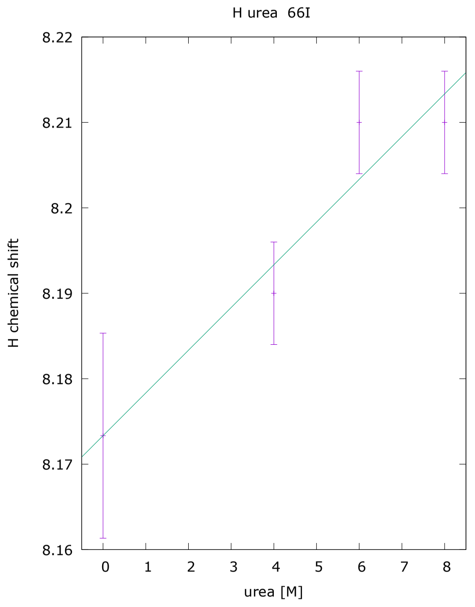
H urea 66I
H chemical shift
urea [M]

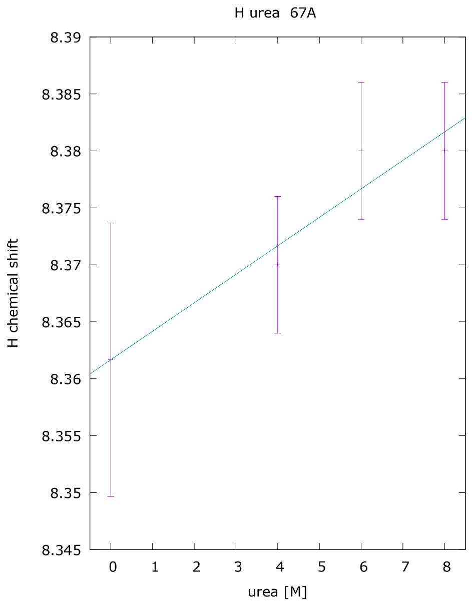
H urea 67A
H chemical shift
urea [M]

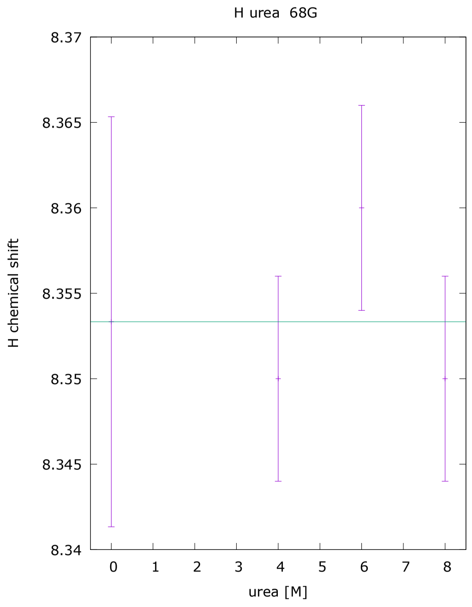
H urea 68G
H chemical shift
urea [M]

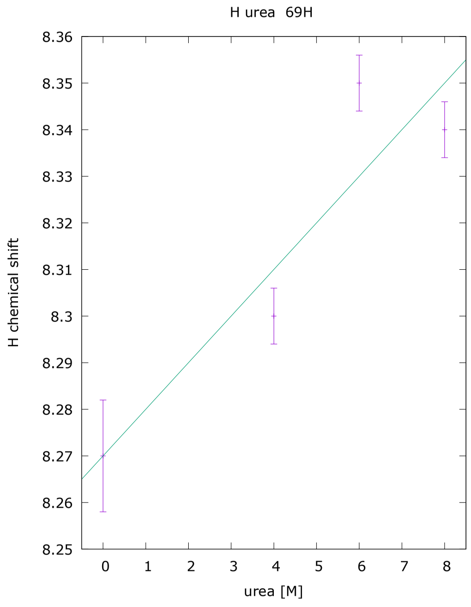
H urea 69H
H chemical shift
urea [M]

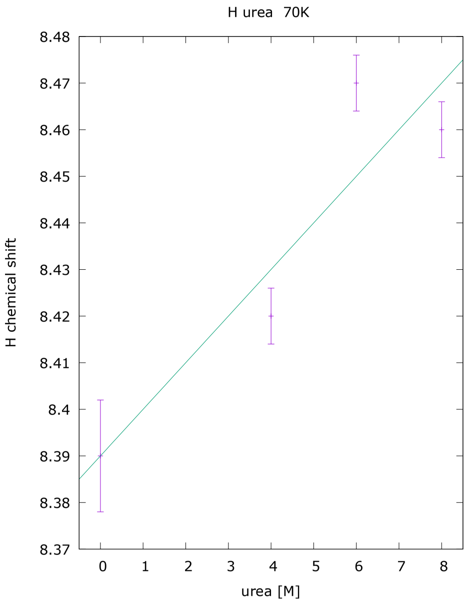
H urea 70K
H chemical shift
urea [M]

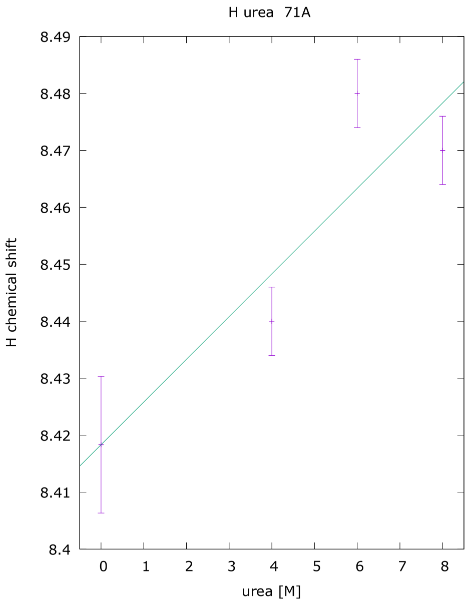
H urea 71A
H chemical shift
urea [M]

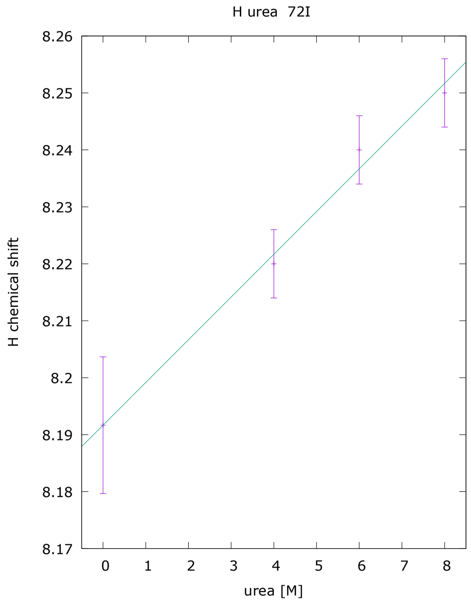
H urea 72I
H chemical shift
urea [M]

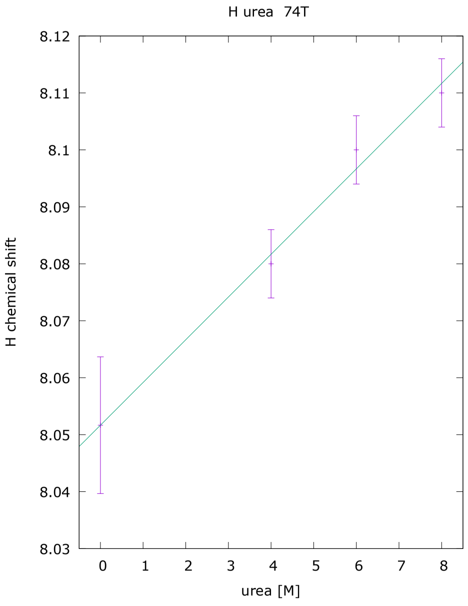
H urea 74T
H chemical shift
urea [M]

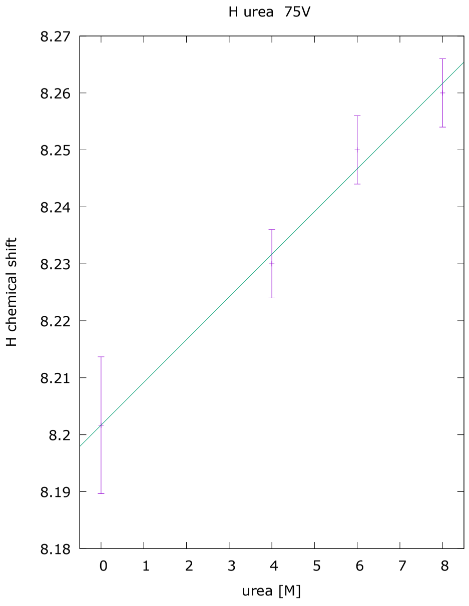
H urea 75V
H chemical shift
urea [M]

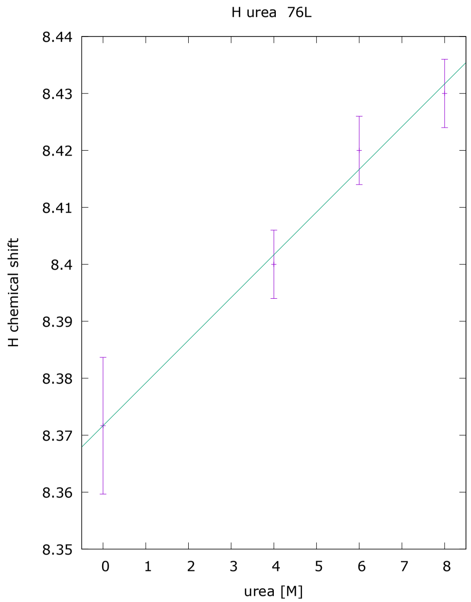
H urea 76L
H chemical shift
8.44
8.43
8.42
8.41
8.4
8.39
8.38
8.37
8.36
8.35
0 1 2 3 4 5 6 7 8
urea [M]

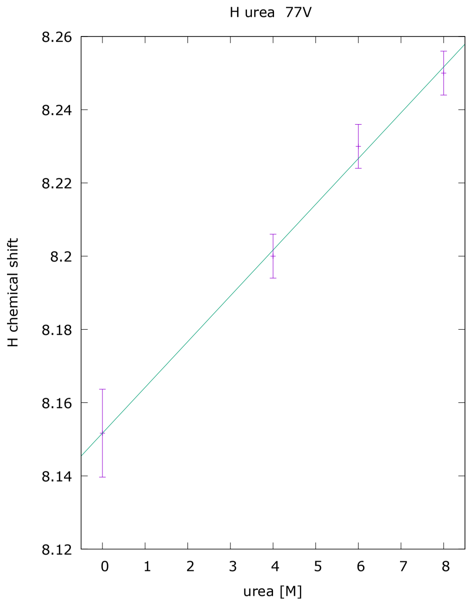
H urea 77V
H chemical shift
8.26
8.24
8.22
8.2
8.18
8.16
8.14
8.12
0 1 2 3 4 5 6 7 8
urea [M]

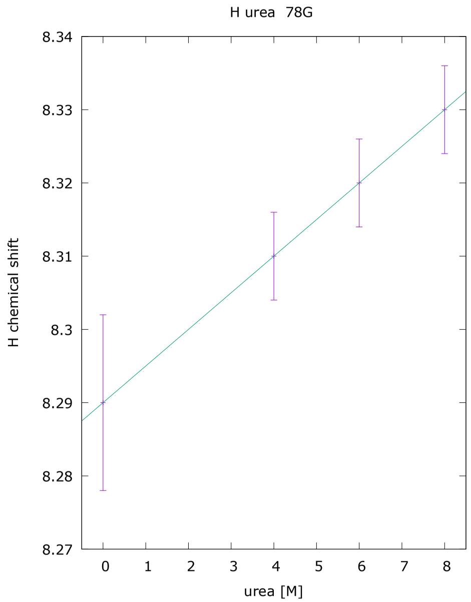
H urea 78G
H chemical shift
8.34
8.33
8.32
8.31
8.3
8.29
8.28
8.27
0 1 2 3 4 5 6 7 8
urea [M]

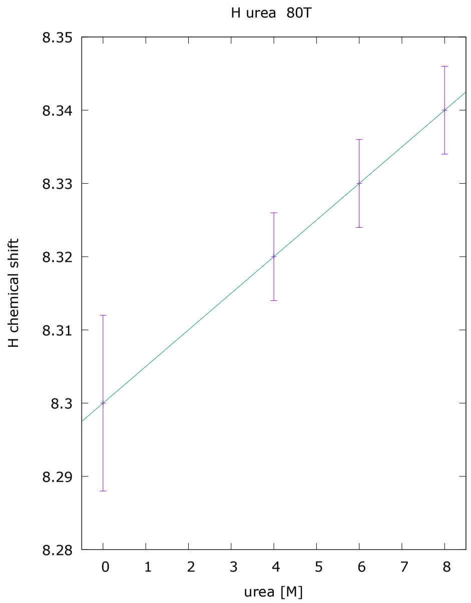
H urea 80T
H chemical shift
8.35
8.34
8.33
8.32
8.31
8.3
8.29
8.28
0 1 2 3 4 5 6 7 8
urea [M]

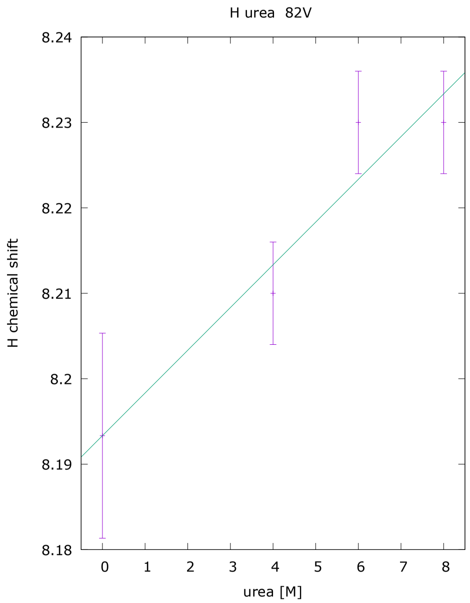
H urea 82V
H chemical shift
8.24
8.23
8.22
8.21
8.2
8.19
8.18
0 1 2 3 4 5 6 7 8
urea [M]

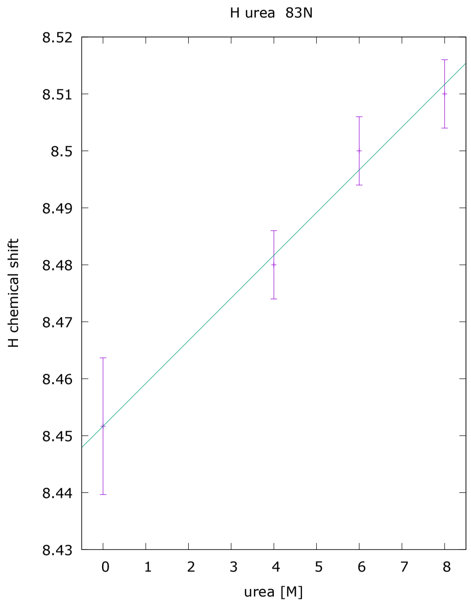
H urea 83N
H chemical shift
8.52
8.51
8.5
8.49
8.48
8.47
8.46
8.45
8.44
8.43
0 1 2 3 4 5 6 7 8
urea [M]

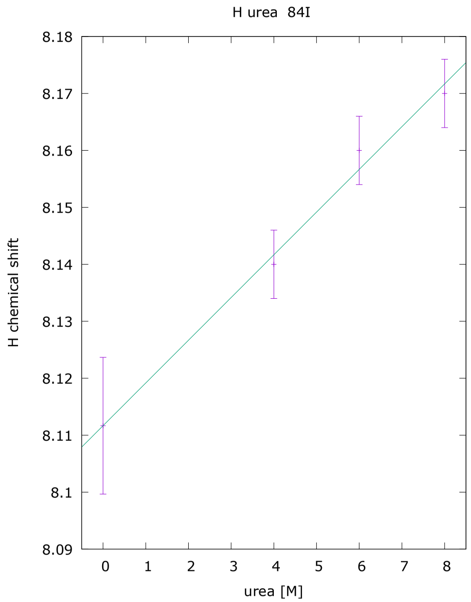
H urea 84I
H chemical shift
urea [M]

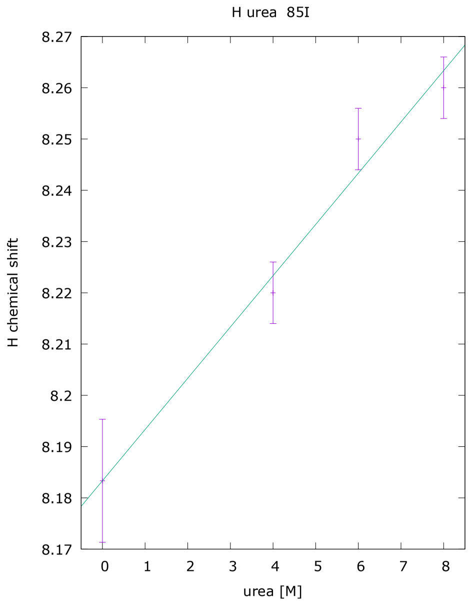
H urea 85I
H chemical shift
urea [M]

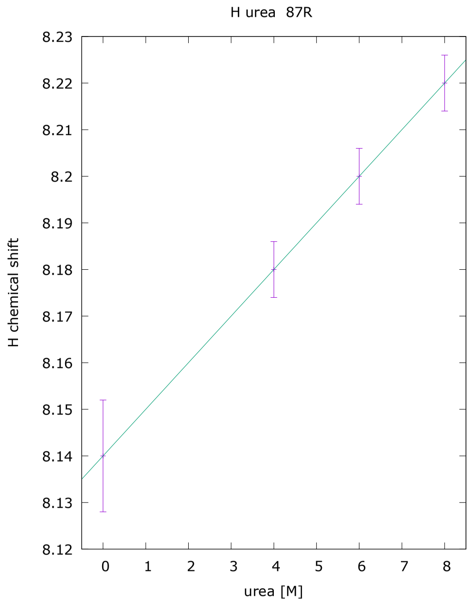
H urea 87R
H chemical shift
urea [M]

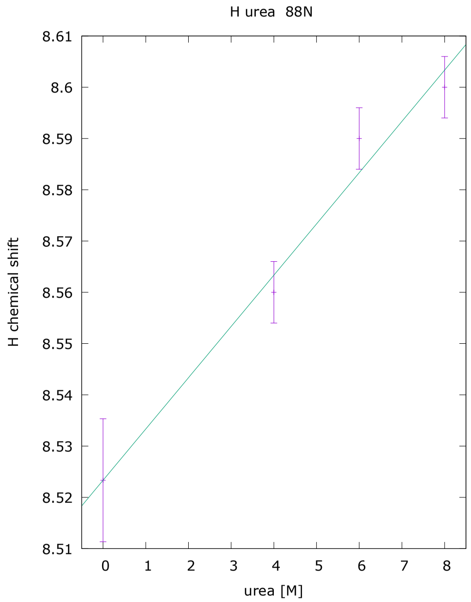
H urea 88N
H chemical shift
urea [M]

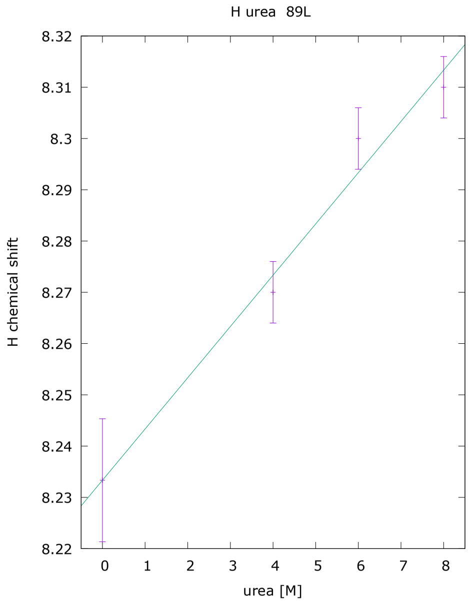
H urea 89L
H chemical shift
urea [M]

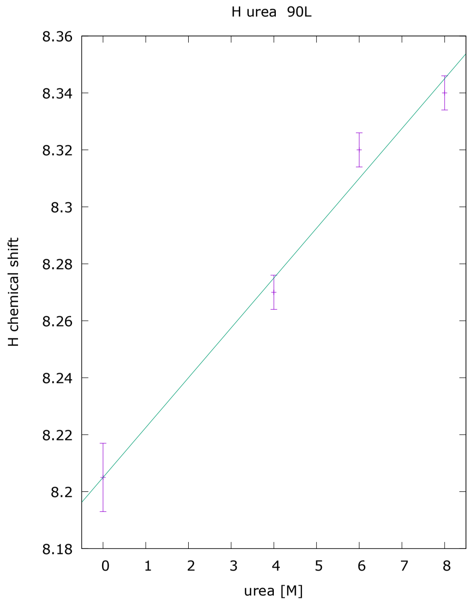
H urea 90L
H chemical shift
urea [M]

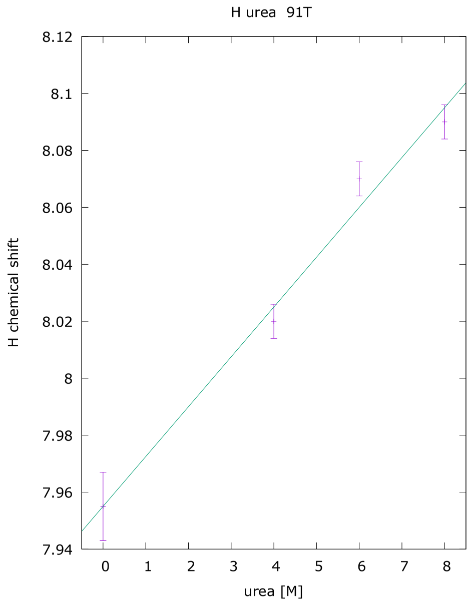
H urea 91T
H chemical shift
urea [M]

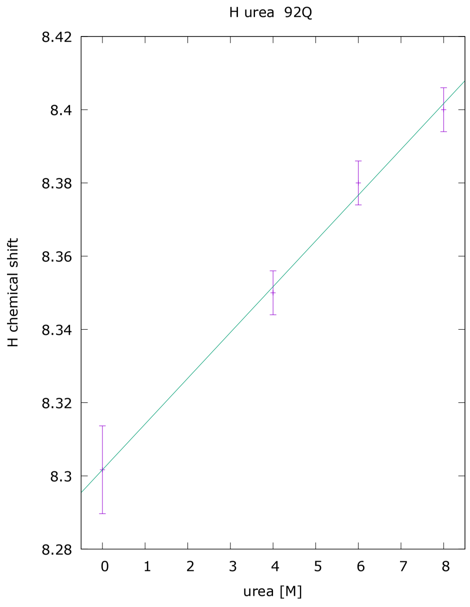
H urea 92Q
H chemical shift
urea [M]

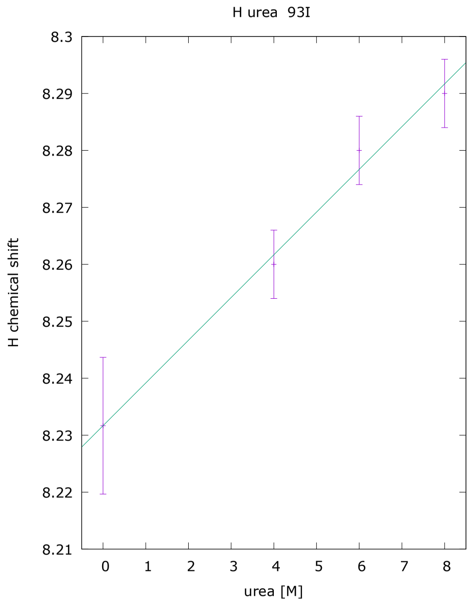
H urea 93I
H chemical shift
urea [M]

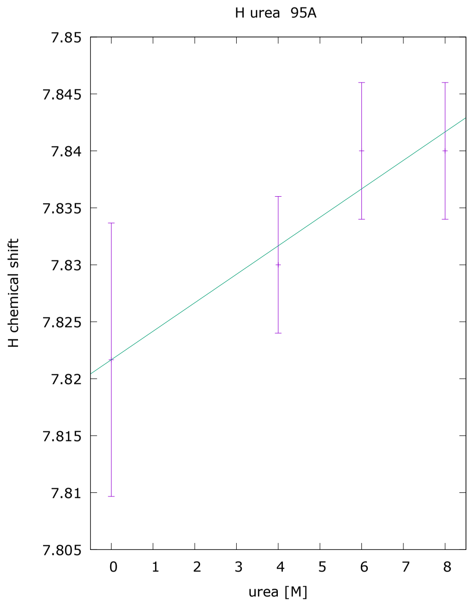
H urea 95A
H chemical shift
urea [M]

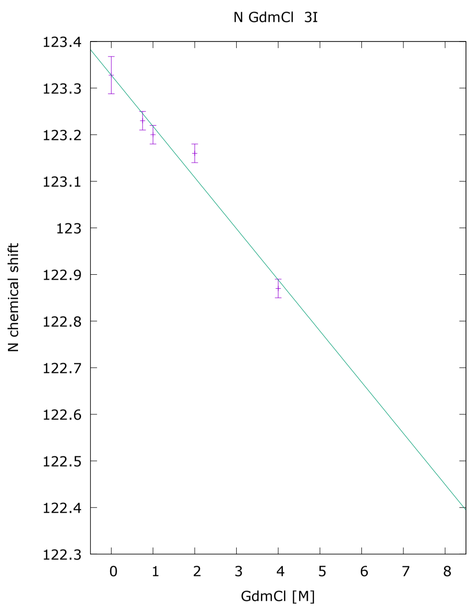
N GdmCl 3I
N chemical shift
GdmCl [M]
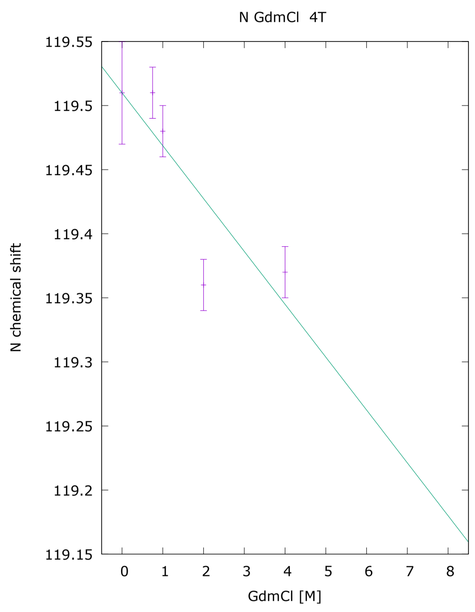
N GdmCl 4T
N chemical shift
GdmCl [M]
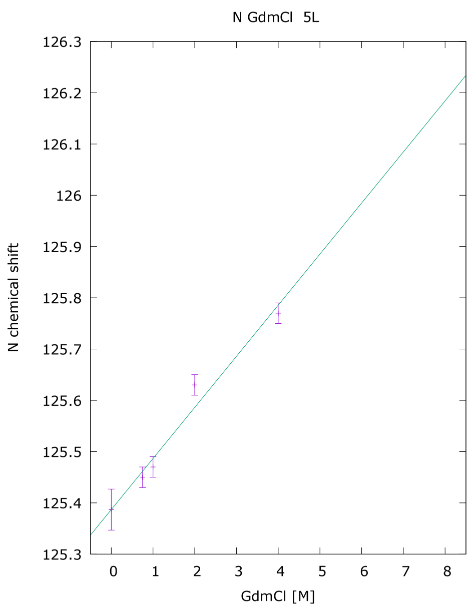
N GdmCl 5L
N chemical shift
GdmCl [M]
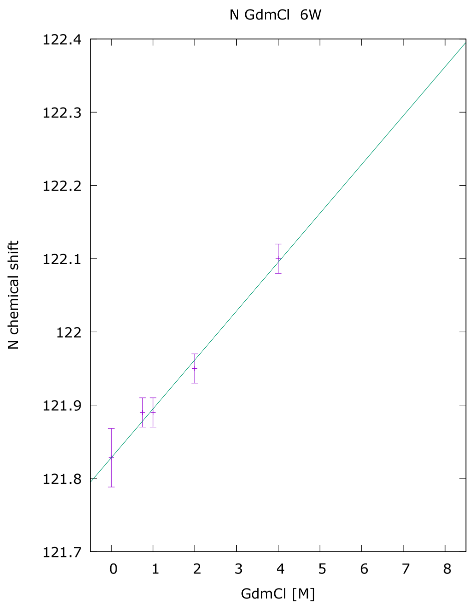
N GdmCl 6W
N chemical shift
GdmCl [M]
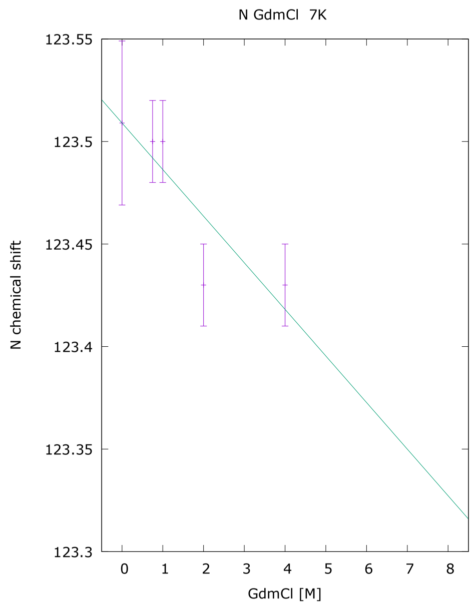
N GdmCl 7K
N chemical shift
GdmCl [M]
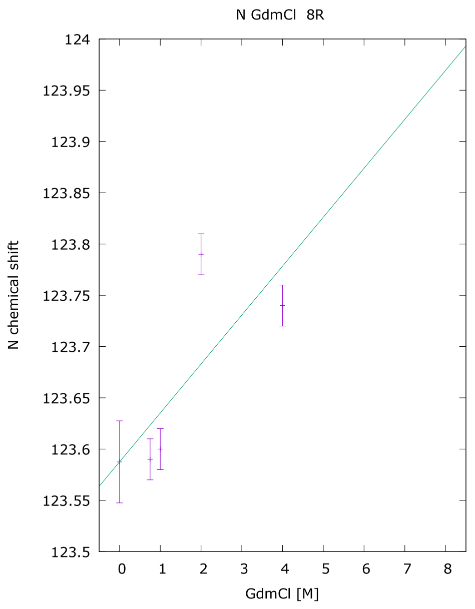
N GdmCl 8R
N chemical shift
GdmCl [M]

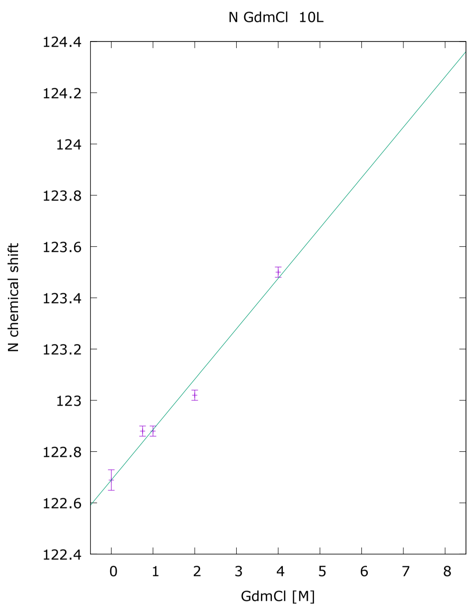
N GdmCl 10L
N chemical shift
GdmCl [M]

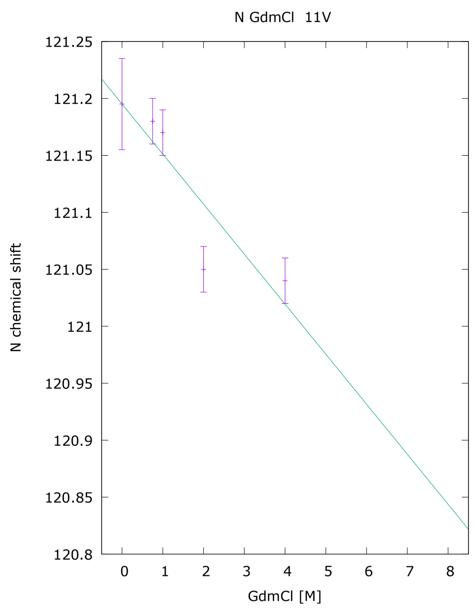
N GdmCl 11V
N chemical shift
GdmCl [M]

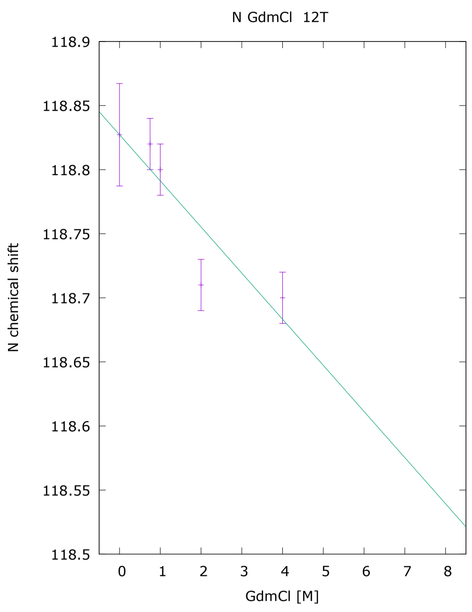
N GdmCl 12T
N chemical shift
GdmCl [M]

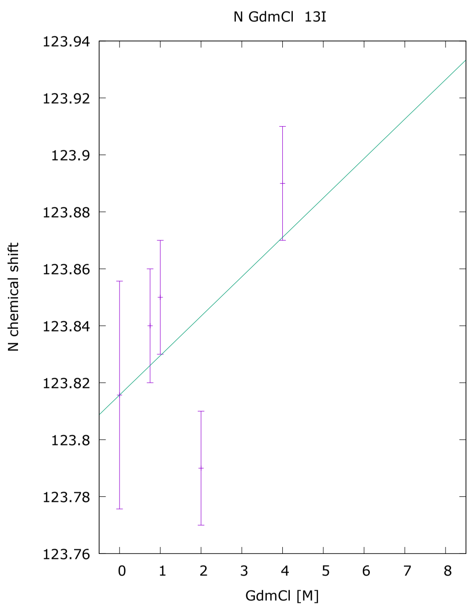
N GdmCl 13I
N chemical shift
GdmCl [M]

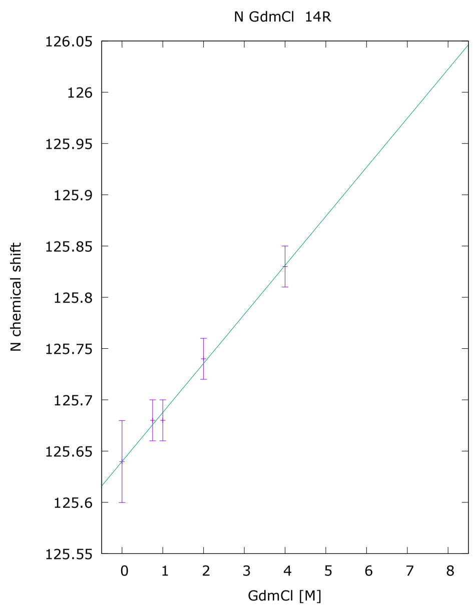
N GdmCl 14R
N chemical shift
GdmCl [M]

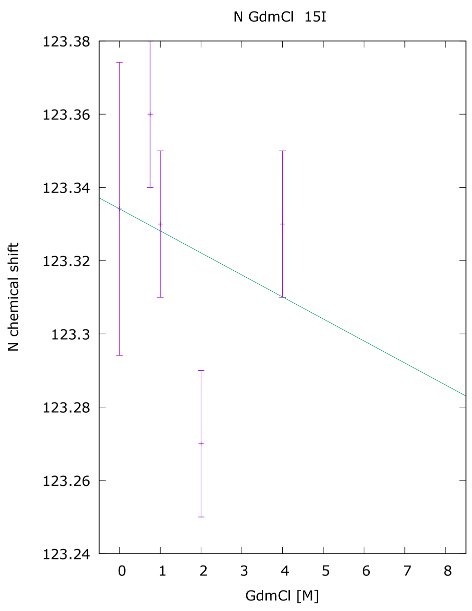
N GdmCl 15I
N chemical shift
GdmCl [M]

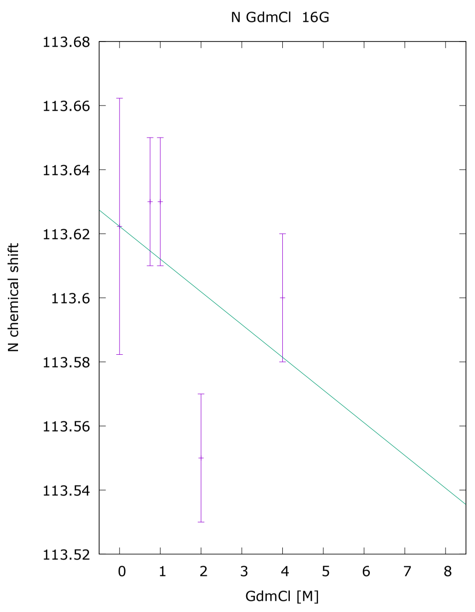
N GdmCl 16G
N chemical shift
GdmCl [M]

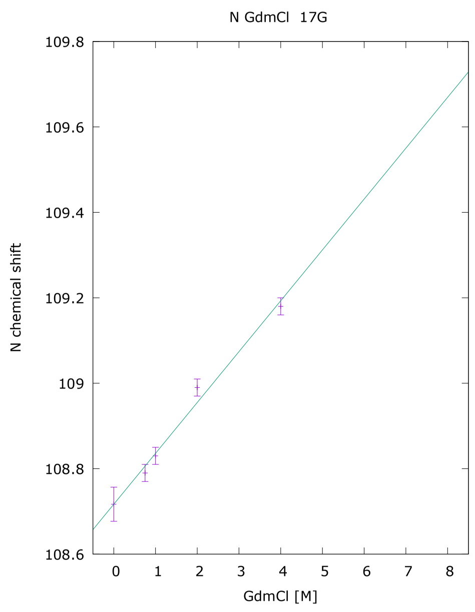
N GdmCl 17G
N chemical shift
GdmCl [M]

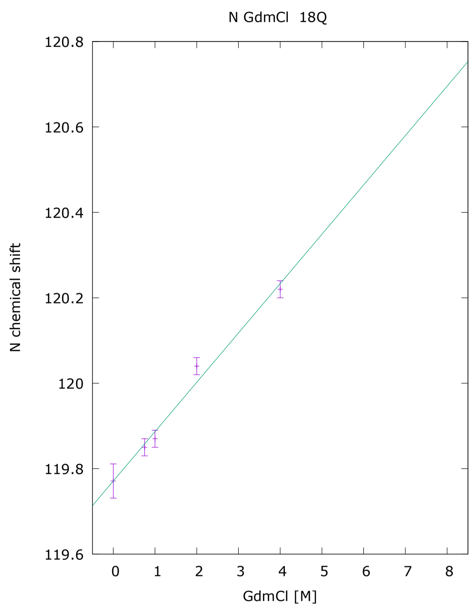
N GdmCl 18Q
N chemical shift
GdmCl [M]

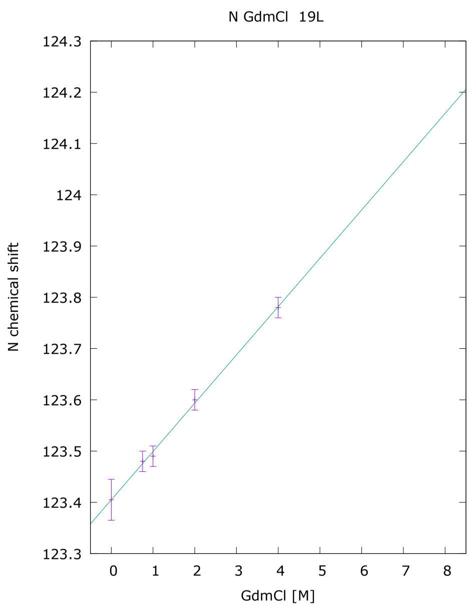
N GdmCl 19L
N chemical shift
GdmCl [M]

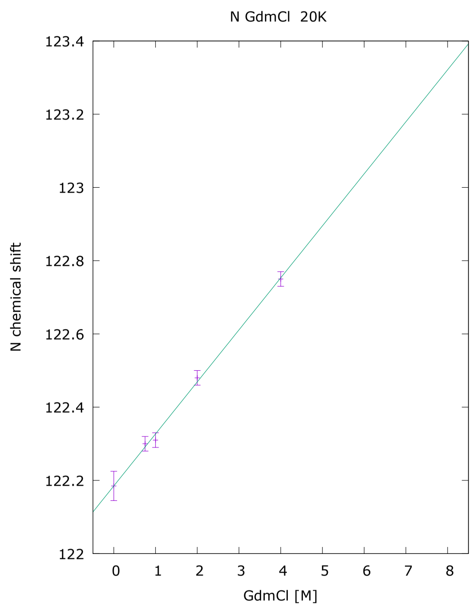
N GdmCl 20K
N chemical shift
GdmCl [M]

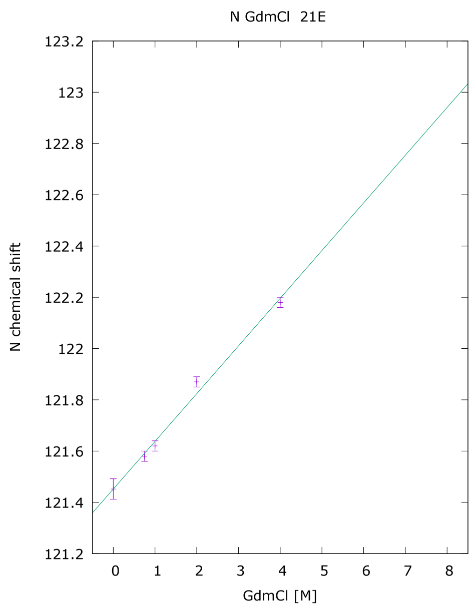
N GdmCl 21E
N chemical shift
GdmCl [M]

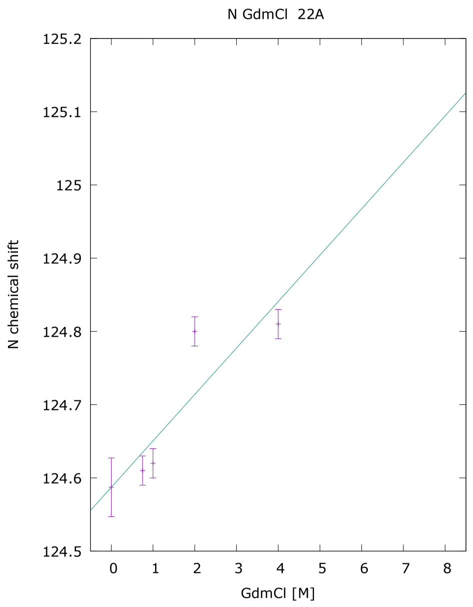
N GdmCl 22A
N chemical shift
GdmCl [M]

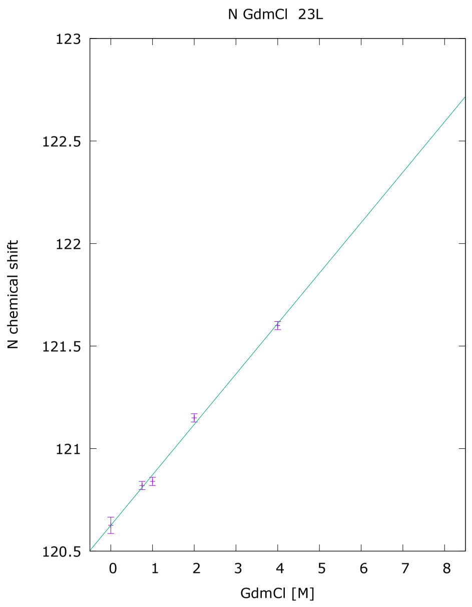
N GdmCl 23L
N chemical shift
GdmCl [M]

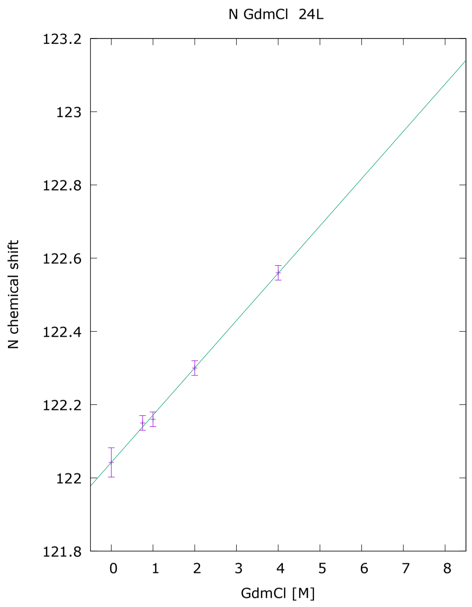
N GdmCl 24L
N chemical shift
GdmCl [M]

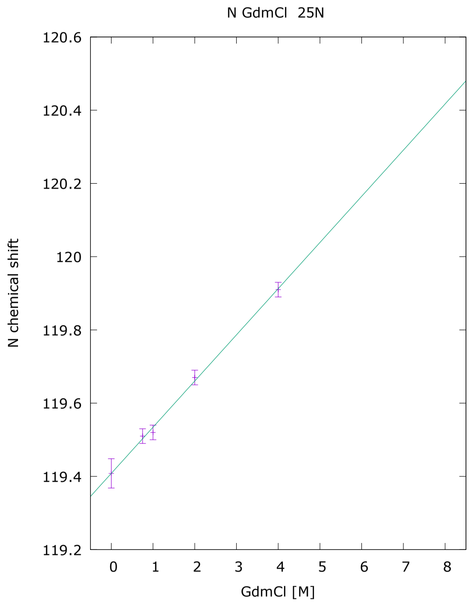
N GdmCl 25N
N chemical shift
GdmCl [M]

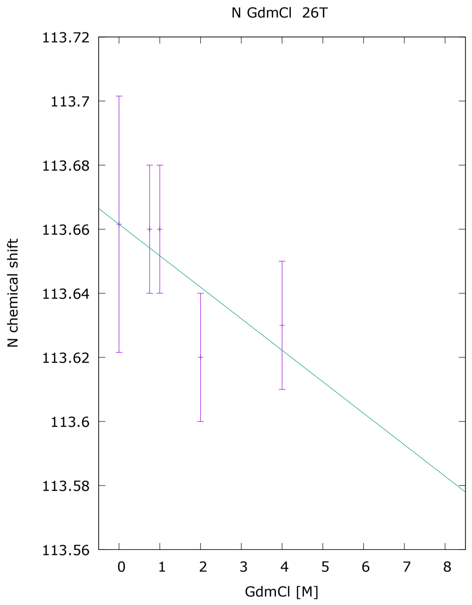
N GdmCl 26T
N chemical shift
GdmCl [M]

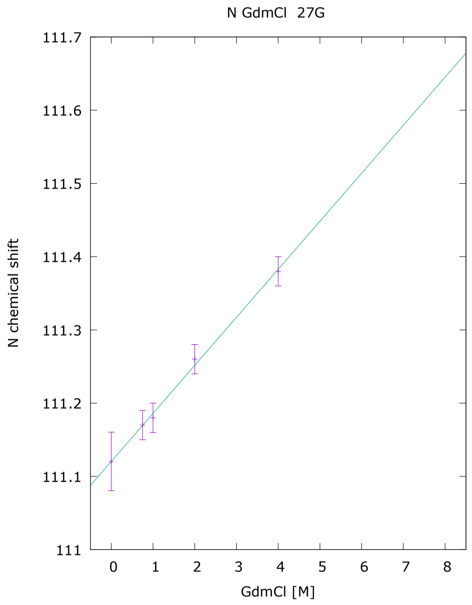
N GdmCl 27G
N chemical shift
GdmCl [M]

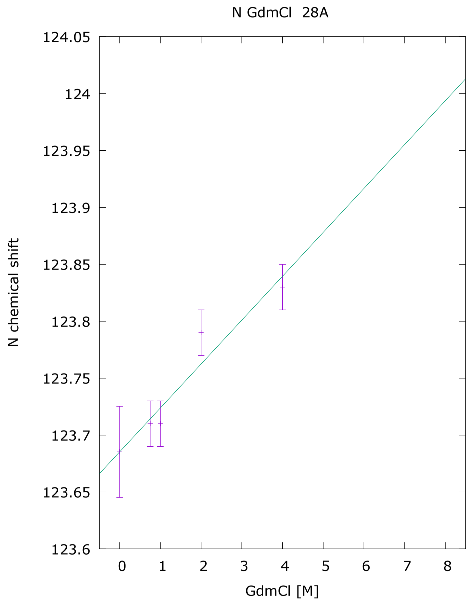
N GdmCl 28A
N chemical shift
GdmCl [M]

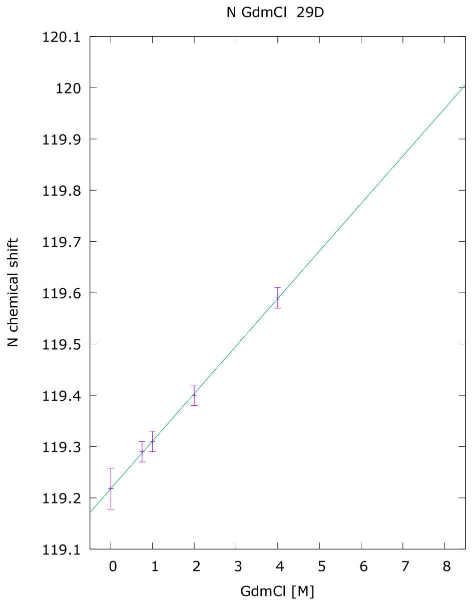
N GdmCl 29D
N chemical shift
GdmCl [M]

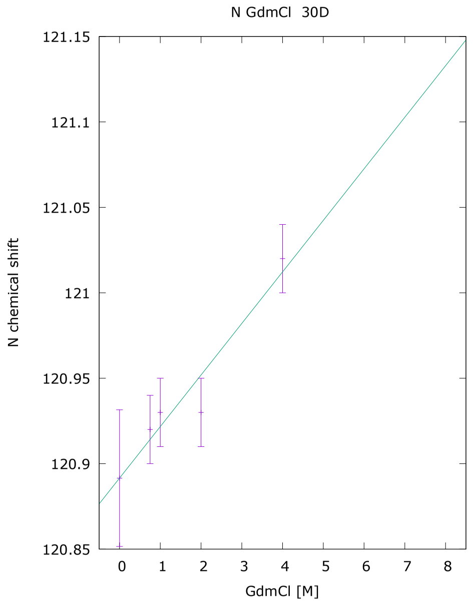
N GdmCl 30D
N chemical shift
GdmCl [M]

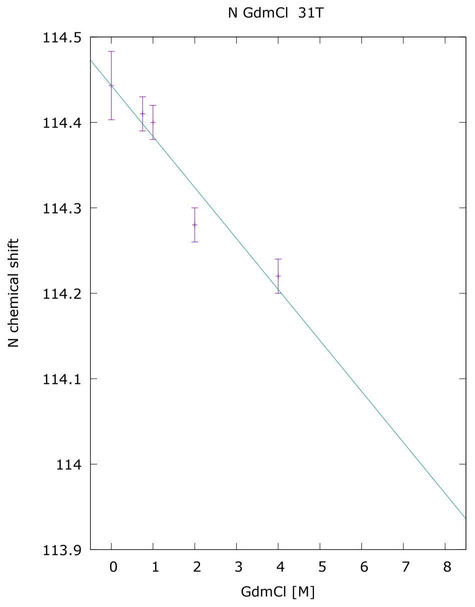
N GdmCl 31T
N chemical shift
GdmCl [M]

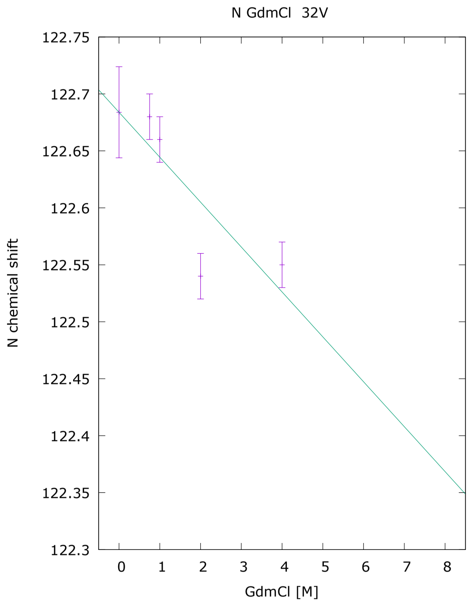
N GdmCl 32V
N chemical shift
GdmCl [M]

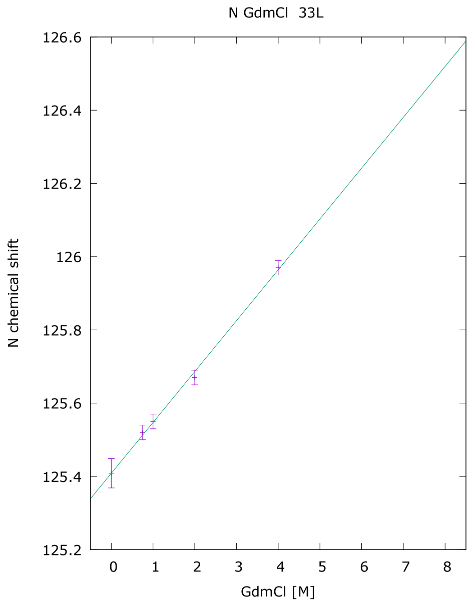
N GdmCl 33L
N chemical shift
GdmCl [M]

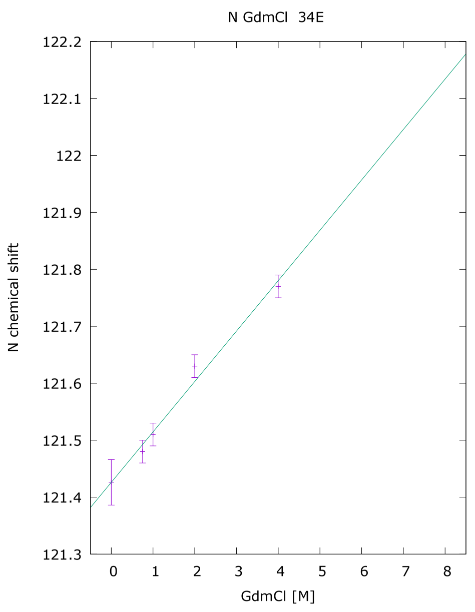
N GdmCl 34E
N chemical shift
GdmCl [M]

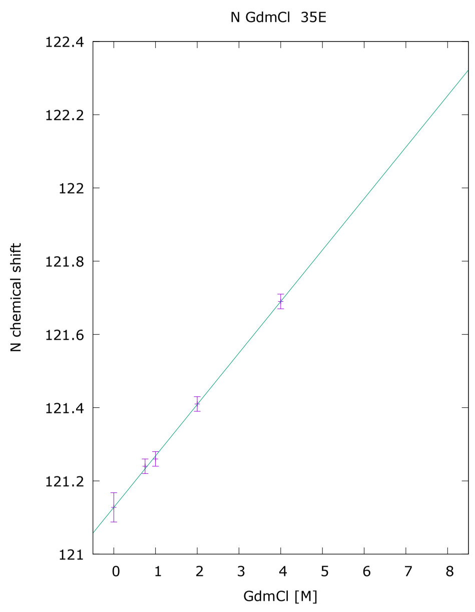
N GdmCl 35E
N chemical shift
GdmCl [M]

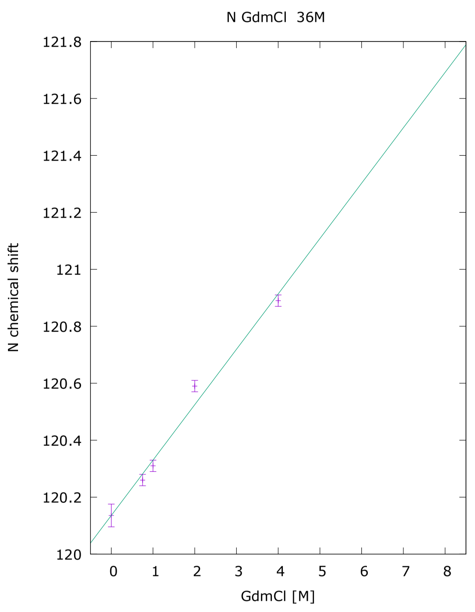
N GdmCl 36M
N chemical shift
GdmCl [M]

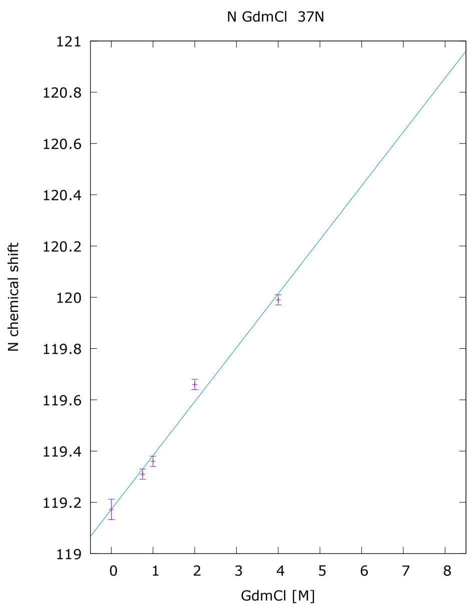
N GdmCl 37N
N chemical shift
GdmCl [M]

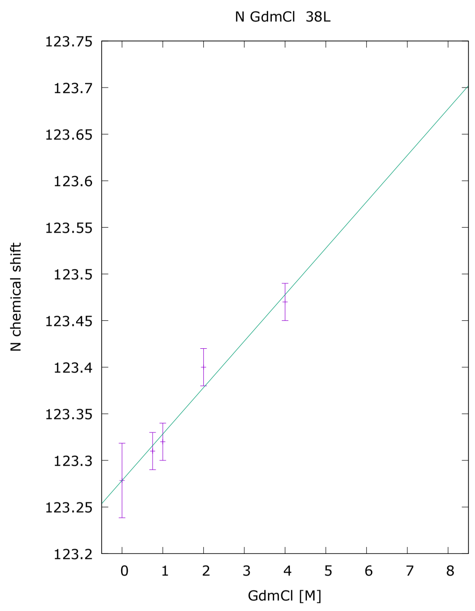
N GdmCl 38L
N chemical shift
GdmCl [M]

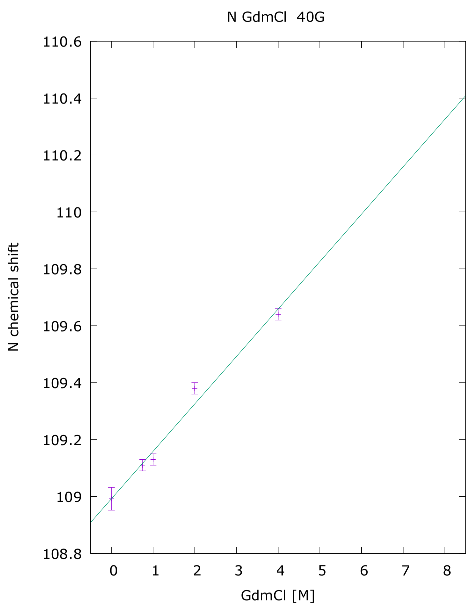
N GdmCl 40G
N chemical shift
GdmCl [M]

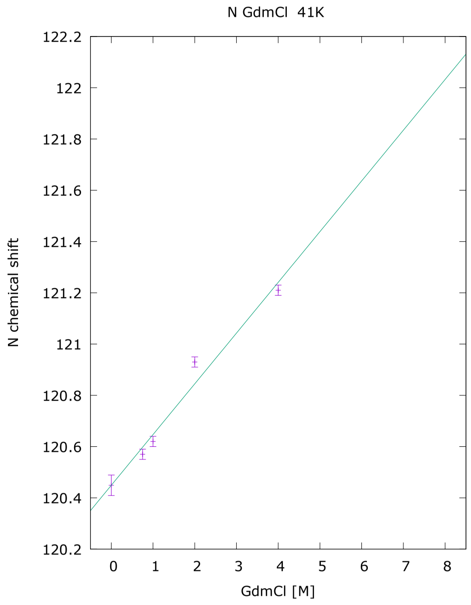
N GdmCl 41K
N chemical shift
GdmCl [M]
122.2
122
121.8
121.6
121.4
121.2
121
120.8
120.6
120.4
120.2
0
1
2
3
4
5
6
7
8

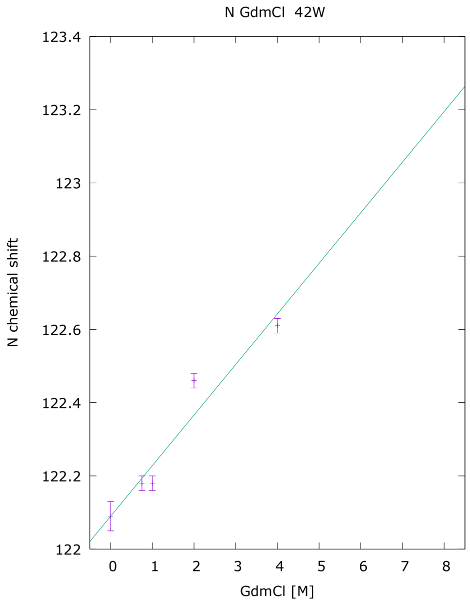
N GdmCl 42W
N chemical shift
GdmCl [M]
123.4
123.2
123
122.8
122.6
122.4
122.2
122
0
1
2
3
4
5
6
7
8

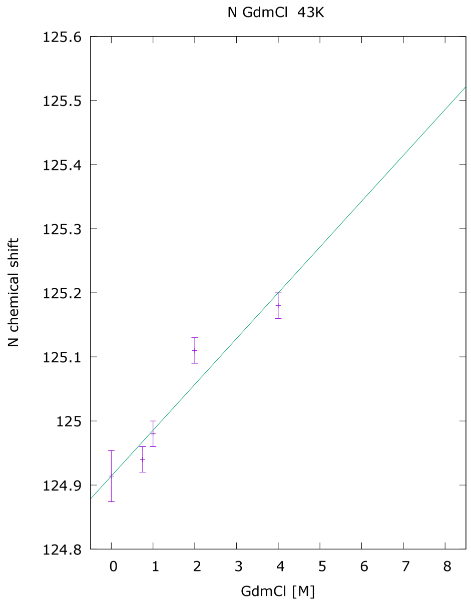
N GdmCl 43K
N chemical shift
GdmCl [M]
125.6
125.5
125.4
125.3
125.2
125.1
125
124.9
124.8
0
1
2
3
4
5
6
7
8

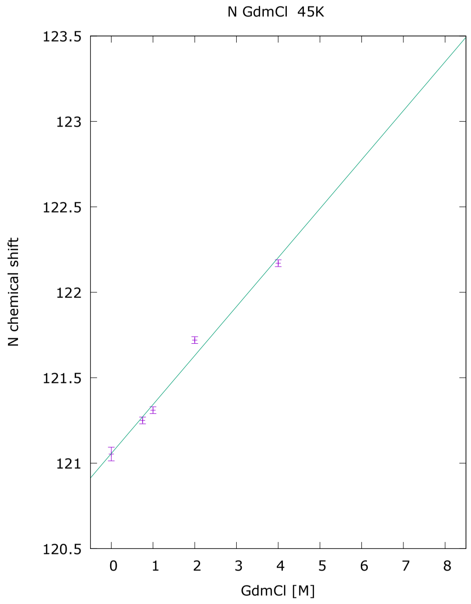
N GdmCl 45K
N chemical shift
GdmCl [M]
123.5
123
122.5
122
121.5
121
120.5
0
1
2
3
4
5
6
7
8

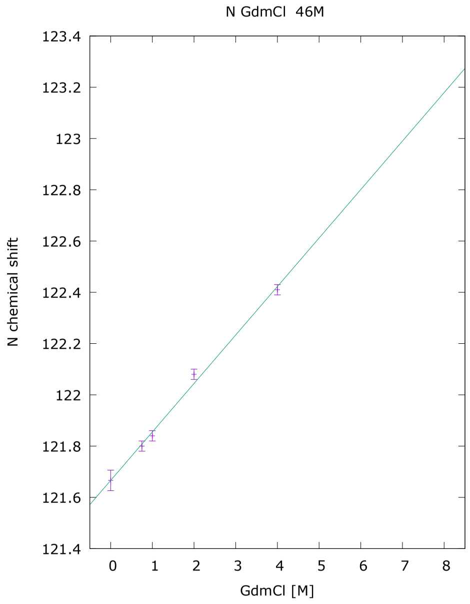
N GdmCl 46M
N chemical shift
GdmCl [M]
123.4
123.2
123
122.8
122.6
122.4
122.2
122
121.8
121.6
121.4
0
1
2
3
4
5
6
7
8

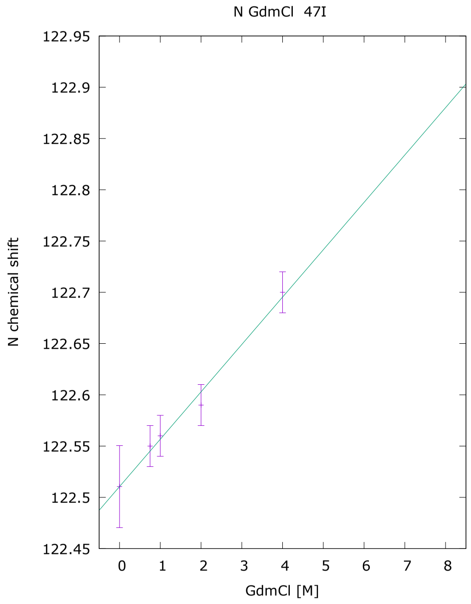
N GdmCl 47I
N chemical shift
GdmCl [M]
122.95
122.9
122.85
122.8
122.75
122.7
122.65
122.6
122.55
122.5
122.45
0
1
2
3
4
5
6
7
8

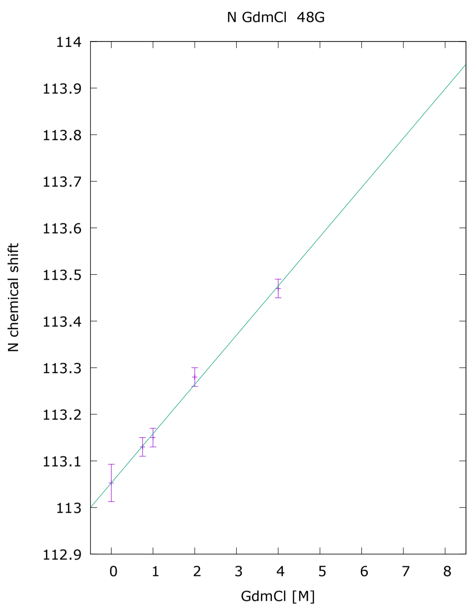
N GdmCl 48G
N chemical shift
GdmCl [M]

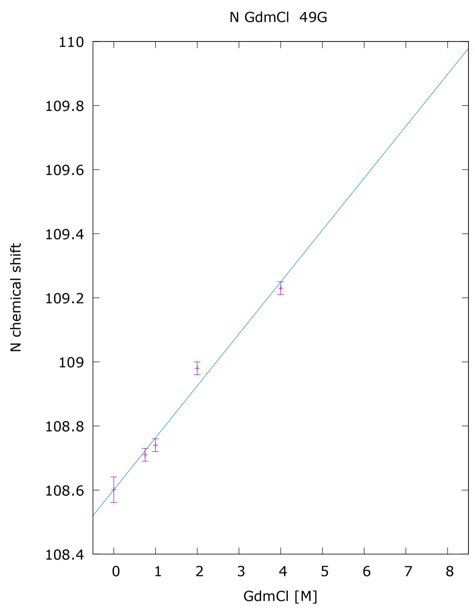
N GdmCl 49G
N chemical shift
GdmCl [M]

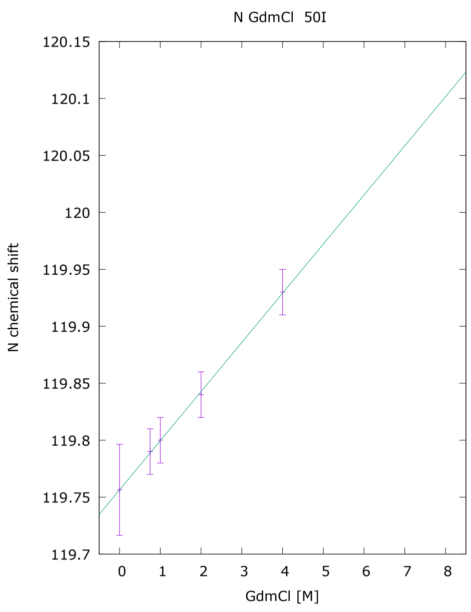
N GdmCl 50I
N chemical shift
GdmCl [M]

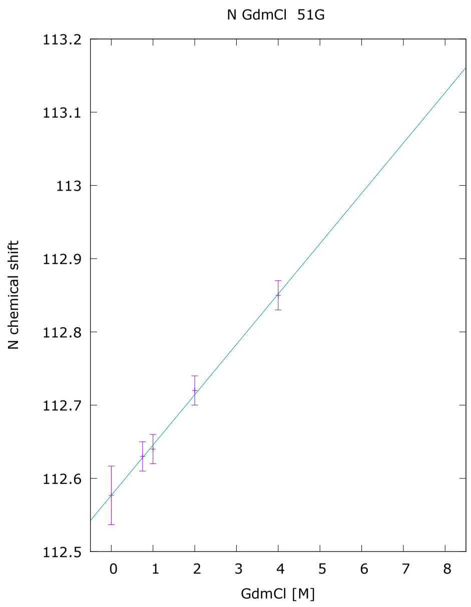
N GdmCl 51G
N chemical shift
GdmCl [M]

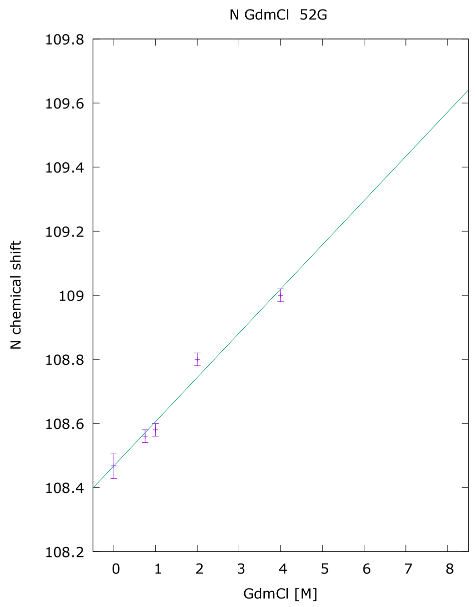
N GdmCl 52G
N chemical shift
GdmCl [M]

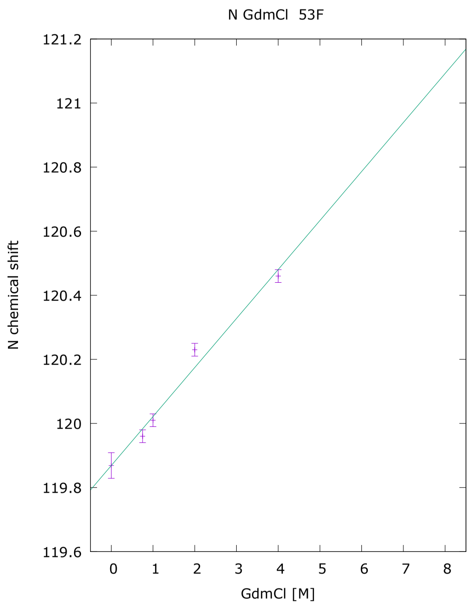
N GdmCl 53F
N chemical shift
GdmCl [M]

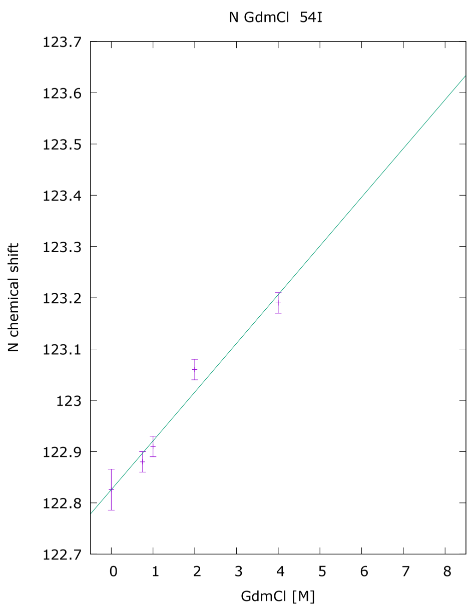
N GdmCl 54I
N chemical shift
GdmCl [M]

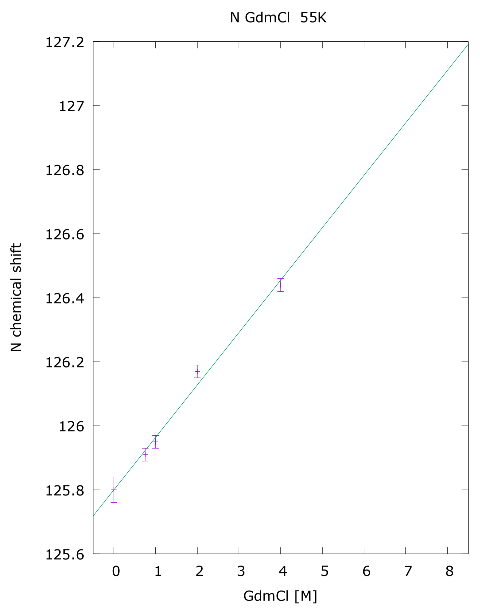
N GdmCl 55K
N chemical shift
GdmCl [M]

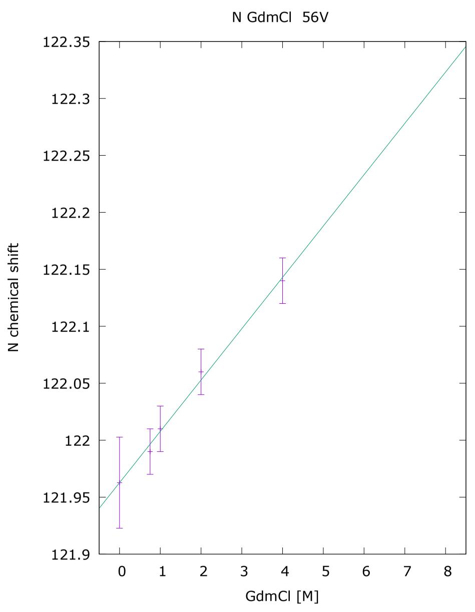
N GdmCl 56V
N chemical shift
GdmCl [M]

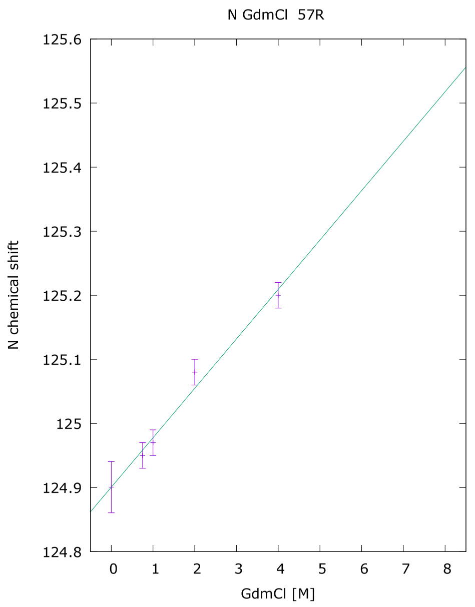
N GdmCl 57R
N chemical shift
GdmCl [M]

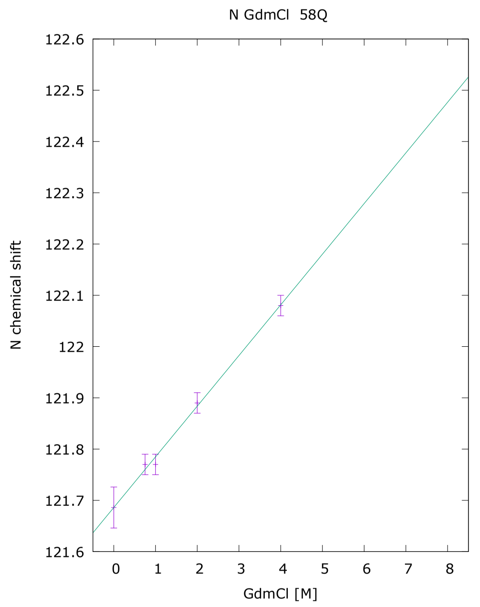
N GdmCl 58Q
N chemical shift
GdmCl [M]

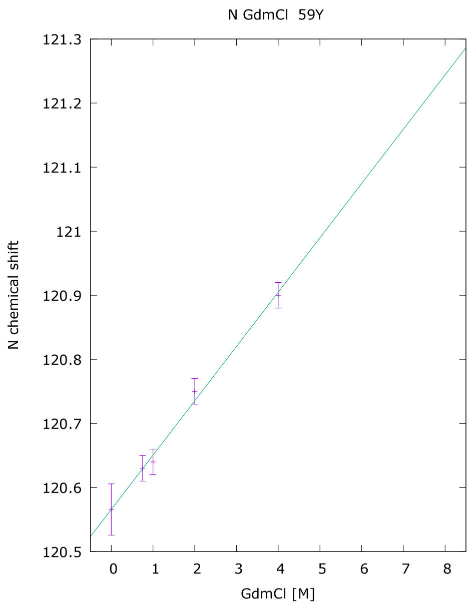
N GdmCl 59Y
N chemical shift
GdmCl [M]

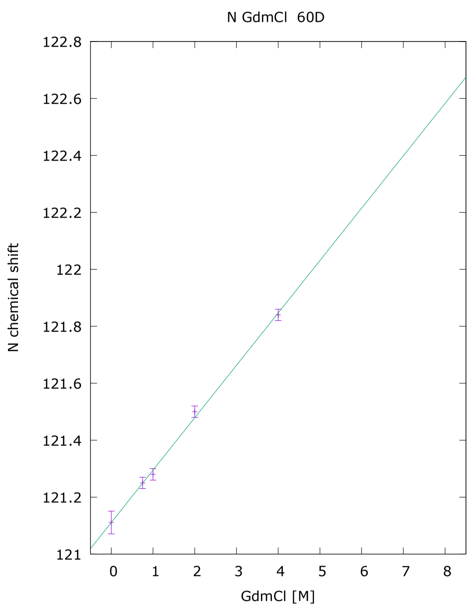
N GdmCl 60D
N chemical shift
GdmCl [M]

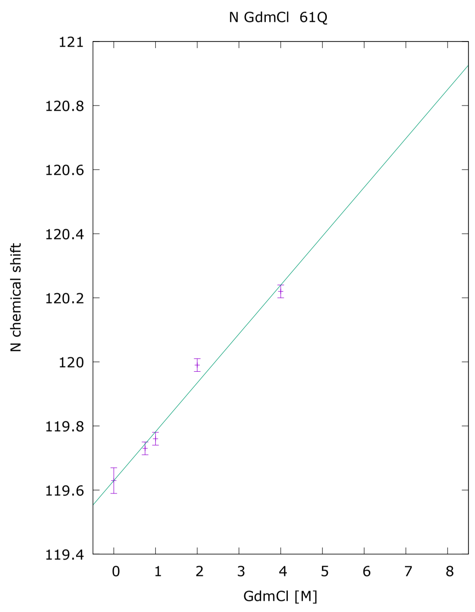
N GdmCl 61Q
N chemical shift
GdmCl [M]

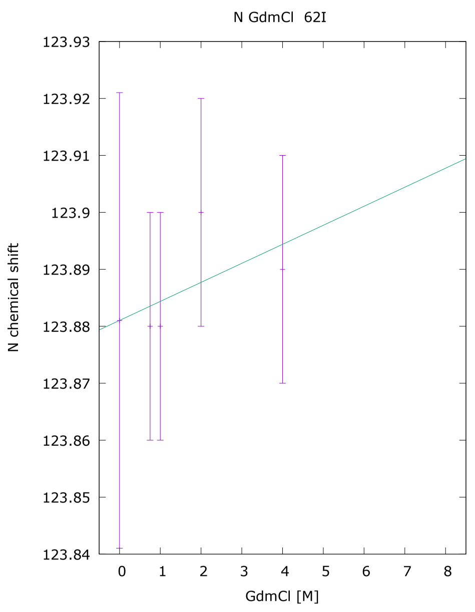
N GdmCl 62I
N chemical shift
GdmCl [M]

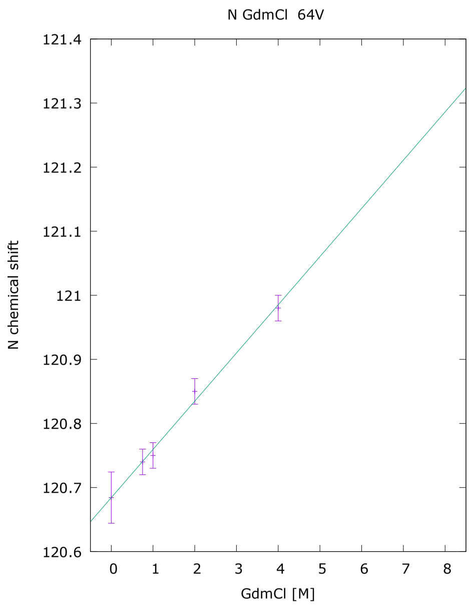
N GdmCl 64V
N chemical shift
GdmCl [M]

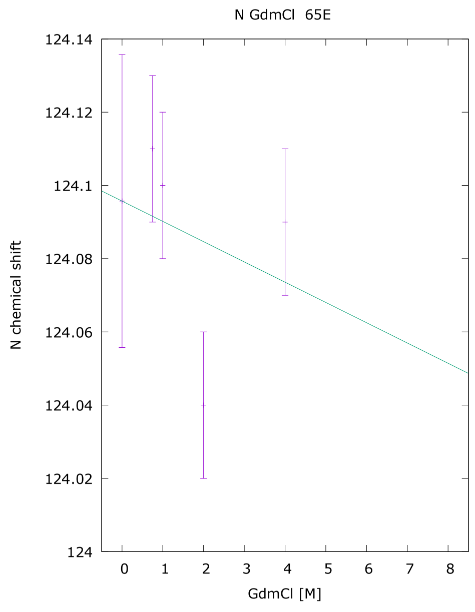
N GdmCl 65E
N chemical shift
GdmCl [M]

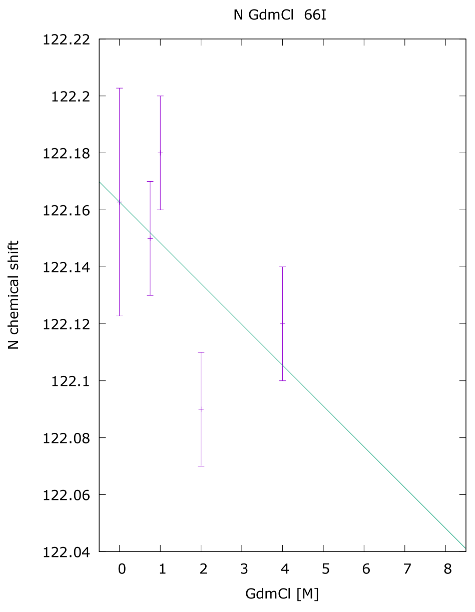
N GdmCl 66I
N chemical shift
GdmCl [M]

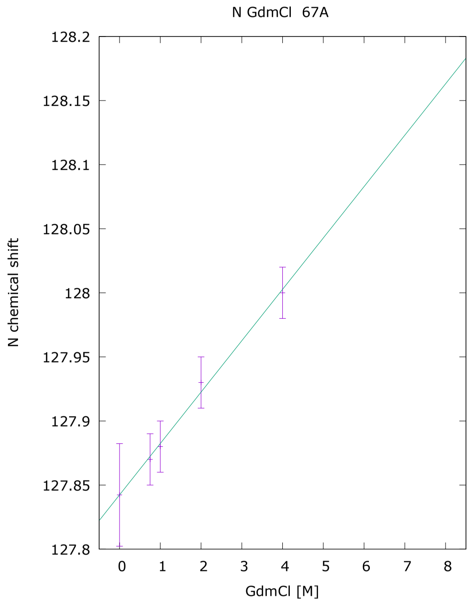
N GdmCl 67A
N chemical shift
GdmCl [M]

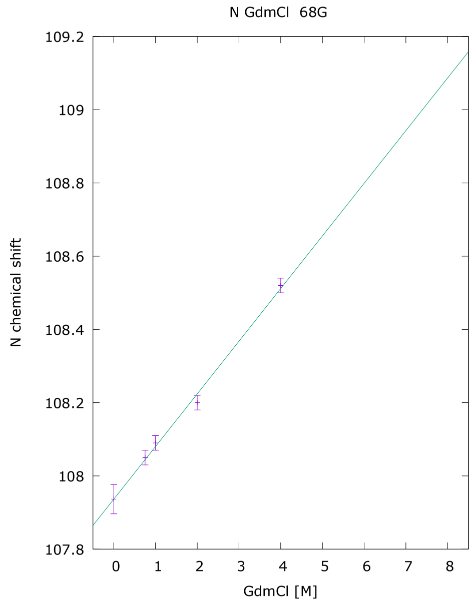
N GdmCl 68G
N chemical shift
GdmCl [M]

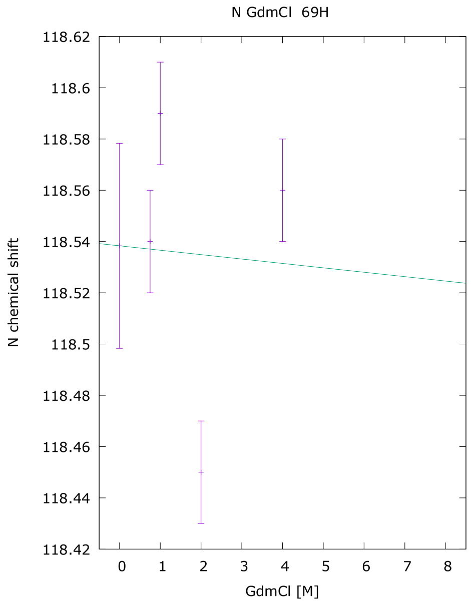
N GdmCl 69H
N chemical shift
GdmCl [M]

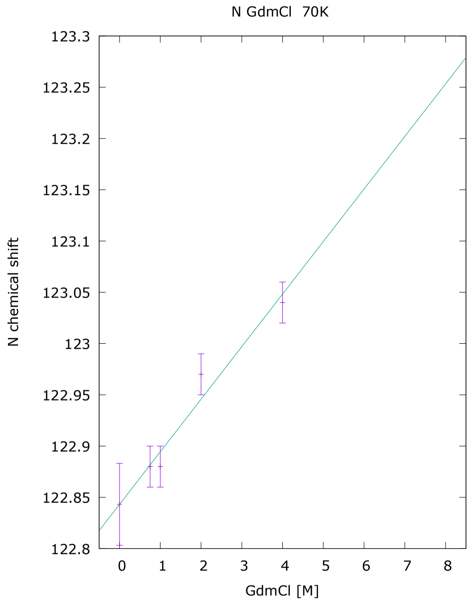
N GdmCl 70K
N chemical shift
GdmCl [M]

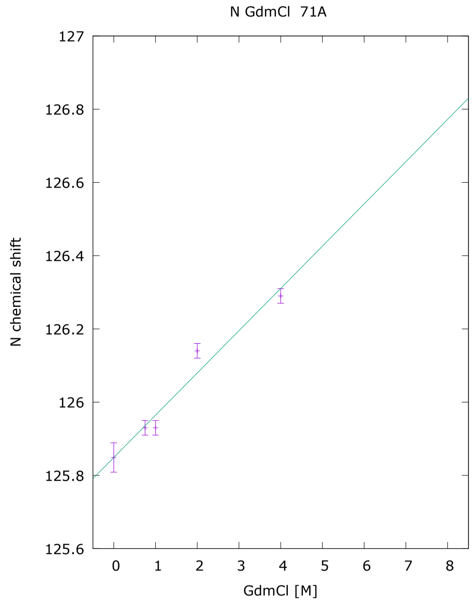
N GdmCl 71A
N chemical shift
GdmCl [M]

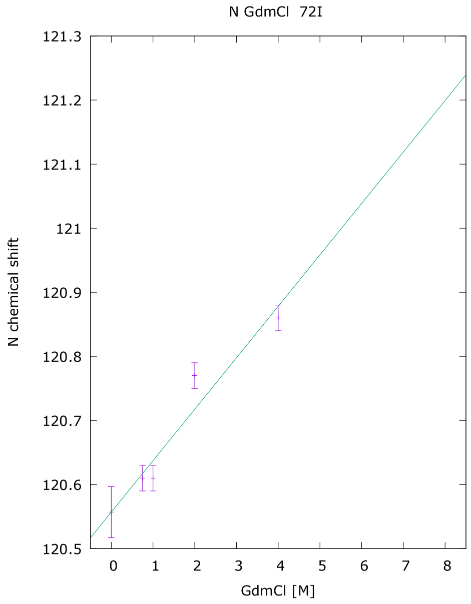
N GdmCl 72I
N chemical shift
GdmCl [M]

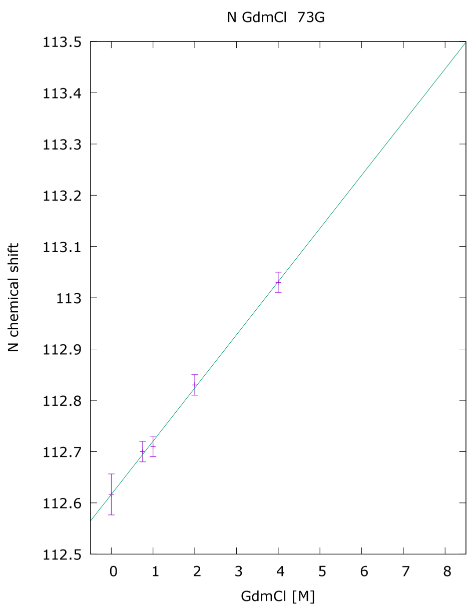
N GdmCl 73G
N chemical shift
GdmCl [M]

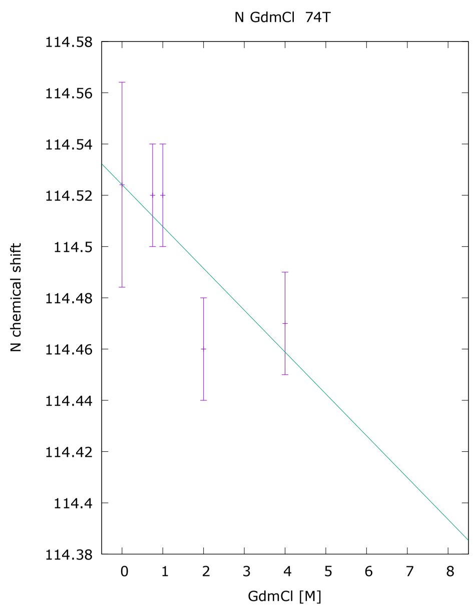
N GdmCl 74T
N chemical shift
GdmCl [M]

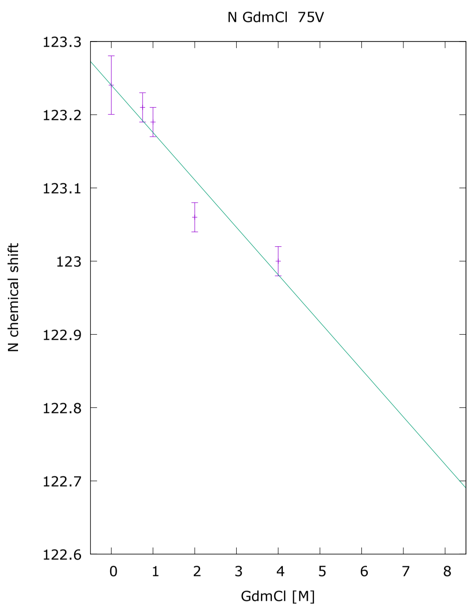
N GdmCl 75V
N chemical shift
GdmCl [M]

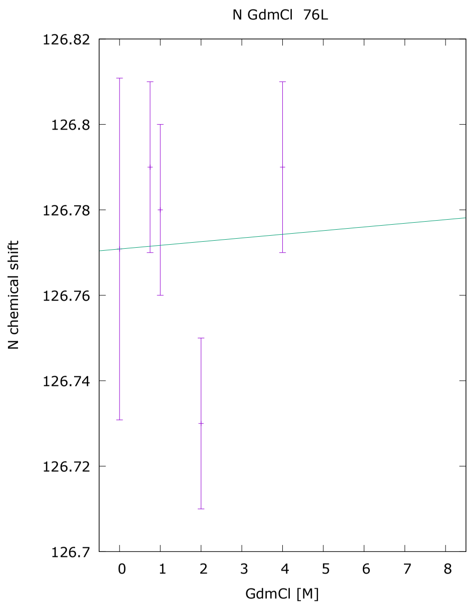
N GdmCl 76L
N chemical shift
GdmCl [M]

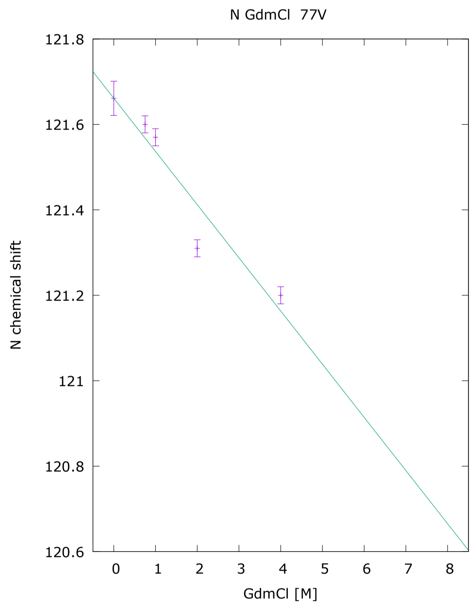
N GdmCl 77V
N chemical shift
GdmCl [M]

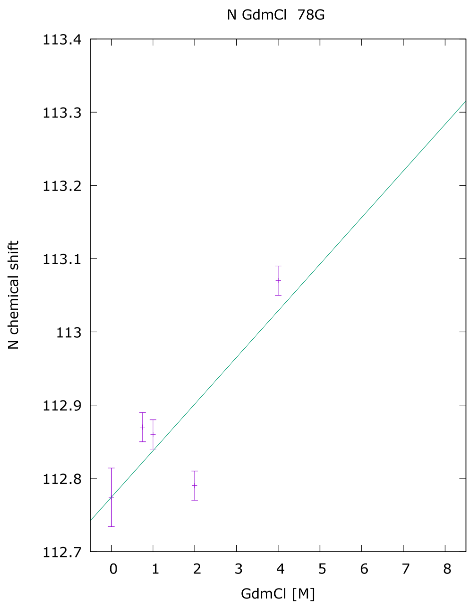
N GdmCl 78G
N chemical shift
GdmCl [M]

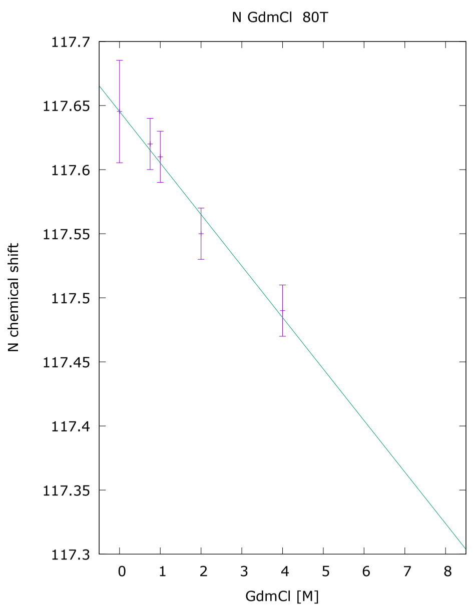
N GdmCl 80T
N chemical shift
GdmCl [M]

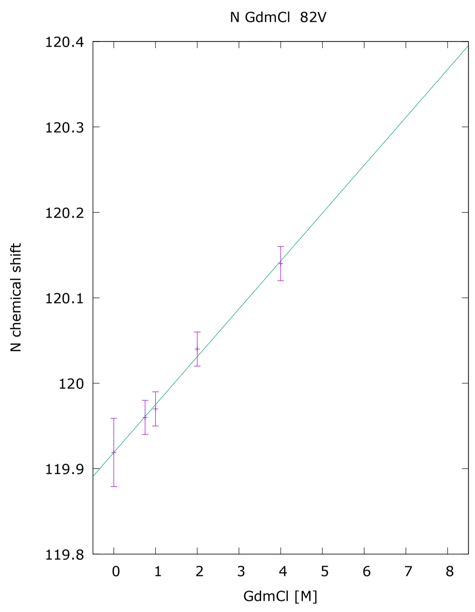
N GdmCl 82V
N chemical shift
GdmCl [M]

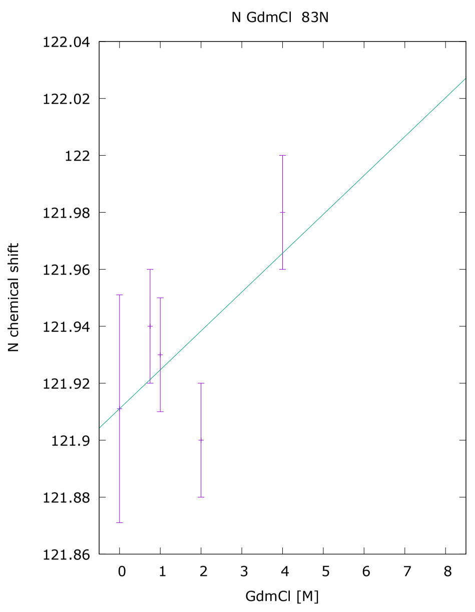
N GdmCl 83N
N chemical shift
GdmCl [M]

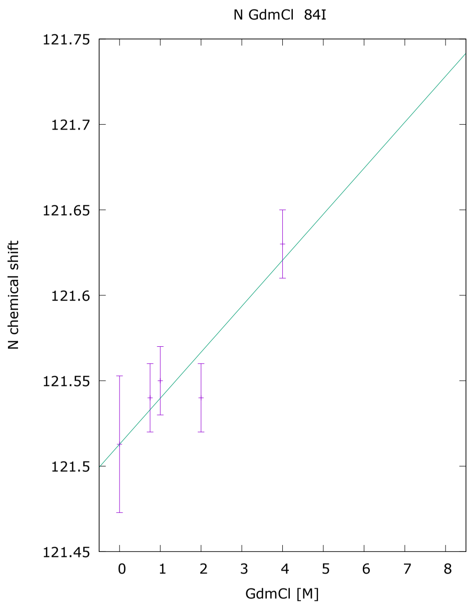
N GdmCl 84I
N chemical shift
GdmCl [M]

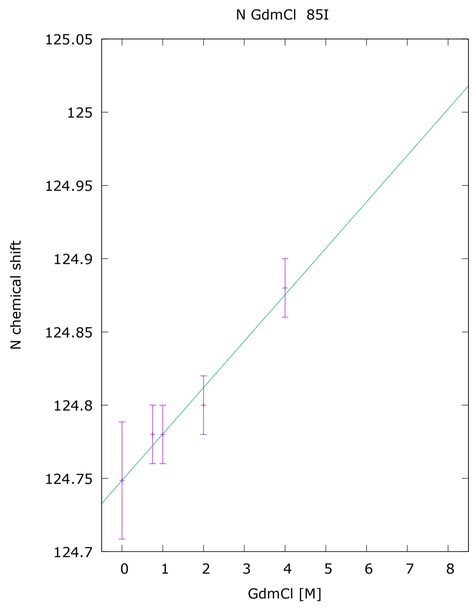
N GdmCl 85I
N chemical shift
GdmCl [M]

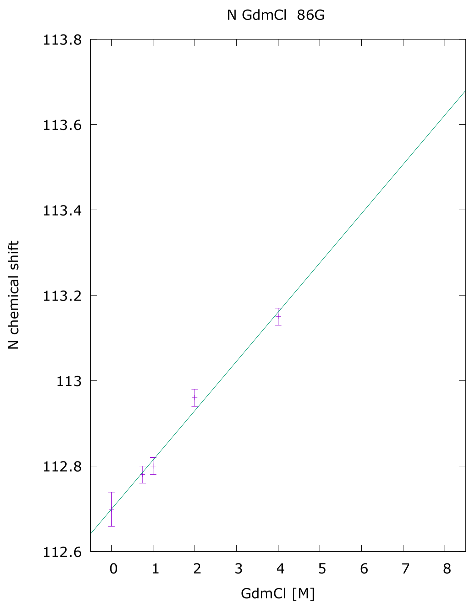
N GdmCl 86G
N chemical shift
GdmCl [M]

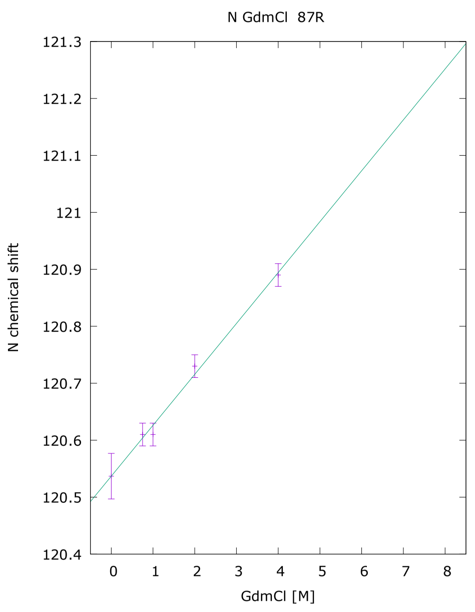
N GdmCl 87R
N chemical shift
GdmCl [M]

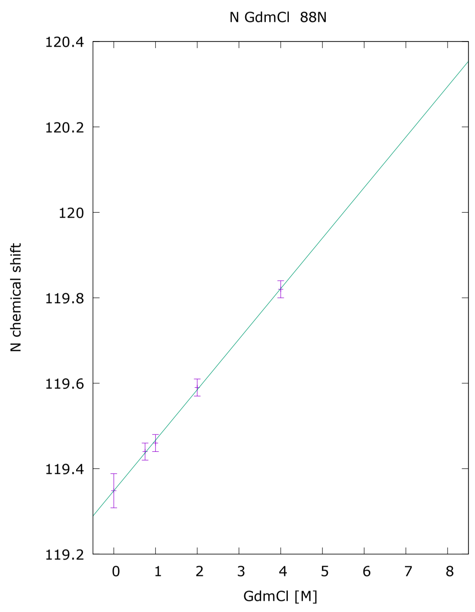
N GdmCl 88N
N chemical shift
GdmCl [M]

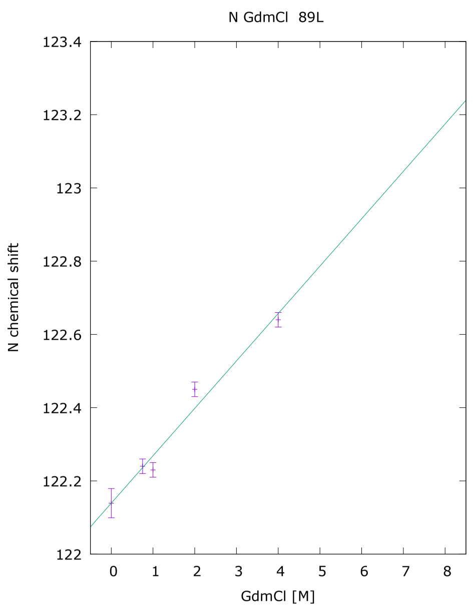
N GdmCl 89L
N chemical shift
GdmCl [M]

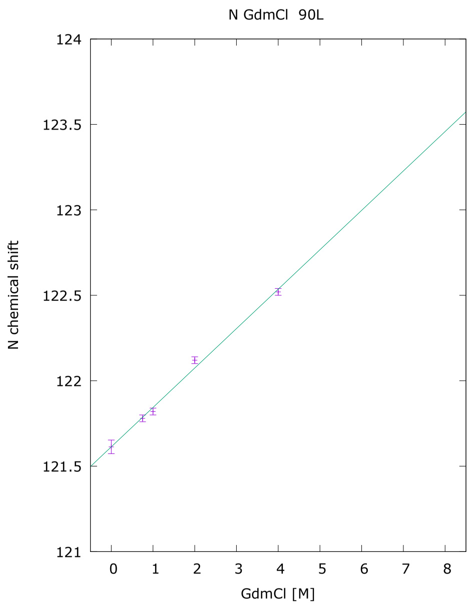
N GdmCl 90L
N chemical shift
GdmCl [M]

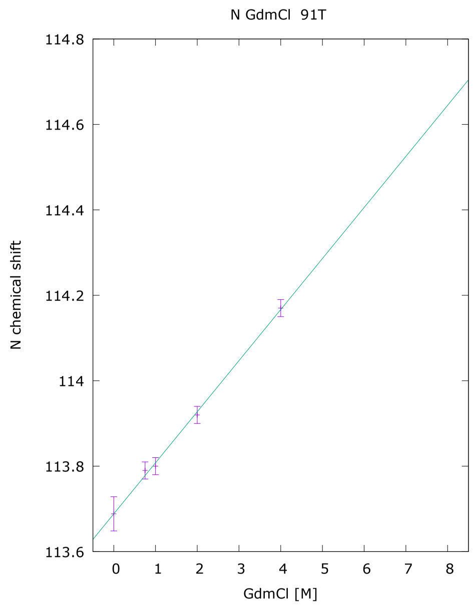
N GdmCl 91T
N chemical shift
GdmCl [M]

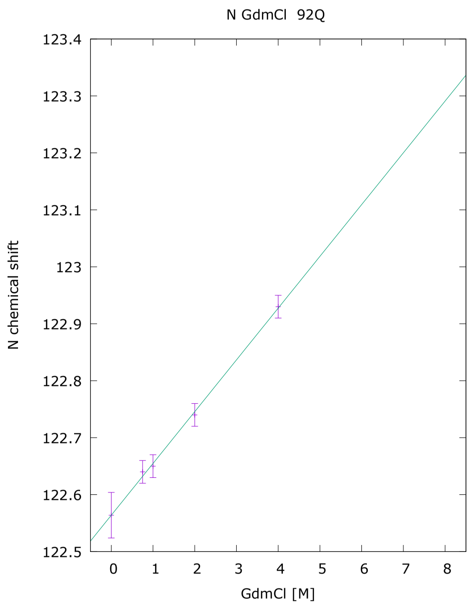
N GdmCl 92Q
N chemical shift
GdmCl [M]

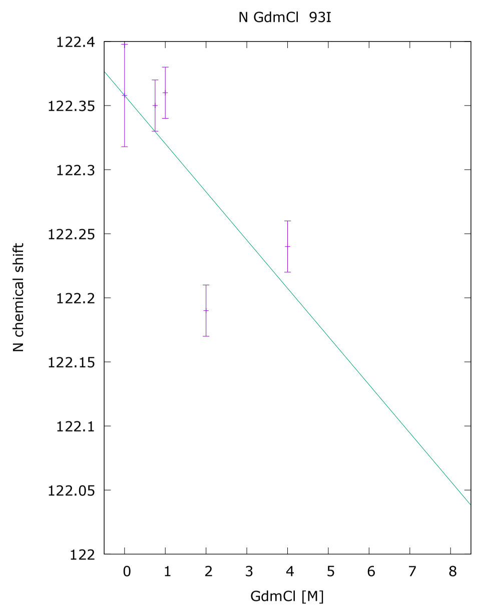
N GdmCl 93I
N chemical shift
GdmCl [M]
122.4
122.35
122.3
122.25
122.2
122.15
122.1
122.05
122
0 1 2 3 4 5 6 7 8

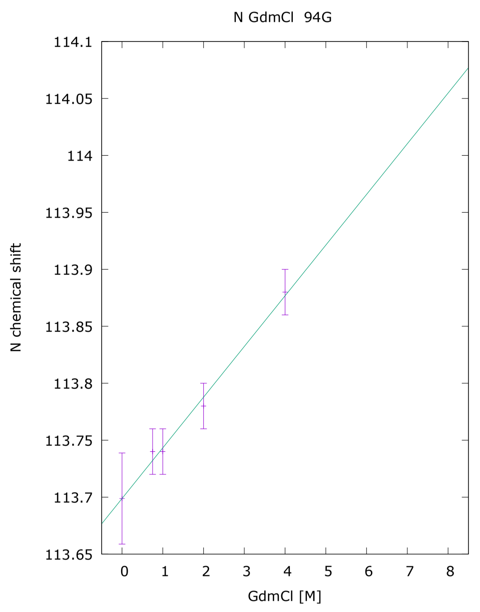
N GdmCl 94G
N chemical shift
GdmCl [M]
114.1
114.05
114
113.95
113.9
113.85
113.8
113.75
113.7
113.65
0 1 2 3 4 5 6 7 8

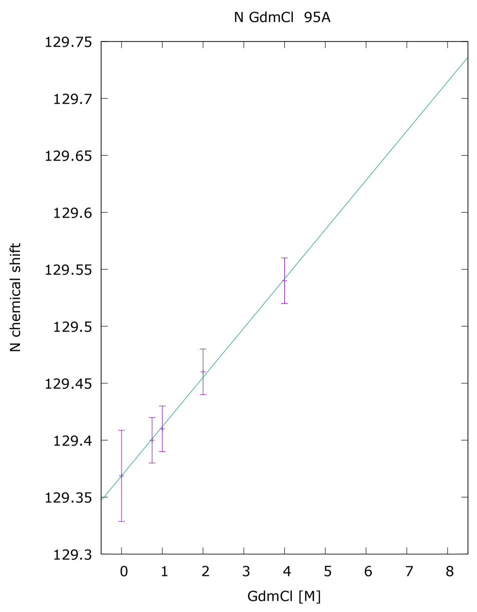
N GdmCl 95A
N chemical shift
GdmCl [M]
129.75
129.7
129.65
129.6
129.55
129.5
129.45
129.4
129.35
129.3
0 1 2 3 4 5 6 7 8

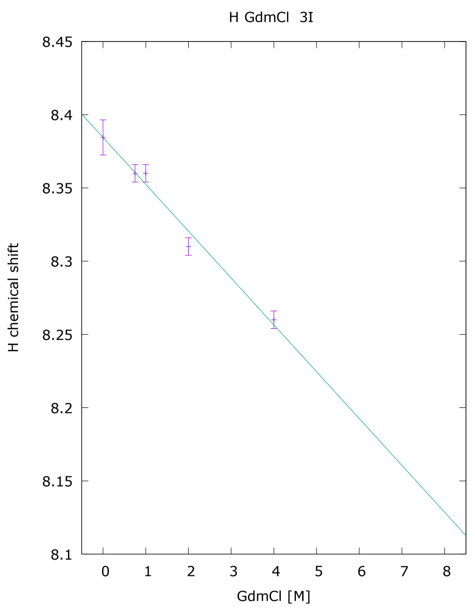
H GdmCl 3I
H chemical shift
8.45
8.4
8.35
8.3
8.25
8.2
8.15
8.1
0 1 2 3 4 5 6 7 8
GdmCl [M]

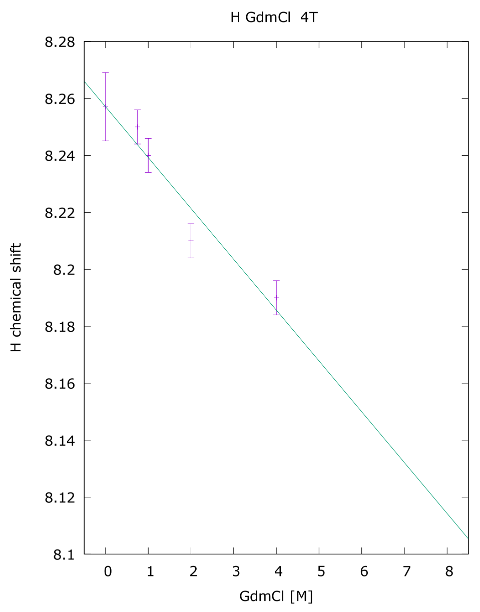
H GdmCl 4T
H chemical shift
8.28
8.26
8.24
8.22
8.2
8.18
8.16
8.14
8.12
8.1
0 1 2 3 4 5 6 7 8
GdmCl [M]

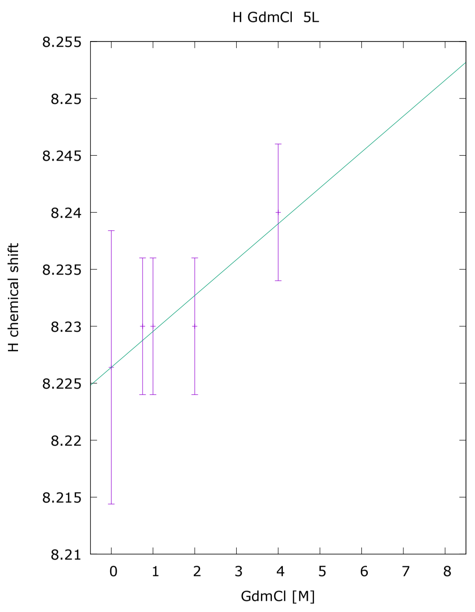
H GdmCl 5L
H chemical shift
8.255
8.25
8.245
8.24
8.235
8.23
8.225
8.22
8.215
8.21
0 1 2 3 4 5 6 7 8
GdmCl [M]

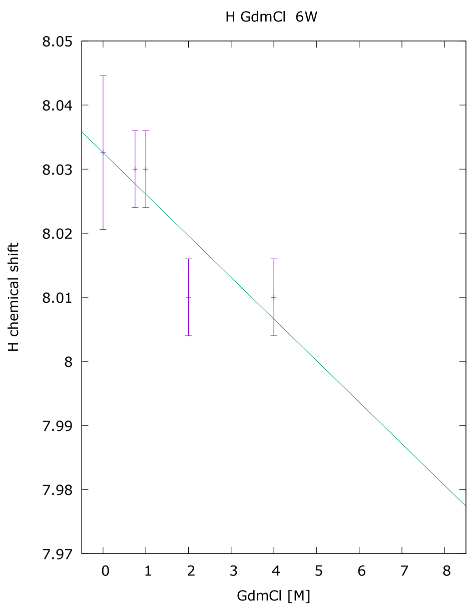
H GdmCl 6W
H chemical shift
8.05
8.04
8.03
8.02
8.01
8
7.99
7.98
7.97
0 1 2 3 4 5 6 7 8
GdmCl [M]

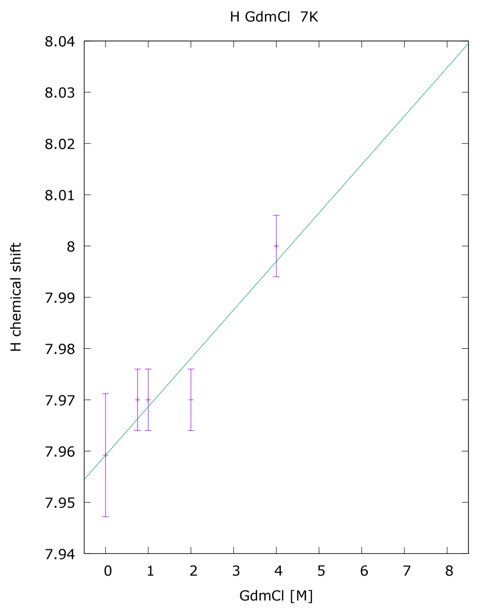
H GdmCl 7K
H chemical shift
8.04
8.03
8.02
8.01
8
7.99
7.98
7.97
7.96
7.95
7.94
0 1 2 3 4 5 6 7 8
GdmCl [M]

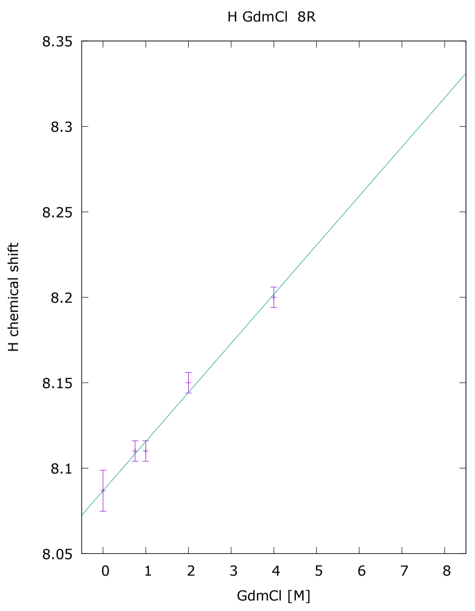
H GdmCl 8R
H chemical shift
8.35
8.3
8.25
8.2
8.15
8.1
8.05
0 1 2 3 4 5 6 7 8
GdmCl [M]

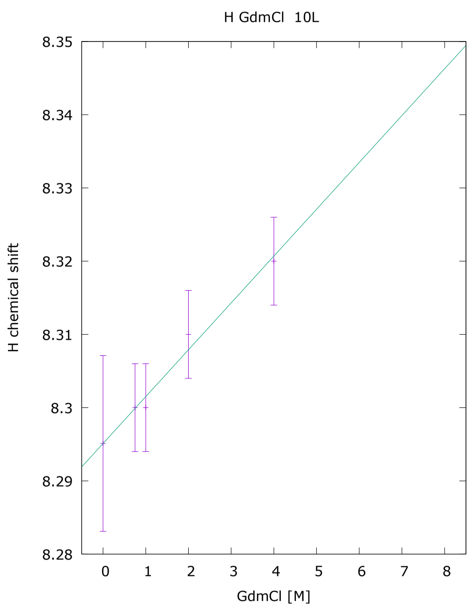
H GdmCl 10L
H chemical shift
GdmCl [M]

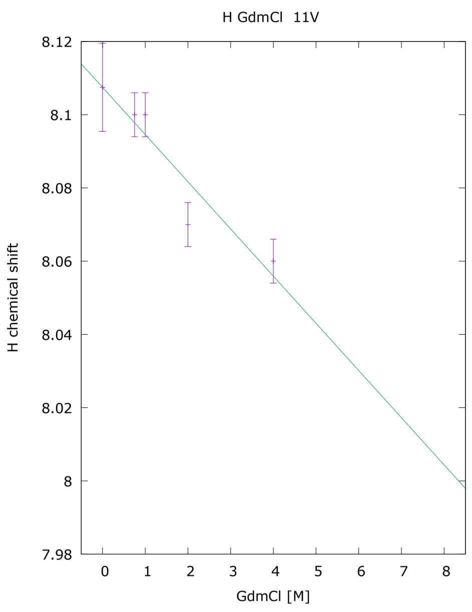
H GdmCl 11V
H chemical shift
GdmCl [M]

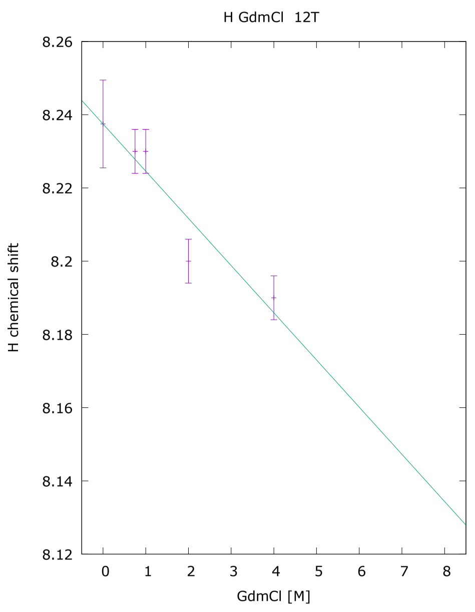
H GdmCl 12T
H chemical shift
GdmCl [M]

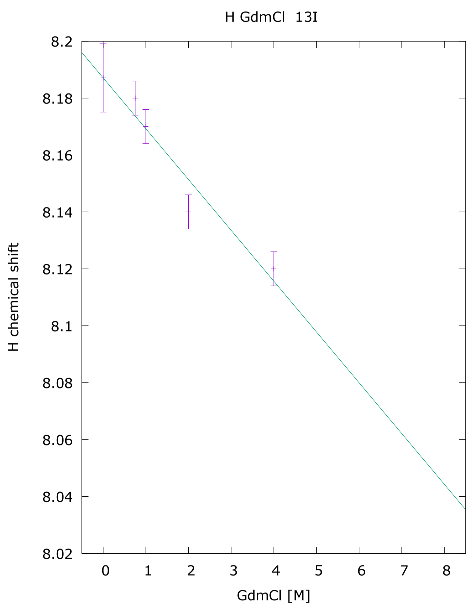
H GdmCl 13I
H chemical shift
GdmCl [M]

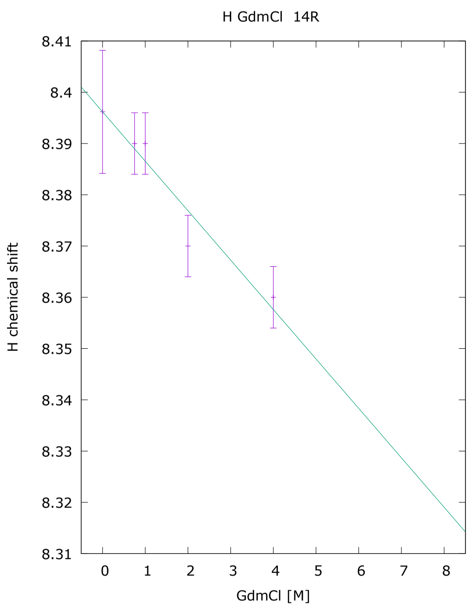
H GdmCl 14R
H chemical shift
GdmCl [M]

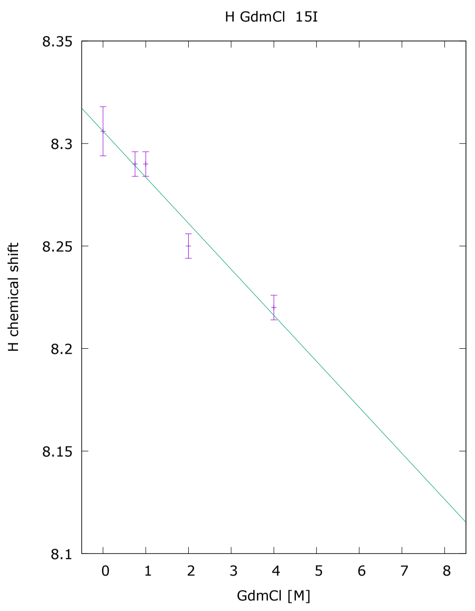
H GdmCl 15I
H chemical shift
GdmCl [M]

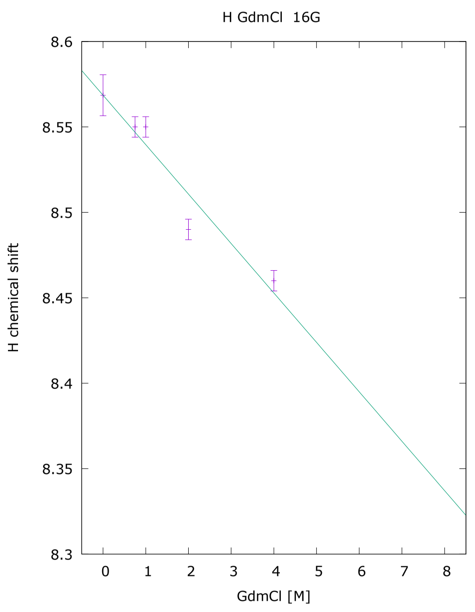
H GdmCl 16G
H chemical shift
GdmCl [M]
8.6
8.55
8.5
8.45
8.4
8.35
8.3
0 1 2 3 4 5 6 7 8

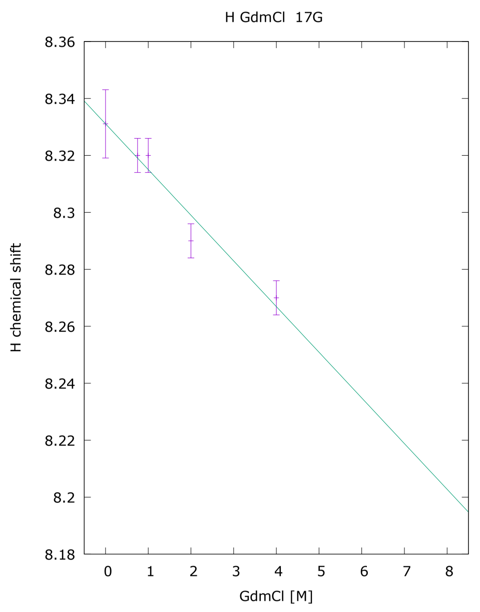
H GdmCl 17G
H chemical shift
GdmCl [M]
8.36
8.34
8.32
8.3
8.28
8.26
8.24
8.22
8.2
8.18
0 1 2 3 4 5 6 7 8

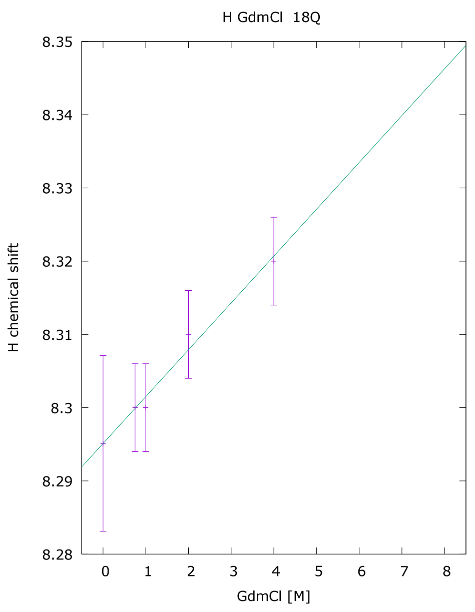
H GdmCl 18Q
H chemical shift
GdmCl [M]
8.35
8.34
8.33
8.32
8.31
8.3
8.29
8.28
0 1 2 3 4 5 6 7 8

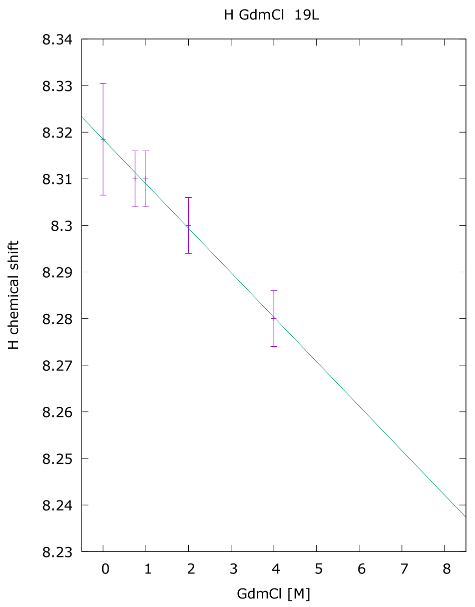
H GdmCl 19L
H chemical shift
GdmCl [M]
8.34
8.33
8.32
8.31
8.3
8.29
8.28
8.27
8.26
8.25
8.24
8.23
0 1 2 3 4 5 6 7 8

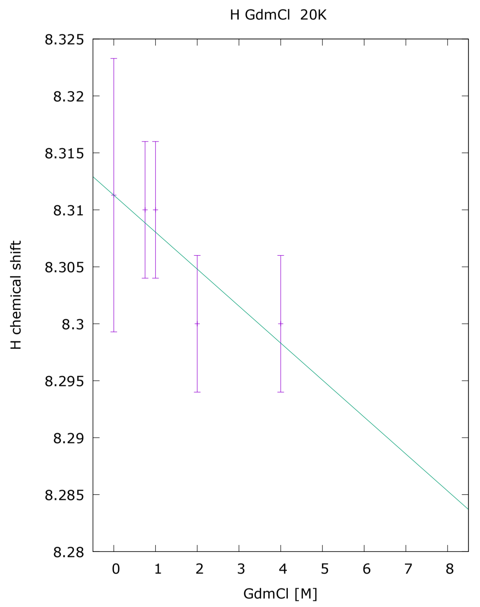
H GdmCl 20K
H chemical shift
GdmCl [M]
8.325
8.32
8.315
8.31
8.305
8.3
8.295
8.29
8.285
8.28
0 1 2 3 4 5 6 7 8

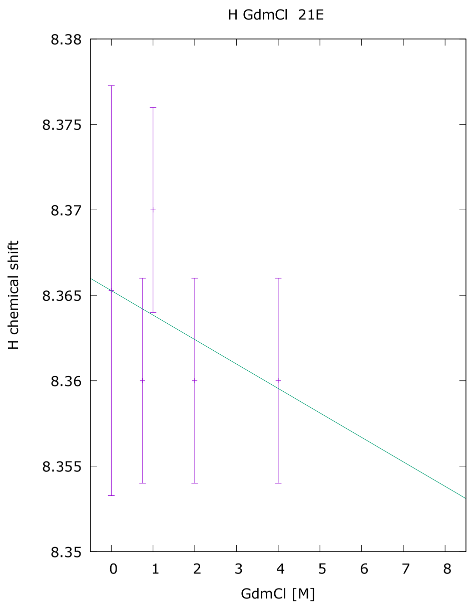
H GdmCl 21E
H chemical shift
GdmCl [M]
8.38
8.375
8.37
8.365
8.36
8.355
8.35
0 1 2 3 4 5 6 7 8

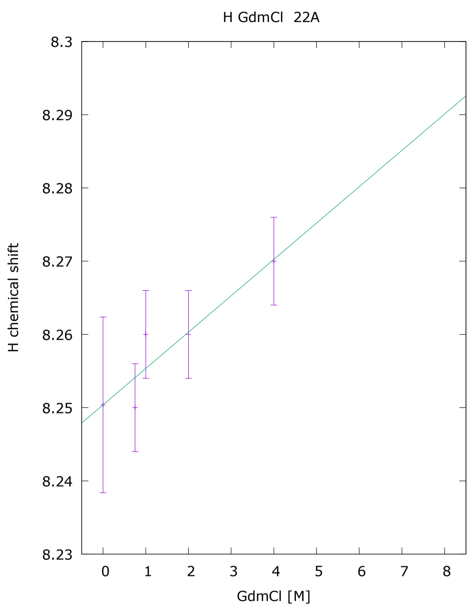
H GdmCl 22A
8.3
8.29
8.28
8.27
8.26
8.25
8.24
8.23
H chemical shift
0 1 2 3 4 5 6 7 8
GdmCl [M]
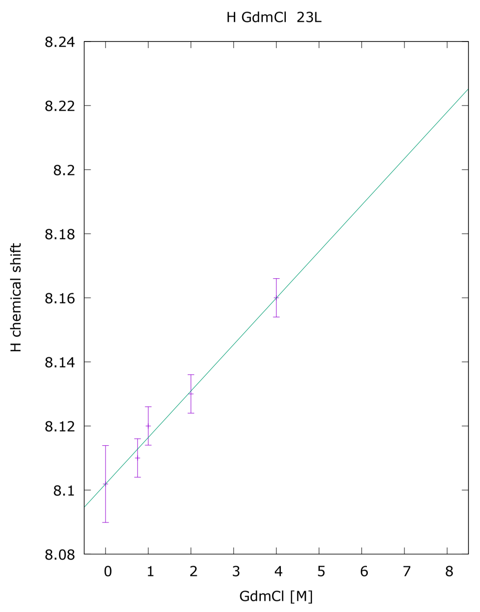
H GdmCl 23L
8.24
8.22
8.2
8.18
8.16
8.14
8.12
8.1
8.08
H chemical shift
0 1 2 3 4 5 6 7 8
GdmCl [M]
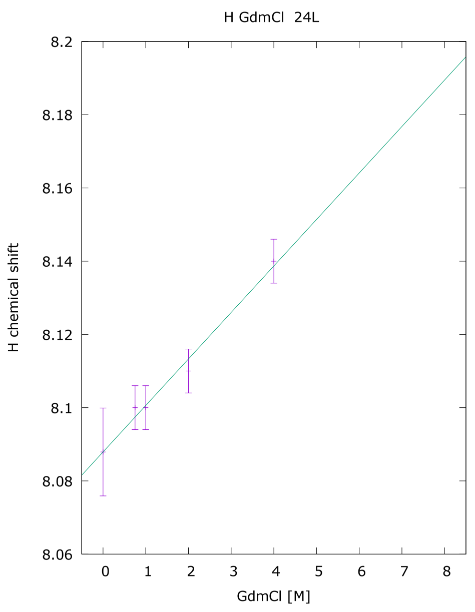
H GdmCl 24L
8.2
8.18
8.16
8.14
8.12
8.1
8.08
8.06
H chemical shift
0 1 2 3 4 5 6 7 8
GdmCl [M]
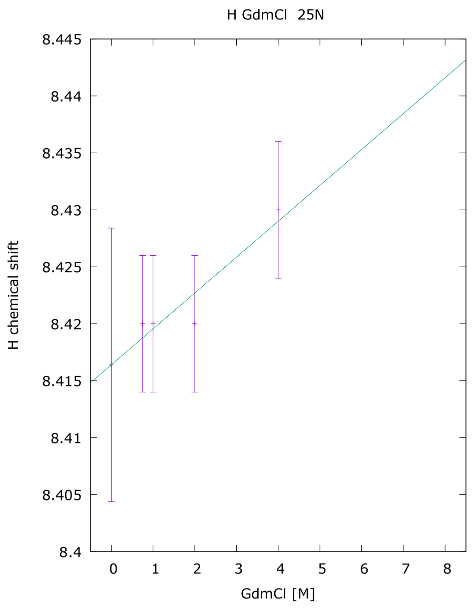
H GdmCl 25N
8.445
8.44
8.435
8.43
8.425
8.42
8.415
8.41
8.405
8.4
H chemical shift
0 1 2 3 4 5 6 7 8
GdmCl [M]
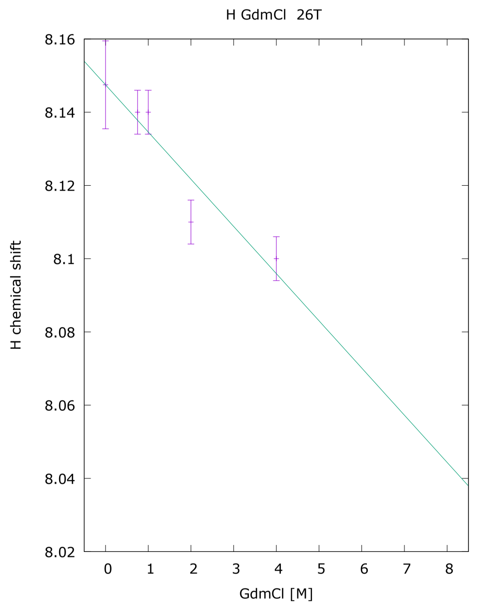
H GdmCl 26T
8.16
8.14
8.12
8.1
8.08
8.06
8.04
8.02
H chemical shift
0 1 2 3 4 5 6 7 8
GdmCl [M]
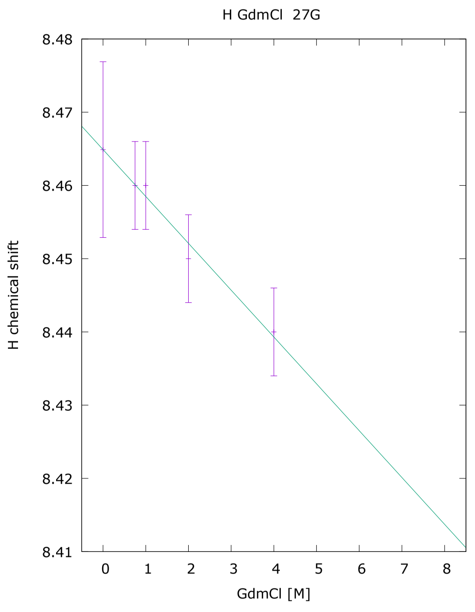
H GdmCl 27G
8.48
8.47
8.46
8.45
8.44
8.43
8.42
8.41
H chemical shift
0 1 2 3 4 5 6 7 8
GdmCl [M]

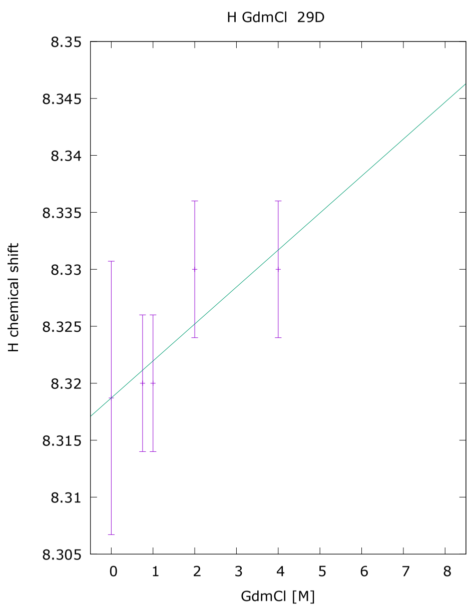
H GdmCl 29D
H chemical shift
GdmCl [M]

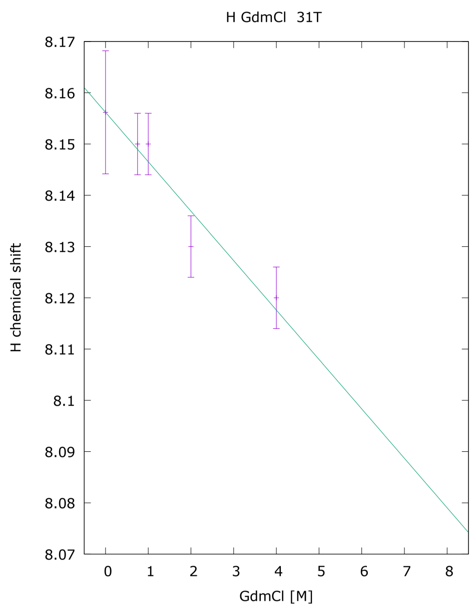
H GdmCl 31T
H chemical shift
GdmCl [M]

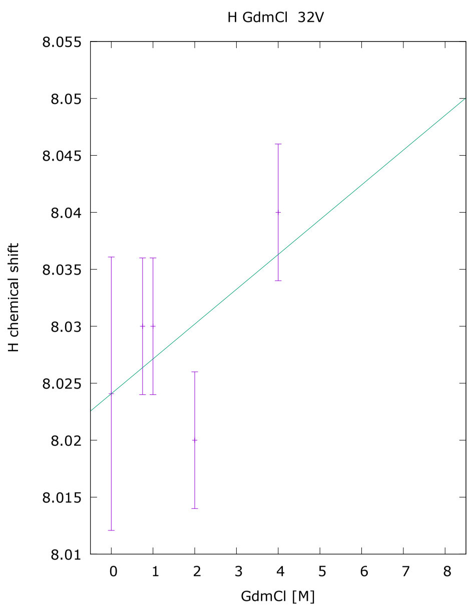
H GdmCl 32V
H chemical shift
GdmCl [M]

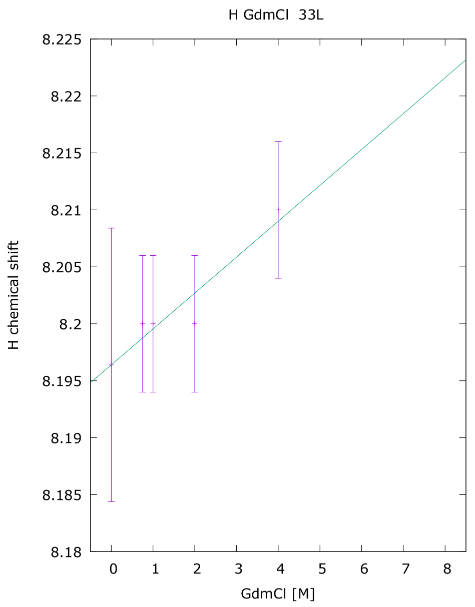
H GdmCl 33L
H chemical shift
GdmCl [M]

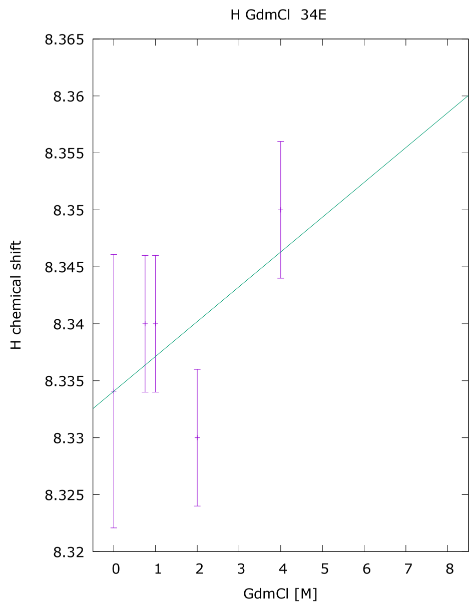
H GdmCl 34E
H chemical shift
GdmCl [M]

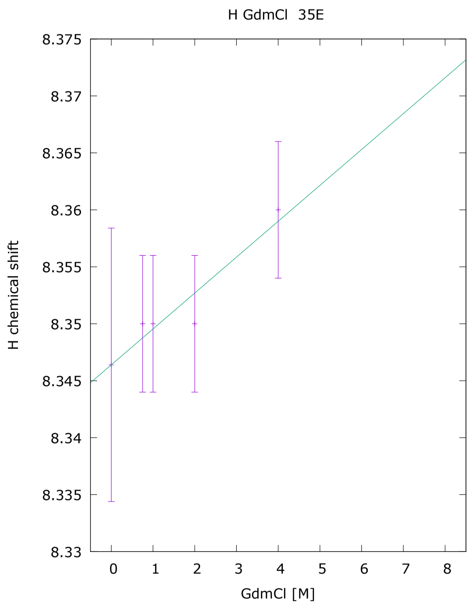
H GdmCl 35E
H chemical shift
GdmCl [M]

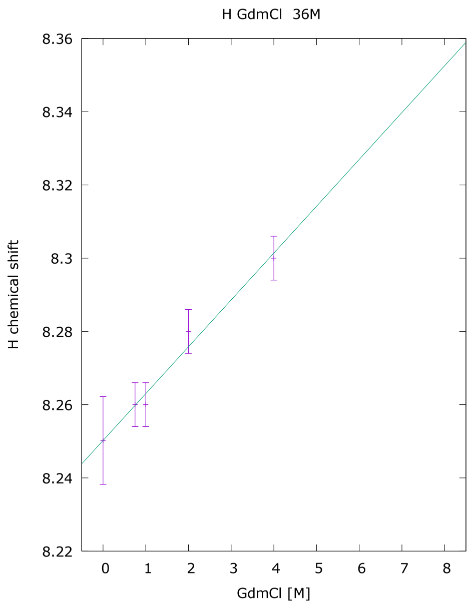
H GdmCl 36M
H chemical shift
8.36
8.34
8.32
8.3
8.28
8.26
8.24
8.22
0 1 2 3 4 5 6 7 8
GdmCl [M]

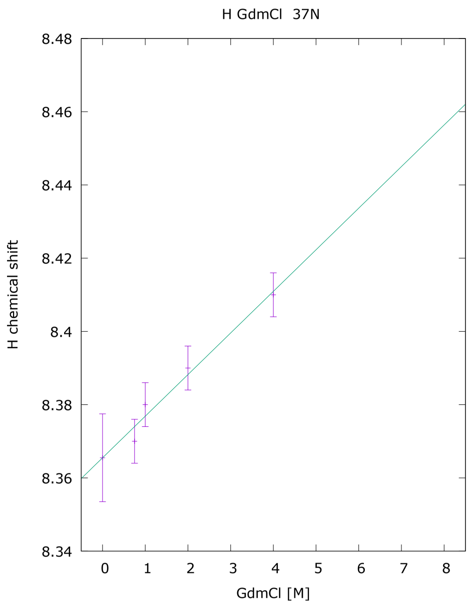
H GdmCl 37N
H chemical shift
8.48
8.46
8.44
8.42
8.4
8.38
8.36
8.34
0 1 2 3 4 5 6 7 8
GdmCl [M]

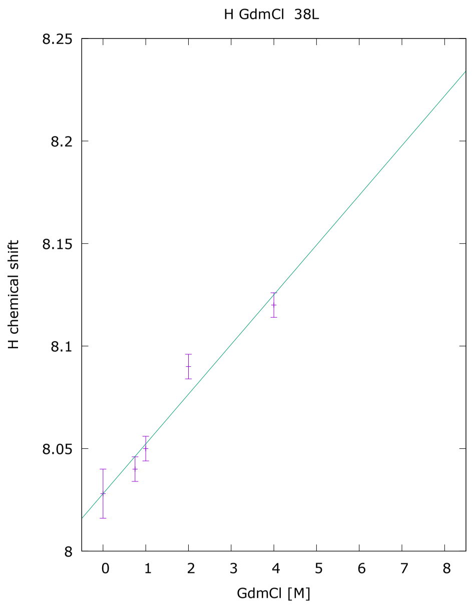
H GdmCl 38L
H chemical shift
8.25
8.2
8.15
8.1
8.05
8
0 1 2 3 4 5 6 7 8
GdmCl [M]

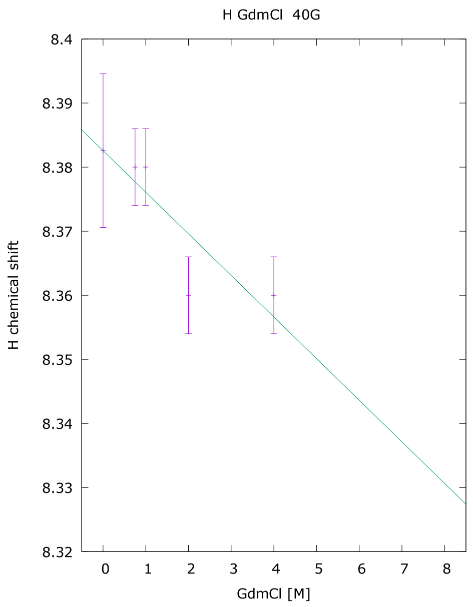
H GdmCl 40G
H chemical shift
8.4
8.39
8.38
8.37
8.36
8.35
8.34
8.33
8.32
0 1 2 3 4 5 6 7 8
GdmCl [M]

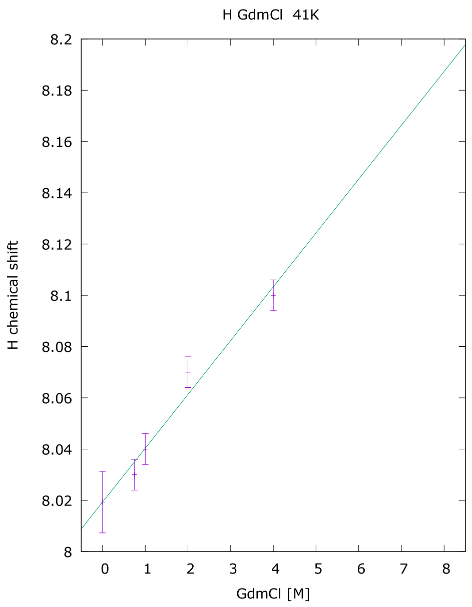
H GdmCl 41K
H chemical shift
8.2
8.18
8.16
8.14
8.12
8.1
8.08
8.06
8.04
8.02
8
0 1 2 3 4 5 6 7 8
GdmCl [M]

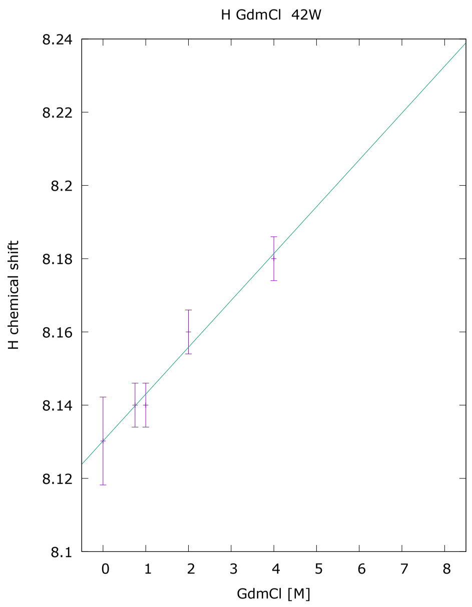
H GdmCl 42W
H chemical shift
8.24
8.22
8.2
8.18
8.16
8.14
8.12
8.1
0 1 2 3 4 5 6 7 8
GdmCl [M]

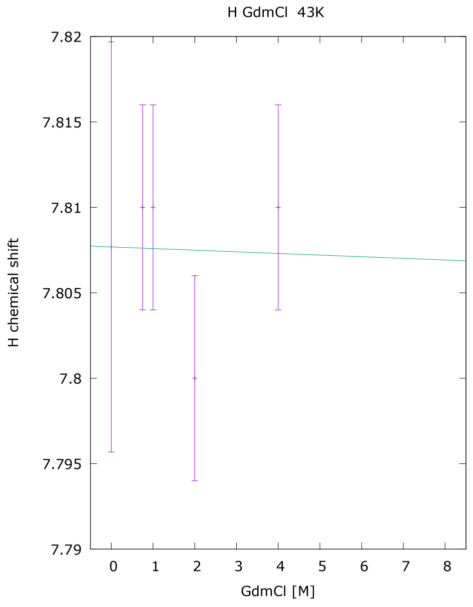
H GdmCl 43K
H chemical shift
GdmCl [M]

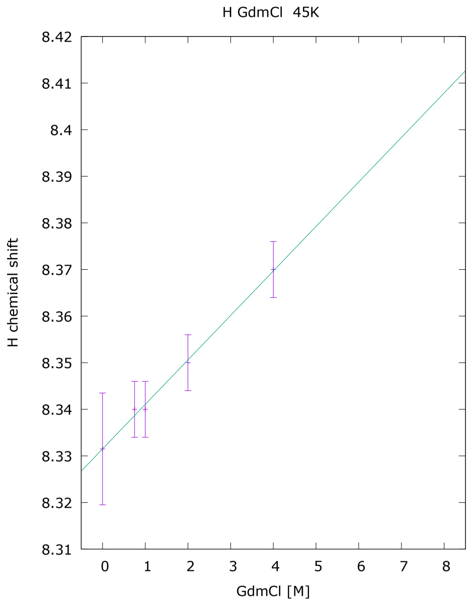
H GdmCl 45K
H chemical shift
GdmCl [M]

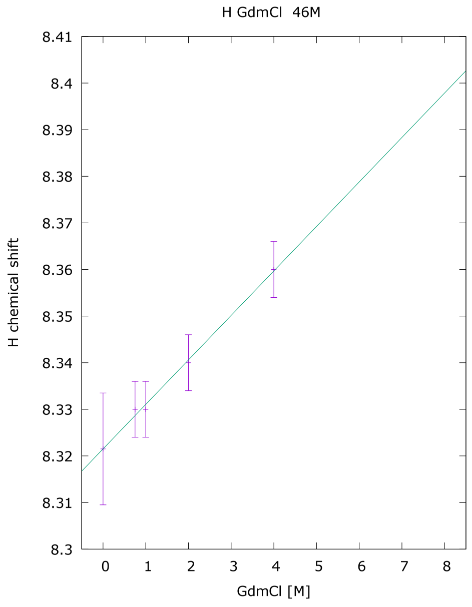
H GdmCl 46M
H chemical shift
GdmCl [M]

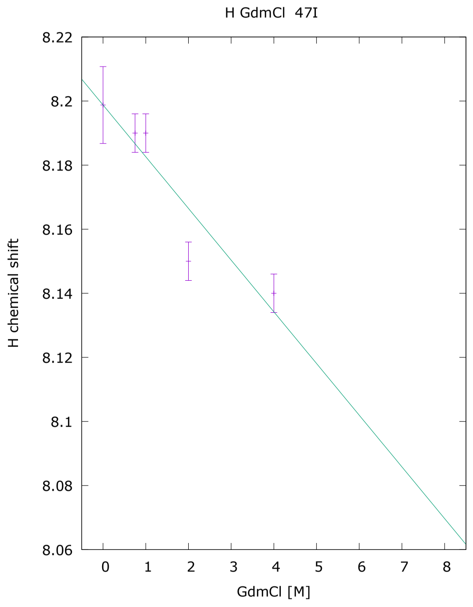
H GdmCl 47I
H chemical shift
GdmCl [M]

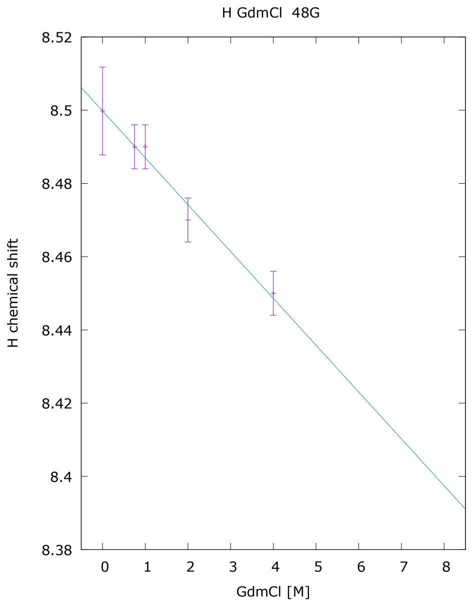
H GdmCl 48G
H chemical shift
GdmCl [M]

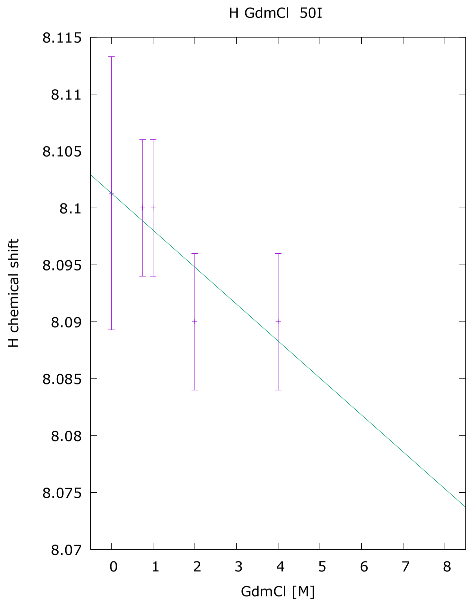
H GdmCl 50I
H chemical shift
GdmCl [M]

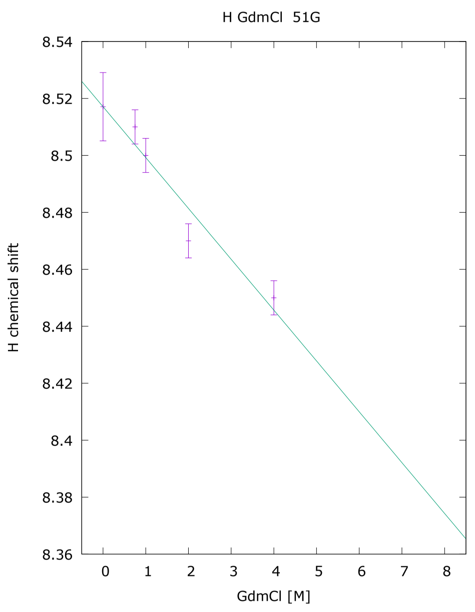
H GdmCl 51G
H chemical shift
GdmCl [M]

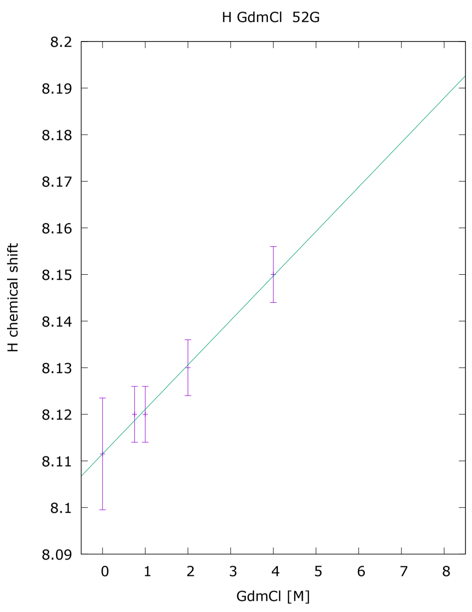
H GdmCl 52G
H chemical shift
GdmCl [M]

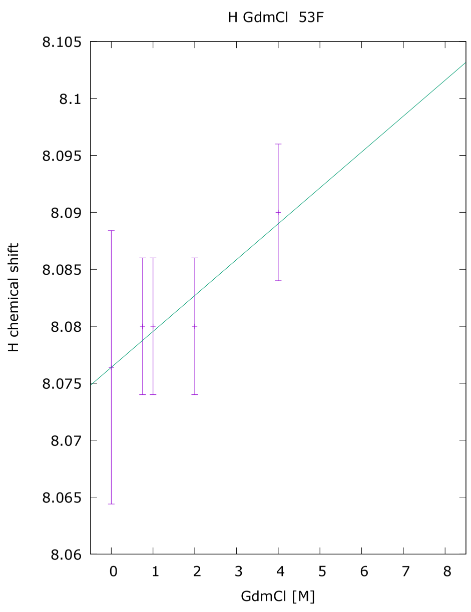
H GdmCl 53F
H chemical shift
GdmCl [M]

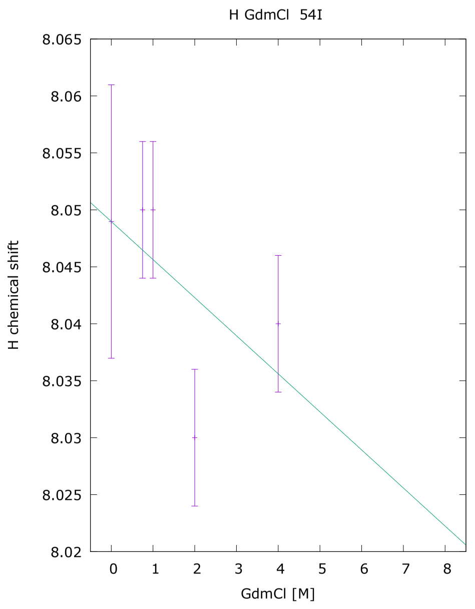
H GdmCl 54I
H chemical shift
GdmCl [M]

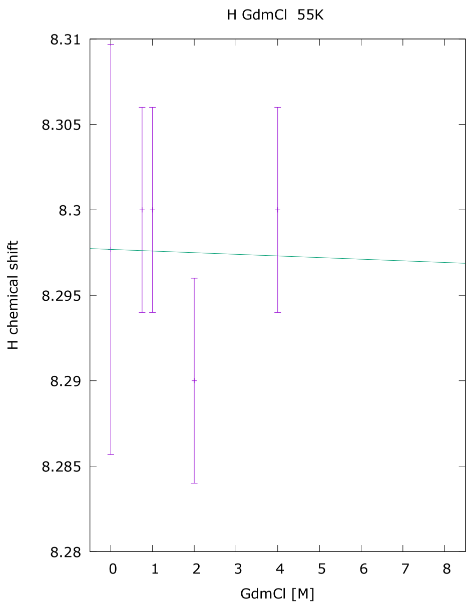
H GdmCl 55K
H chemical shift
GdmCl [M]

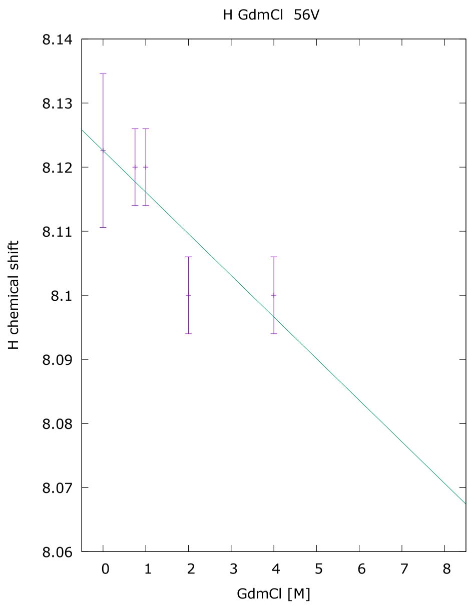
H GdmCl 56V
H chemical shift
GdmCl [M]

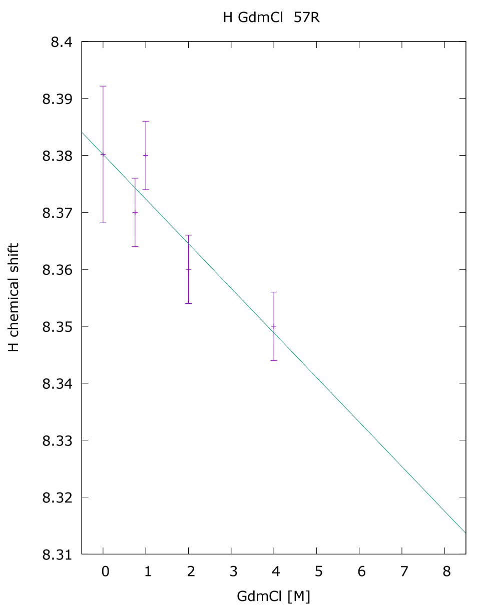
H GdmCl 57R
H chemical shift
GdmCl [M]
8.4
8.39
8.38
8.37
8.36
8.35
8.34
8.33
8.32
8.31
0 1 2 3 4 5 6 7 8

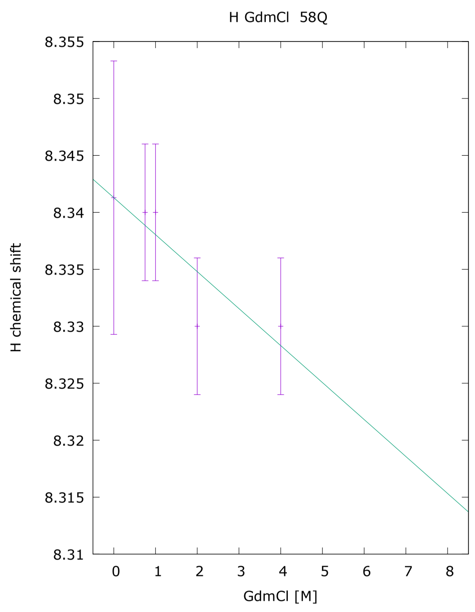
H GdmCl 58Q
H chemical shift
GdmCl [M]
8.355
8.35
8.345
8.34
8.335
8.33
8.325
8.32
8.315
8.31
0 1 2 3 4 5 6 7 8

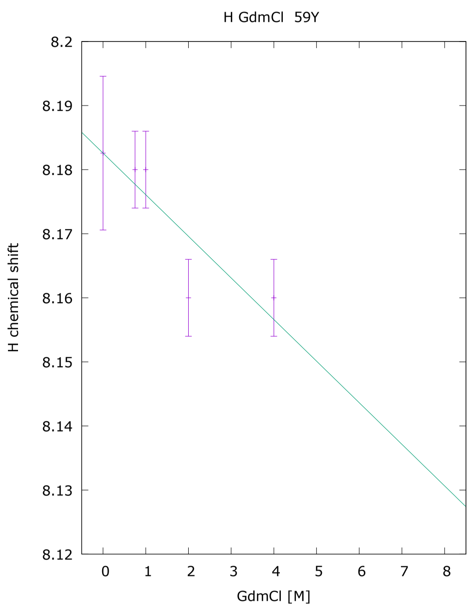
H GdmCl 59Y
H chemical shift
GdmCl [M]
8.2
8.19
8.18
8.17
8.16
8.15
8.14
8.13
8.12
0 1 2 3 4 5 6 7 8

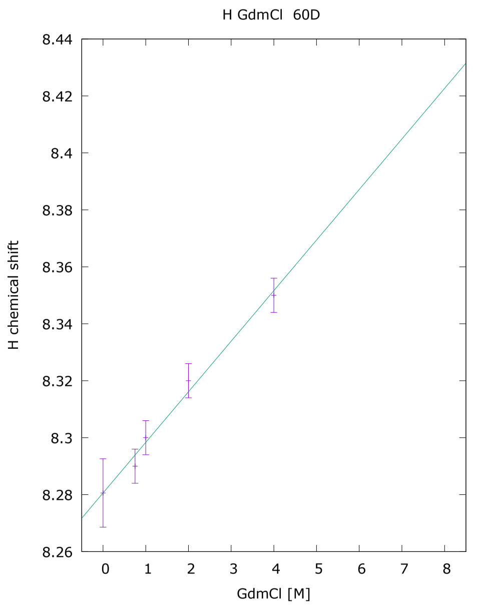
H GdmCl 60D
H chemical shift
GdmCl [M]
8.44
8.42
8.4
8.38
8.36
8.34
8.32
8.3
8.28
8.26
0 1 2 3 4 5 6 7 8

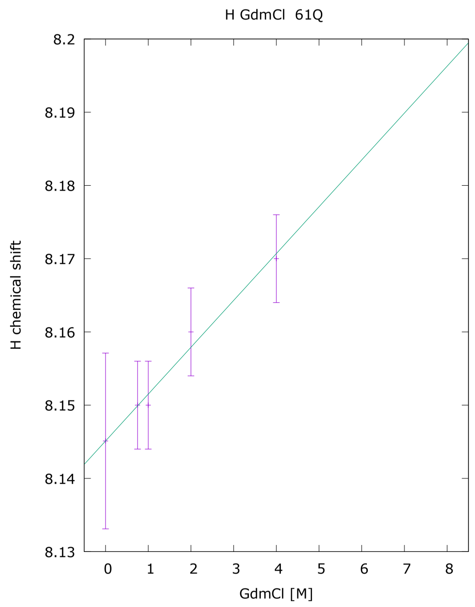
H GdmCl 61Q
H chemical shift
GdmCl [M]
8.2
8.19
8.18
8.17
8.16
8.15
8.14
8.13
0 1 2 3 4 5 6 7 8

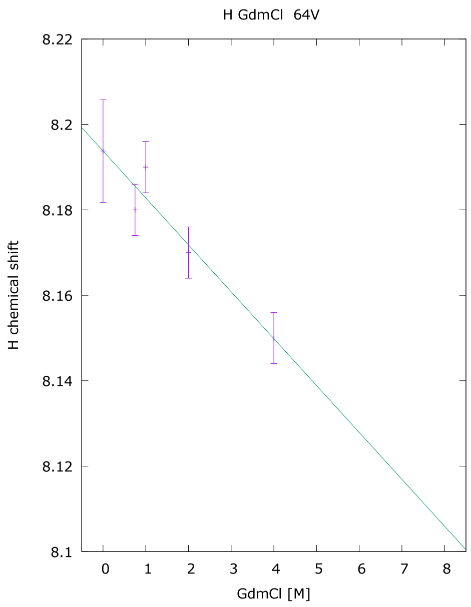
H GdmCl 64V
H chemical shift
GdmCl [M]
8.22
8.2
8.18
8.16
8.14
8.12
8.1
0 1 2 3 4 5 6 7 8

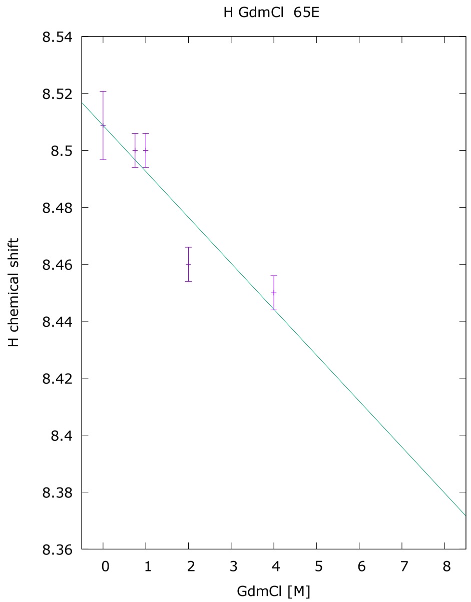
H GdmCl 65E
H chemical shift
GdmCl [M]
8.54
8.52
8.5
8.48
8.46
8.44
8.42
8.4
8.38
8.36
0 1 2 3 4 5 6 7 8
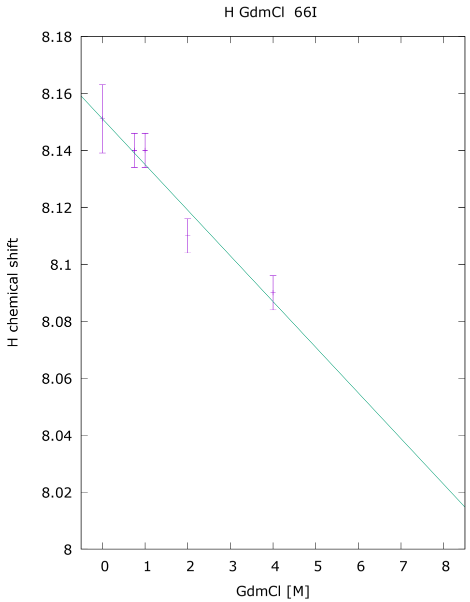
H GdmCl 66I
H chemical shift
GdmCl [M]
8.18
8.16
8.14
8.12
8.1
8.08
8.06
8.04
8.02
8
0 1 2 3 4 5 6 7 8
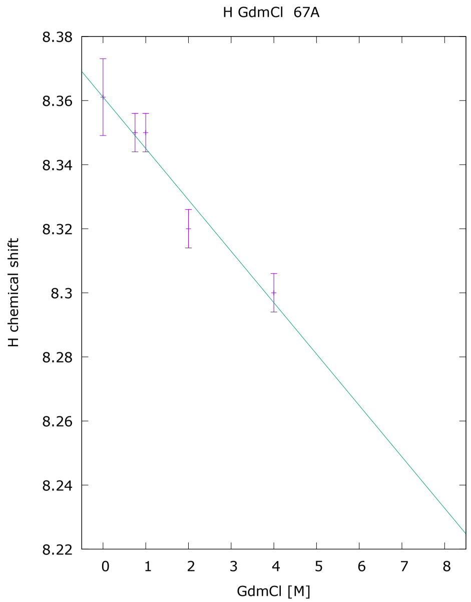
H GdmCl 67A
H chemical shift
GdmCl [M]
8.38
8.36
8.34
8.32
8.3
8.28
8.26
8.24
8.22
0 1 2 3 4 5 6 7 8
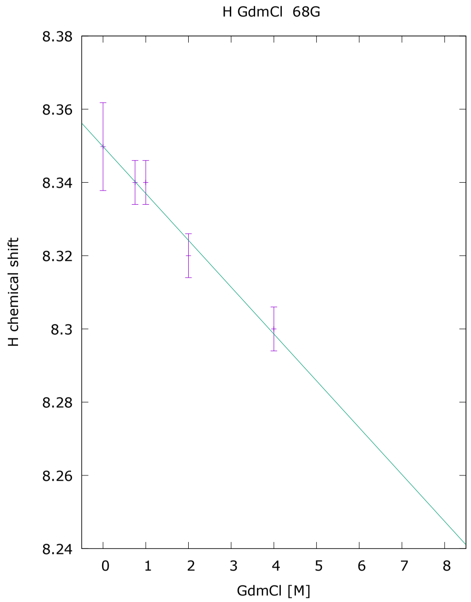
H GdmCl 68G
H chemical shift
GdmCl [M]
8.38
8.36
8.34
8.32
8.3
8.28
8.26
8.24
0 1 2 3 4 5 6 7 8
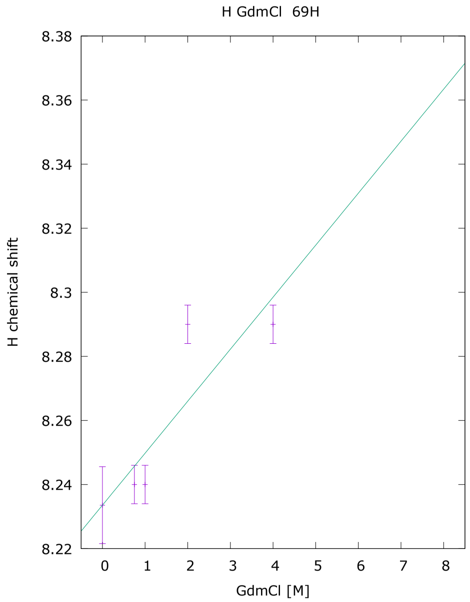
H GdmCl 69H
H chemical shift
GdmCl [M]
8.38
8.36
8.34
8.32
8.3
8.28
8.26
8.24
8.22
0 1 2 3 4 5 6 7 8
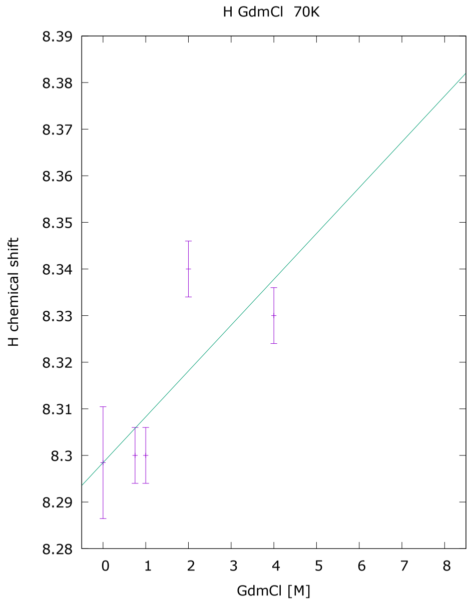
H GdmCl 70K
H chemical shift
GdmCl [M]
8.39
8.38
8.37
8.36
8.35
8.34
8.33
8.32
8.31
8.3
8.29
8.28
0 1 2 3 4 5 6 7 8

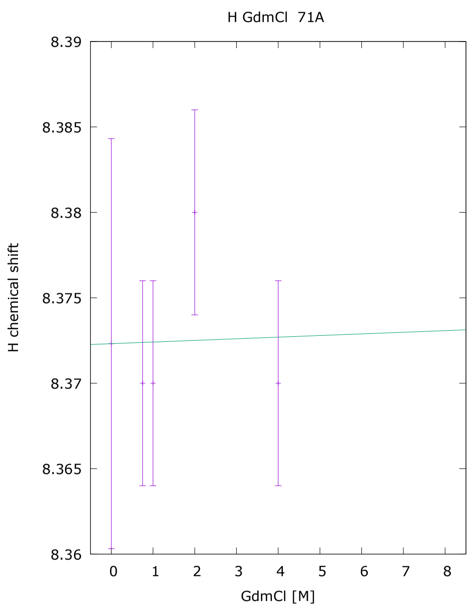
H GdmCl 71A
H chemical shift
GdmCl [M]

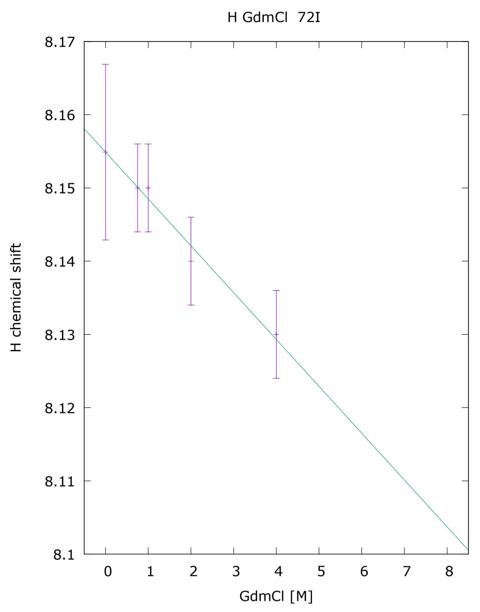
H GdmCl 72I
H chemical shift
GdmCl [M]

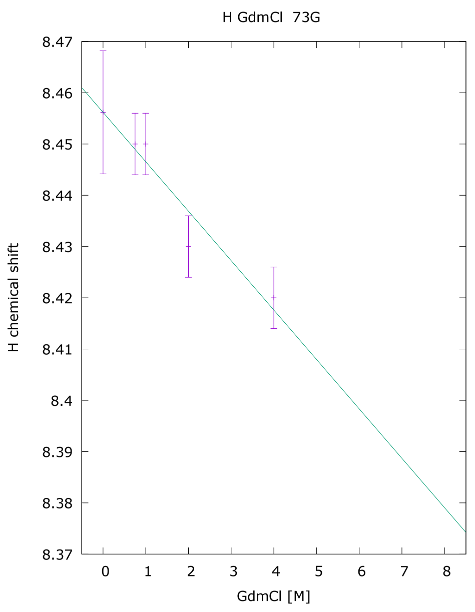
H GdmCl 73G
H chemical shift
GdmCl [M]

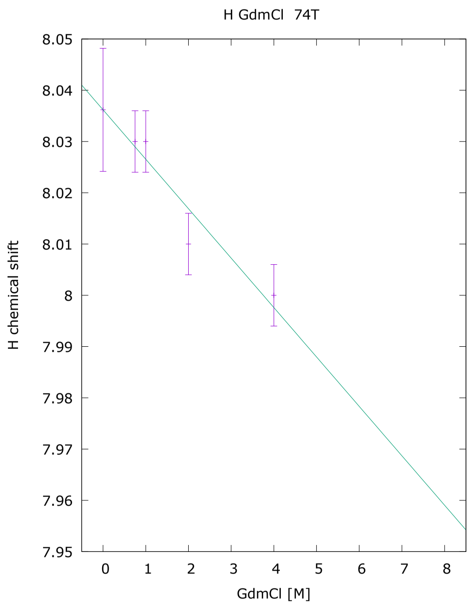
H GdmCl 74T
H chemical shift
GdmCl [M]

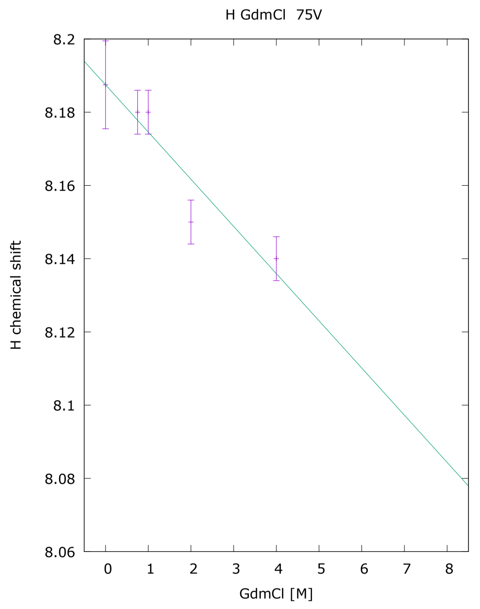
H GdmCl 75V
H chemical shift
GdmCl [M]

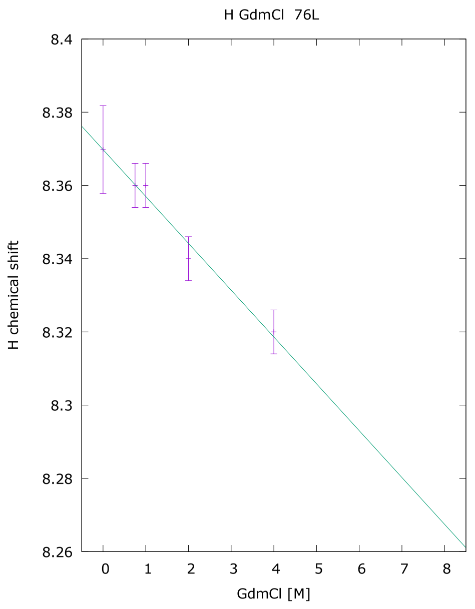
H GdmCl 76L
H chemical shift
GdmCl [M]

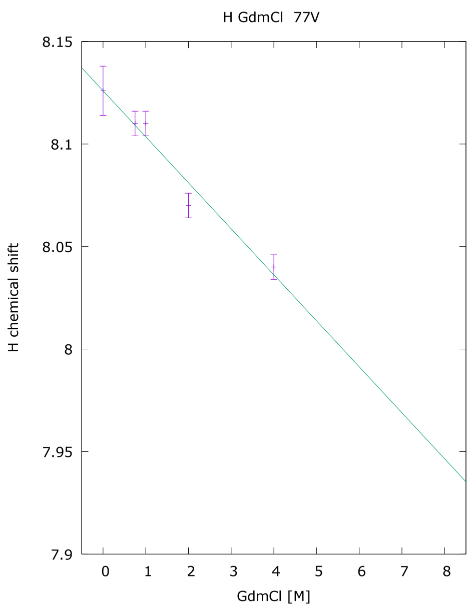
H GdmCl 77V
H chemical shift
GdmCl [M]

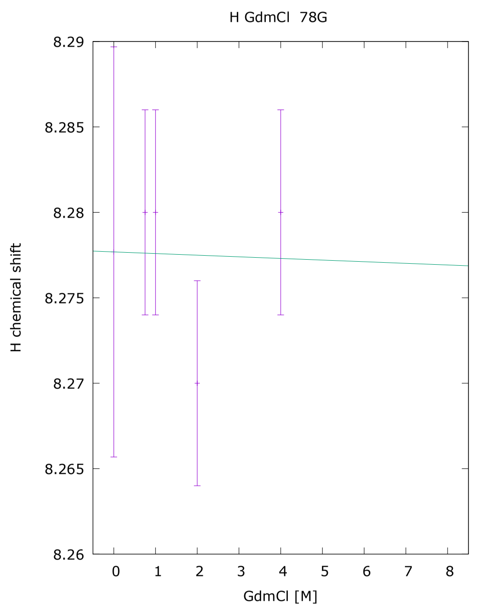
H GdmCl 78G
H chemical shift
GdmCl [M]

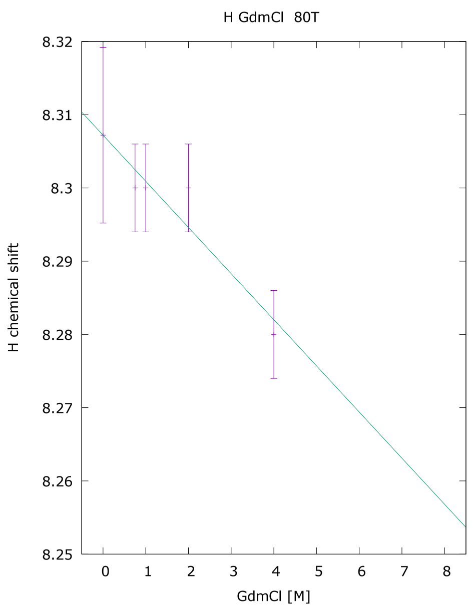
H GdmCl 80T
H chemical shift
GdmCl [M]

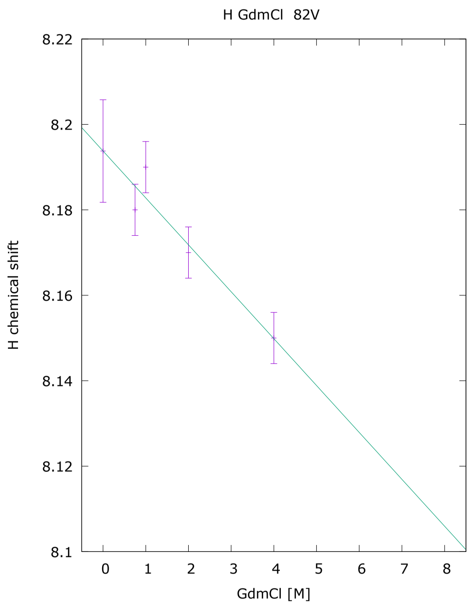
H GdmCl 82V
H chemical shift
GdmCl [M]

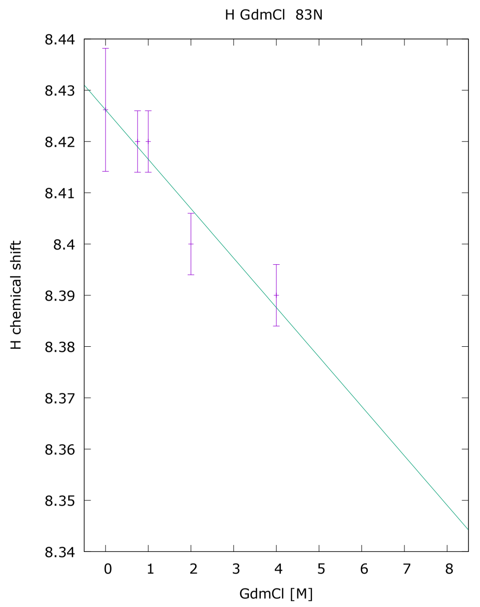
H GdmCl 83N
H chemical shift
GdmCl [M]

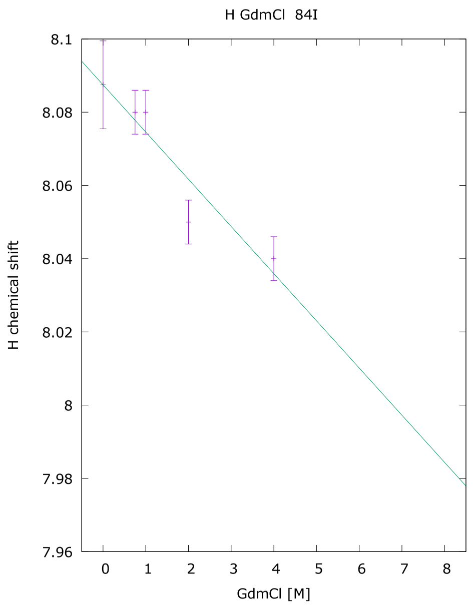
H GdmCl 84I
H chemical shift
GdmCl [M]

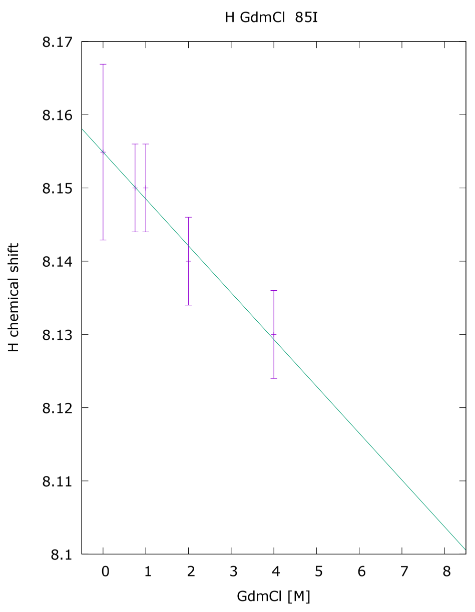
H GdmCl 85I
H chemical shift
8.17
8.16
8.15
8.14
8.13
8.12
8.11
8.1
0 1 2 3 4 5 6 7 8
GdmCl [M]

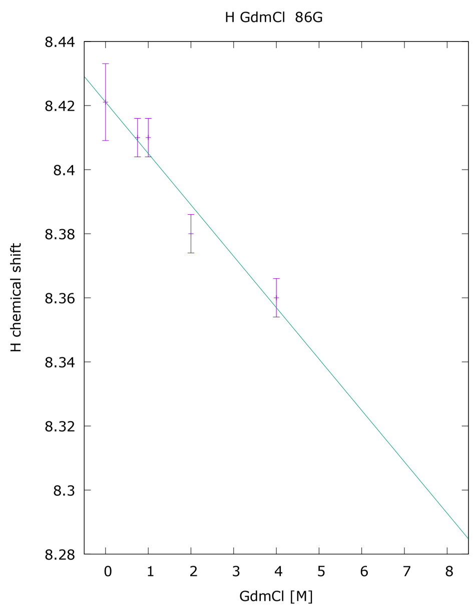
H GdmCl 86G
H chemical shift
8.44
8.42
8.4
8.38
8.36
8.34
8.32
8.3
8.28
0 1 2 3 4 5 6 7 8
GdmCl [M]

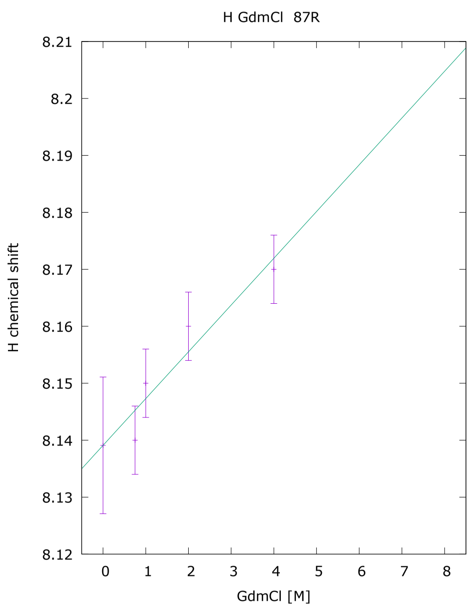
H GdmCl 87R
H chemical shift
8.21
8.2
8.19
8.18
8.17
8.16
8.15
8.14
8.13
8.12
0 1 2 3 4 5 6 7 8
GdmCl [M]

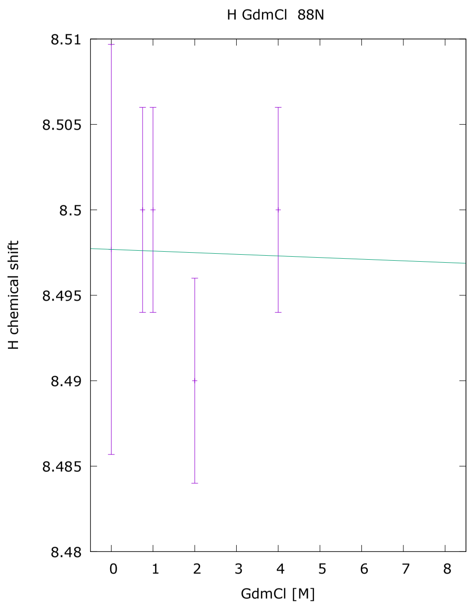
H GdmCl 88N
H chemical shift
8.51
8.505
8.5
8.495
8.49
8.485
8.48
0 1 2 3 4 5 6 7 8
GdmCl [M]

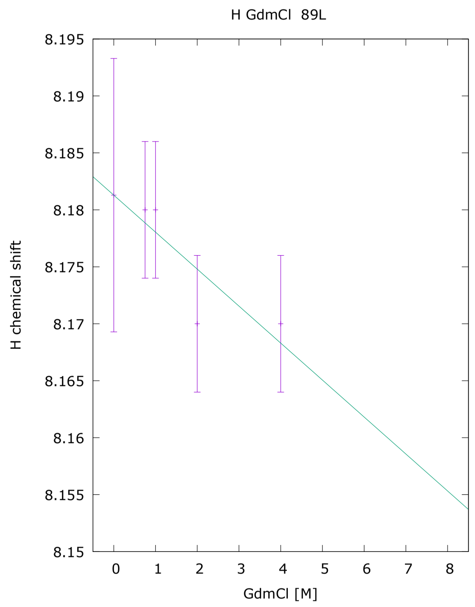
H GdmCl 89L
H chemical shift
8.195
8.19
8.185
8.18
8.175
8.17
8.165
8.16
8.155
8.15
0 1 2 3 4 5 6 7 8
GdmCl [M]

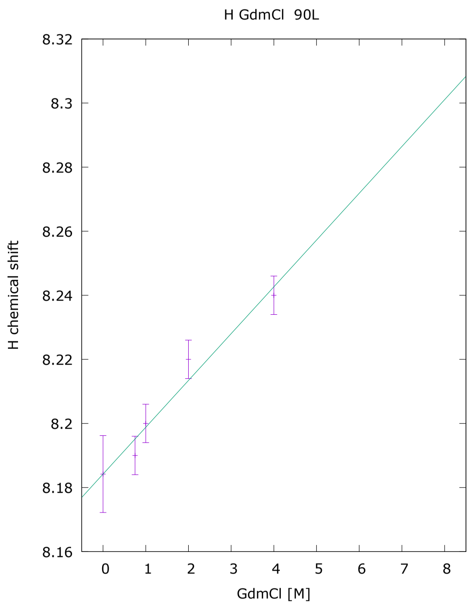
H GdmCl 90L
H chemical shift
8.32
8.3
8.28
8.26
8.24
8.22
8.2
8.18
8.16
0 1 2 3 4 5 6 7 8
GdmCl [M]

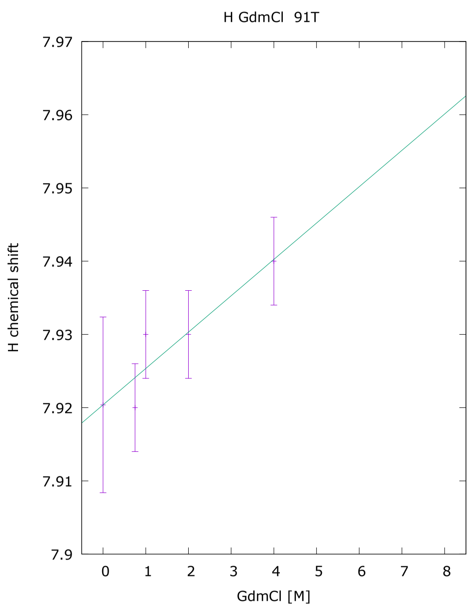
H GdmCl 91T
H chemical shift
GdmCl [M]

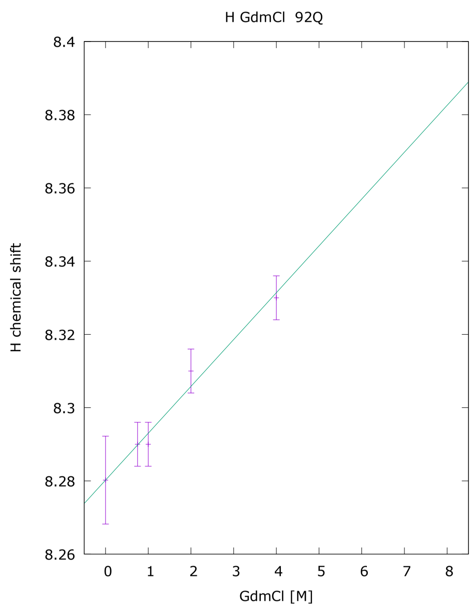
H GdmCl 92Q
H chemical shift
GdmCl [M]

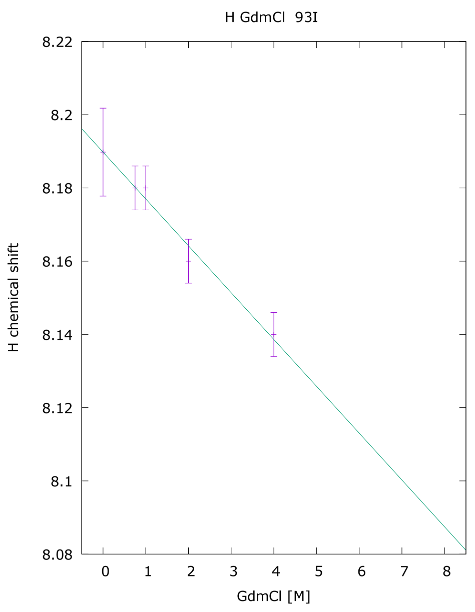
H GdmCl 93I
H chemical shift
GdmCl [M]

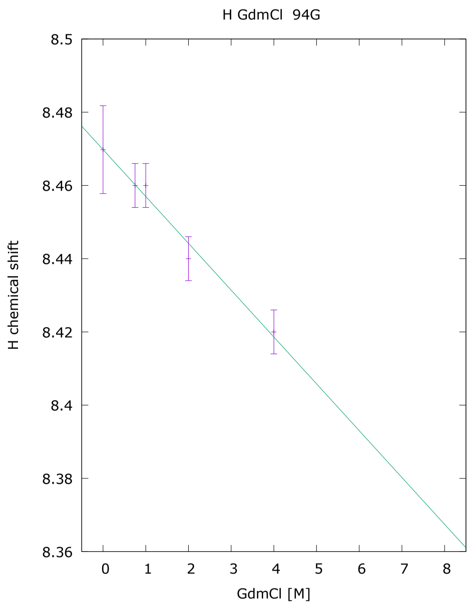
H GdmCl 94G
H chemical shift
GdmCl [M]

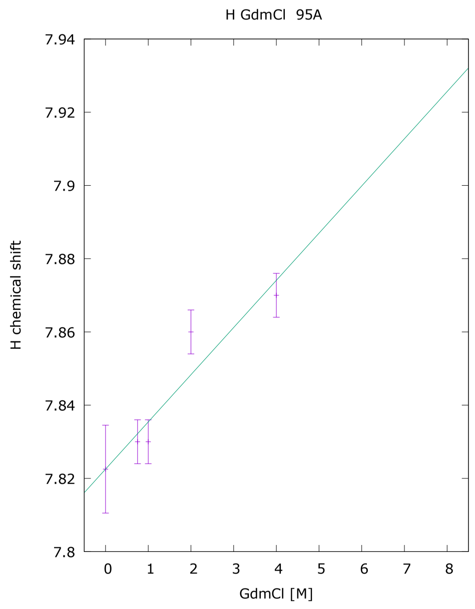
H GdmCl 95A
H chemical shift
GdmCl [M]

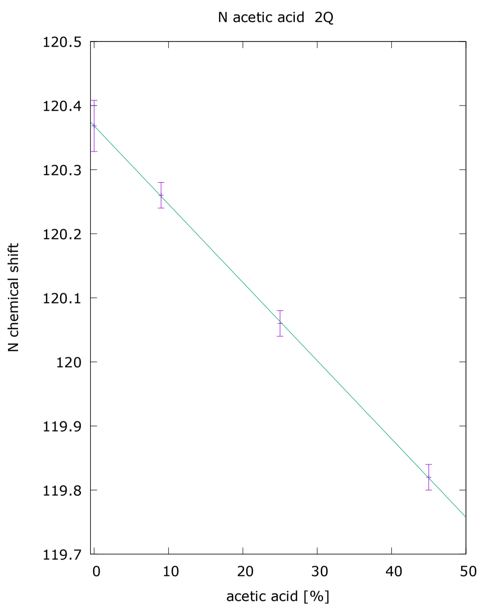
N acetic acid 2Q
N chemical shift
acetic acid [%]

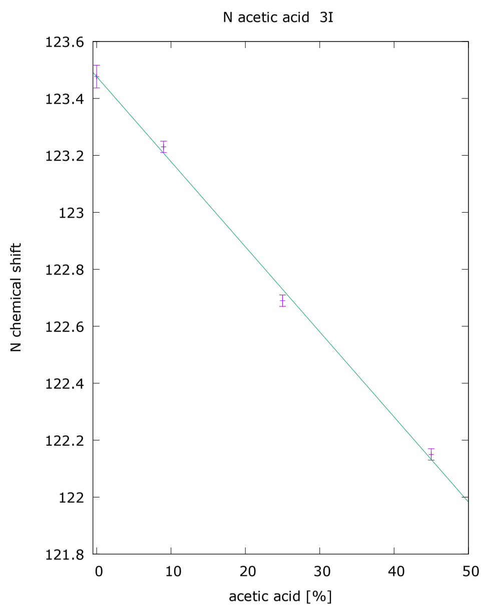
N acetic acid 3I
N chemical shift
acetic acid [%]

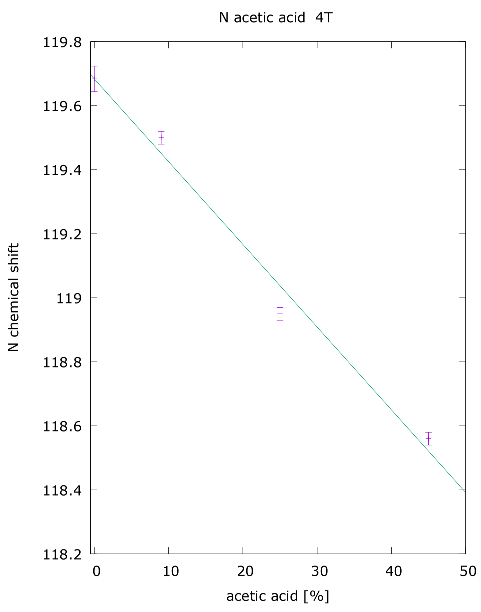
N acetic acid 4T
N chemical shift
acetic acid [%]

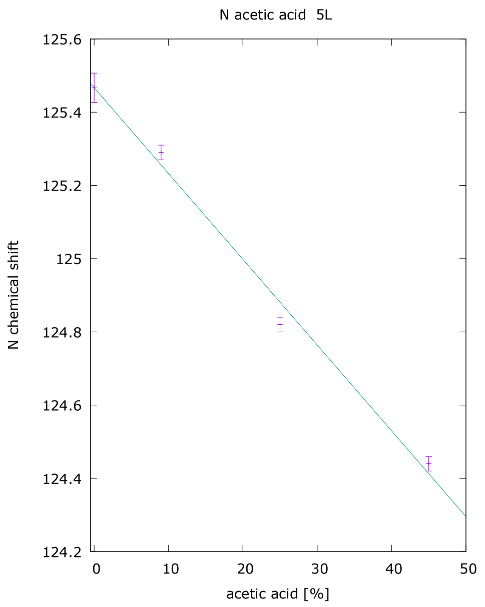
N acetic acid 5L
N chemical shift
acetic acid [%]

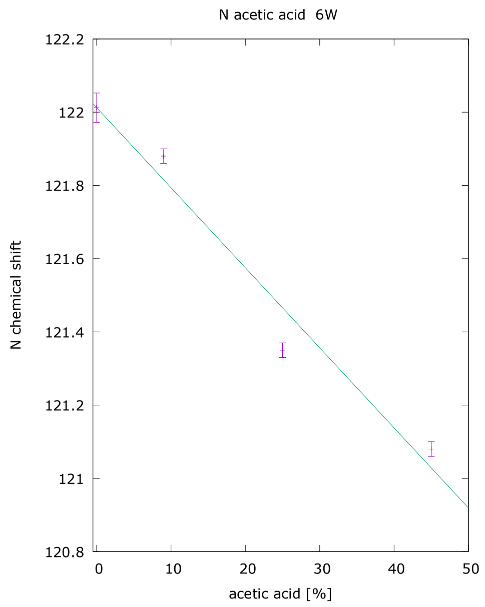
N acetic acid 6W
N chemical shift
acetic acid [%]

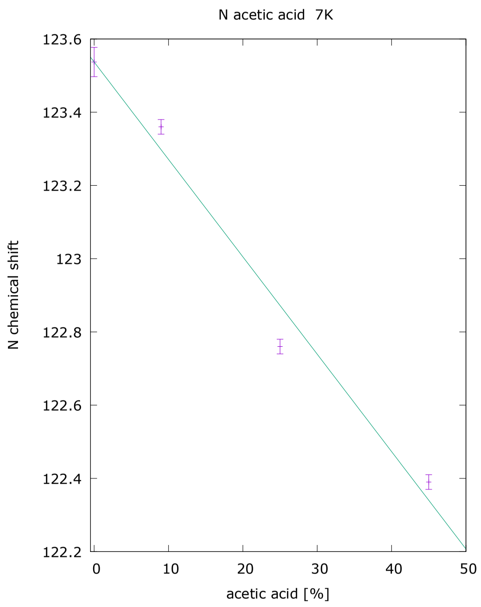
N acetic acid 7K
N chemical shift
acetic acid [%]

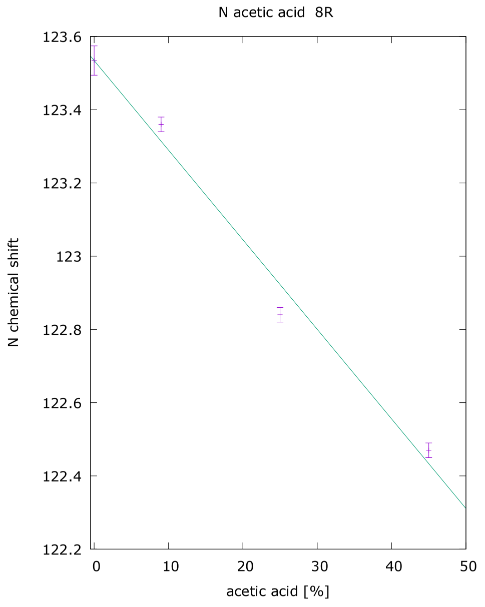
N acetic acid 8R
N chemical shift
123.6
123.4
123.2
123
122.8
122.6
122.4
122.2
0
10
20
30
40
50
acetic acid [%]

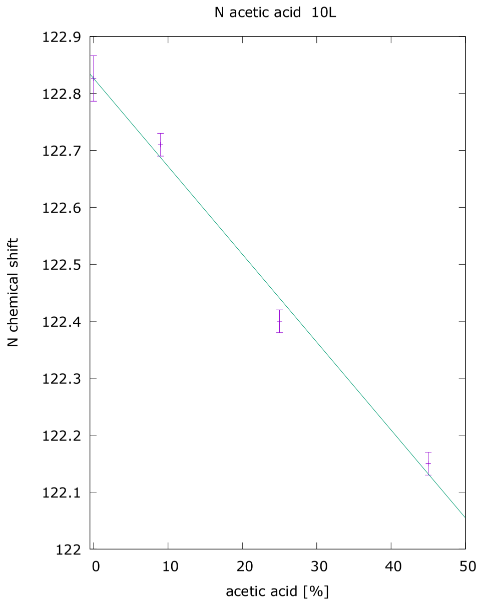
N acetic acid 10L
N chemical shift
122.9
122.8
122.7
122.6
122.5
122.4
122.3
122.2
122.1
122
0
10
20
30
40
50
acetic acid [%]

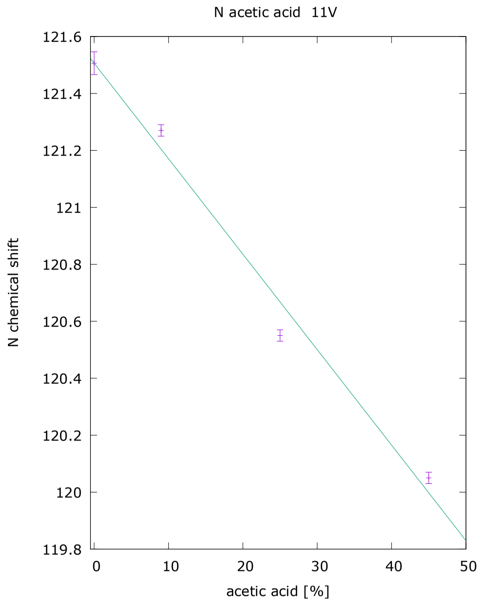
N acetic acid 11V
N chemical shift
121.6
121.4
121.2
121
120.8
120.6
120.4
120.2
120
119.8
0
10
20
30
40
50
acetic acid [%]

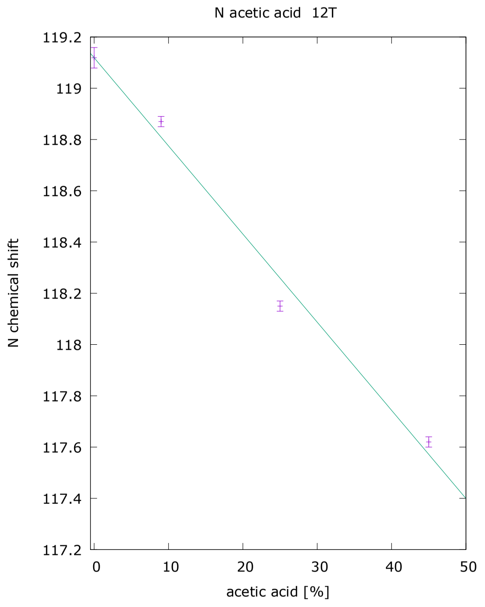
N acetic acid 12T
N chemical shift
119.2
119
118.8
118.6
118.4
118.2
118
117.8
117.6
117.4
117.2
0
10
20
30
40
50
acetic acid [%]

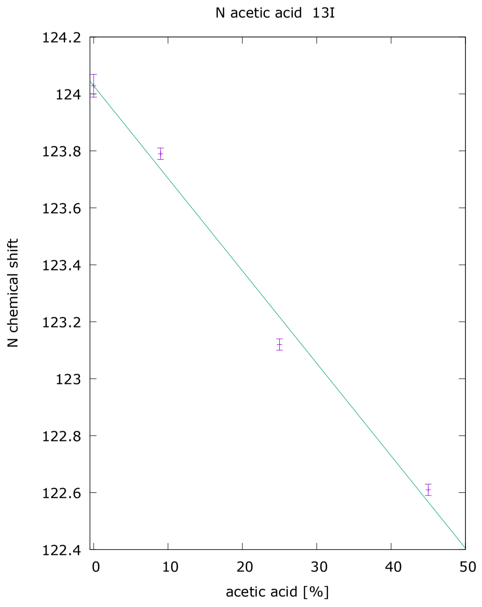
N acetic acid 13I
N chemical shift
124.2
124
123.8
123.6
123.4
123.2
123
122.8
122.6
122.4
0
10
20
30
40
50
acetic acid [%]

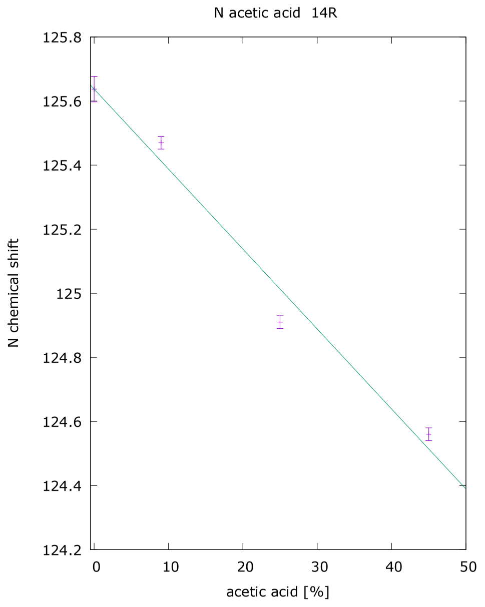
N acetic acid 14R
N chemical shift
125.8
125.6
125.4
125.2
125
124.8
124.6
124.4
124.2
0
10
20
30
40
50
acetic acid [%]

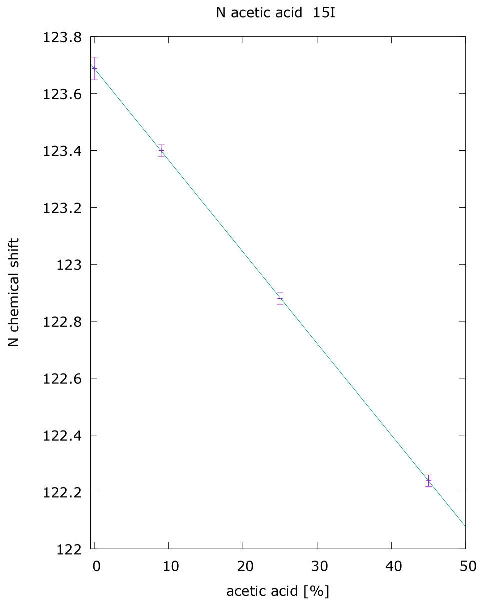
N acetic acid 15I
N chemical shift
acetic acid [%]

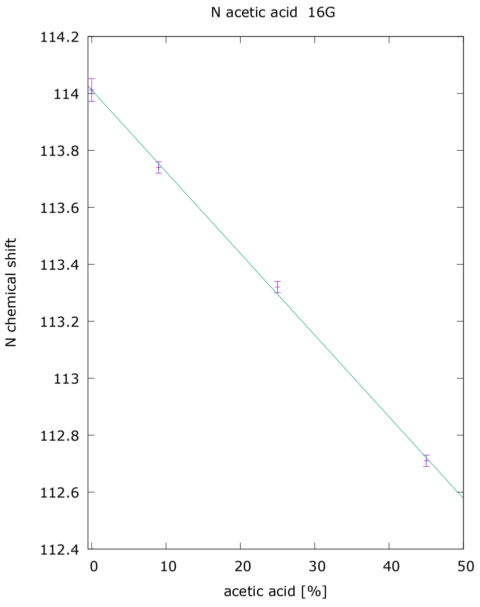
N acetic acid 16G
N chemical shift
acetic acid [%]

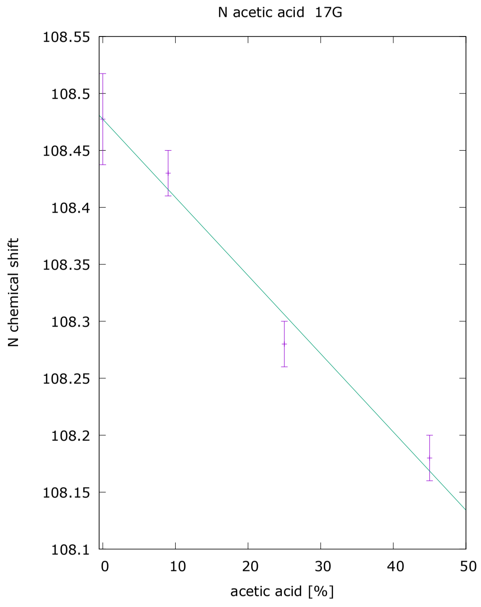
N acetic acid 17G
N chemical shift
acetic acid [%]

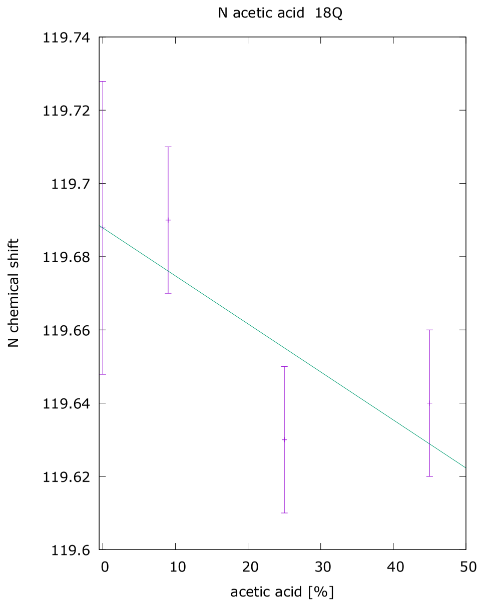
N acetic acid 18Q
N chemical shift
acetic acid [%]

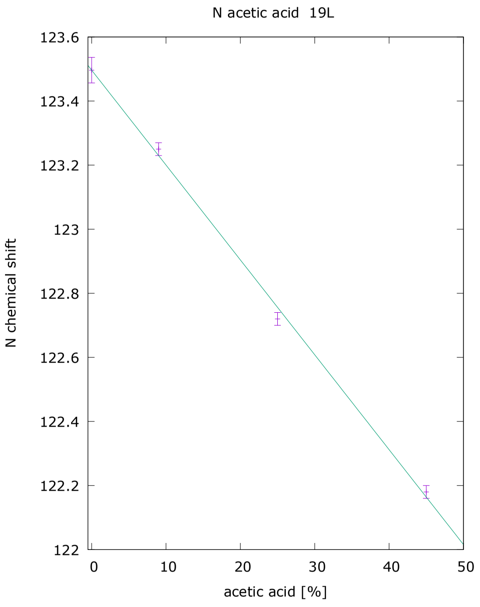
N acetic acid 19L
N chemical shift
acetic acid [%]

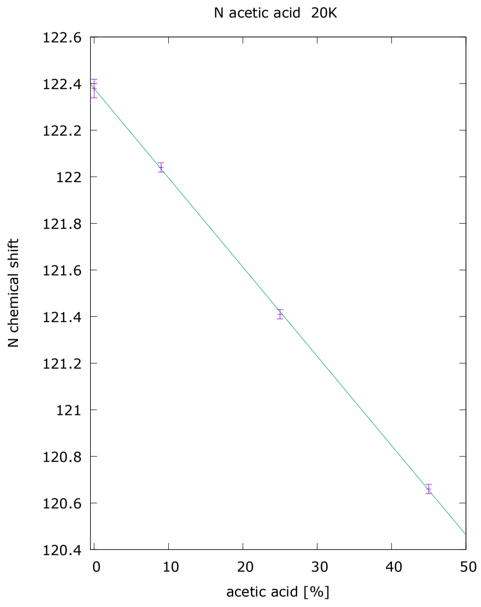
N acetic acid 20K
N chemical shift
acetic acid [%]

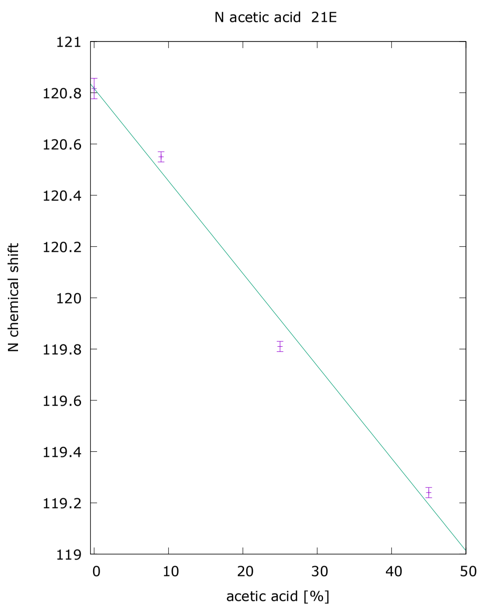
N acetic acid 21E
N chemical shift
acetic acid [%]
121
120.8
120.6
120.4
120.2
120
119.8
119.6
119.4
119.2
119
0
10
20
30
40
50

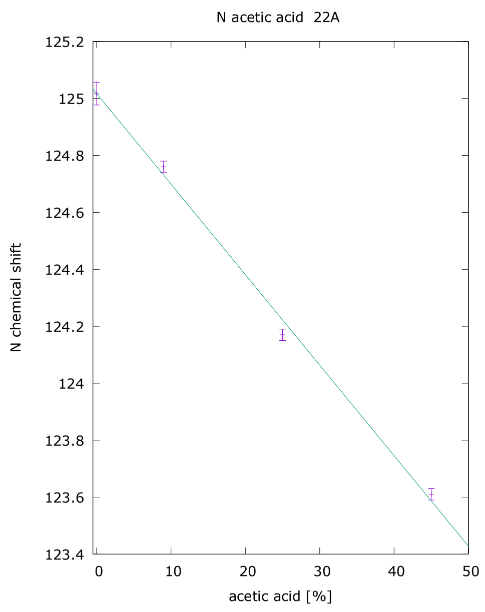
N acetic acid 22A
N chemical shift
acetic acid [%]
125.2
125
124.8
124.6
124.4
124.2
124
123.8
123.6
123.4
0
10
20
30
40
50

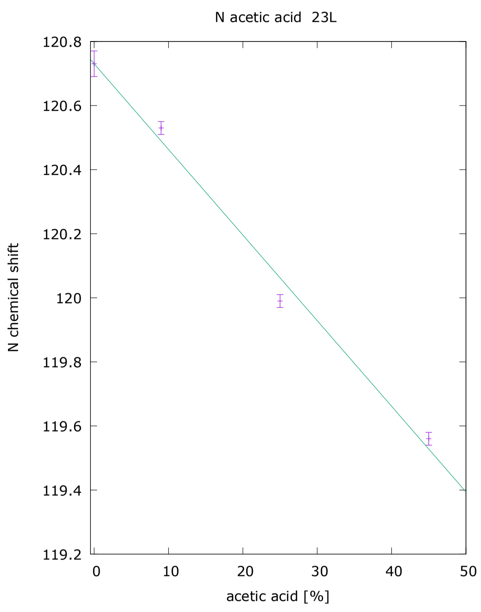
N acetic acid 23L
N chemical shift
acetic acid [%]
120.8
120.6
120.4
120.2
120
119.8
119.6
119.4
119.2
0
10
20
30
40
50

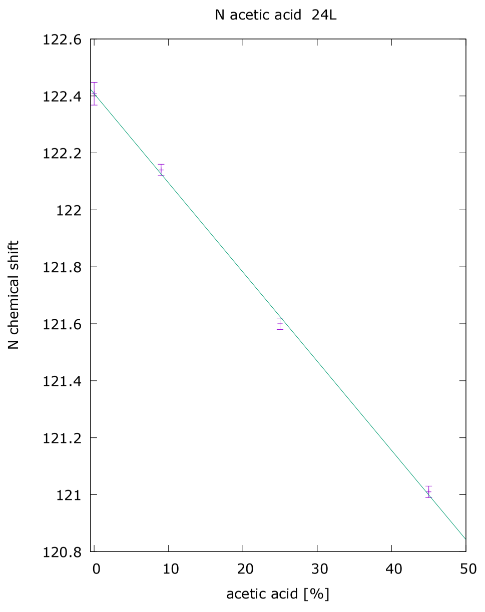
N acetic acid 24L
N chemical shift
acetic acid [%]
122.6
122.4
122.2
122
121.8
121.6
121.4
121.2
121
120.8
0
10
20
30
40
50

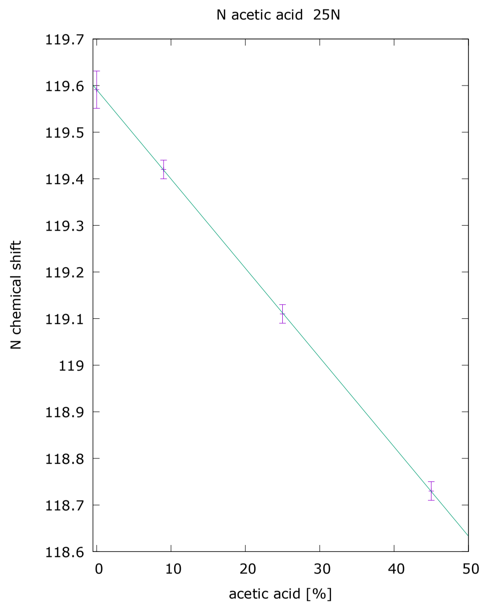
N acetic acid 25N
N chemical shift
acetic acid [%]
119.7
119.6
119.5
119.4
119.3
119.2
119.1
119
118.9
118.8
118.7
118.6
0
10
20
30
40
50

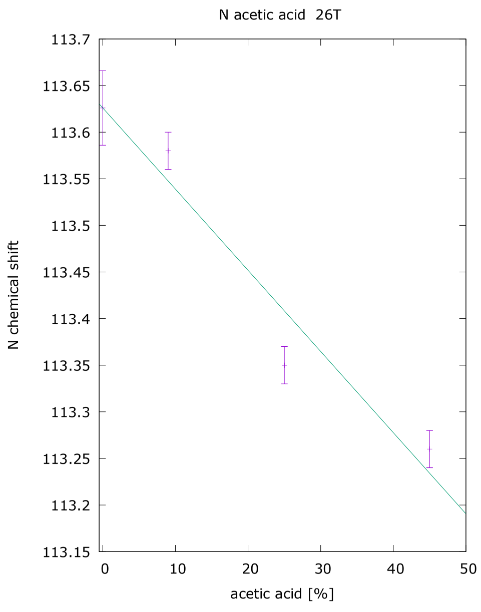
N acetic acid 26T
N chemical shift
acetic acid [%]
113.7
113.65
113.6
113.55
113.5
113.45
113.4
113.35
113.3
113.25
113.2
113.15
0
10
20
30
40
50

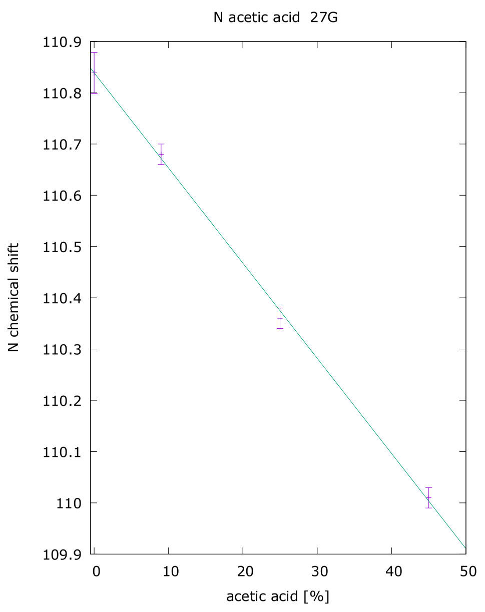
N acetic acid 27G
N chemical shift
acetic acid [%]

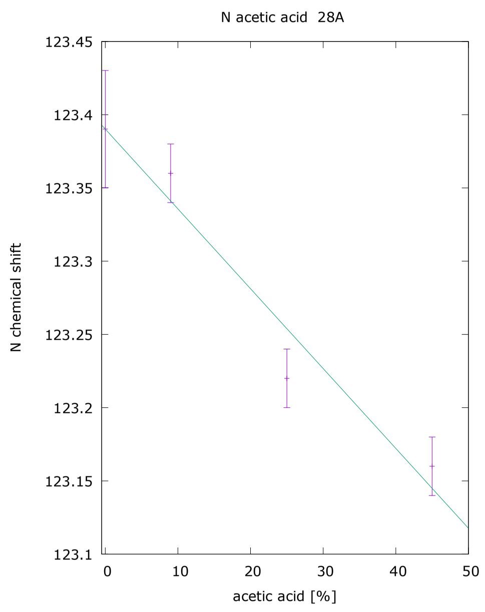
N acetic acid 28A
N chemical shift
acetic acid [%]

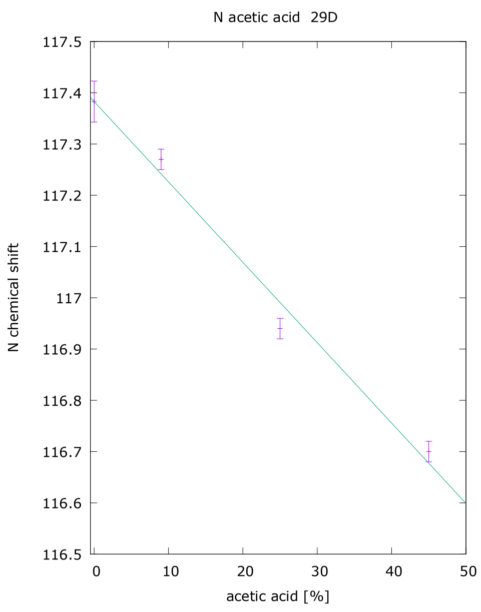
N acetic acid 29D
N chemical shift
acetic acid [%]

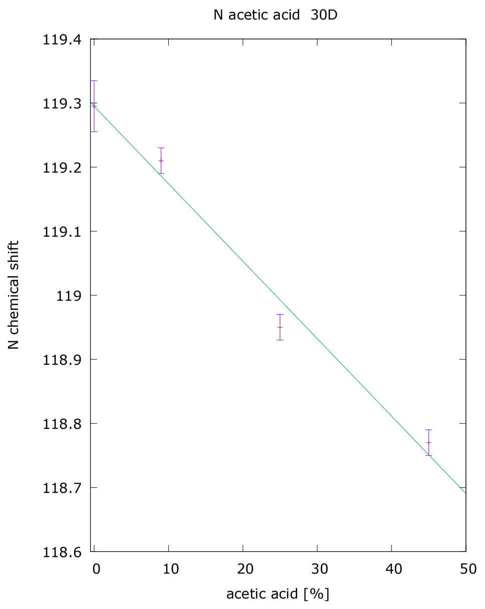
N acetic acid 30D
N chemical shift
acetic acid [%]

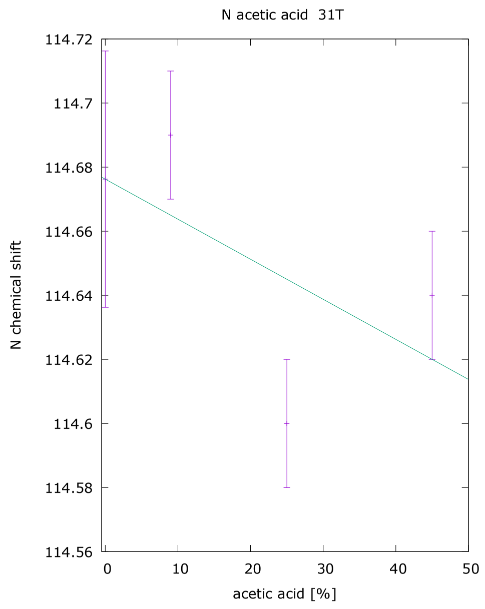
N acetic acid 31T
N chemical shift
acetic acid [%]

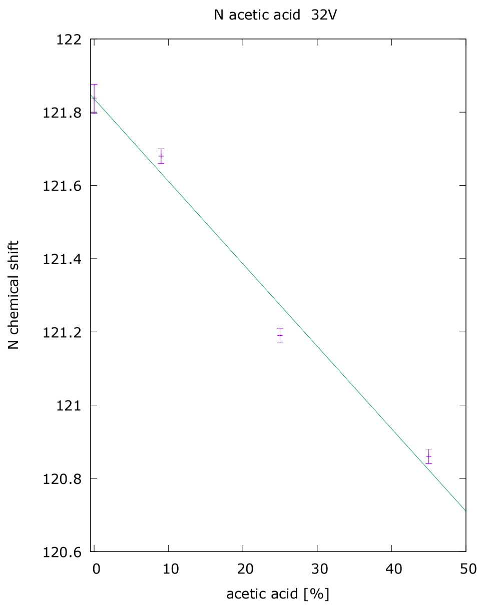
N acetic acid 32V
N chemical shift
acetic acid [%]

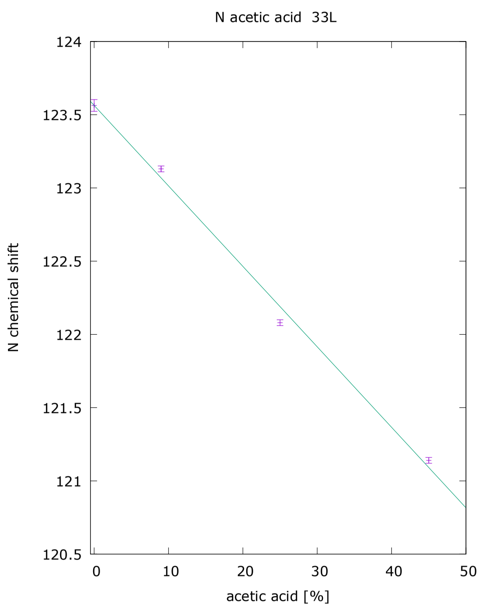
N acetic acid 33L
N chemical shift
acetic acid [%]

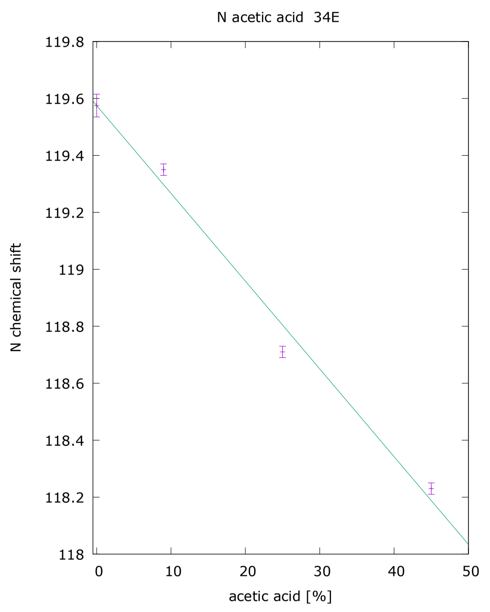
N acetic acid 34E
N chemical shift
acetic acid [%]

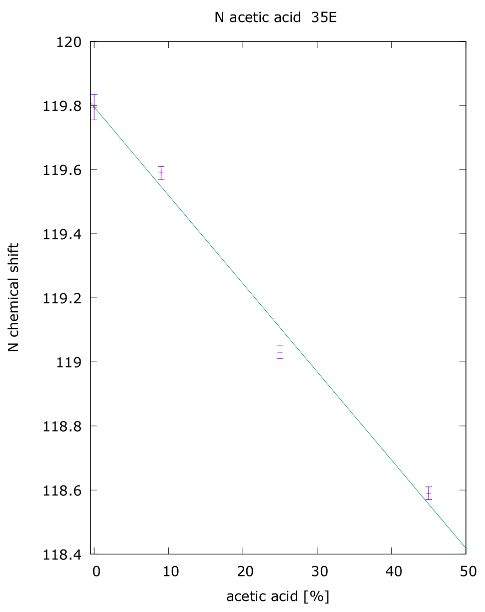
N acetic acid 35E
N chemical shift
acetic acid [%]

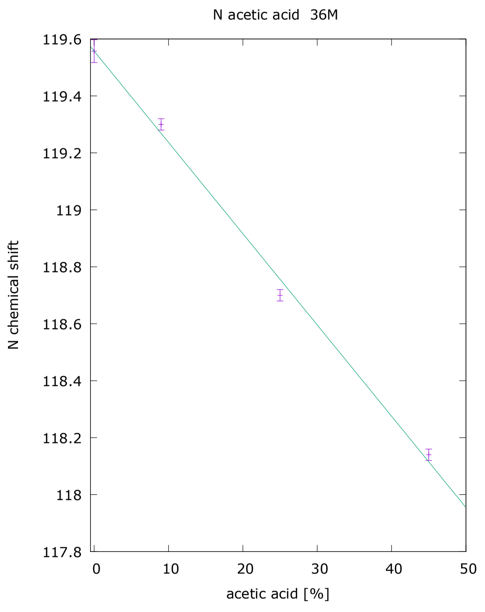
N acetic acid 36M
N chemical shift
acetic acid [%]

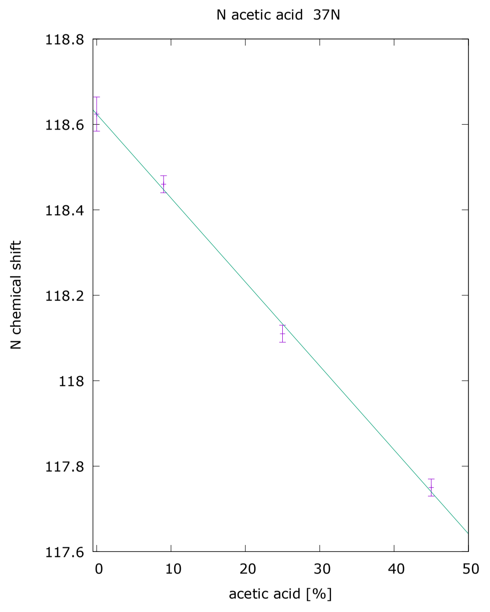
N acetic acid 37N
N chemical shift
acetic acid [%]

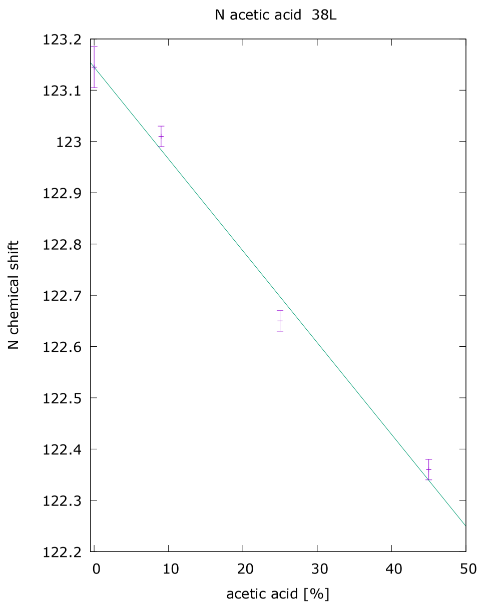
N acetic acid 38L
N chemical shift
acetic acid [%]

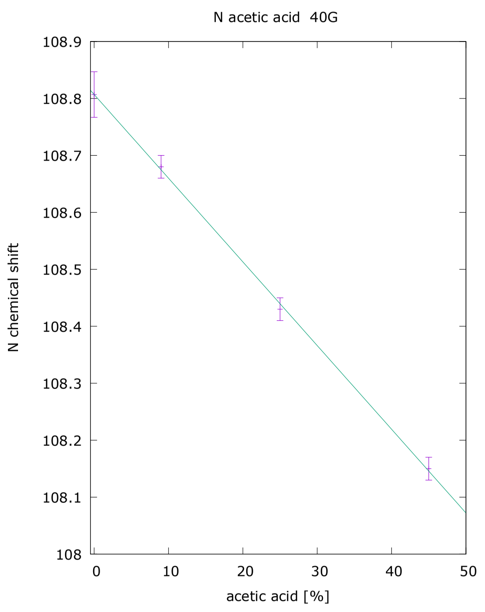
N acetic acid 40G
N chemical shift
acetic acid [%]

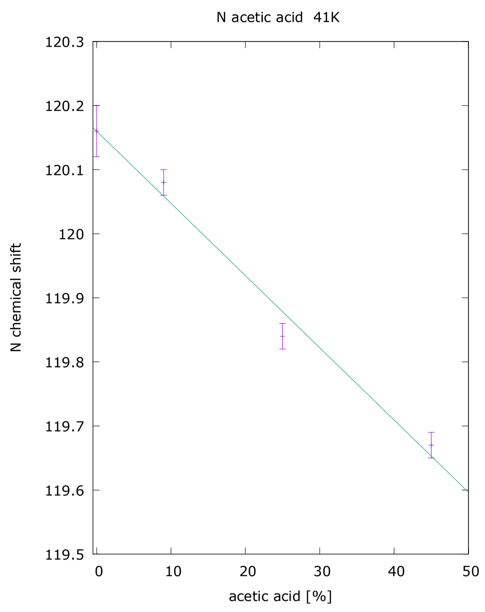
N acetic acid 41K
N chemical shift
acetic acid [%]

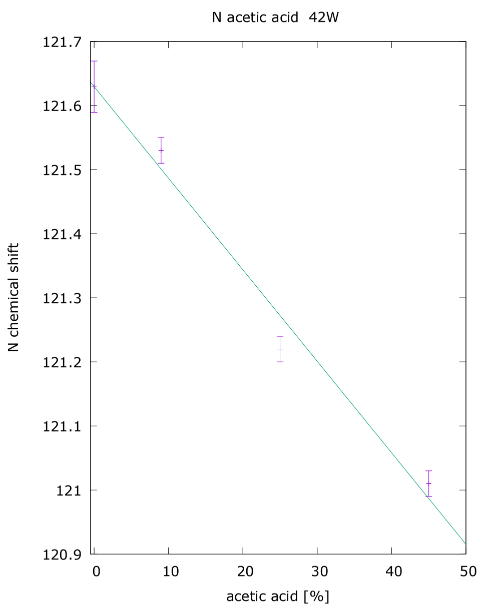
N acetic acid 42W
N chemical shift
acetic acid [%]

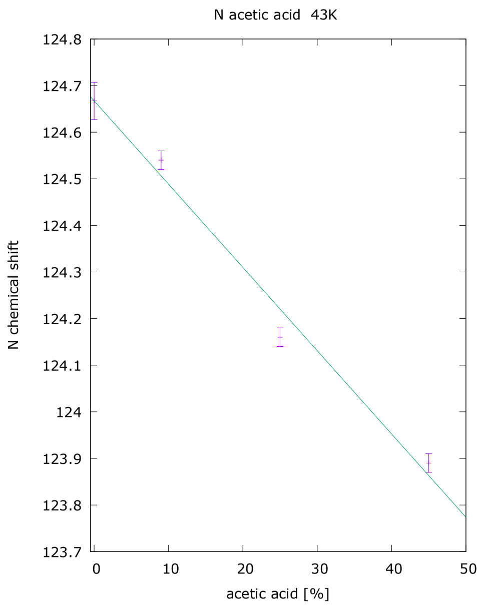
N acetic acid 43K
N chemical shift
acetic acid [%]

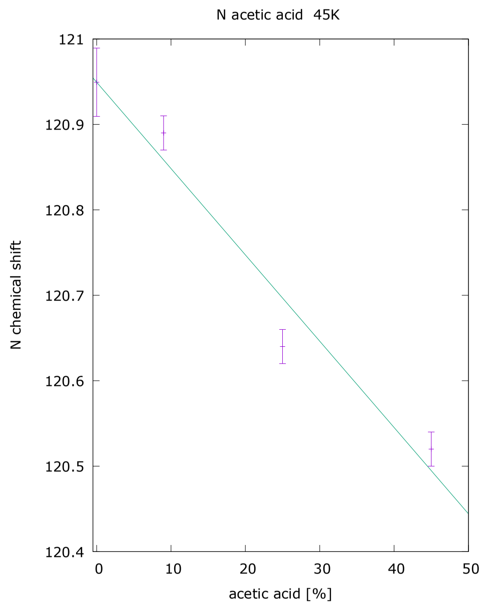
N acetic acid 45K
N chemical shift
acetic acid [%]

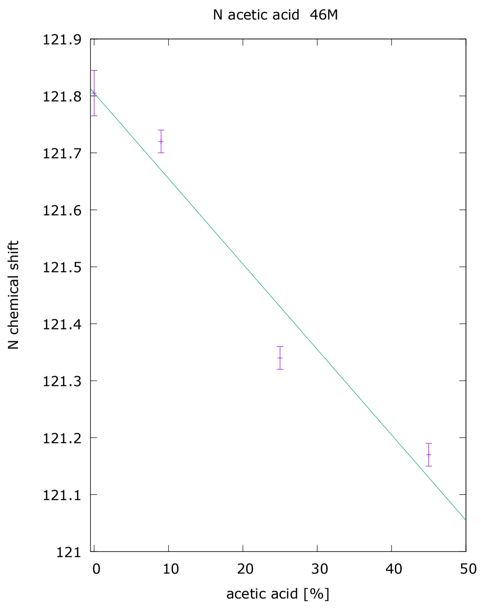
N acetic acid 46M
N chemical shift
acetic acid [%]

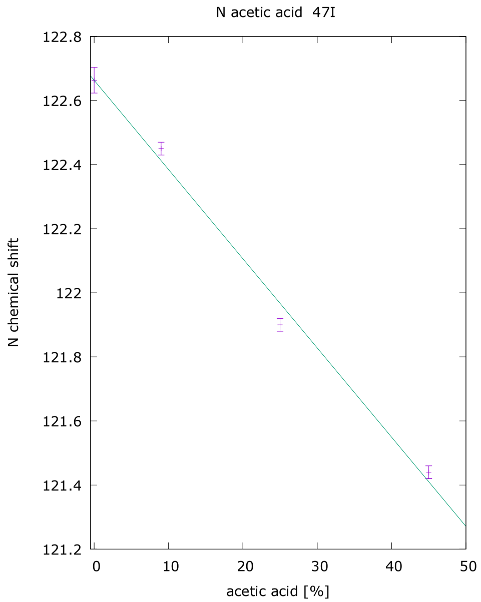
N acetic acid 47I
N chemical shift
acetic acid [%]

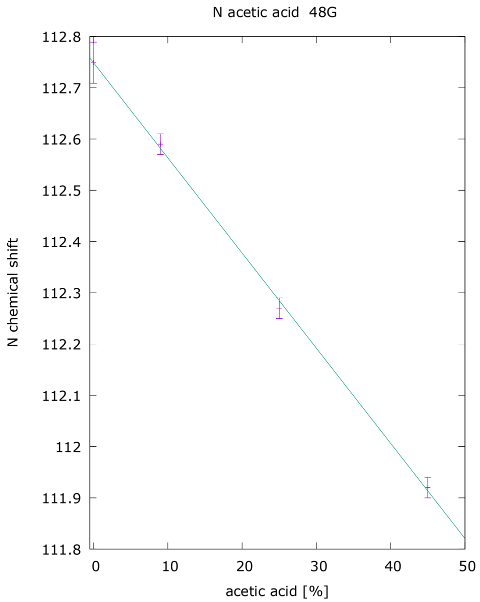
N acetic acid 48G
N chemical shift
acetic acid [%]

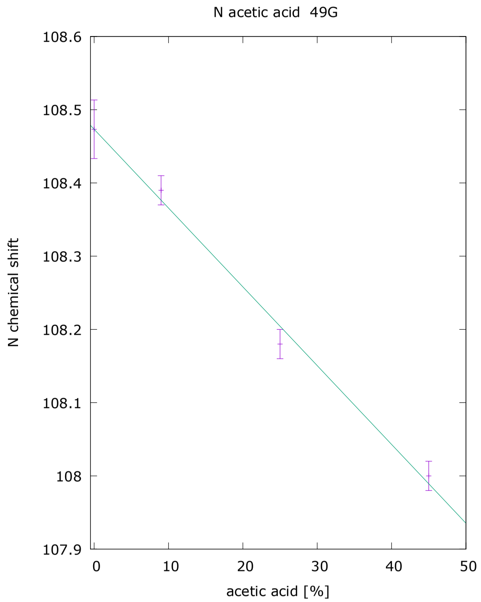
N acetic acid 49G
N chemical shift
acetic acid [%]

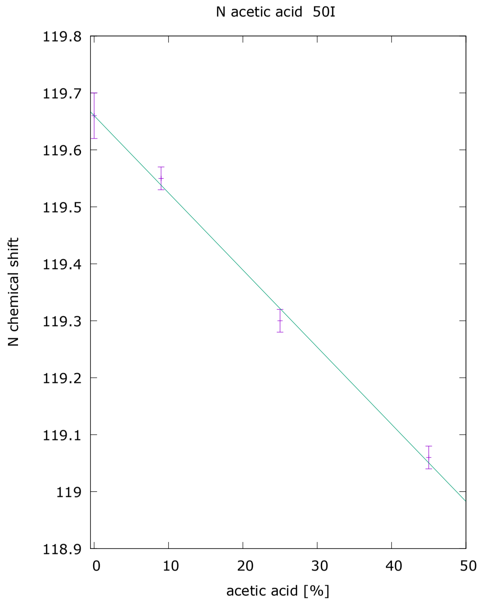
N acetic acid 50I
N chemical shift
acetic acid [%]

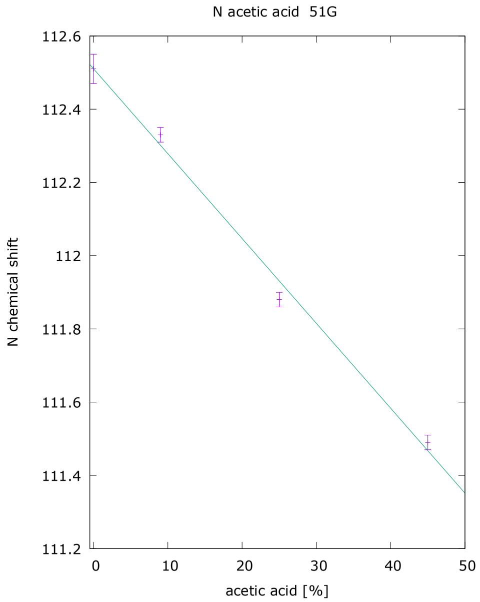
N acetic acid 51G
N chemical shift
acetic acid [%]

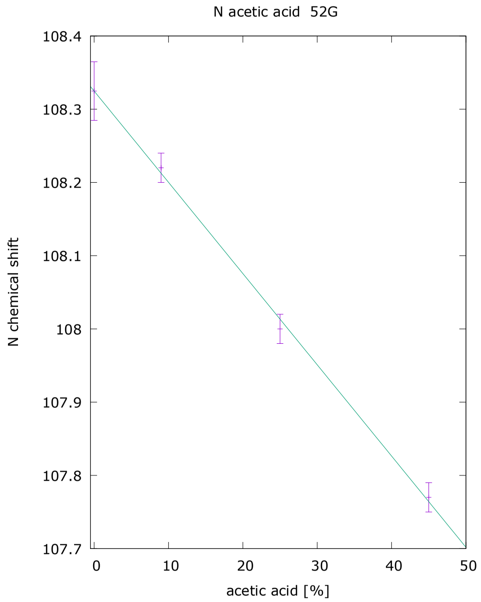
N acetic acid 52G
N chemical shift
acetic acid [%]

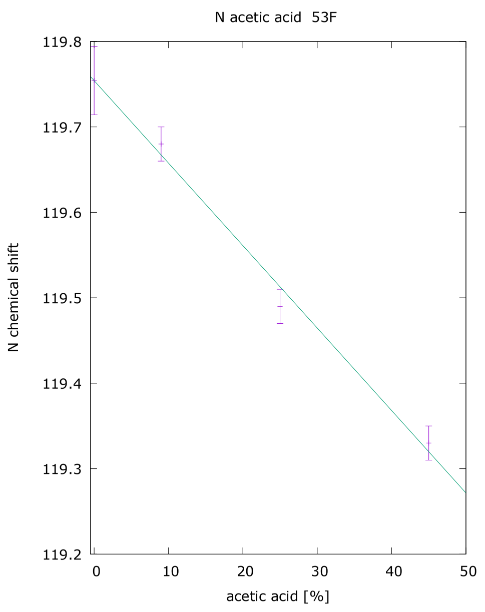
N acetic acid 53F
N chemical shift
119.8
119.7
119.6
119.5
119.4
119.3
119.2
0
10
20
30
40
50
acetic acid [%]

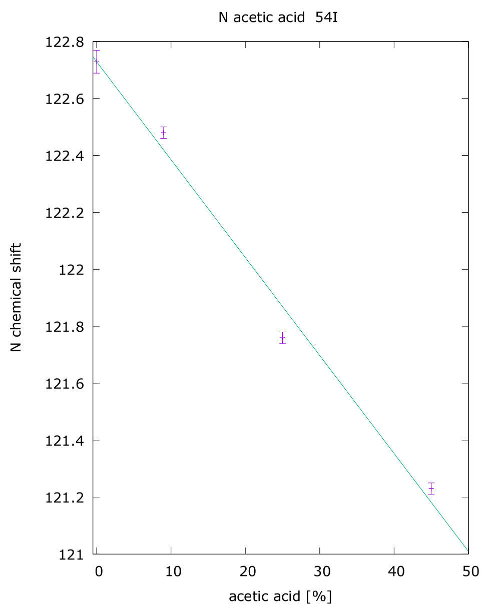
N acetic acid 54I
N chemical shift
122.8
122.6
122.4
122.2
122
121.8
121.6
121.4
121.2
121
0
10
20
30
40
50
acetic acid [%]

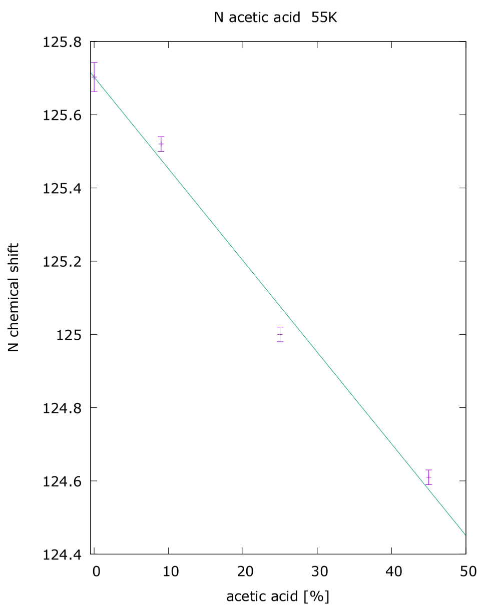
N acetic acid 55K
N chemical shift
125.8
125.6
125.4
125.2
125
124.8
124.6
124.4
0
10
20
30
40
50
acetic acid [%]

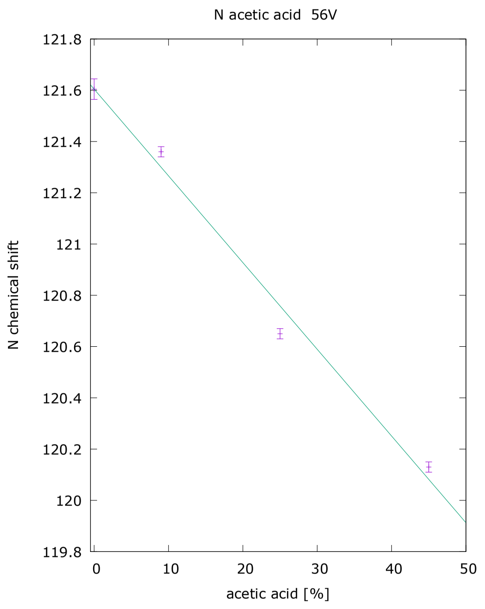
N acetic acid 56V
N chemical shift
121.8
121.6
121.4
121.2
121
120.8
120.6
120.4
120.2
120
119.8
0
10
20
30
40
50
acetic acid [%]

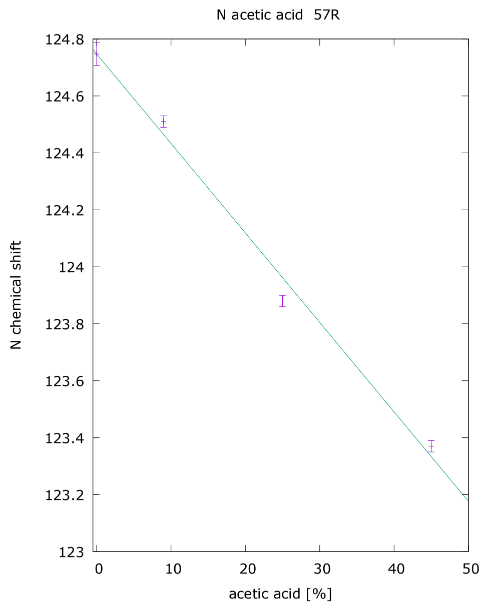
N acetic acid 57R
N chemical shift
124.8
124.6
124.4
124.2
124
123.8
123.6
123.4
123.2
123
0
10
20
30
40
50
acetic acid [%]

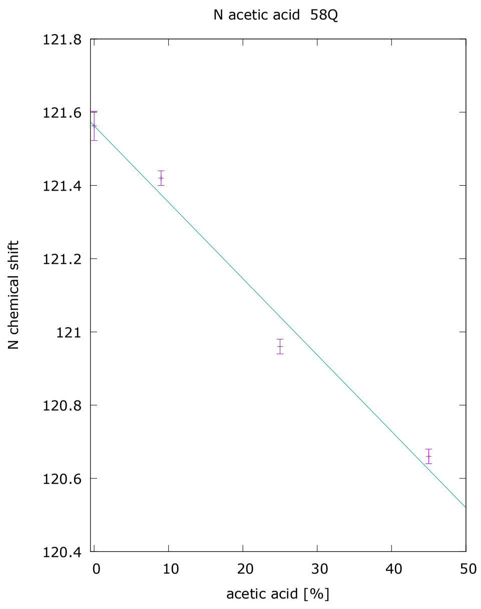
N acetic acid 58Q
N chemical shift
121.8
121.6
121.4
121.2
121
120.8
120.6
120.4
0
10
20
30
40
50
acetic acid [%]

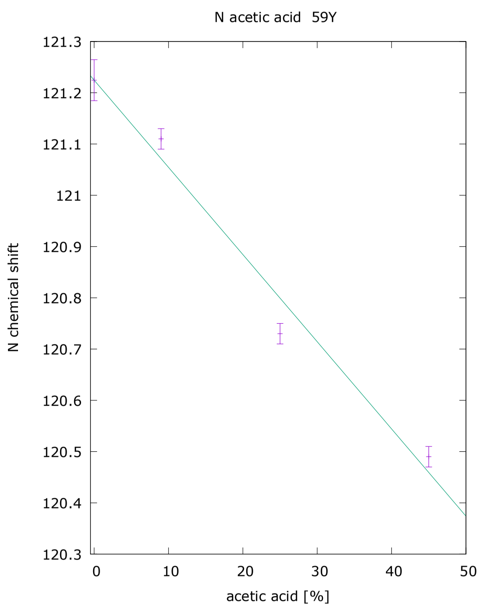
N acetic acid 59Y
N chemical shift
121.3
121.2
121.1
121
120.9
120.8
120.7
120.6
120.5
120.4
120.3
0
10
20
30
40
50
acetic acid [%]

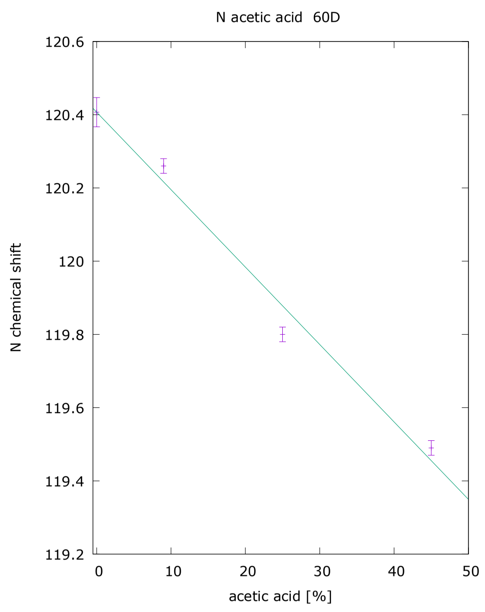
N acetic acid 60D
N chemical shift
120.6
120.4
120.2
120
119.8
119.6
119.4
119.2
0
10
20
30
40
50
acetic acid [%]

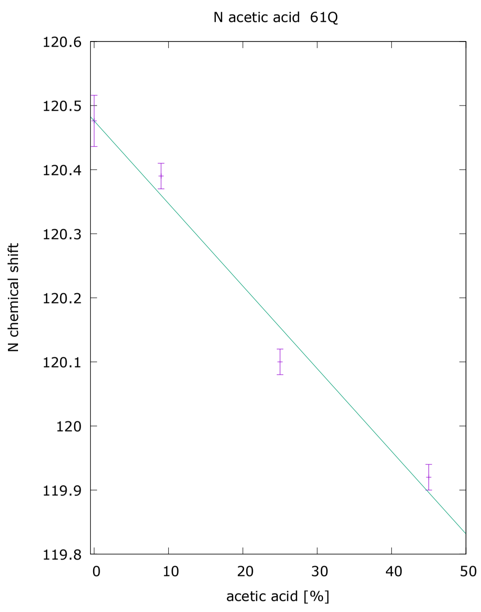
N acetic acid 61Q
N chemical shift
120.6
120.5
120.4
120.3
120.2
120.1
120
119.9
119.8
0
10
20
30
40
50
acetic acid [%]

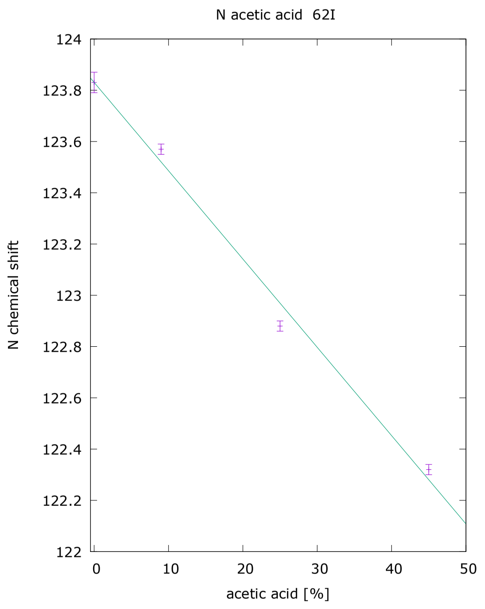
N acetic acid 62I
N chemical shift
124
123.8
123.6
123.4
123.2
123
122.8
122.6
122.4
122.2
122
0
10
20
30
40
50
acetic acid [%]

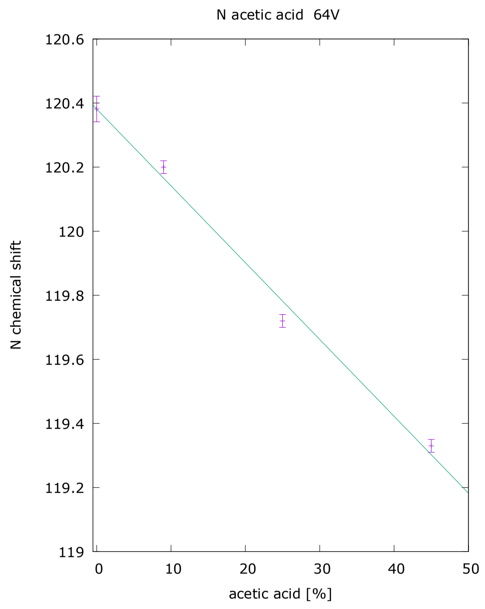
N acetic acid 64V
N chemical shift
120.6
120.4
120.2
120
119.8
119.6
119.4
119.2
119
0
10
20
30
40
50
acetic acid [%]

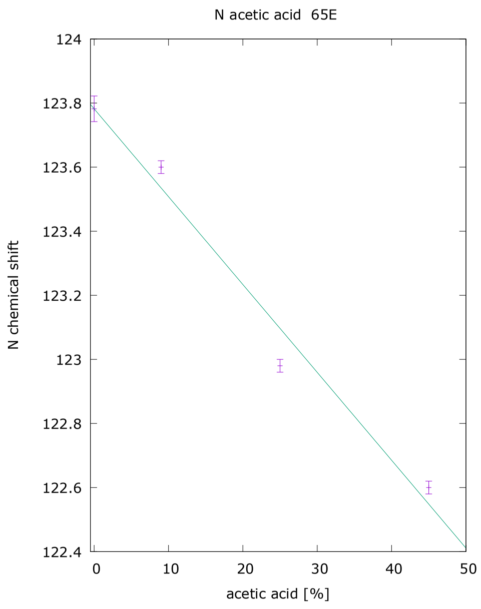
N acetic acid 65E
N chemical shift
124
123.8
123.6
123.4
123.2
123
122.8
122.6
122.4
0
10
20
30
40
50
acetic acid [%]

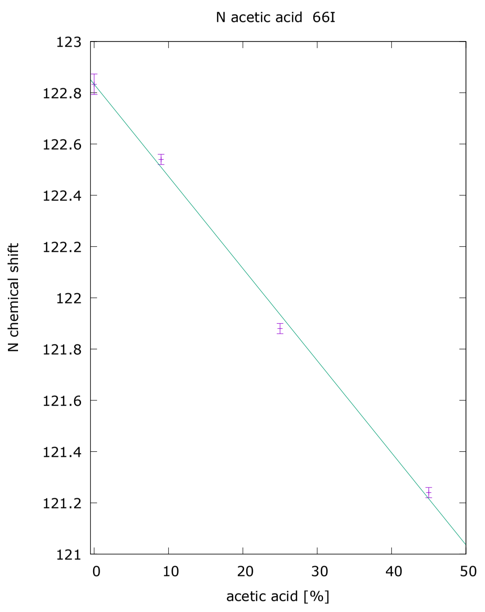
N acetic acid 66I
N chemical shift
acetic acid [%]

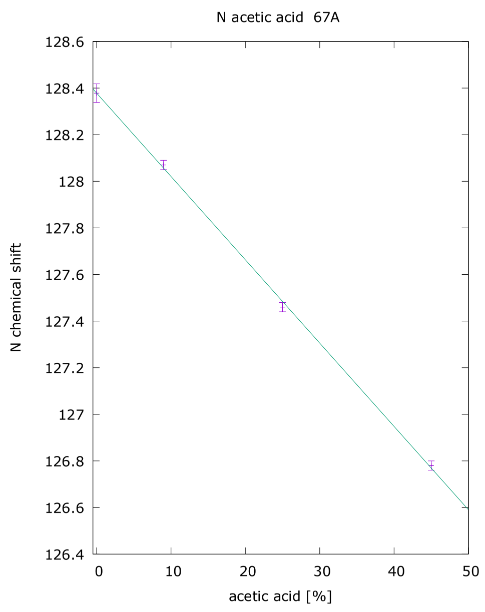
N acetic acid 67A
N chemical shift
acetic acid [%]

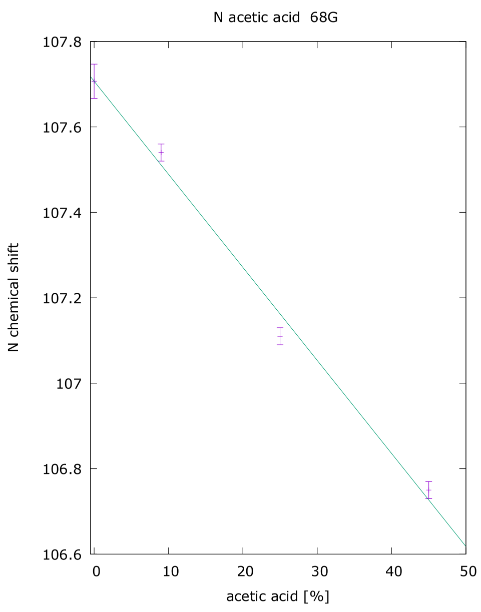
N acetic acid 68G
N chemical shift
acetic acid [%]

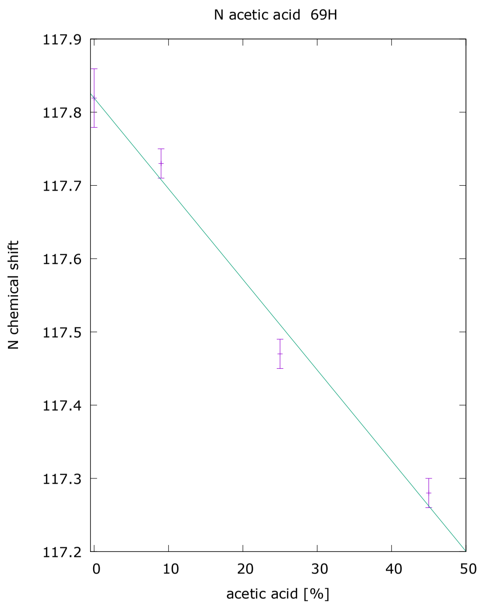
N acetic acid 69H
N chemical shift
acetic acid [%]

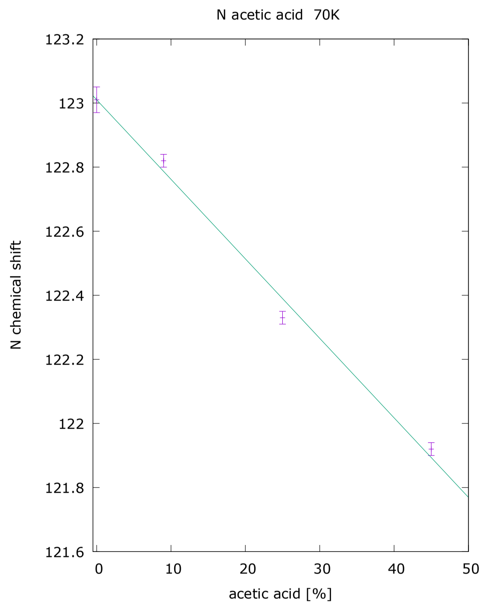
N acetic acid 70K
N chemical shift
acetic acid [%]

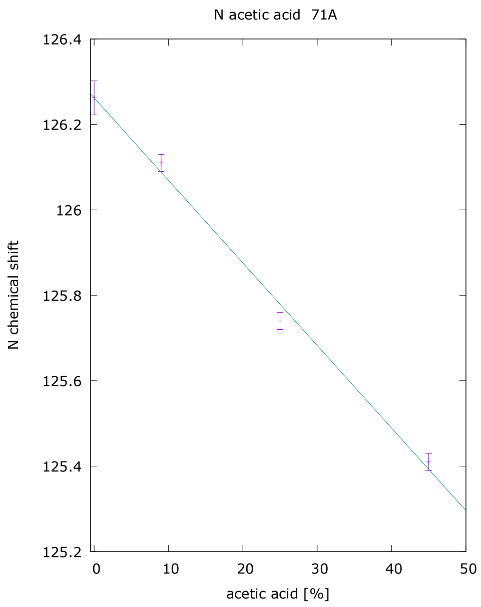
N acetic acid 71A
N chemical shift
acetic acid [%]

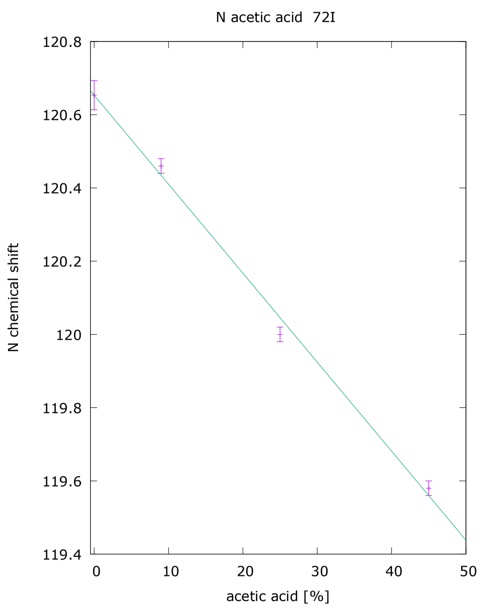
N acetic acid 72I
N chemical shift
120.8
120.6
120.4
120.2
120
119.8
119.6
119.4
0
10
20
30
40
50
acetic acid [%]

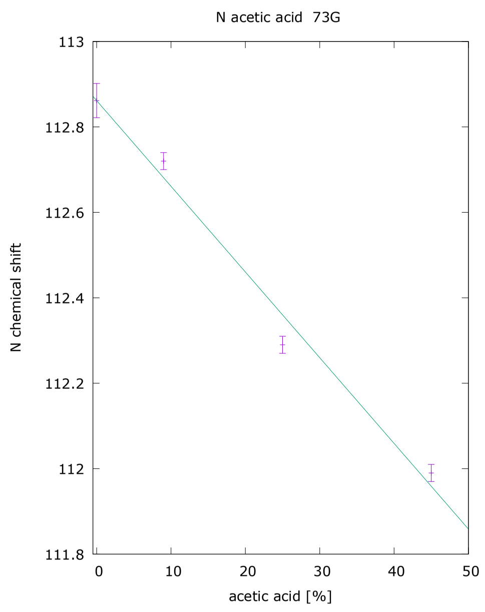
N acetic acid 73G
N chemical shift
113
112.8
112.6
112.4
112.2
112
111.8
0
10
20
30
40
50
acetic acid [%]

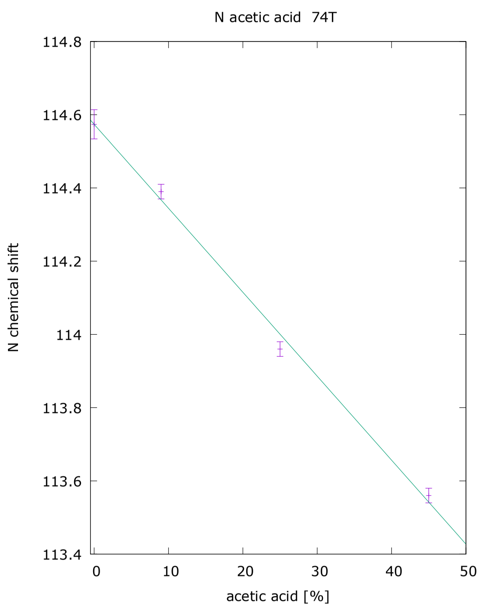
N acetic acid 74T
N chemical shift
114.8
114.6
114.4
114.2
114
113.8
113.6
113.4
0
10
20
30
40
50
acetic acid [%]

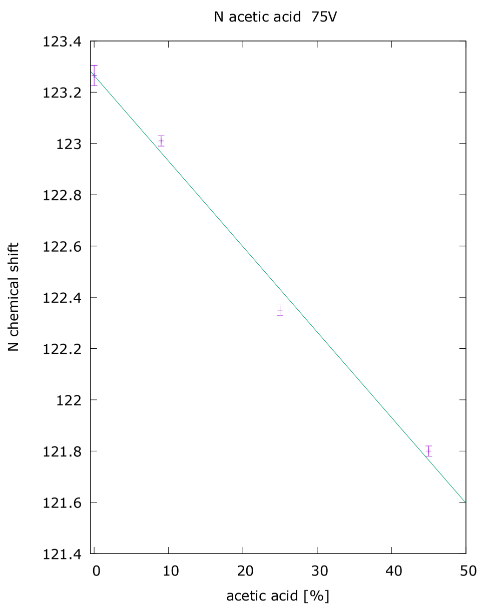
N acetic acid 75V
N chemical shift
123.4
123.2
123
122.8
122.6
122.4
122.2
122
121.8
121.6
121.4
0
10
20
30
40
50
acetic acid [%]

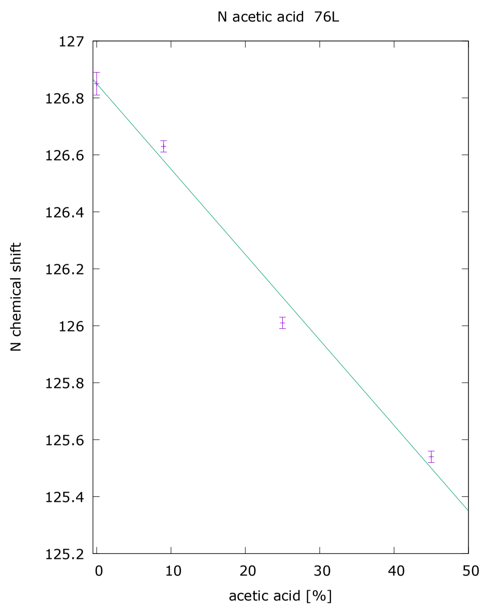
N acetic acid 76L
N chemical shift
127
126.8
126.6
126.4
126.2
126
125.8
125.6
125.4
125.2
0
10
20
30
40
50
acetic acid [%]

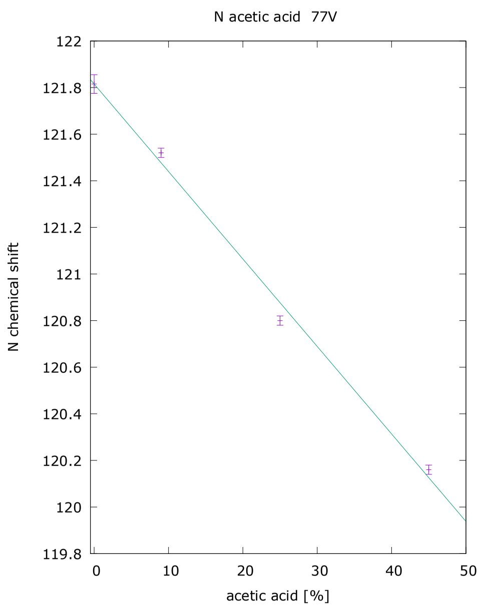
N acetic acid 77V
N chemical shift
122
121.8
121.6
121.4
121.2
121
120.8
120.6
120.4
120.2
120
119.8
0
10
20
30
40
50
acetic acid [%]

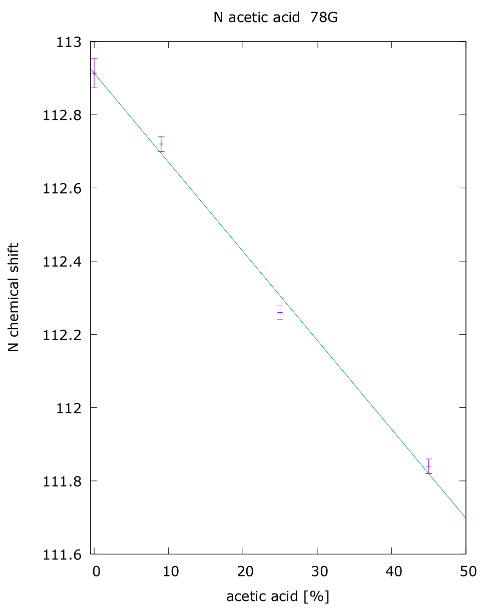
N acetic acid 78G
N chemical shift
acetic acid [%]

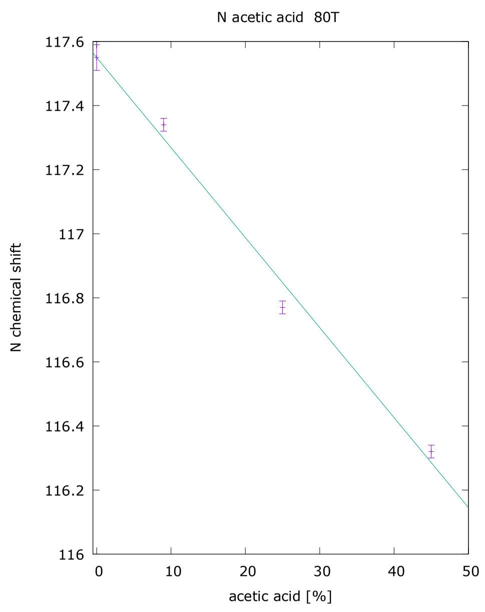
N acetic acid 80T
N chemical shift
acetic acid [%]

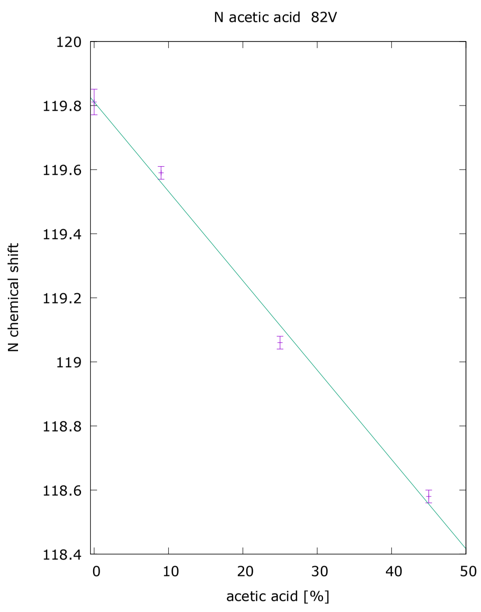
N acetic acid 82V
N chemical shift
acetic acid [%]

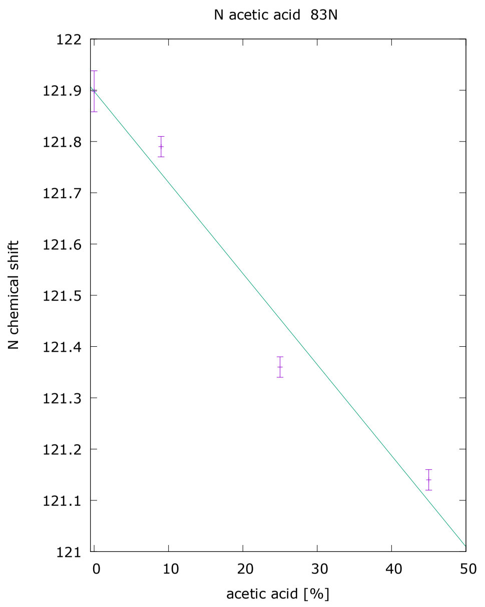
N acetic acid 83N
N chemical shift
acetic acid [%]

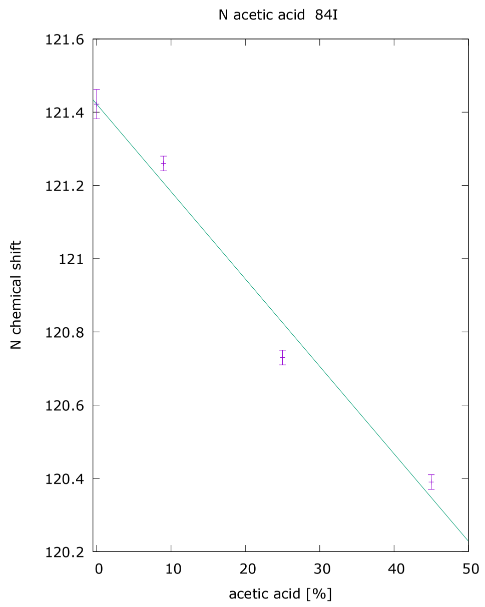
N acetic acid 84I
N chemical shift
acetic acid [%]

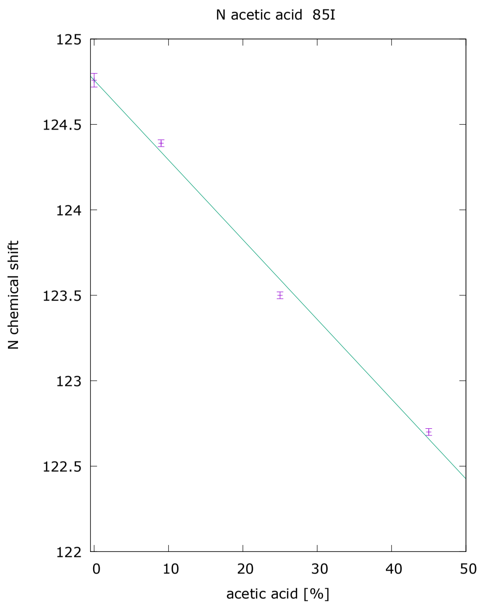
N acetic acid 85I
N chemical shift
acetic acid [%]

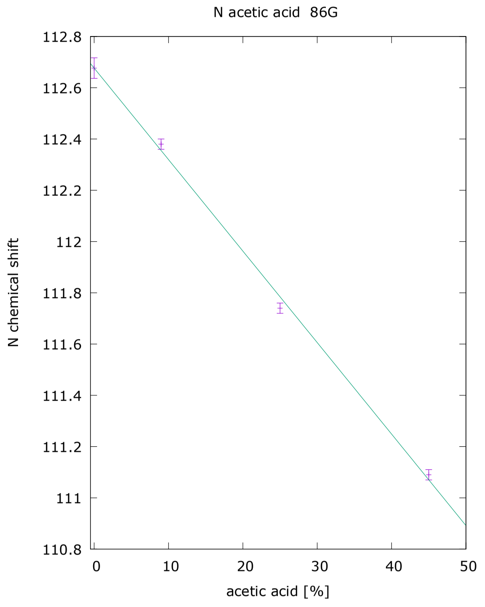
N acetic acid 86G
N chemical shift
112.8
112.6
112.4
112.2
112
111.8
111.6
111.4
111.2
111
110.8
0
10
20
30
40
50
acetic acid [%]

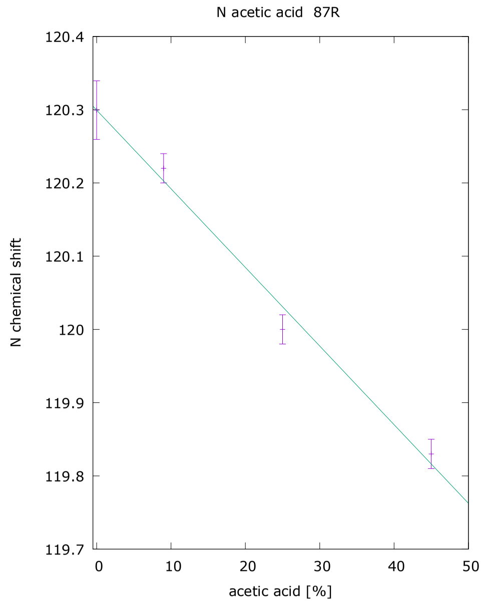
N acetic acid 87R
N chemical shift
120.4
120.3
120.2
120.1
120
119.9
119.8
119.7
0
10
20
30
40
50
acetic acid [%]

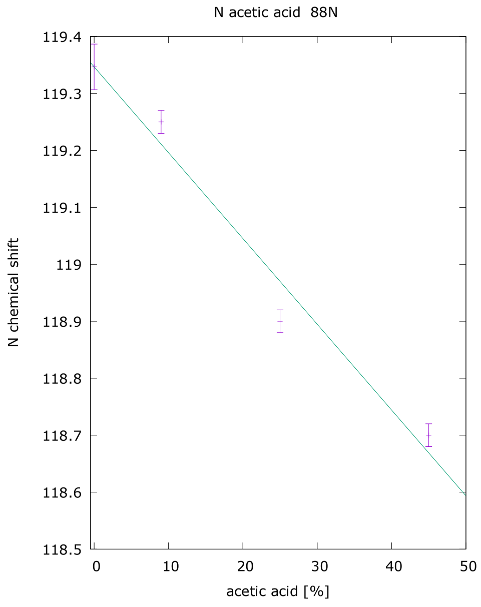
N acetic acid 88N
N chemical shift
119.4
119.3
119.2
119.1
119
118.9
118.8
118.7
118.6
118.5
0
10
20
30
40
50
acetic acid [%]

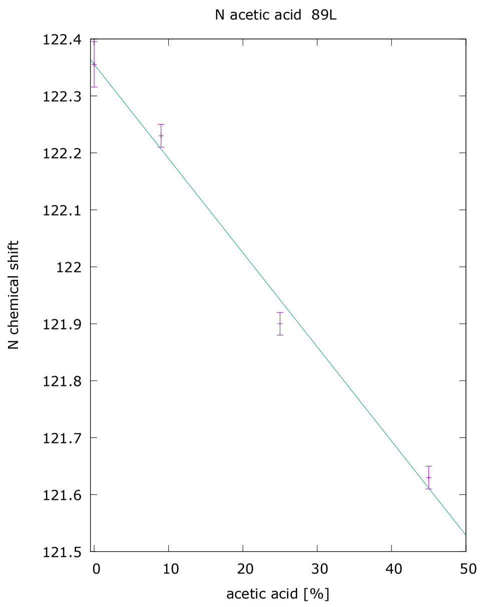
N acetic acid 89L
N chemical shift
122.4
122.3
122.2
122.1
122
121.9
121.8
121.7
121.6
121.5
0
10
20
30
40
50
acetic acid [%]

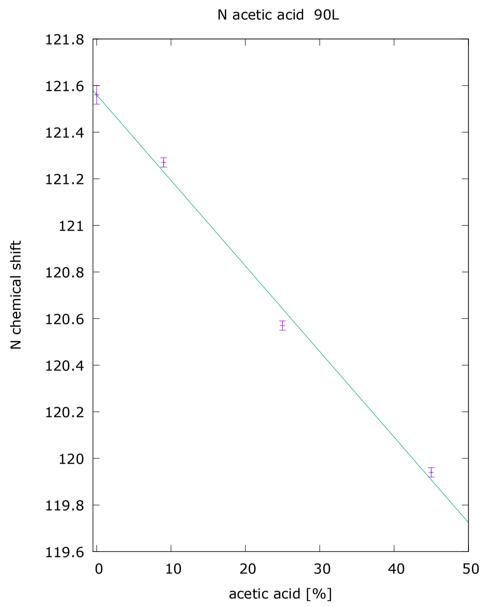
N acetic acid 90L
N chemical shift
121.8
121.6
121.4
121.2
121
120.8
120.6
120.4
120.2
120
119.8
119.6
0
10
20
30
40
50
acetic acid [%]

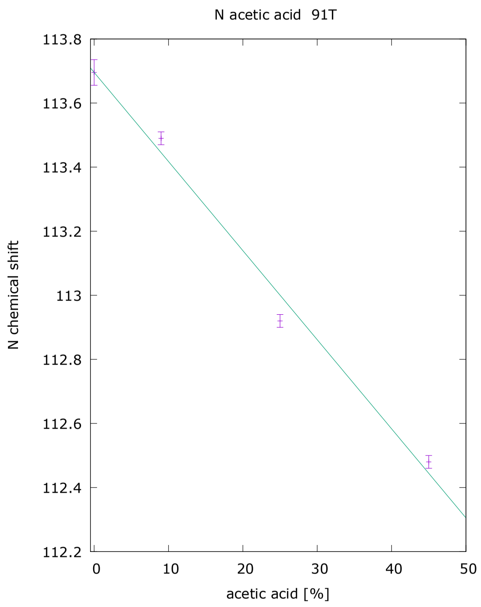
N acetic acid 91T
N chemical shift
113.8
113.6
113.4
113.2
113
112.8
112.6
112.4
112.2
0
10
20
30
40
50
acetic acid [%]

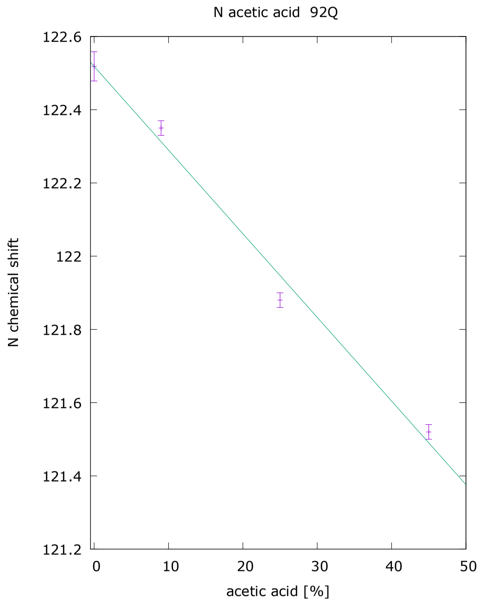
N acetic acid 92Q
N chemical shift
122.6
122.4
122.2
122
121.8
121.6
121.4
121.2
0
10
20
30
40
50
acetic acid [%]

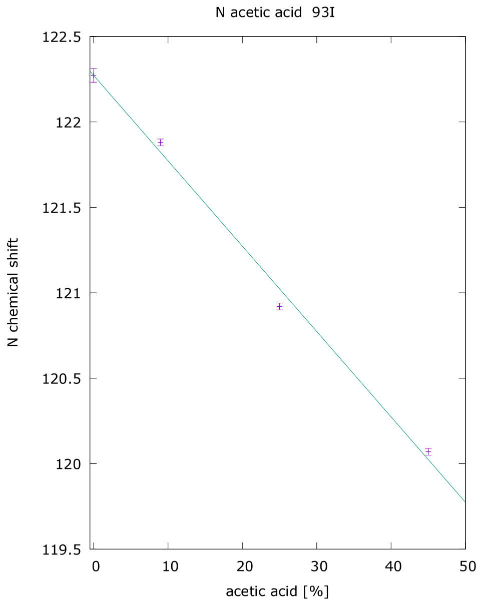
N acetic acid 93I
N chemical shift
122.5
122
121.5
121
120.5
120
119.5
0
10
20
30
40
50
acetic acid [%]

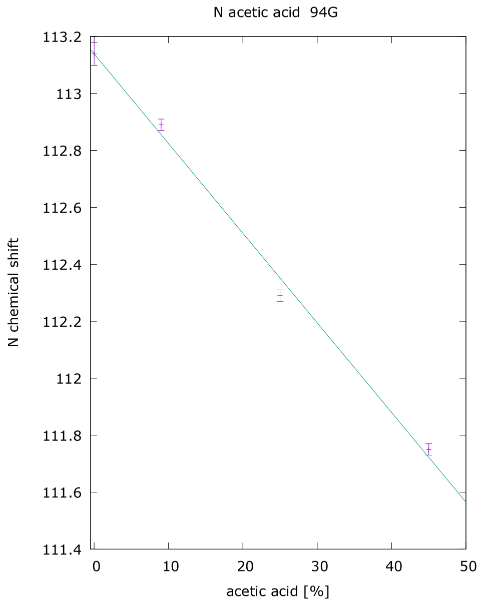
N acetic acid 94G
N chemical shift
113.2
113
112.8
112.6
112.4
112.2
112
111.8
111.6
111.4
0
10
20
30
40
50
acetic acid [%]

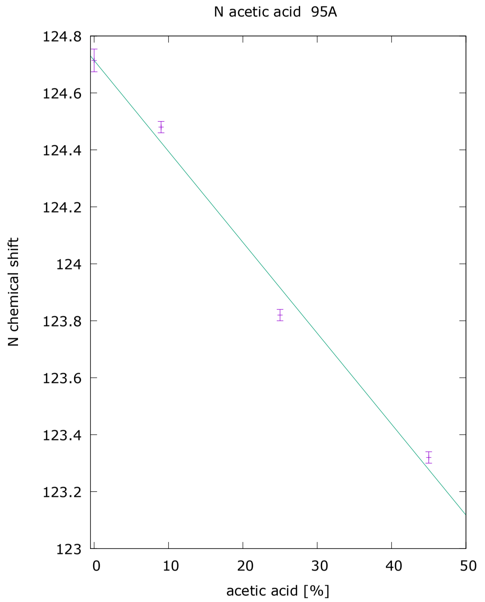
N acetic acid 95A
N chemical shift
124.8
124.6
124.4
124.2
124
123.8
123.6
123.4
123.2
123
0
10
20
30
40
50
acetic acid [%]

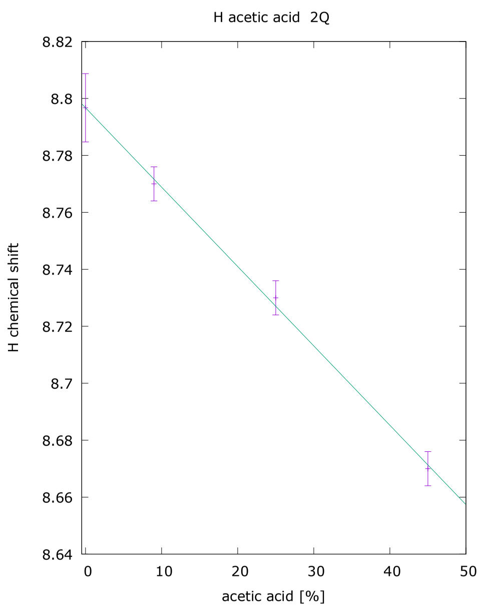
H acetic acid 2Q
H chemical shift
acetic acid [%]

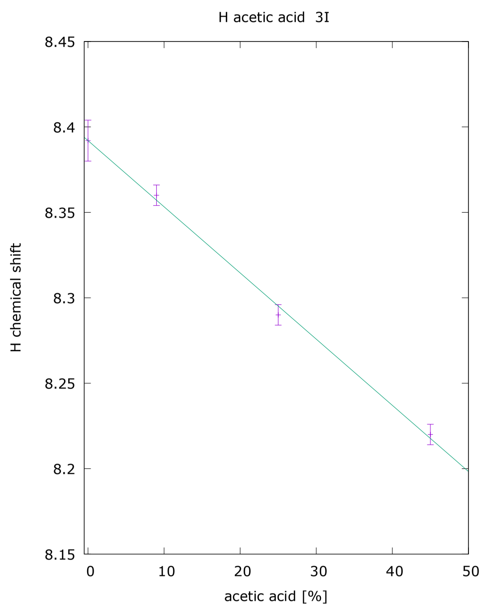
H acetic acid 3I
H chemical shift
acetic acid [%]

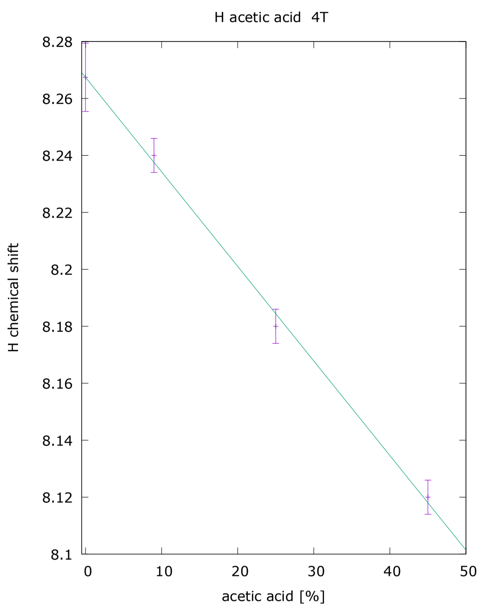
H acetic acid 4T
H chemical shift
acetic acid [%]

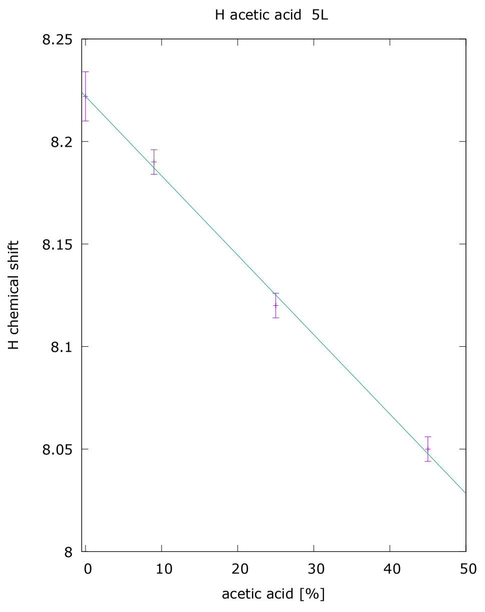
H acetic acid 5L
H chemical shift
acetic acid [%]

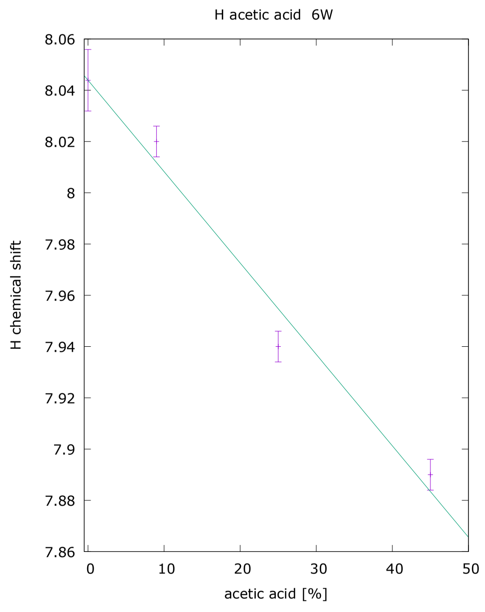
H acetic acid 6W
H chemical shift
acetic acid [%]

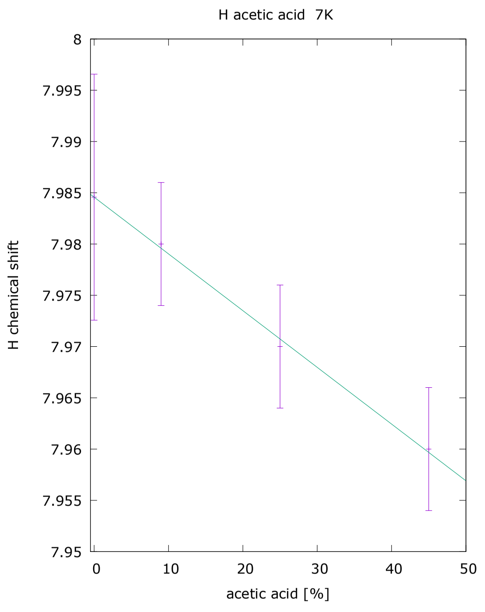
H acetic acid 7K
H chemical shift
acetic acid [%]

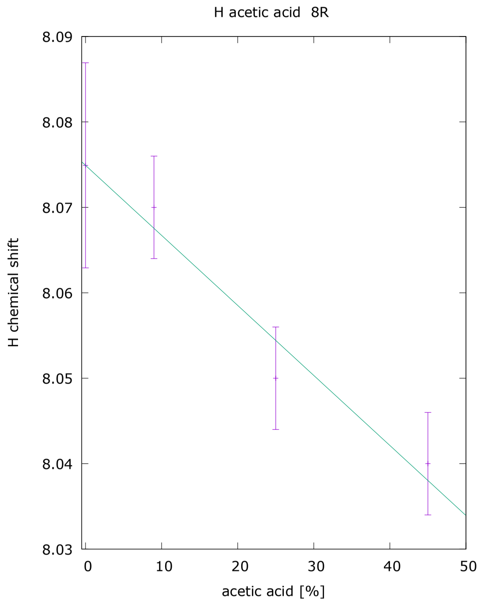
H acetic acid 8R
H chemical shift
acetic acid [%]

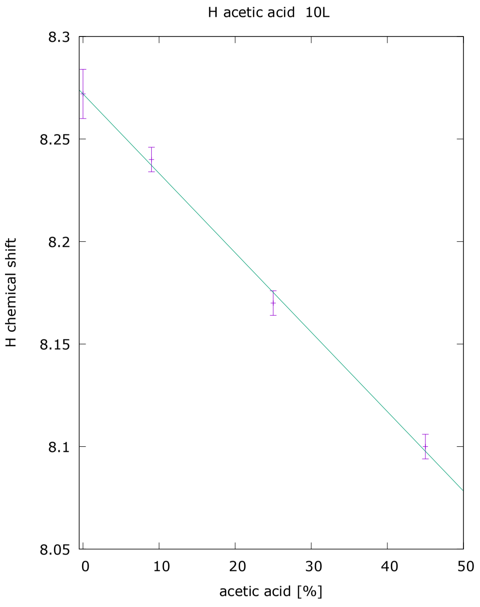
H acetic acid 10L
H chemical shift
acetic acid [%]

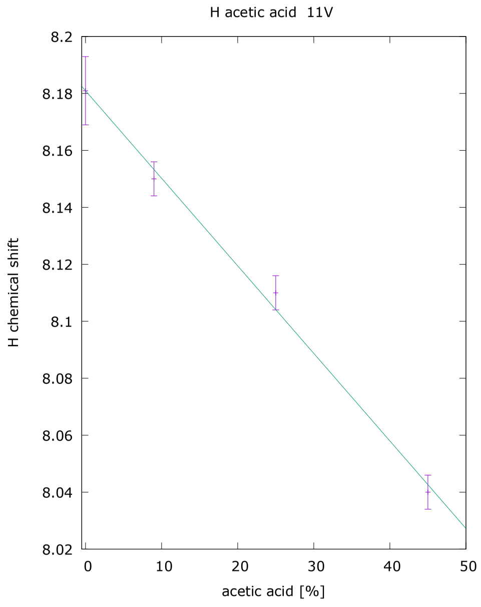
H acetic acid 11V
H chemical shift
acetic acid [%]

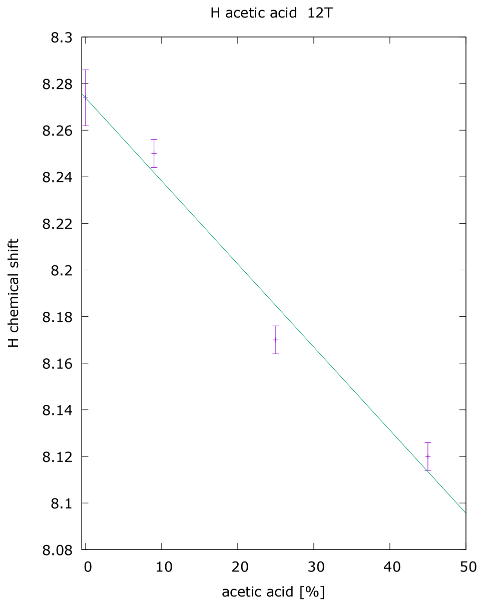
H acetic acid 12T
H chemical shift
acetic acid [%]

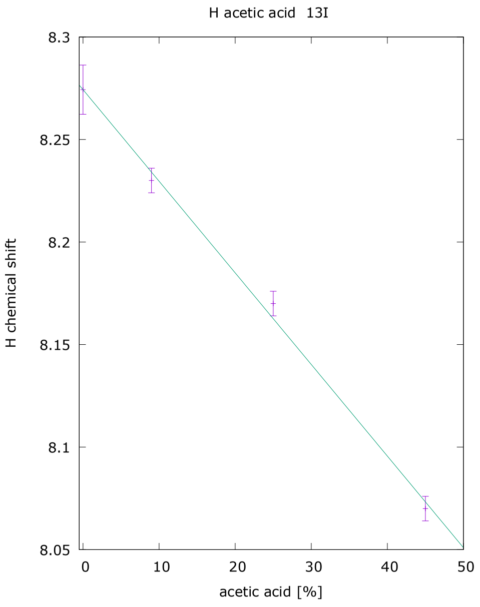
H acetic acid 13I
H chemical shift
acetic acid [%]

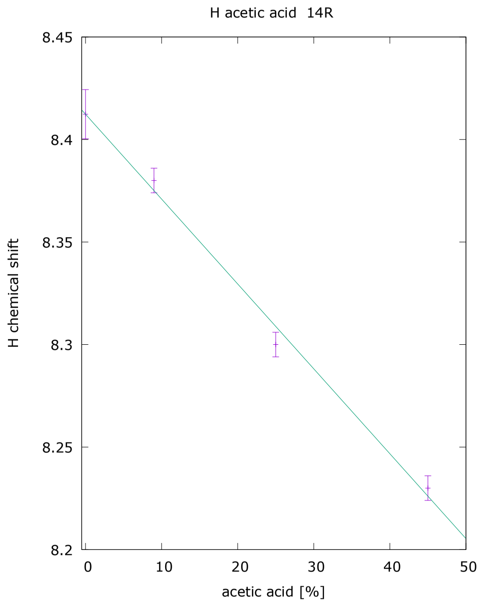
H acetic acid 14R
H chemical shift
acetic acid [%]

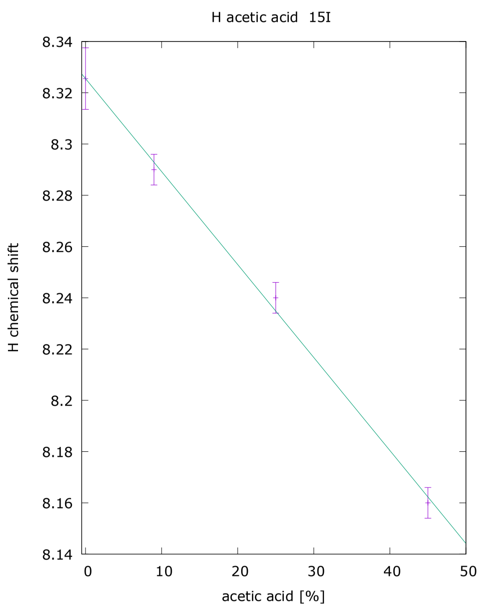
H acetic acid 15I
H chemical shift
acetic acid [%]

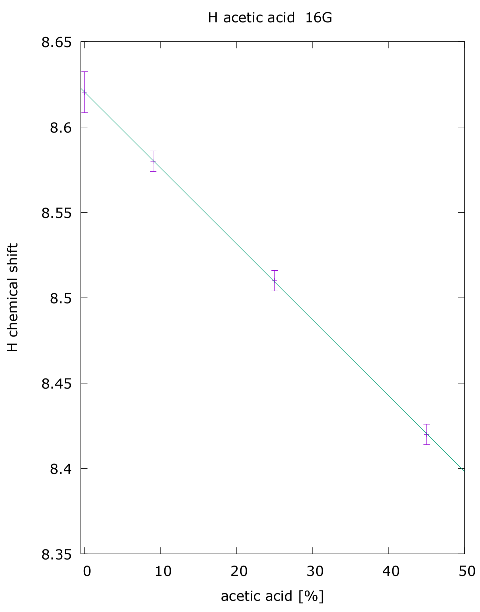
H acetic acid 16G
H chemical shift
acetic acid [%]

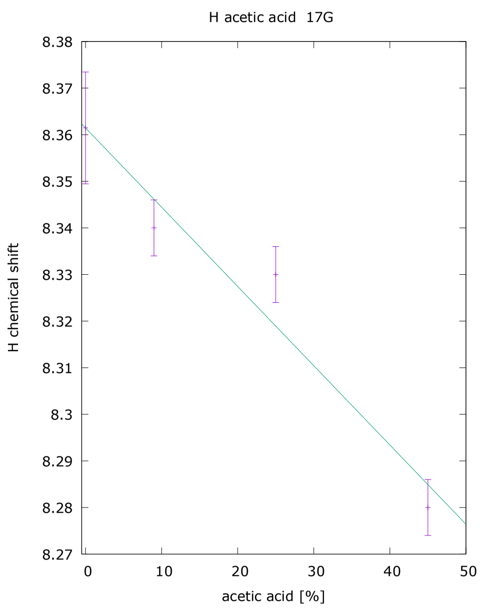
H acetic acid 17G
H chemical shift
acetic acid [%]

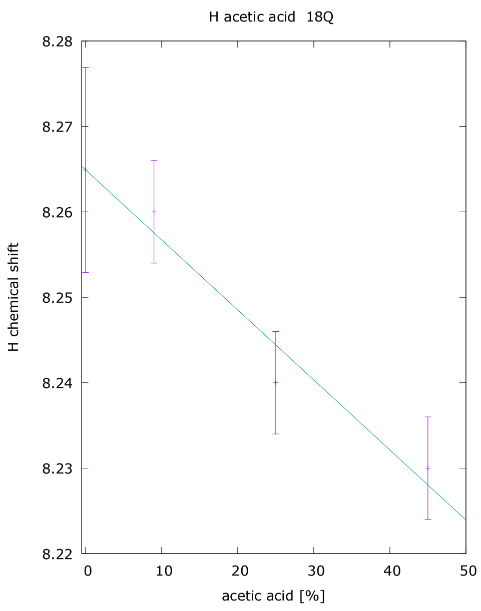
H acetic acid 18Q
H chemical shift
acetic acid [%]

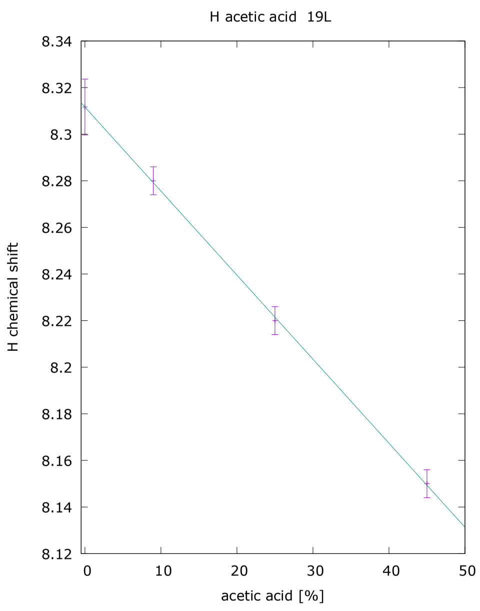
H acetic acid 19L
H chemical shift
acetic acid [%]

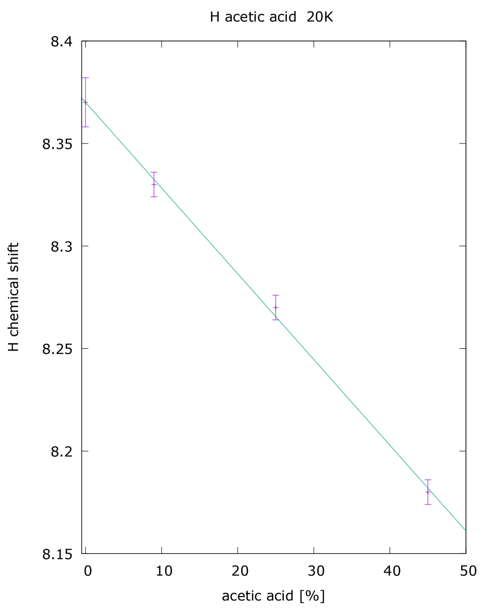
H acetic acid 20K
H chemical shift
acetic acid [%]

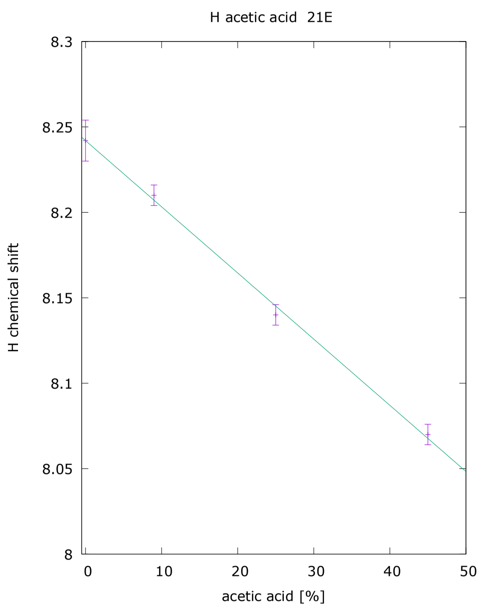
H acetic acid 21E
H chemical shift
acetic acid [%]

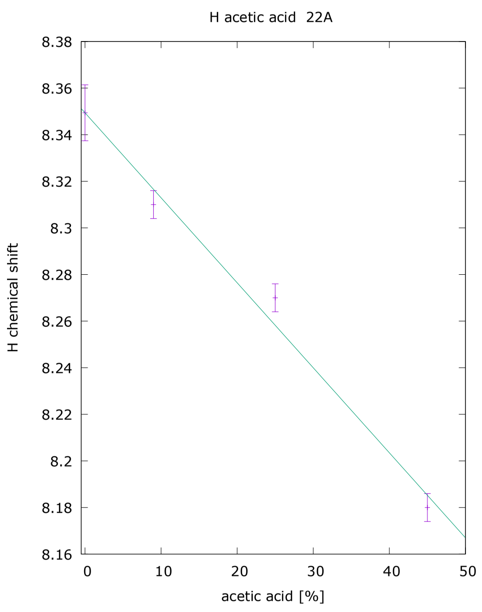
H acetic acid 22A
H chemical shift
acetic acid [%]

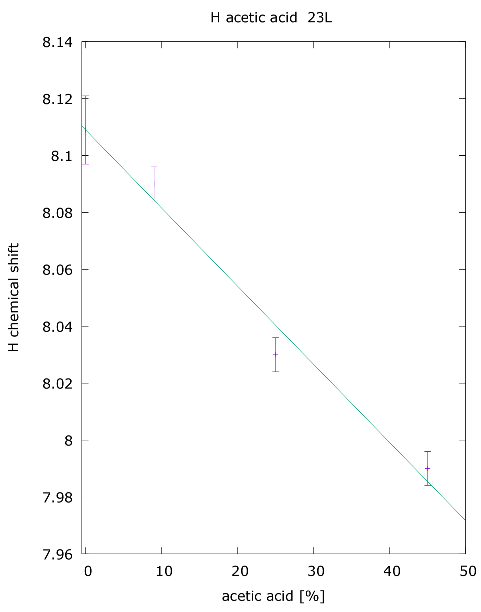
H acetic acid 23L
H chemical shift
acetic acid [%]

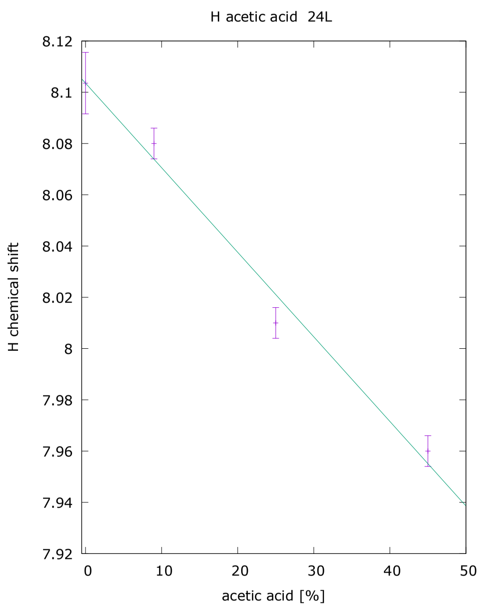
H acetic acid 24L
H chemical shift
acetic acid [%]

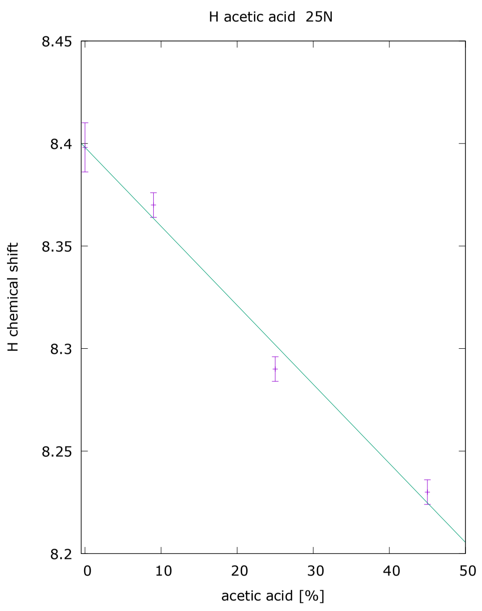
H acetic acid 25N
H chemical shift
acetic acid [%]

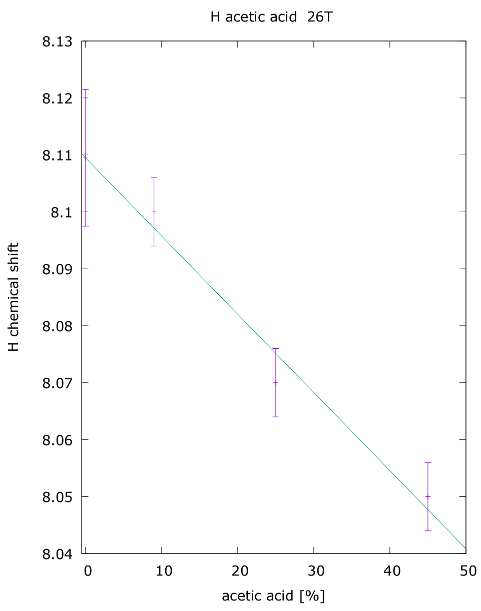
H acetic acid 26T
H chemical shift
acetic acid [%]

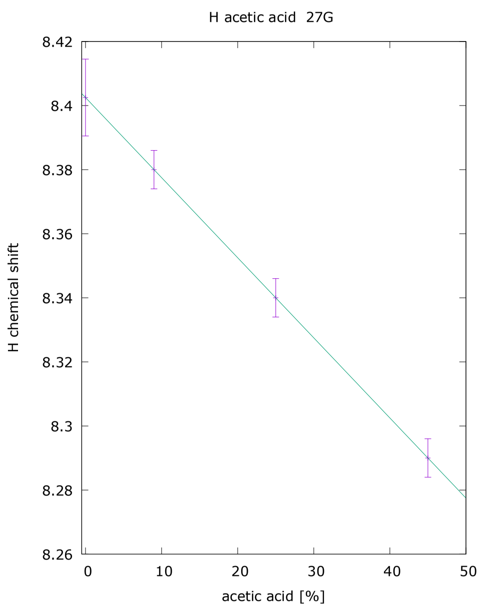
H acetic acid 27G
H chemical shift
acetic acid [%]

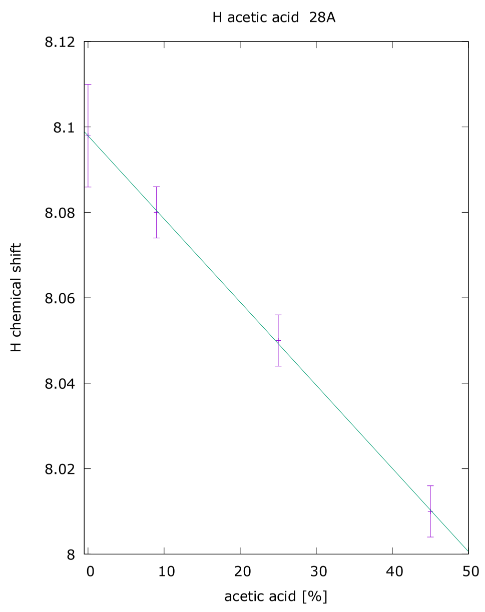
H acetic acid 28A
H chemical shift
acetic acid [%]

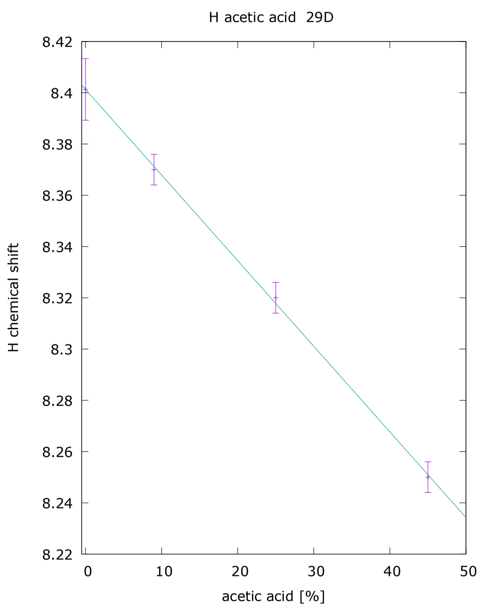
H acetic acid 29D
H chemical shift
acetic acid [%]

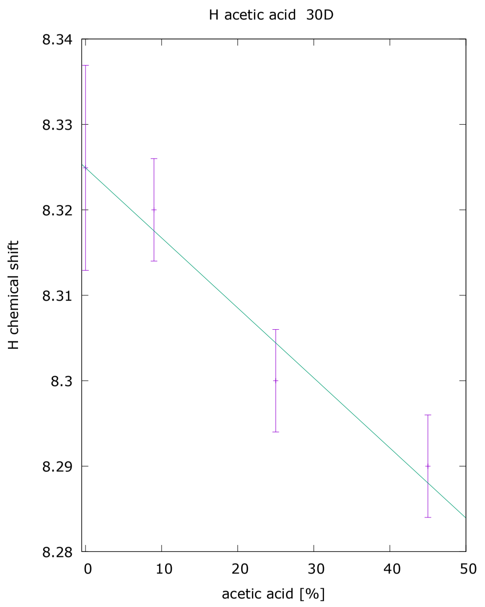
H acetic acid 30D
H chemical shift
acetic acid [%]

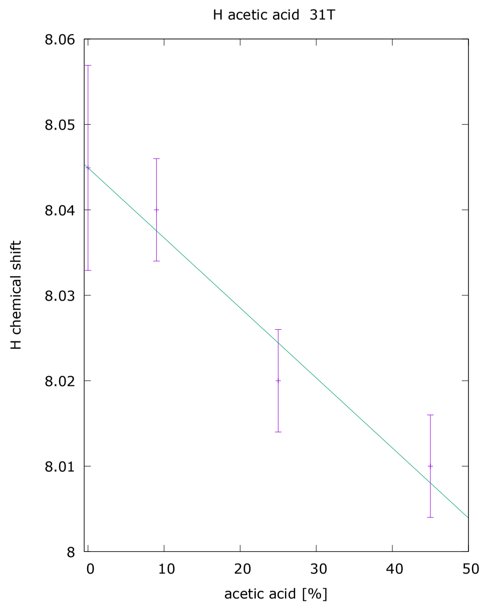
H acetic acid 31T
H chemical shift
acetic acid [%]

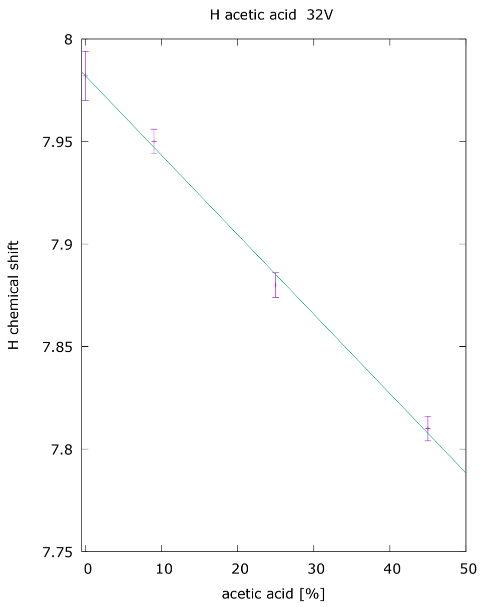
H acetic acid 32V
H chemical shift
acetic acid [%]

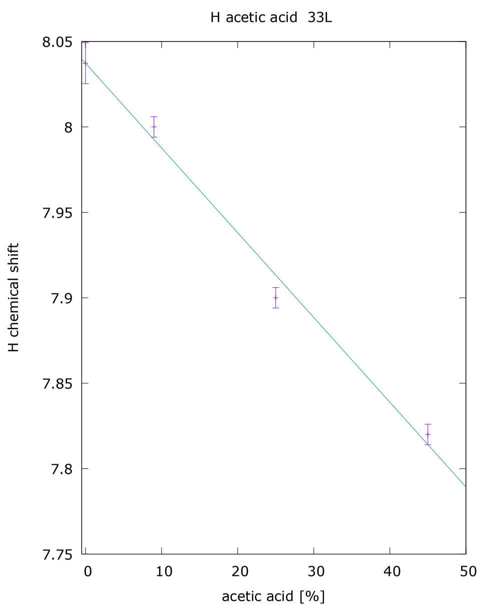
H acetic acid 33L
H chemical shift
acetic acid [%]

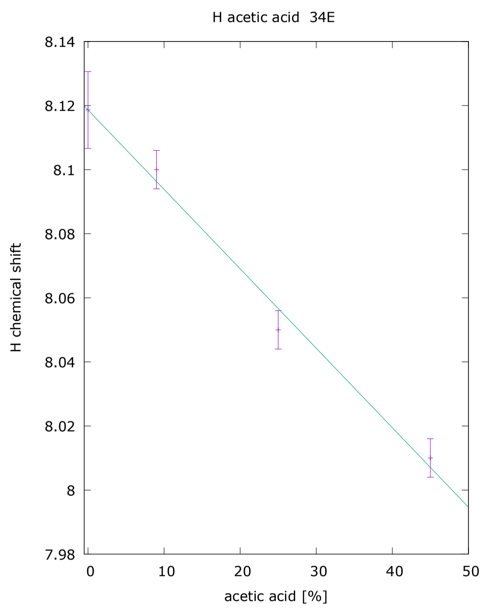
H acetic acid 34E
H chemical shift
acetic acid [%]

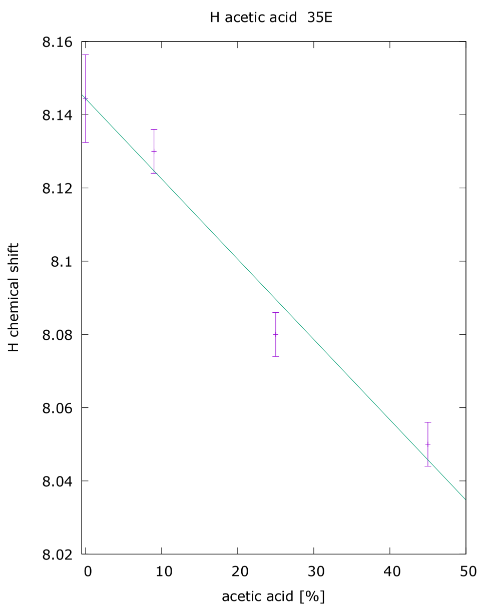
H acetic acid 35E
H chemical shift
acetic acid [%]

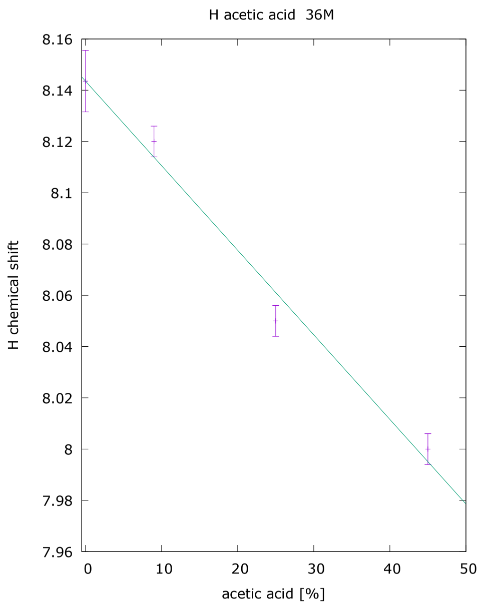
H acetic acid 36M
H chemical shift
acetic acid [%]

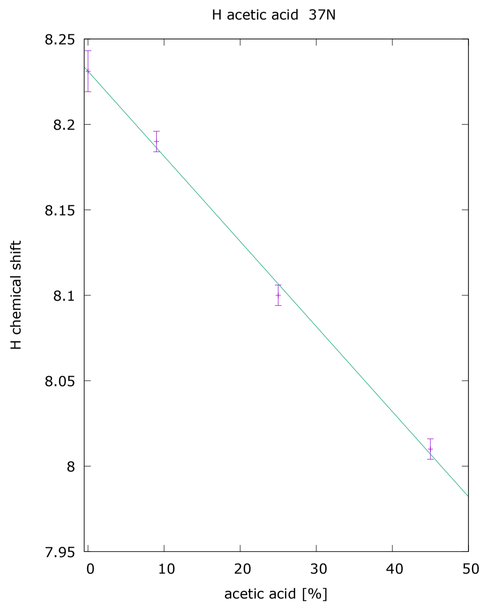
H acetic acid 37N
H chemical shift
acetic acid [%]

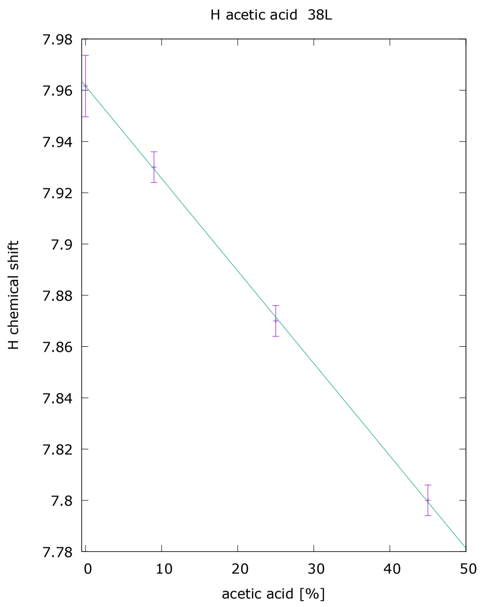
H acetic acid 38L
H chemical shift
acetic acid [%]

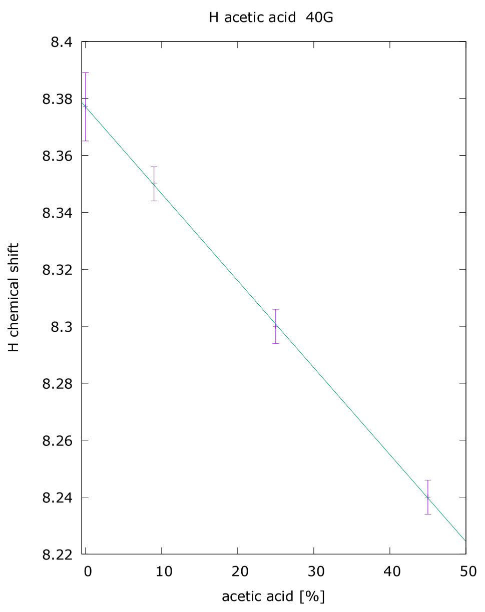
H acetic acid 40G
H chemical shift
8.4
8.38
8.36
8.34
8.32
8.3
8.28
8.26
8.24
8.22
0
10
20
30
40
50
acetic acid [%]

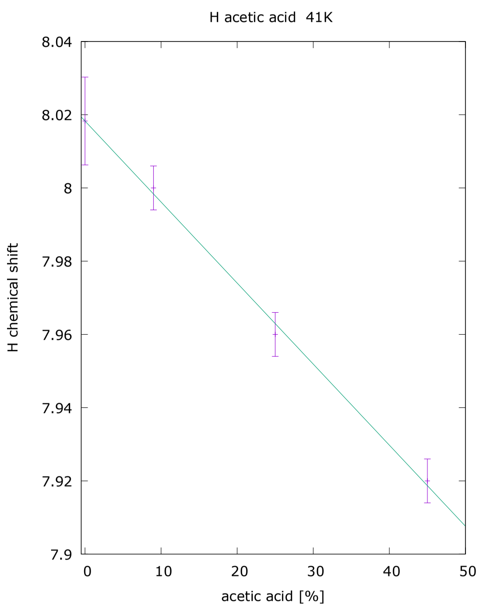
H acetic acid 41K
H chemical shift
8.04
8.02
8
7.98
7.96
7.94
7.92
7.9
0
10
20
30
40
50
acetic acid [%]

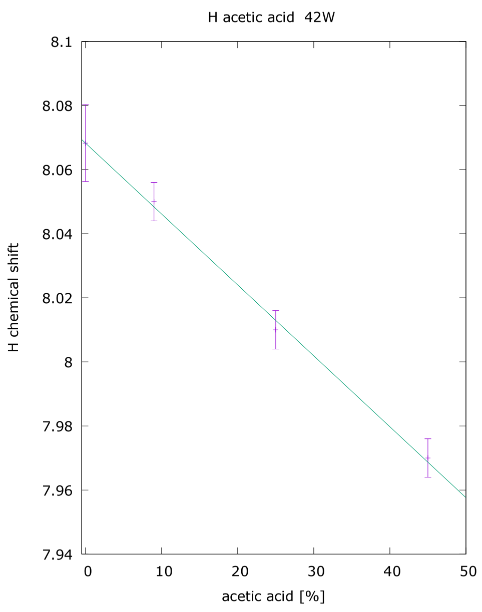
H acetic acid 42W
H chemical shift
8.1
8.08
8.06
8.04
8.02
8
7.98
7.96
7.94
0
10
20
30
40
50
acetic acid [%]

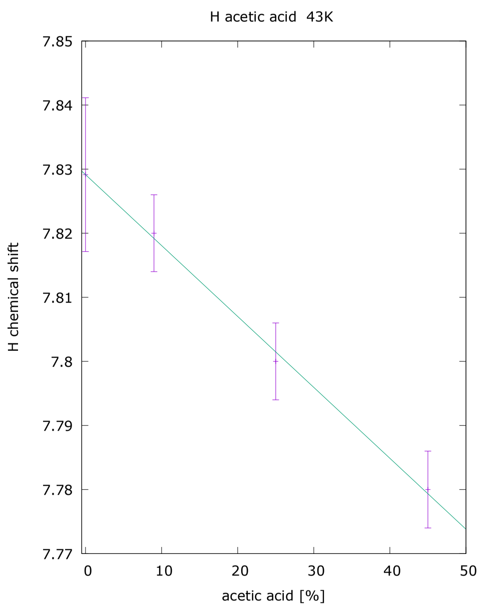
H acetic acid 43K
H chemical shift
7.85
7.84
7.83
7.82
7.81
7.8
7.79
7.78
7.77
0
10
20
30
40
50
acetic acid [%]

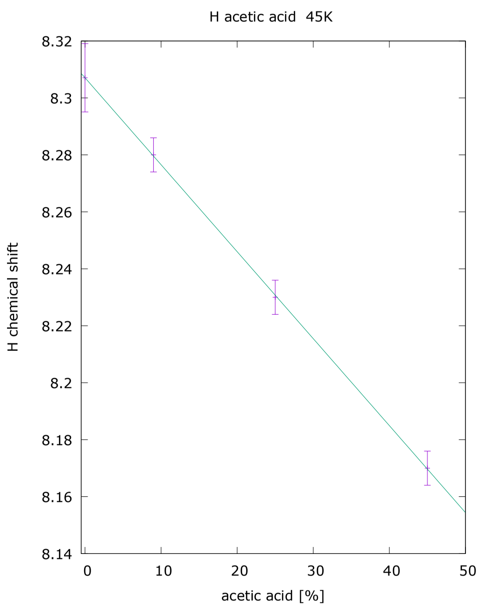
H acetic acid 45K
H chemical shift
8.32
8.3
8.28
8.26
8.24
8.22
8.2
8.18
8.16
8.14
0
10
20
30
40
50
acetic acid [%]

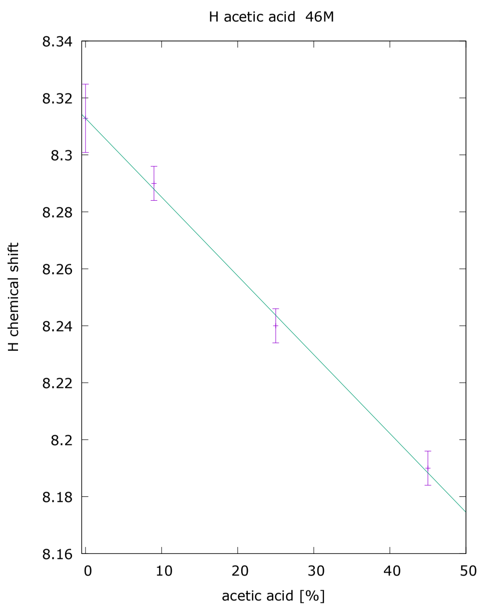
H acetic acid 46M
H chemical shift
8.34
8.32
8.3
8.28
8.26
8.24
8.22
8.2
8.18
8.16
0
10
20
30
40
50
acetic acid [%]

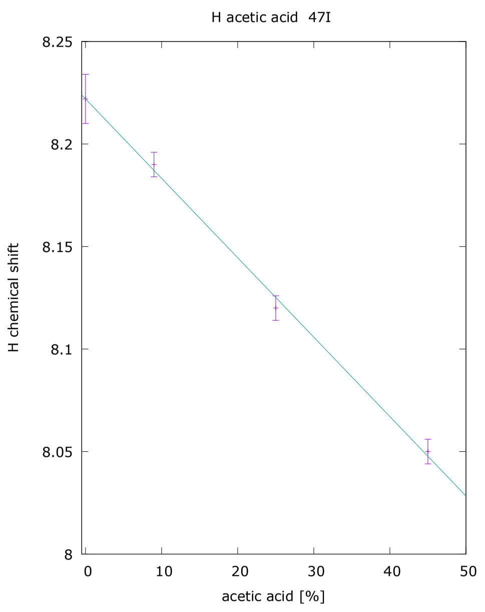
H acetic acid 47I
H chemical shift
8.25
8.2
8.15
8.1
8.05
8
0
10
20
30
40
50
acetic acid [%]

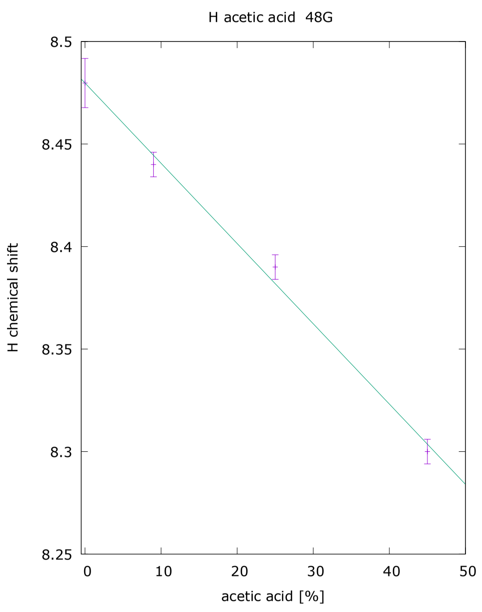
H acetic acid 48G
H chemical shift
8.5
8.45
8.4
8.35
8.3
8.25
0
10
20
30
40
50
acetic acid [%]

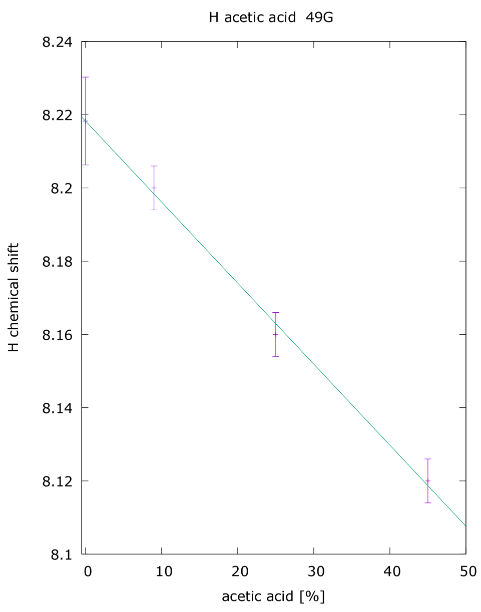
H acetic acid 49G
H chemical shift
8.24
8.22
8.2
8.18
8.16
8.14
8.12
8.1
0
10
20
30
40
50
acetic acid [%]

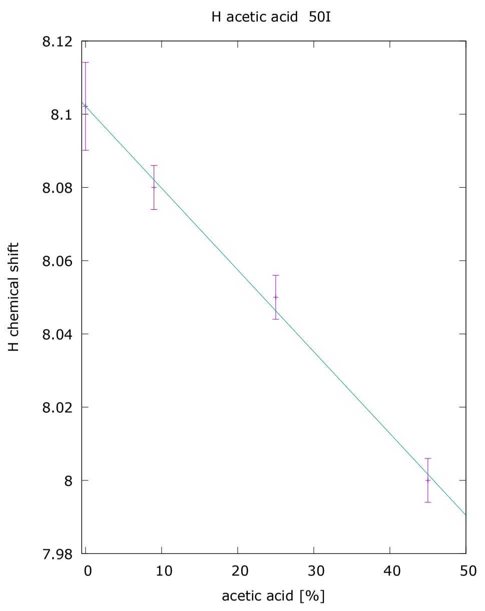
H acetic acid 50I
H chemical shift
8.12
8.1
8.08
8.06
8.04
8.02
8
7.98
0
10
20
30
40
50
acetic acid [%]

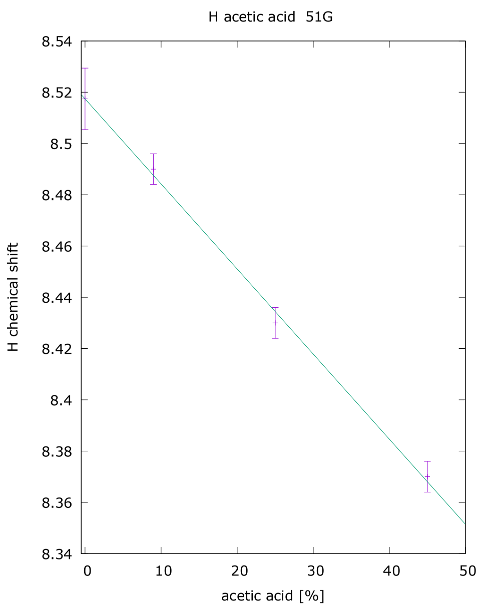
H acetic acid 51G
H chemical shift
8.54
8.52
8.5
8.48
8.46
8.44
8.42
8.4
8.38
8.36
8.34
0
10
20
30
40
50
acetic acid [%]

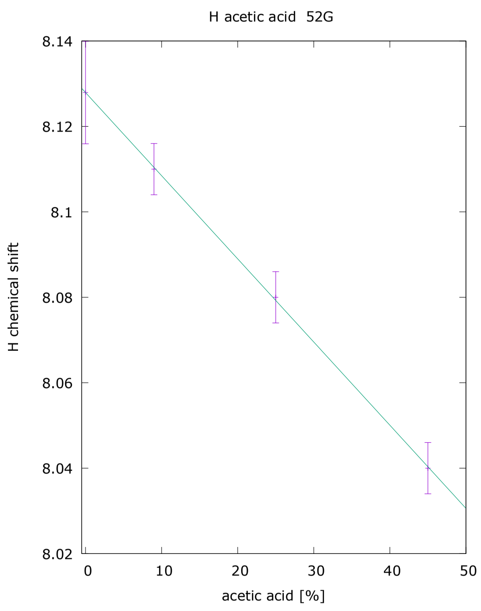
H acetic acid 52G
H chemical shift
8.14
8.12
8.1
8.08
8.06
8.04
8.02
0
10
20
30
40
50
acetic acid [%]

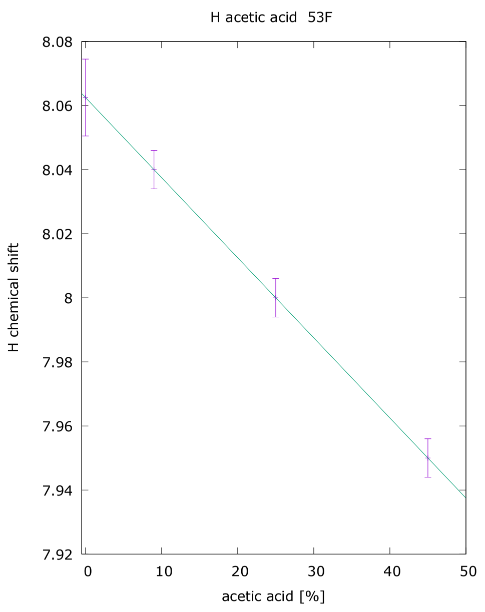
H acetic acid 53F
H chemical shift
acetic acid [%]

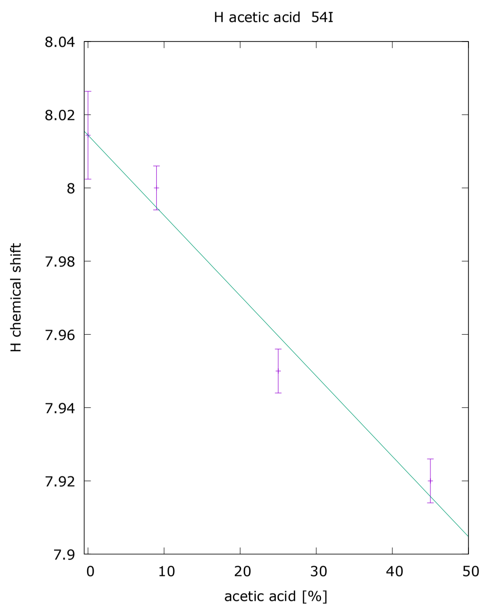
H acetic acid 54I
H chemical shift
acetic acid [%]

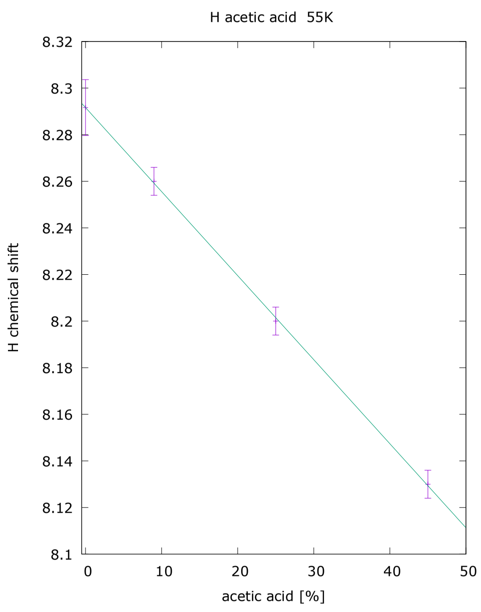
H acetic acid 55K
H chemical shift
acetic acid [%]

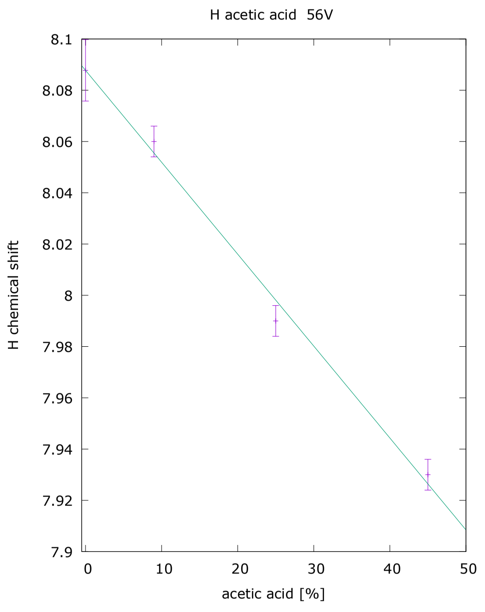
H acetic acid 56V
H chemical shift
acetic acid [%]

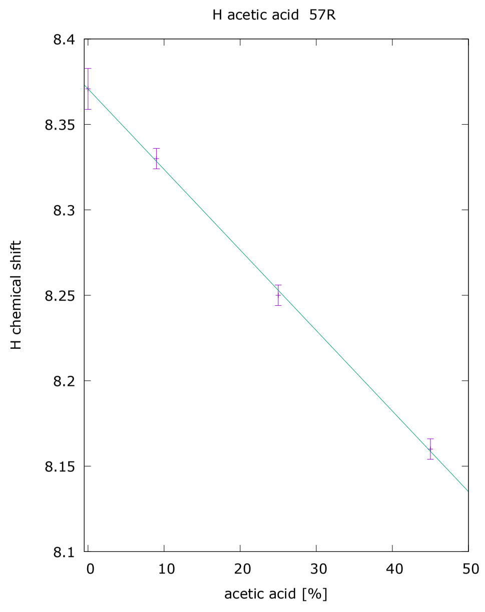
H acetic acid 57R
H chemical shift
acetic acid [%]

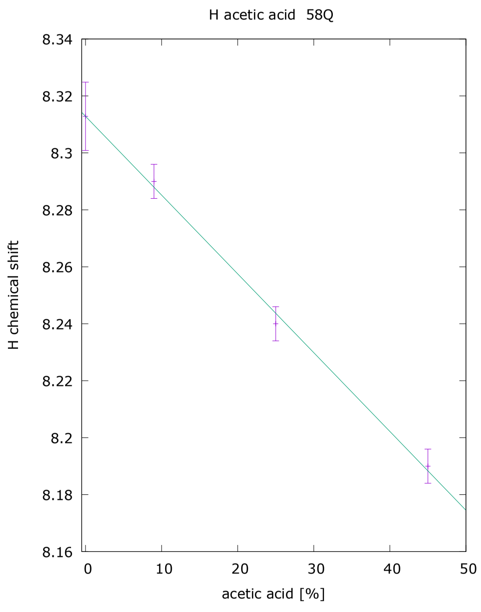
H acetic acid 58Q
H chemical shift
acetic acid [%]

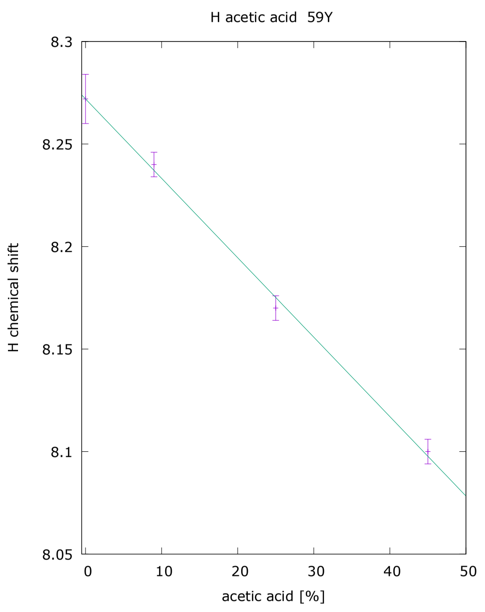
H acetic acid 59Y
H chemical shift
acetic acid [%]

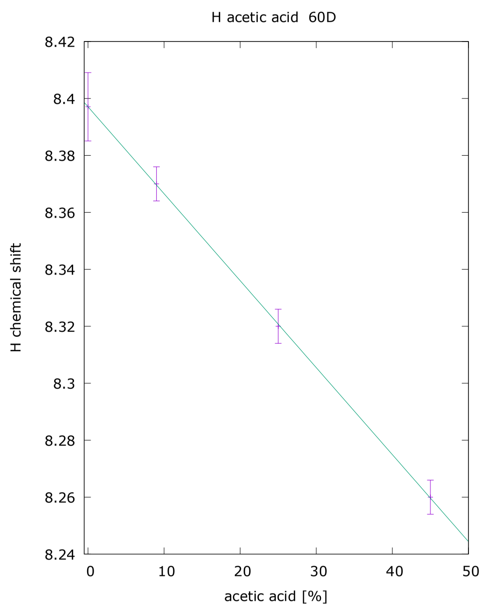
H acetic acid 60D
H chemical shift
acetic acid [%]

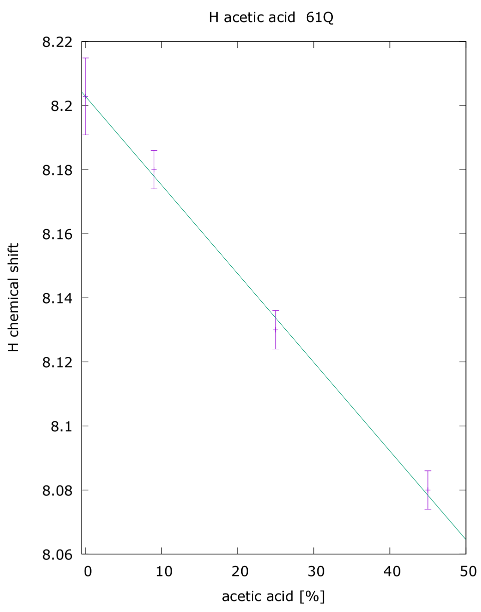
H acetic acid 61Q
H chemical shift
acetic acid [%]

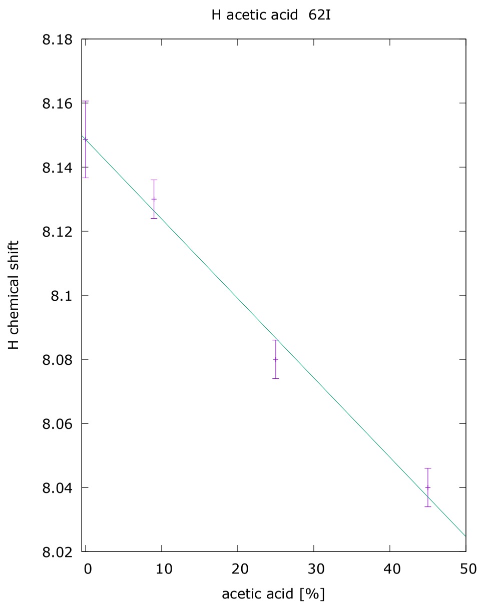
H acetic acid 62I
H chemical shift
acetic acid [%]

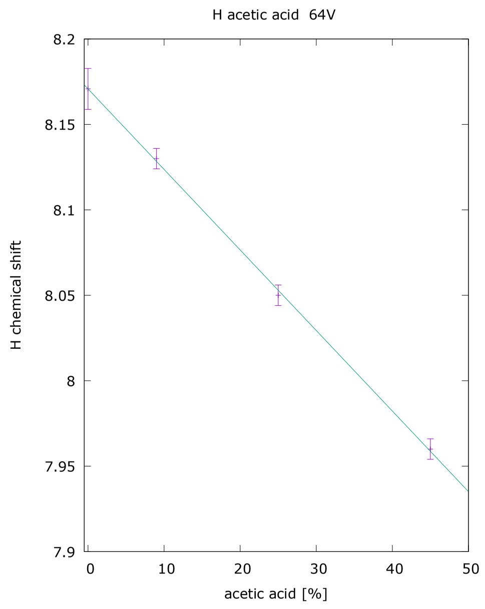
H acetic acid 64V
H chemical shift
acetic acid [%]

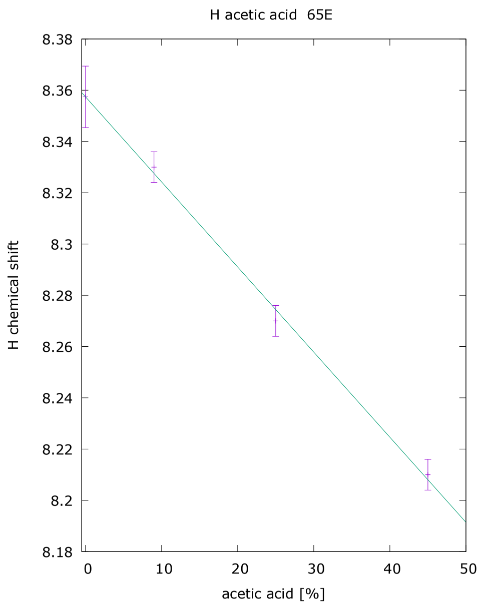
H acetic acid 65E
H chemical shift
acetic acid [%]

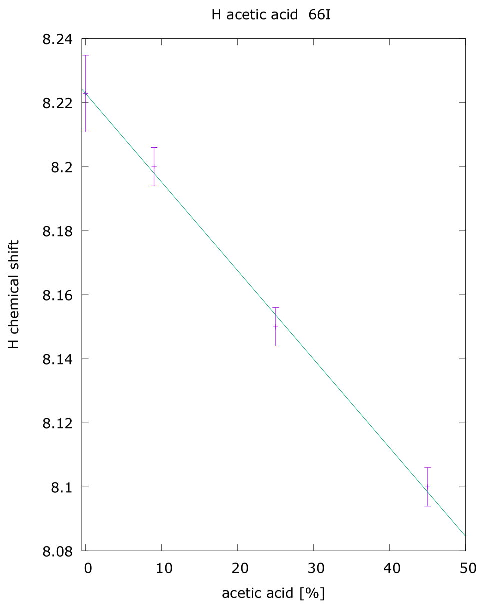
H acetic acid 66I
H chemical shift
8.24
8.22
8.2
8.18
8.16
8.14
8.12
8.1
8.08
0
10
20
30
40
50
acetic acid [%]

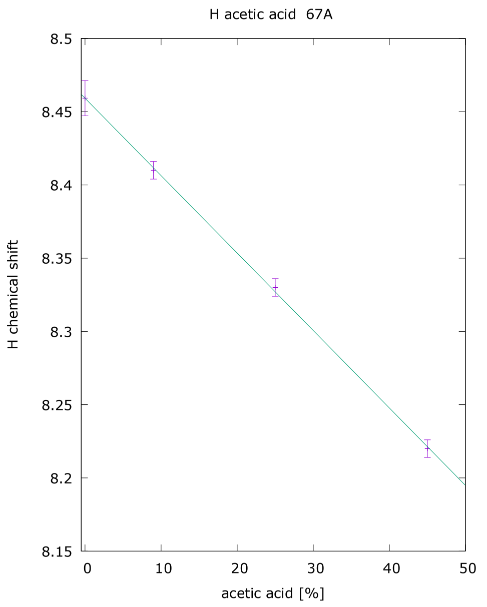
H acetic acid 67A
H chemical shift
8.5
8.45
8.4
8.35
8.3
8.25
8.2
8.15
0
10
20
30
40
50
acetic acid [%]

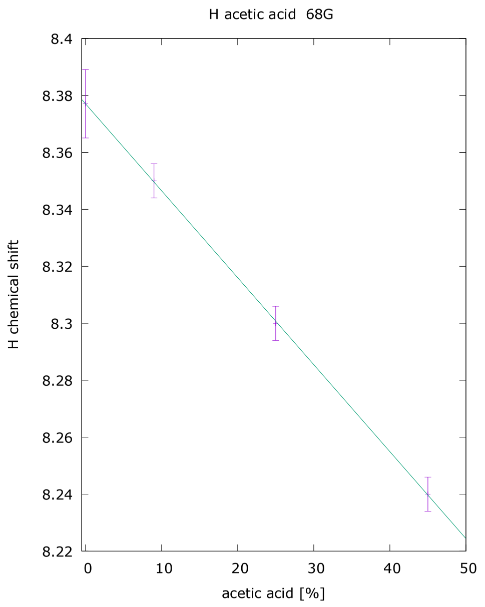
H acetic acid 68G
H chemical shift
8.4
8.38
8.36
8.34
8.32
8.3
8.28
8.26
8.24
8.22
0
10
20
30
40
50
acetic acid [%]

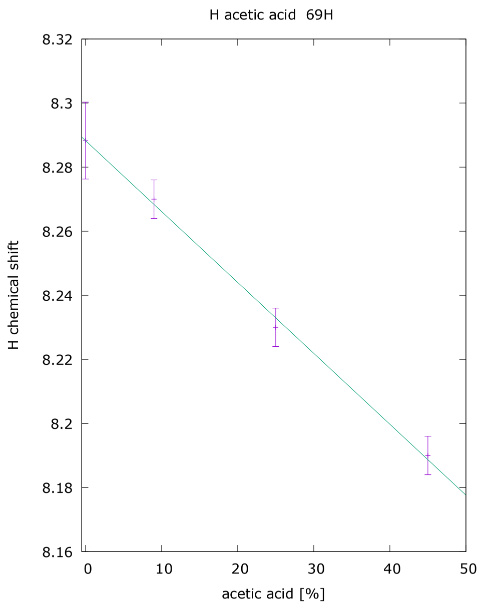
H acetic acid 69H
H chemical shift
8.32
8.3
8.28
8.26
8.24
8.22
8.2
8.18
8.16
0
10
20
30
40
50
acetic acid [%]

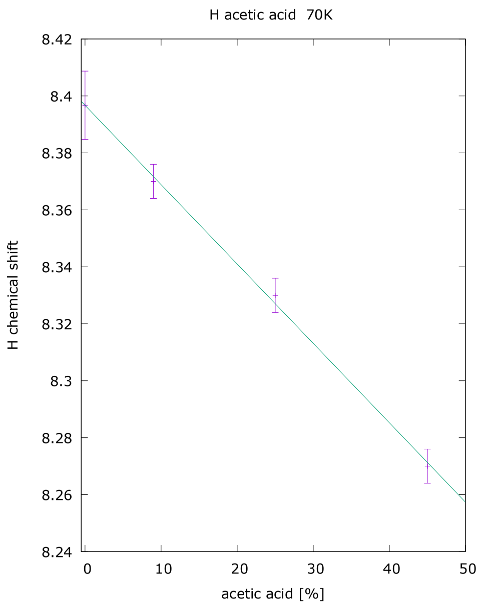
H acetic acid 70K
H chemical shift
8.42
8.4
8.38
8.36
8.34
8.32
8.3
8.28
8.26
8.24
0
10
20
30
40
50
acetic acid [%]

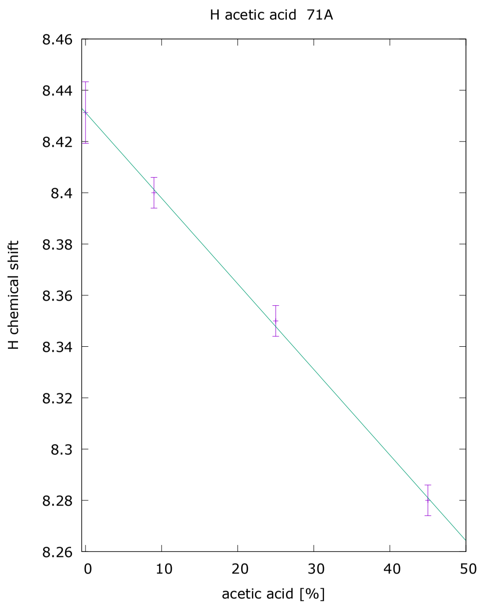
H acetic acid 71A
H chemical shift
8.46
8.44
8.42
8.4
8.38
8.36
8.34
8.32
8.3
8.28
8.26
0
10
20
30
40
50
acetic acid [%]

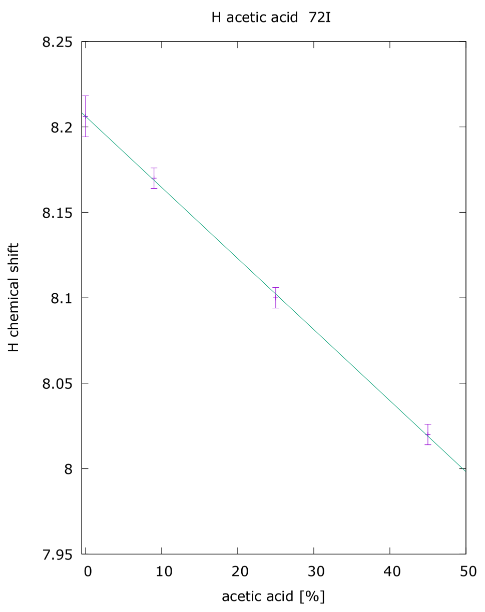
H acetic acid 72I
H chemical shift
acetic acid [%]

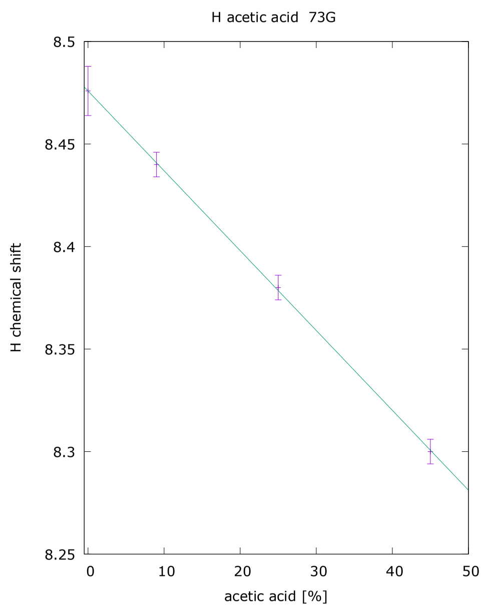
H acetic acid 73G
H chemical shift
acetic acid [%]

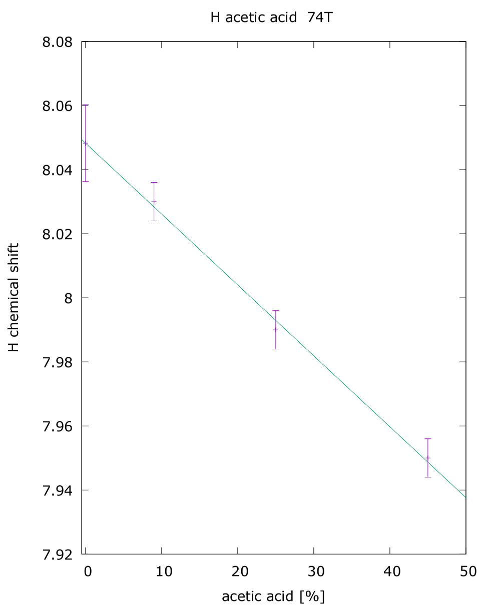
H acetic acid 74T
H chemical shift
acetic acid [%]

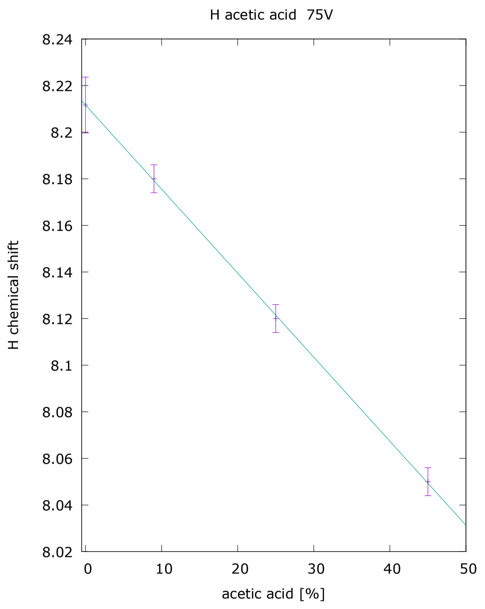
H acetic acid 75V
H chemical shift
acetic acid [%]

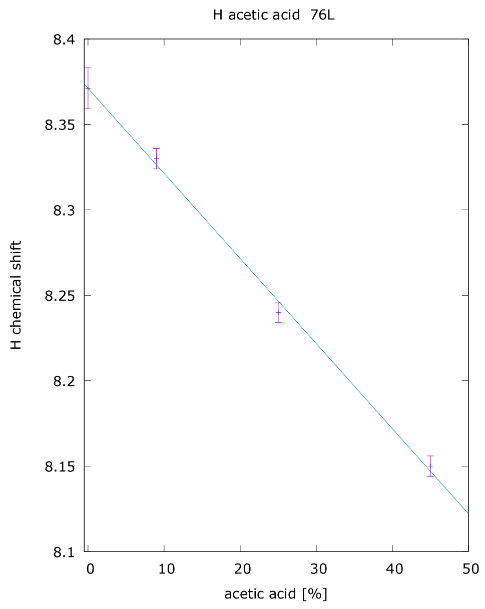
H acetic acid 76L
H chemical shift
acetic acid [%]

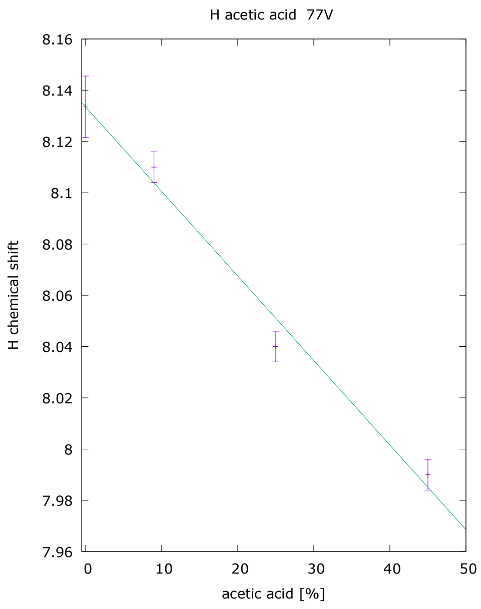
H acetic acid 77V
H chemical shift
acetic acid [%]

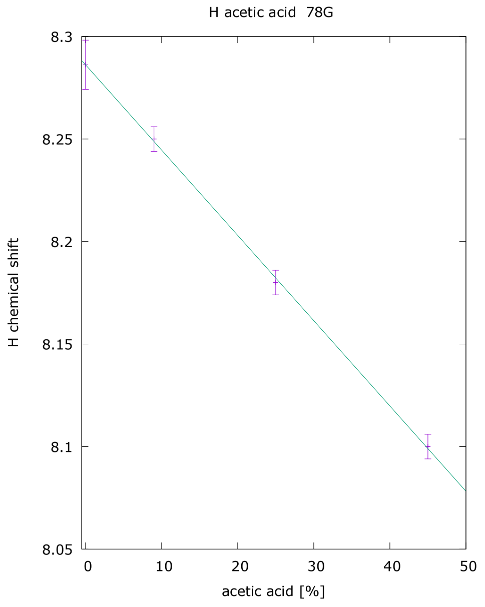
H acetic acid 78G
H chemical shift
acetic acid [%]

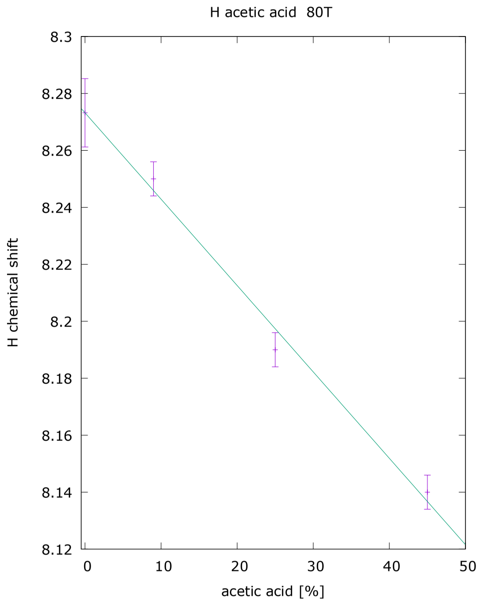
H acetic acid 80T
H chemical shift
acetic acid [%]

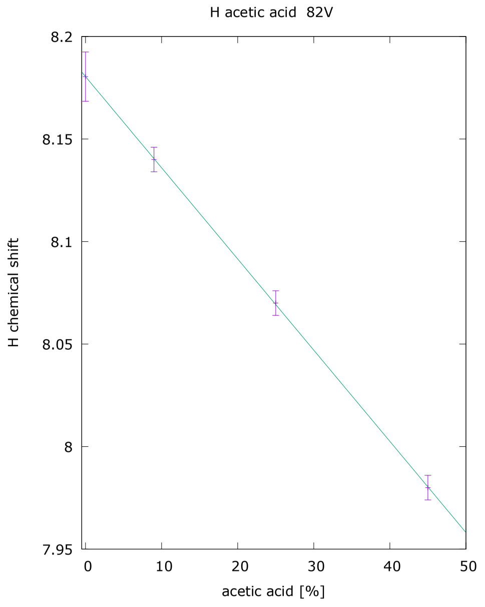
H acetic acid 82V
H chemical shift
acetic acid [%]

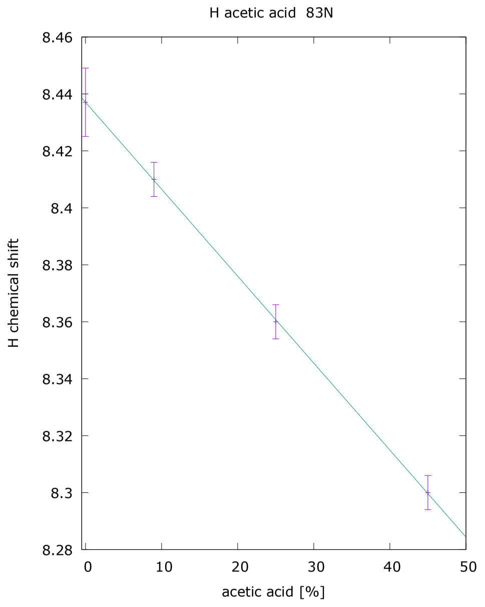
H acetic acid 83N
H chemical shift
acetic acid [%]

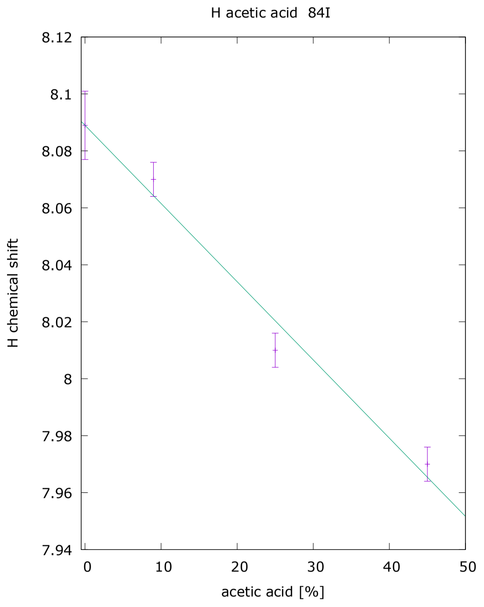
H acetic acid 84I
H chemical shift
acetic acid [%]

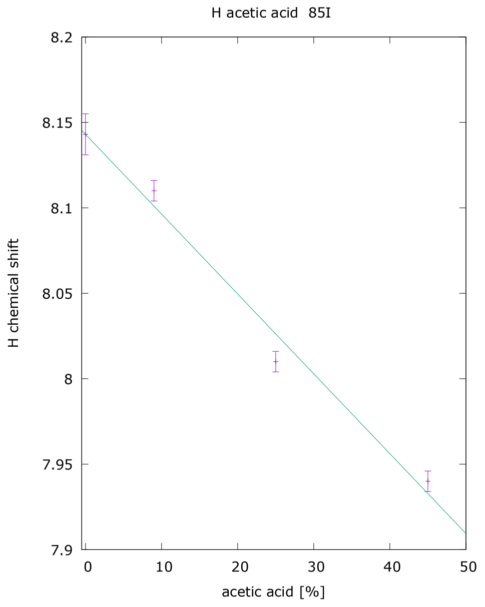
H acetic acid 85I
H chemical shift
acetic acid [%]

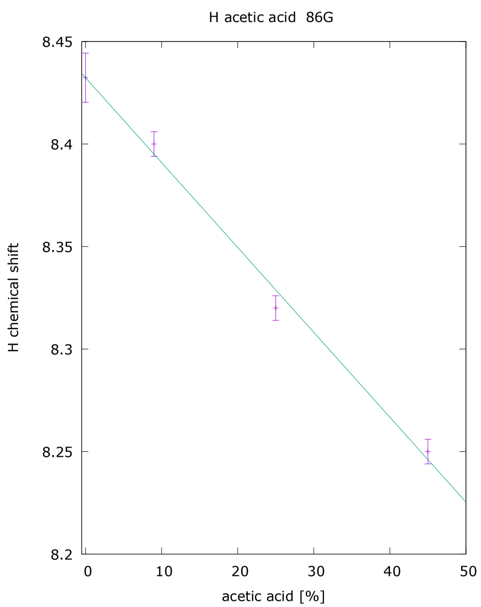
H acetic acid 86G
H chemical shift
8.45
8.4
8.35
8.3
8.25
8.2
0
10
20
30
40
50
acetic acid [%]

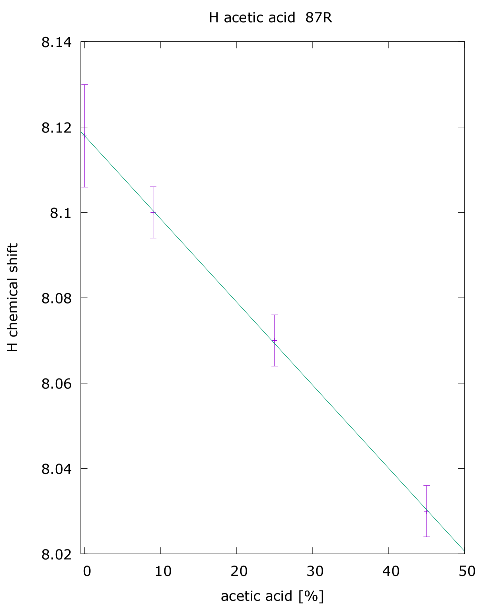
H acetic acid 87R
H chemical shift
8.14
8.12
8.1
8.08
8.06
8.04
8.02
0
10
20
30
40
50
acetic acid [%]

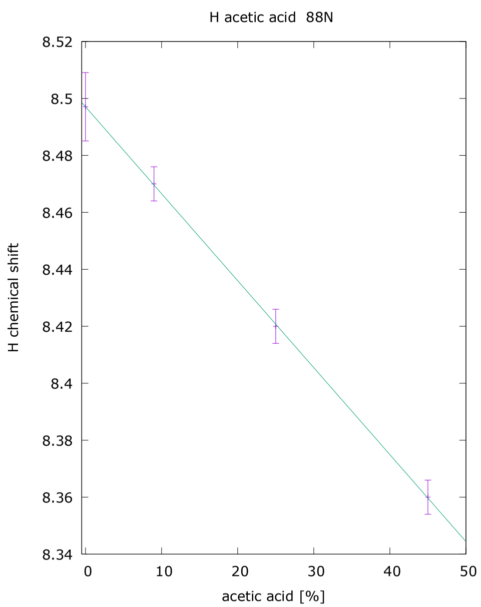
H acetic acid 88N
H chemical shift
8.52
8.5
8.48
8.46
8.44
8.42
8.4
8.38
8.36
8.34
0
10
20
30
40
50
acetic acid [%]

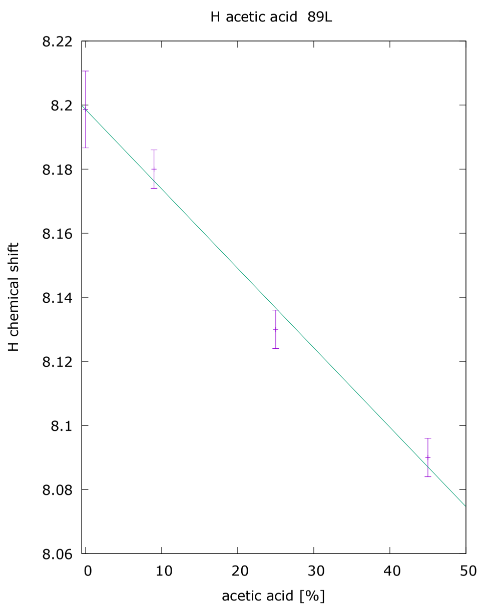
H acetic acid 89L
H chemical shift
8.22
8.2
8.18
8.16
8.14
8.12
8.1
8.08
8.06
0
10
20
30
40
50
acetic acid [%]

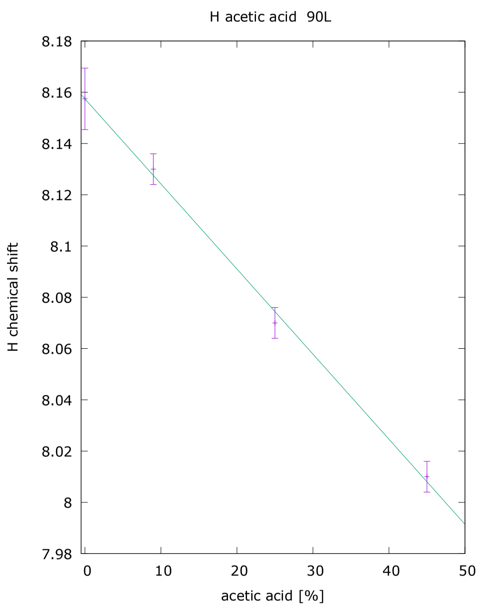
H acetic acid 90L
H chemical shift
8.18
8.16
8.14
8.12
8.1
8.08
8.06
8.04
8.02
8
7.98
0
10
20
30
40
50
acetic acid [%]

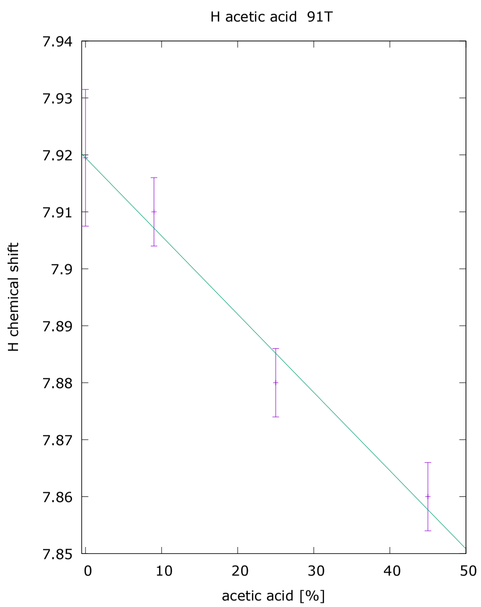
H acetic acid 91T
H chemical shift
7.94
7.93
7.92
7.91
7.9
7.89
7.88
7.87
7.86
7.85
0
10
20
30
40
50
acetic acid [%]

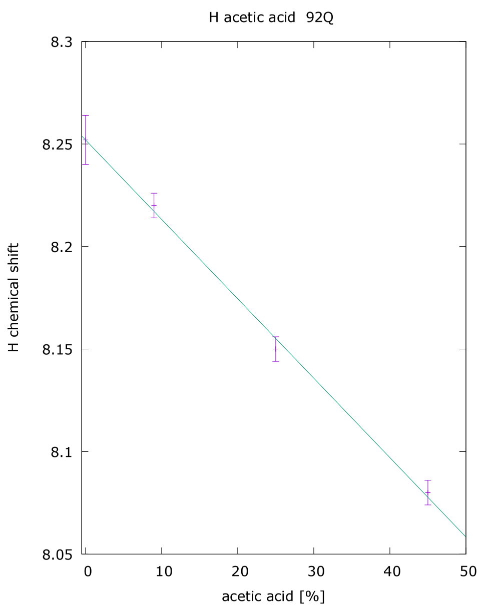
H acetic acid 92Q
H chemical shift
acetic acid [%]

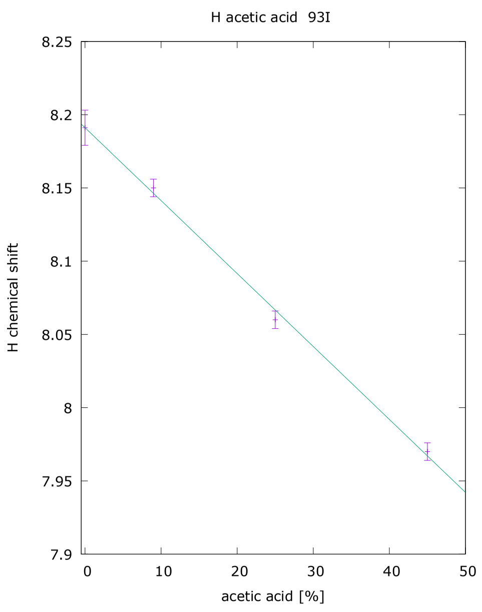
H acetic acid 93I
H chemical shift
acetic acid [%]

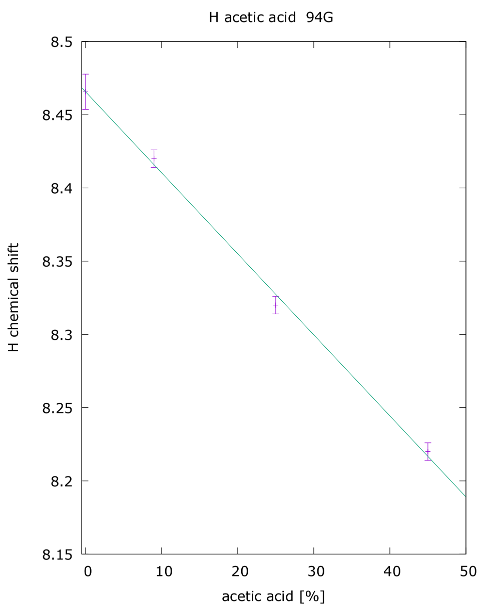
H acetic acid 94G
H chemical shift
acetic acid [%]

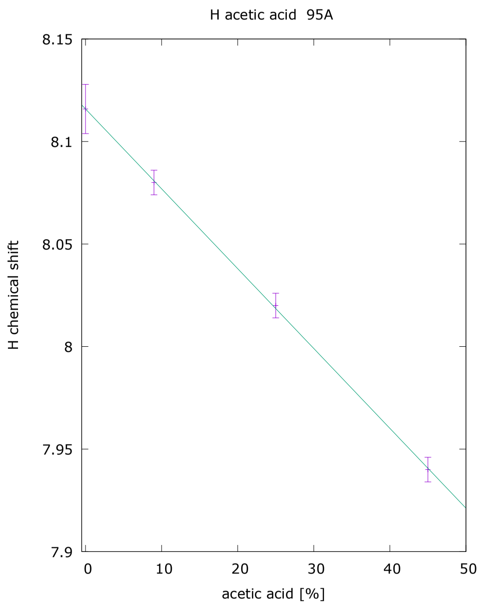
H acetic acid 95A
H chemical shift
acetic acid [%]

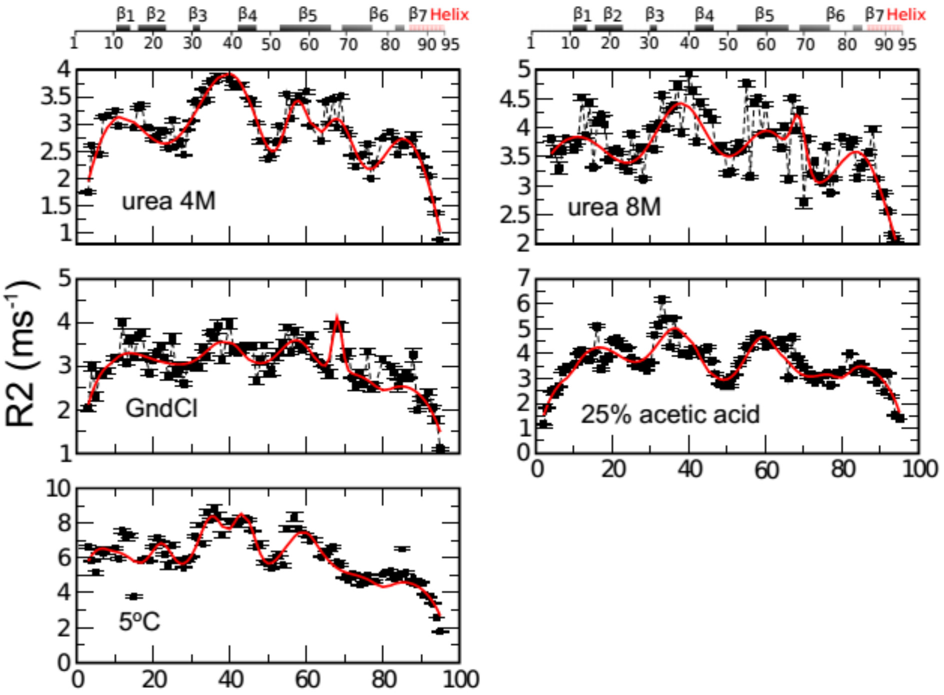

Figure S7: Transverse relaxation rates under different conditions (black dots) and least-square multi-exponential fit (red curve). To avoid overfitting, we performed fits with different number of exponentials, eventually choosing the minimum number of exponentials which gave a chi^2 lower than 5. We could fit 6 (5 °C), 5 (4 M urea), 5 (8 M urea), 4 (1M GdnHCl) and 4 (25% acetic acid) clusters for the *R_2* relaxation rates. Cluster 1 was split up, for reasons of comparison, into clusters (1a and 1b) comprising residues P1-R8 and P9-L24. Clusters 2 and 5 were centered round one (P38) and two prolines (P79, P81), respectively. Clusters 3 and 4 were split by a single proline (P63).

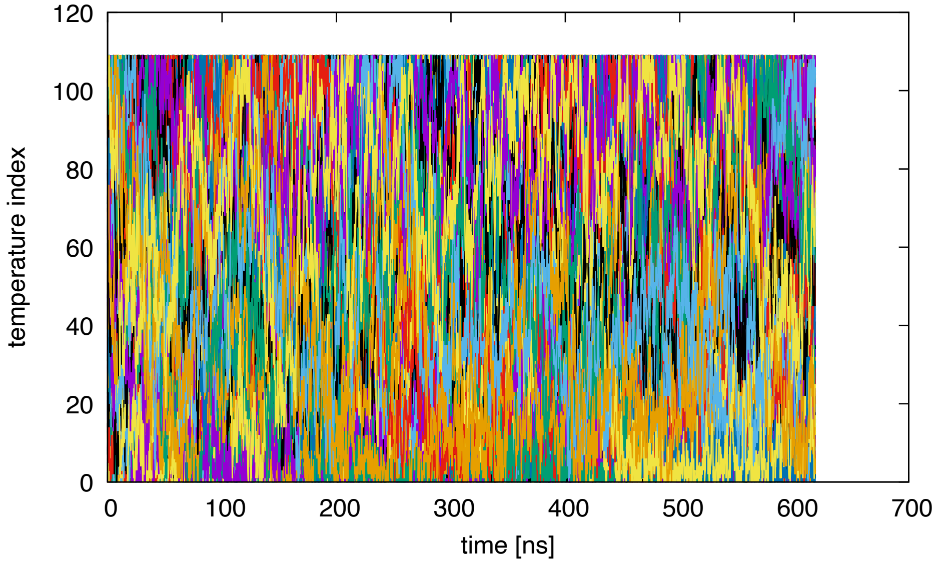

Figure S8: the variation of temperature of the different replicas during the MD simulation.

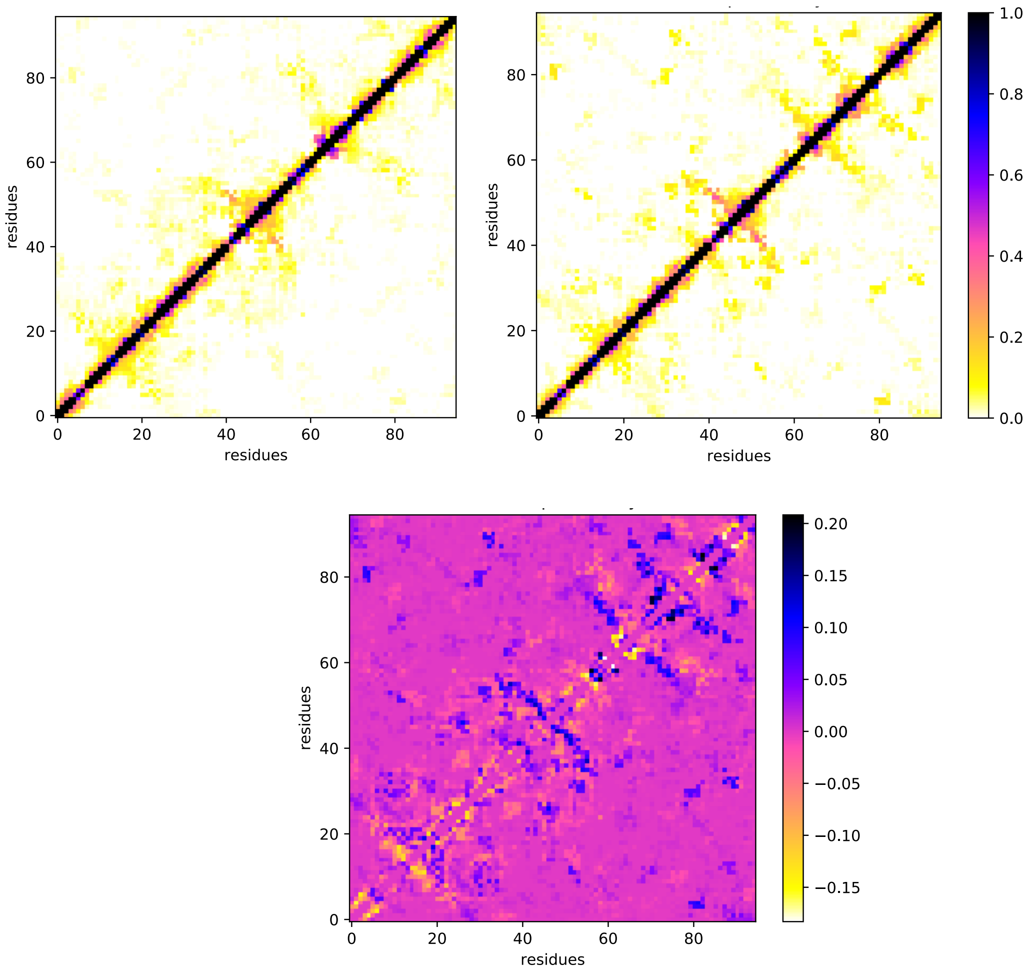

Figure S9: The simulated contact probability between residues of mHIV-1, calculated using only the first half of the simulation (upper-left panel), using only the second half (upper-right panel), and the difference between the two (lower panel).

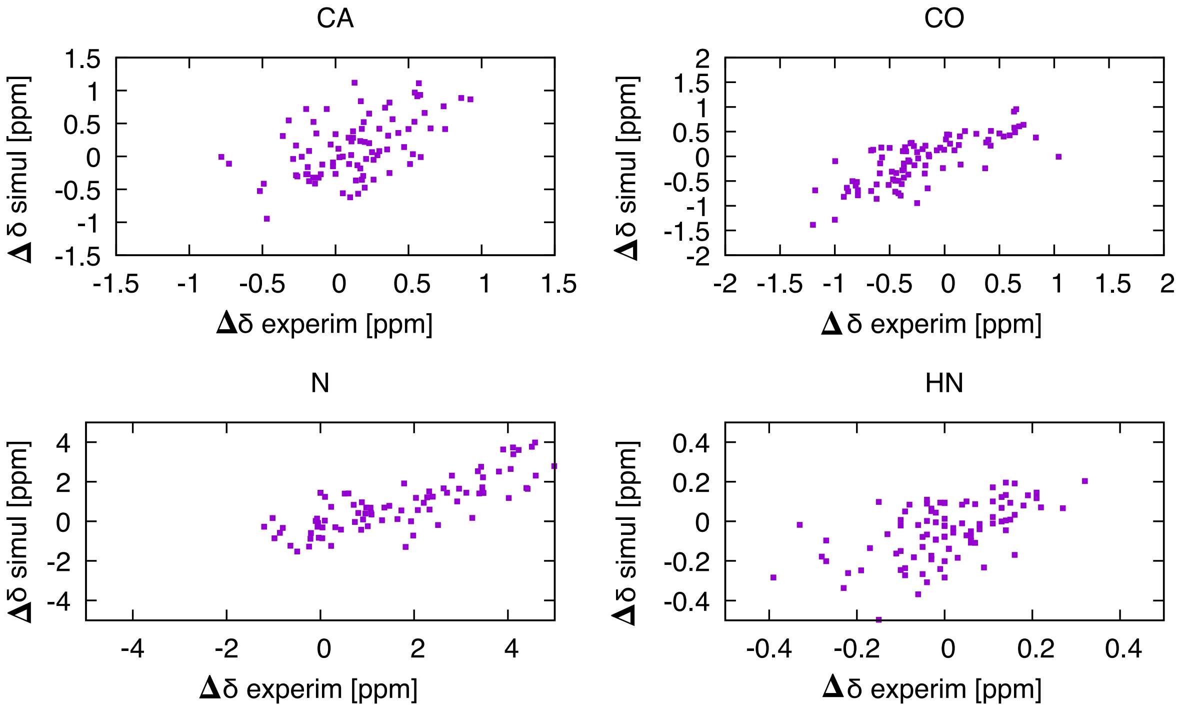

Figure S10: Scatter plots of the simulated secondary chemical shifts versus the experimental values extrapolated at zero denaturant.

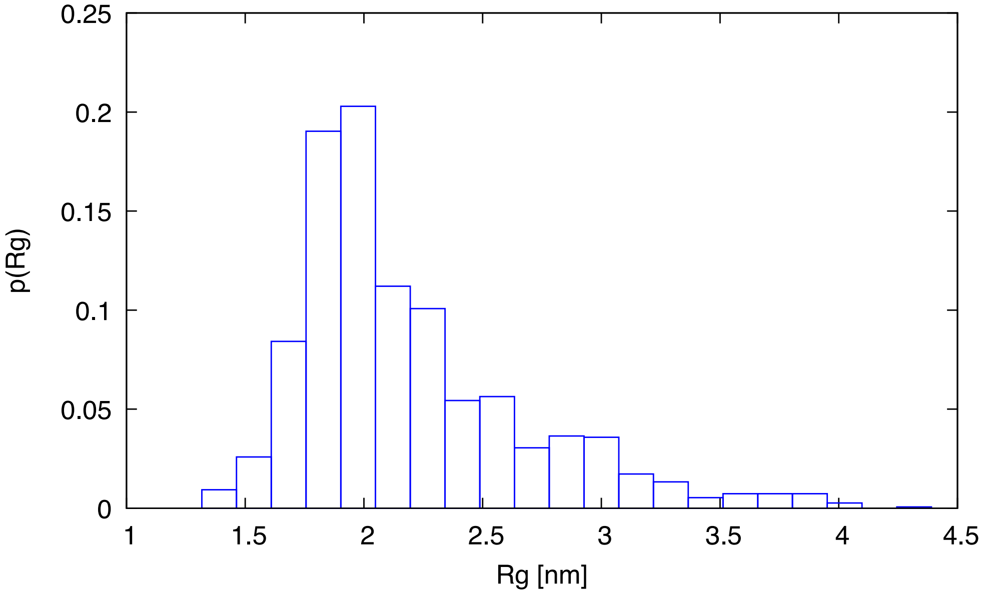

Figure S11: Distribution of the radius of gyration of D_0 obtained from the simulation.

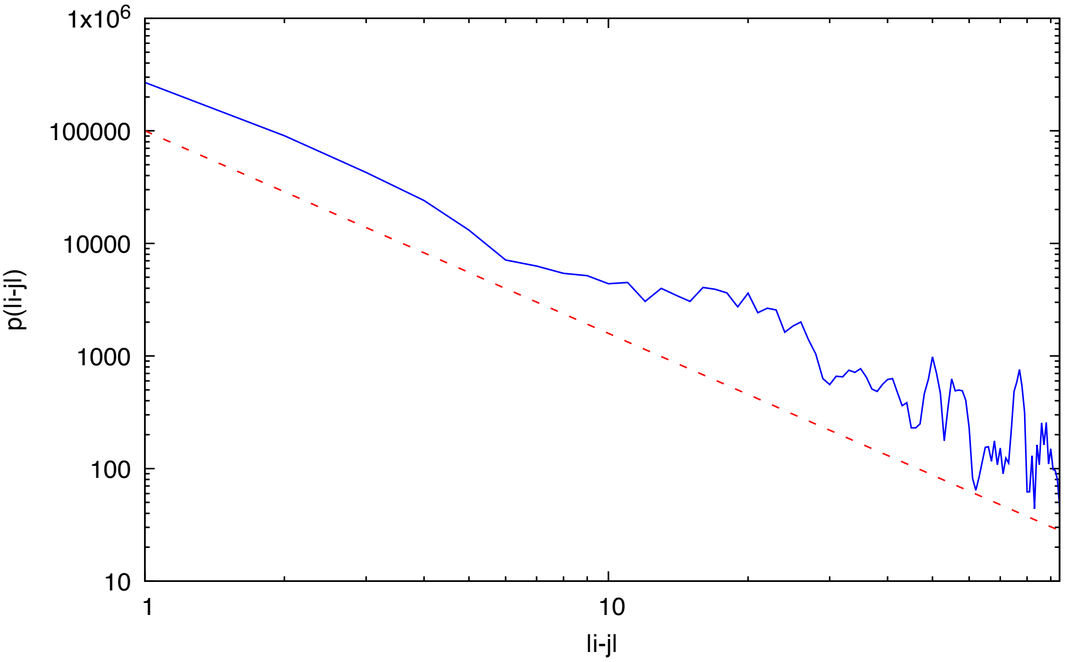

Figure S12: Contact probability in log-log scale between residues as a function of their distance along the chain. The dashed line indicates a power law with power -1.8.

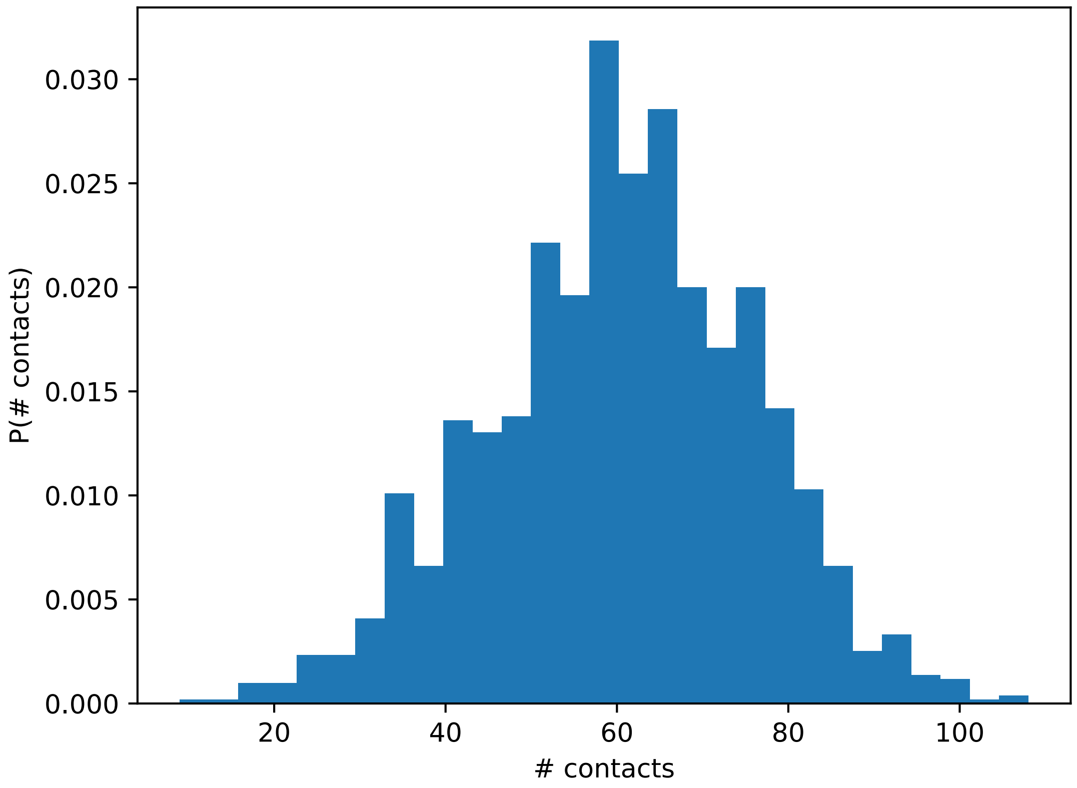

Figure S13: The distribution of number of contacts in the conformations sampled by the simulation at 300K.

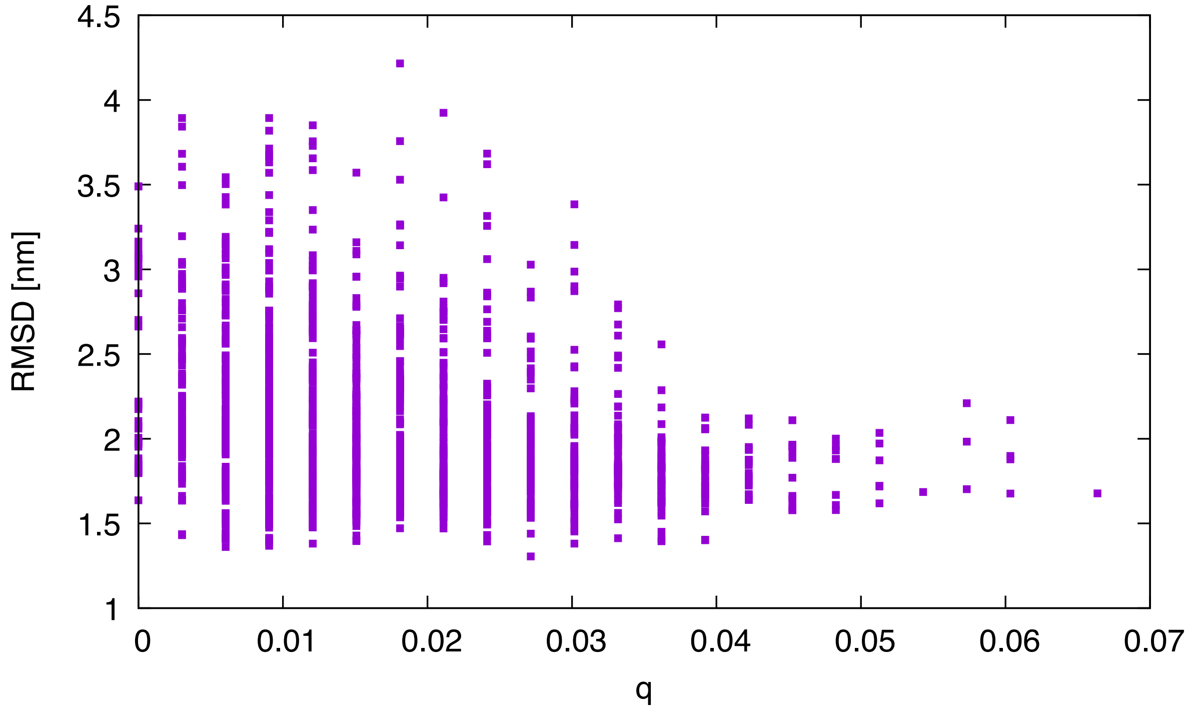

Figure S14: The RMSD of the sampled conformations with respect to the native conformation plotted versus the fraction q_N of native contacts.

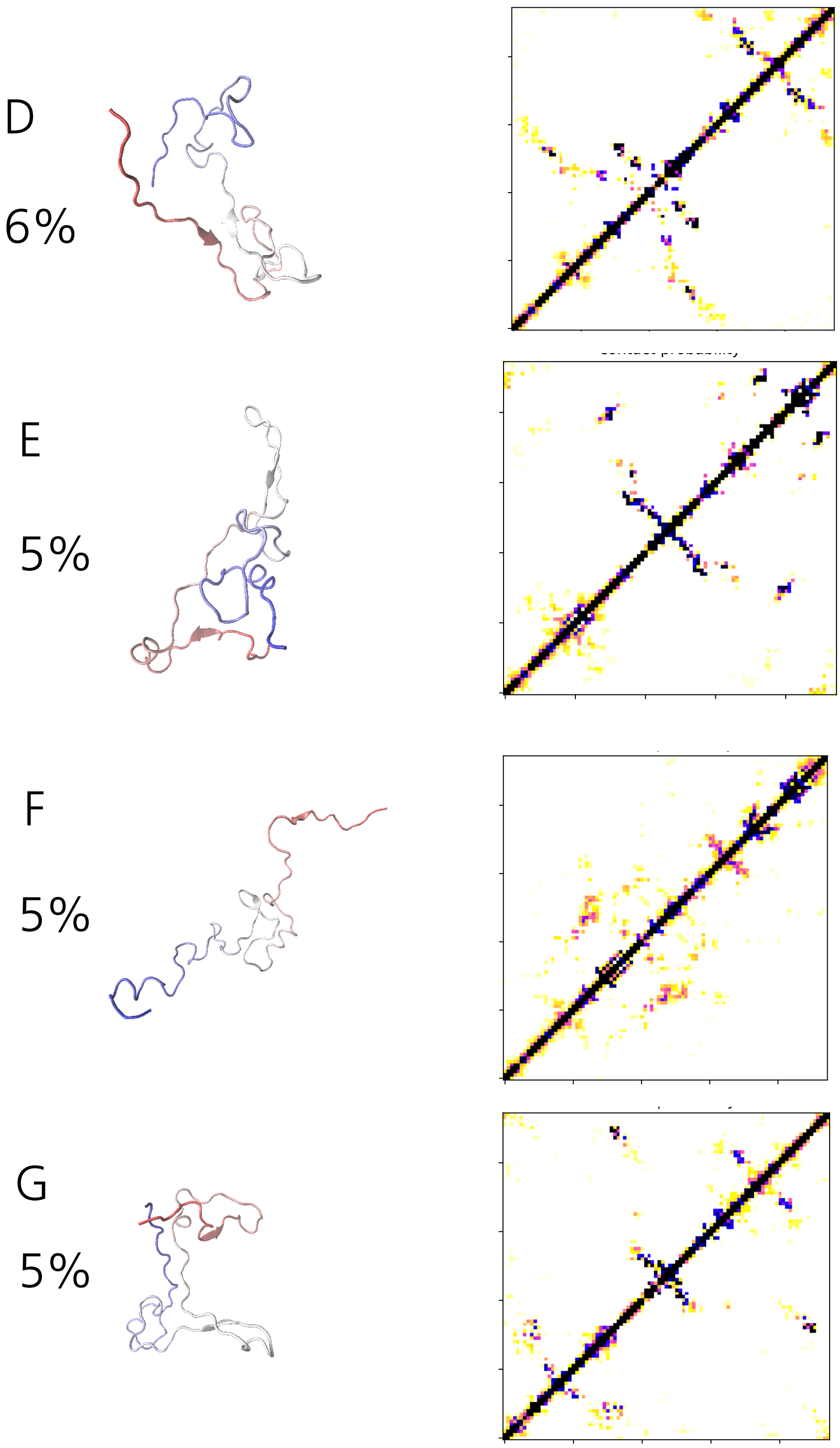

Figure S15: Some minority clusters. The central conformation is shown together with the mean contact map and the relative population of the cluster.
